# Disease Burden, Risk Factors, and Trends of Leukaemia: A Global Analysis

**DOI:** 10.3389/fonc.2022.904292

**Published:** 2022-07-22

**Authors:** Junjie Huang, Sze Chai Chan, Chun Ho Ngai, Veeleah Lok, Lin Zhang, Don Eliseo Lucero-Prisno, Wanghong Xu, Zhi-Jie Zheng, Edmar Elcarte, Mellissa Withers, Martin C. S. Wong

**Affiliations:** ^1^ The Jockey Club School of Public Health and Primary Care, Faculty of Medicine, Chinese University of Hong Kong, Hong Kong, Hong Kong SAR, China; ^2^ Department of Global Public Health, Karolinska Institute, Karolinska University Hospital, Stockholm, Sweden; ^3^ School of Population and Global Health, The University of Melbourne, Carlton, VIC, Australia; ^4^ School of Public Health, The Chinese Academy of Medical Sciences and Peking Union Medical College, Beijing, China; ^5^ Department of Global Health and Development, London School of Hygiene and Tropical Medicine, London, United Kingdom; ^6^ School of Public Health, Fudan University, Shanghai, China; ^7^ Department of Global Health, School of Public Health, Peking University, Beijing, China; ^8^ College of Nursing, University of the Philippines, Manila, Philippines; ^9^ Department of Preventive Medicine, Institute for Global Health, University of Southern California, Los Angeles, CA, United States

**Keywords:** Leukaemia, incidence, mortality, risk factors, temporal trend

## Abstract

Leukaemia accounted for approximately 2.5% of all new cancer incidence and 3.1% of cancer-related mortality. The investigation of its risk factors and epidemiologic trends could help describe the geographical distribution and identify high-risk population groups. This study aimed to evaluate the global incidence, mortality, associated risk factors, and temporal trends of leukaemia by sex, age, and country. We extracted incidence and mortality of leukaemia from *GLOBOCAN*, *CI5*, *WHO mortality database*, *NORDCAN*, and *SEER*. We searched the *WHO Global Health Observatory* data repository for the age-standardised prevalence of lifestyle and metabolic risk factors. We tested the trends by calculating Average Annual Percentage Change (AAPC) from Joinpoint regression. The age-standardized rate of incidence and mortality were 5.4 and 3.3 per 100,000 globally. The incidence and mortality of leukaemia were associated with Human Development Index, Gross Domestics Products per capita, prevalence of smoking, physical activity, overweight, obesity, and hypercholesterolaemia at the country level. Overall, more countries were showing decreasing trends than increasing trends in incidence and mortality. However, an increasing trend of leukaemia incidence was found in Germany, Korea, Japan, Canada and the United Kingdom (AAPC, 2.32-0.98) while its mortality increased in the Philippines, Ecuador, Belarus, and Thailand (AAPC, 2.49-1.23). There was a decreasing trend of leukaemia for the past decade while an increase in incidence and mortality was observed in some populations. More intensive lifestyle modifications should be implemented to control the increasing trends of leukaemia in regions with these trends. Future studies may explore the reasons behind these epidemiological transitions.

## Introduction

Leukaemia is a blood-related malignancy characterized by transformed hematopoietic progenitors and diffuse infiltration of bone marrow. The main types of leukaemia include acute lymphoblastic leukaemia (ALL), acute myeloid leukaemia (AML), chronic lymphocytic leukaemia (CLL), and chronic myeloid leukaemia (CML). Globally in 2020, leukaemia accounted for approximately 2.5% and 3.1% of all new cancer incidence and mortality, respectively (1). The risk of leukaemia varies among populations of different ages, sexes, and geographical locations (2). Such disparities could be attributable to the difference in the prevalence of different environmental and genetic risk factors for leukaemia.

Risk factors for leukaemia include smoking, exposure to certain chemicals, chemotherapy in the past, radiation exposure, rare congenital diseases, certain blood disorders, family history, age, and gender (3). Due to the recent development of novel therapeutic strategies and targeted drugs, the overall survival of leukaemia patients has shown remarkable improvements (4). The epidemiology of leukaemia may have changed over time and may vary by different population groups. Therefore, it is imperative to examine the global disease distribution, risk factors, and trends of leukaemia to inform the development of its preventive strategies tailored for different countries.

Prior studies are limited to certain countries or captured temporal trends using relatively old data (5–7). Furthermore, none comprehensively determined the lifestyle and metabolic risk factors for leukaemia at a country level. This study aims to: 1) investigate the most updated global incidence and mortality of leukaemia by region, sex, and income level 2) explore the global dietary and socioeconomic factors in differentiating trends in leukaemia incidence and mortality worldwide; and 3) examine the recent incidence and mortality trends of leukaemia for the recent past decade among groups of different ages, sexes, and countries.

## Methods

### Data Sources

To retrieve updated statistics on cancer on a global scale, various databases were accessed and explored. The *GLOBOCAN* database was accessed for comprehensive records of the most updated incidence and mortality of leukaemia for 185 countries (8). The Human Development Index (HDI) for each country was extracted from the United Nations (9). Data on gross domestic products (GDP) per capita were retrieved from the World Bank. The prevalence of risk factors for each country was collected from the *Global Health Observatory* (*GHO*) (10), including the prevalence of current smoking, physical inactivity, overweight, obesity, diabetes, and hypercholesterolaemia. For trend analysis, data from the yearly incidence of leukaemia was extracted from the *Cancer Incidence in Five Continents I-X plus (CI5Plus)* for 48 countries. *CI5* is a global cancer database developed by the International Association of Cancer Registries, where the age and sex-associated cancer incidence from different countries can be found to facilitate direct comparison of cancer incidence based on demographic characteristics (11). Data on leukaemia mortality were retrieved from the WHO Mortality Database, where the number of cancer-related deaths is collected (12). In addition, the *Nordic Cancer Registries (NORDCAN) (*13 14) and the *Surveillance, Epidemiology, and End Results (SEER) (* 13) were retrieved to obtain the latest leukaemia incidence and mortality data of Northern European countries and the United States, respectively. In our analysis, leukaemia was defined using the International Classification of Diseases 10^th^ revision (ICD-10) C91-95. The countries were divided into nine regions in the trend analysis for presentation, including Asia, Oceania, Northern America, Southern America, Northern Europe, Western Europe, Southern Europe, Eastern Europe, and Africa. For easier reference and comparison, incidence and mortality across countries and age groups were presented in the form of age-standardized rates (ASRs) per 100,000 after adjustment according to the Segi-Doll standardized population.

### Statistical Analysis

To present the global incidence and mortality of leukaemia, choropleth maps were constructed. Associations between HDI, GDP per capita, and potential risk factors and incidence and mortality of leukaemia for each country were examined by univariable linear regression analysis for men and women separately. Beta coefficients (*β*) and the corresponding 95% confidence intervals (CI) were calculated from the regression. The *β* estimates measure the degree of change in ASR of incidence or mortality of leukaemia per unit increase in the prevalence of risk factors. The corresponding Average Annual Percentage Change (AAPC) for different regions and countries were then calculated for the temporal trend of cancer incidence and mortality of leukaemia on a global scale (14). In trend analysis with transitions, AAPC is preferred over annual percentage change (APC) because it considers the length of the time segment and it does not assume linearity (15). The AAPCs were estimated using Joinpoint regression analysis software, which is developed by the Surveillance, Epidemiology, and End Results Program (SEER) under the United States National Cancer Institute. As a normal practice in epidemiology research for cancer, data of a period of 10 years were used. The ASRs had undergone a logarithmic transformation and related standard errors had been calculated. They were then used to calculate the AAPC and the 95% Confidence Interval (CI) for all countries and both sexes. The epidemiological trends of incidence and mortality are indicated by the AAPC, with a positive AAPC indicating an increasing trend and vice versa. The 95% CI can be used as an indicator to assess the stability of the trend: an interval overlapping with 0 signifies a stable trend without significant temporal change. In this study, the incidence and mortality of the entire population were examined. The incidence rates of different age groups (below 15, between 15-49, 50 or above, and 0-85+) were compared to evaluate the role of ages; results from both sexes in each group were separately assessed to investigate the role of sex in leukaemia.

## Results

### Global Incidence of Leukaemia in 2020

**Figure 1:**
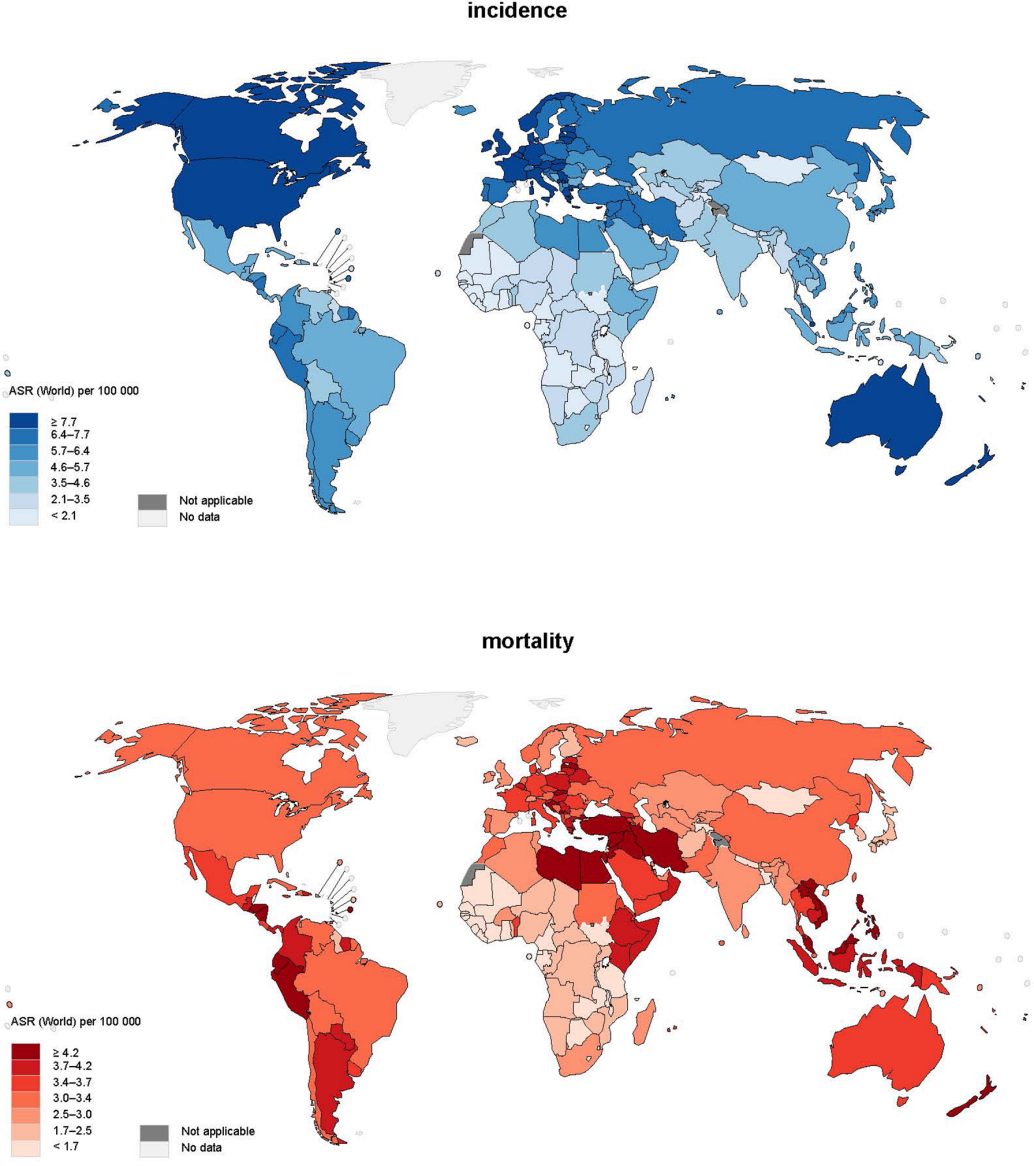
Global incidence and mortality of leukaemia, both sexes, all ages, in 2020.

A total of 474,519 new cases of leukaemia were reported in 2020 (**Figure 1**). The global age-standardized rate of incidence was 5.4 per 100,000 and there was an almost five-fold variation worldwide. North America (ASR = 10.9), Australia and New Zealand (ASR = 10.4), Western Europe (ASR = 8.5), and Northern Europe (ASR = 8.5) had the highest incidence, whereas the lowest incidence was found in Middle Africa (ASR = 2.2), Western Africa (ASR = 2.3), and Eastern Africa (ASR = 3.3). As far as the sex-specific age-standardized rate is concerned, the ASR of men (6.3) was 40% higher than that of women (4.5) worldwide, and larger differences could be found in regions with higher ASRs. Moreover, it was found that countries with higher income levels had a higher incidence; high-income countries (ASR=8.4) had incidence 1.5 times higher than low-income countries (ASR=3.4).

### Global Mortality of Leukaemia in 2020

In terms of mortality, 311,594 related deaths were reported in 2020. There was a smaller regional difference worldwide in mortality from leukaemia, as the majority of regions in Asia, Europe, America, and Australia and New Zealand reported mortality of around 2.5-4.0 per 100,000. It is worth mentioning that Western Asia (ASR = 4.6) had the highest mortality, 40% higher than the world average (ASR = 3.3). Sex was also a pivotal factor in mortality as men (ASR = 4.0) had a mortality of almost 50% higher than women (ASR = 2.7). Regarding the discrepancy in mortality among countries with different levels of income, countries with higher income [upper-middle income (ASR = 3.6), high income (ASR = 3.2)] had around 30% higher risk than lower income (low income (ASR = 2.8) and low-middle income (ASR = 2.7) countries).

### Associations Between Risk Factors and Burden of Leukaemia

**Figure 2:**
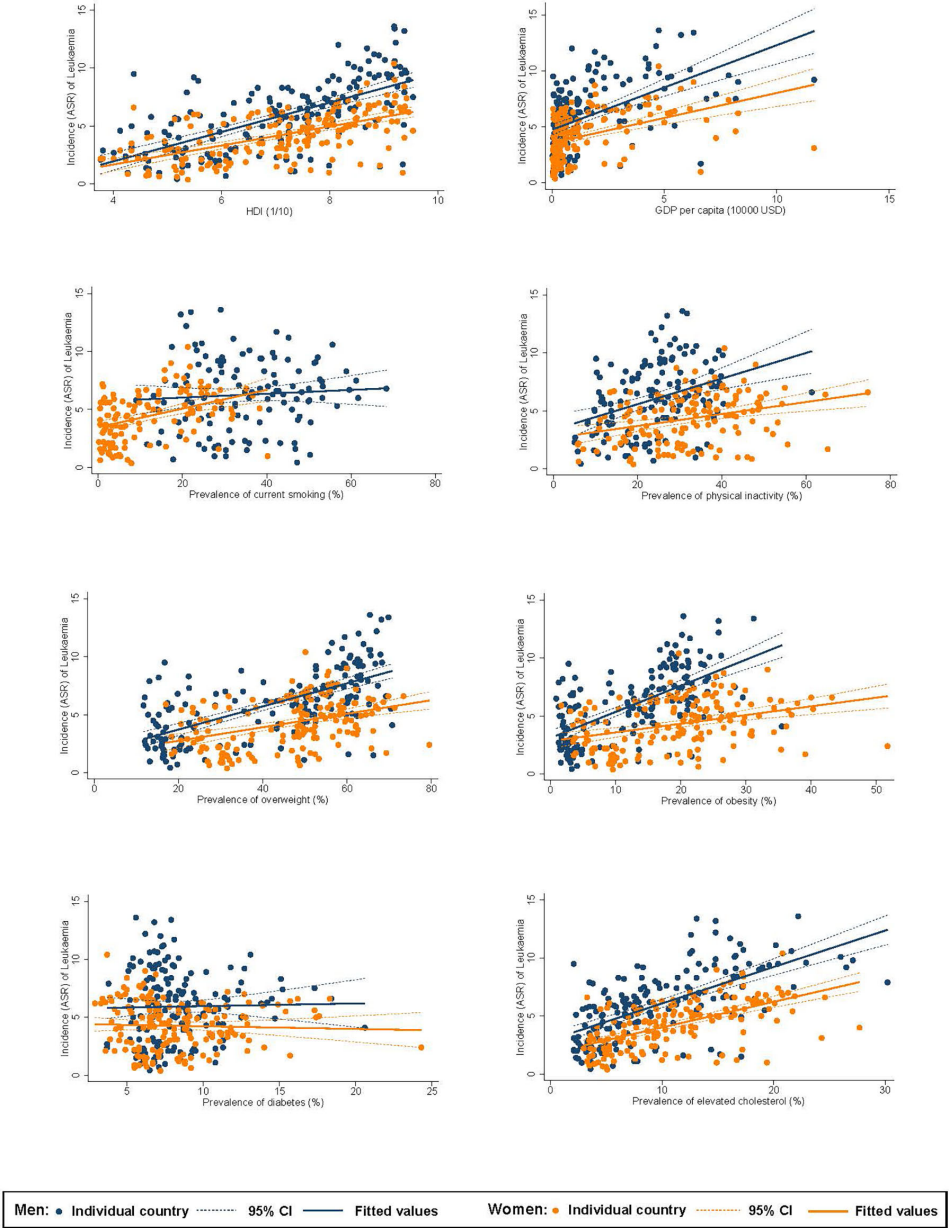
Associations between risk factors and incidence of leukaemia.

**Figure 3:**
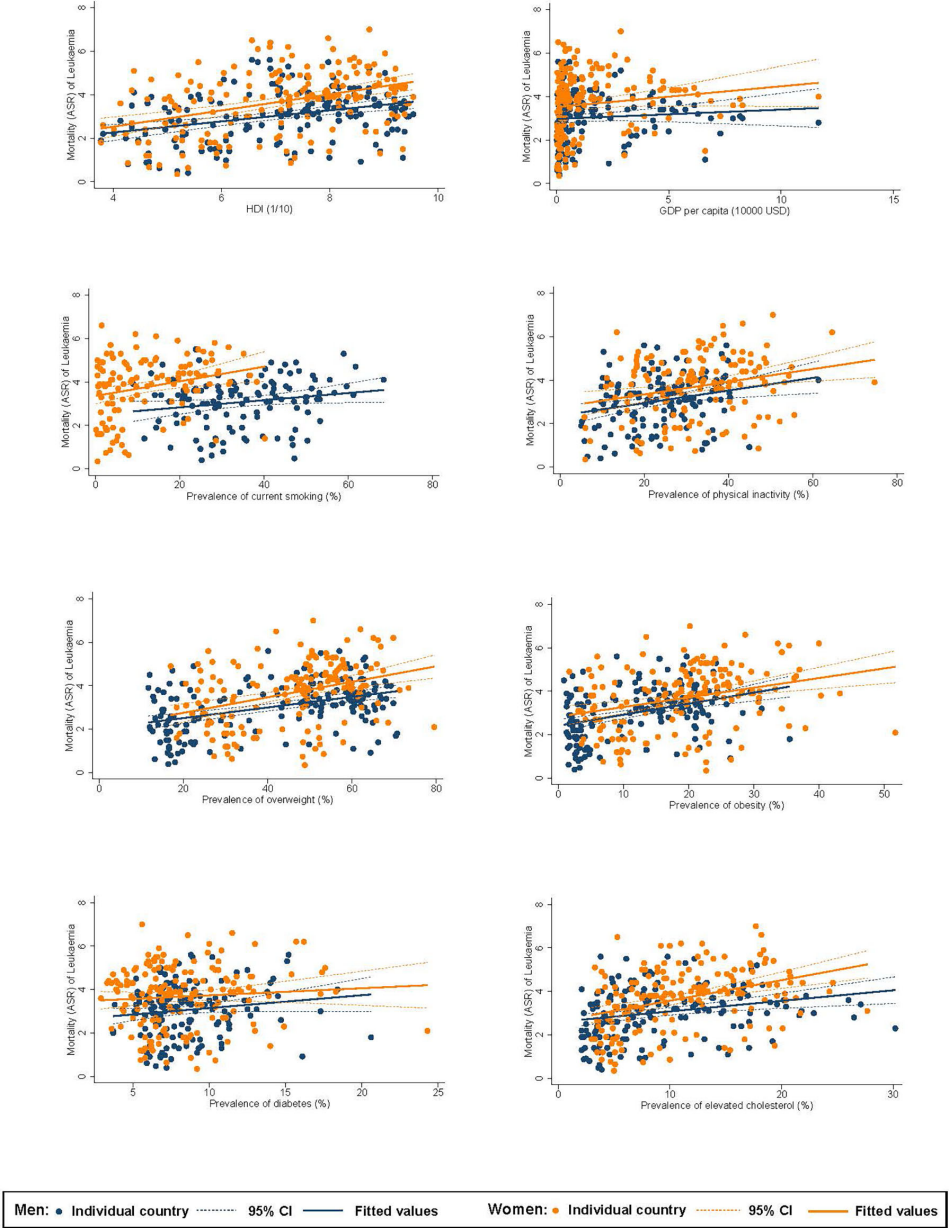
Associations between risk factors and mortality of leukaemia.

Among men, higher ASR of incidence of leukaemia was associated with a higher HDI (*β*=1.27, CI 1.05 to 1.49), GDP per capita (*β*=0.75, CI 0.56 to 0.95), and higher prevalence of inactivity (*β*=0.11, CI 0.06 to 0.16), overweight (*β*=0.10, CI 0.08 to 0.12), obesity (*β*=0.23, CI 0.18 to 0.27), and hypercholesterolaemia (*β*=0.31, CI 0.26 to 0.37; **Figure 2**). For women, higher incidence was associated with a higher HDI (*β*=0.82, CI 0.66 to 0.98), GDP per capita (*β*=0.45, CI 0.31 to 0.58), and higher prevalence of smoking (*β*=0.08, CI 0.05 to 0.11), inactivity (*β*=0.05, CI 0.03 to 0.08), overweight (*β*=0.06, CI 0.04 to 0.08), obesity (*β*=0.08, CI 0.04 to 0.11), and hypercholesterolaemia (*β*=0.22, CI 0.18 to 0.27). Among men, higher ASR of mortality of leukaemia was associated with a higher HDI (*β*=0.25, CI 0.15 to 0.36), and higher prevalence of smoking (*β*=0.02, CI 0.001 to 0.03), inactivity (*β*=0.03, CI 0.01 to 0.05), overweight (*β*=0.02, CI 0.02 to 0.03), obesity (*β*=0.05, CI 0.03 to 0.07), and hypercholesterolaemia (*β*=0.05, CI 0.02 to 0.08; **Figure 3**). For women, higher mortality was associated with a higher HDI (*β*=0.37, CI 0.24 to 0.50) and higher prevalence of smoking (*β*=0.03, CI 0.01 to 0.06), inactivity (*β*=0.03, CI 0.01 to 0.05), overweight (*β*=0.04, CI 0.02 to 0.05), obesity (*β*=0.04, CI 0.02 to 0.07), and hypercholesterolaemia (*β*=0.09, CI 0.06 to 0.13).

### Temporal Trends of Leukaemia

The incidence and mortality trends of leukaemia for each country between 1980 and 2017 are shown in **Supplementary Figure 1**, and the trend regression is presented in **Supplementary Figure 2**. Overall, more countries were showing decreasing trends than increasing trends in incidence in all age groups and both sexes, and such discrepancy was particularly significant for women. As for mortality, more countries were showing decreasing trends than increasing trends for both sexes, and this discrepancy was particularly significant for European countries.

### Incidence Trends of Individuals Aged 0-85+

**Figure 4:**
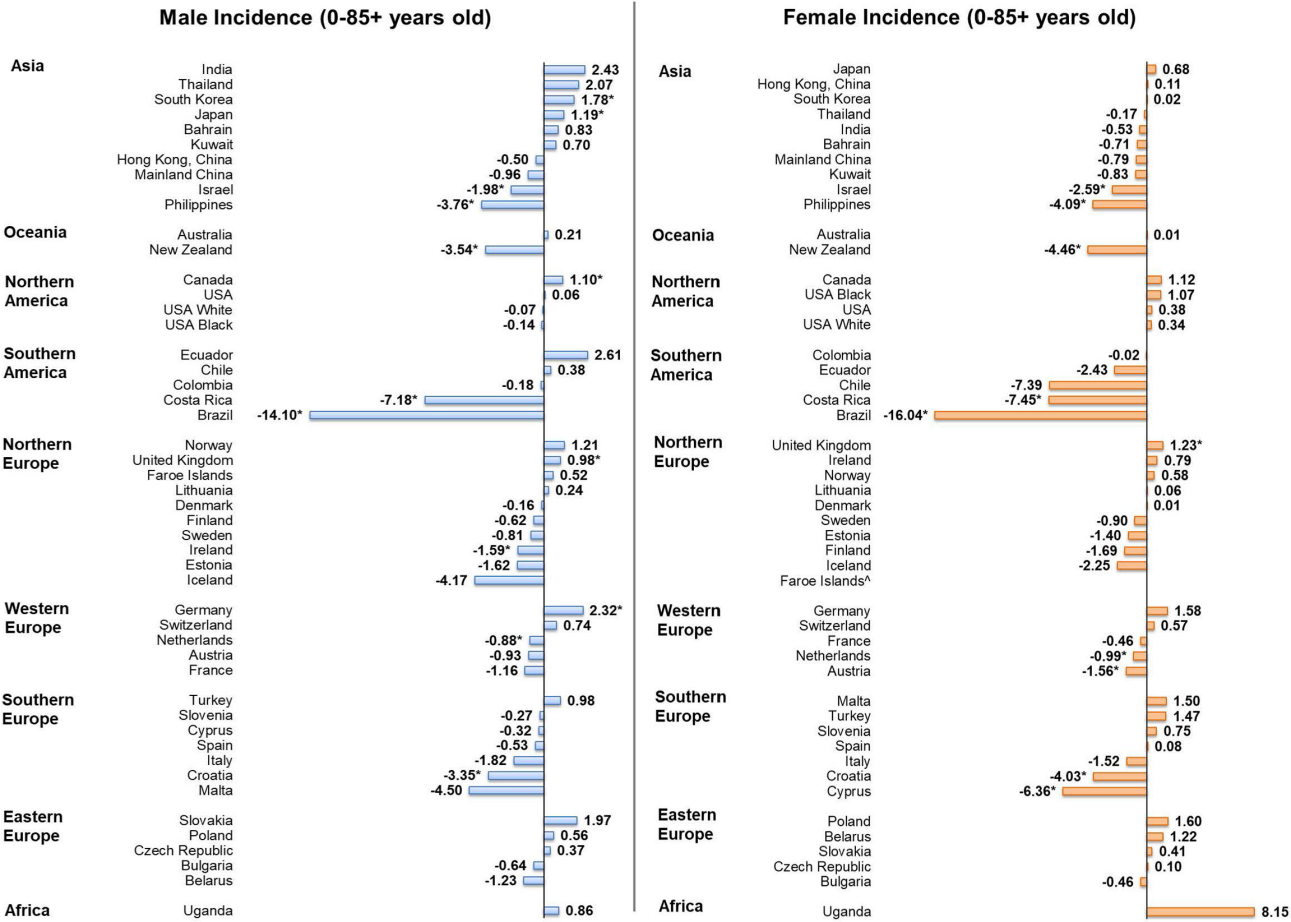
AAPC of incidence of leukaemia in individuals aged 0 – 85+ years old. * p values < 0.05.

Among men, five countries showed increasing trends in incidence (**Figure 4**), including, in descending order, Germany (AAPC = 2.32, 95% CI [0.50, 4.17], p value = 0.019), Korea (AAPC = 1.78, 95% CI [0.66, 2.91], p value = 0.006), Japan (AAPC = 1.19, 95% CI [0.37, 2.02], p value = 0.010), Canada (AAPC = 1.10, 95% CI [0.21, 2.00], p value = 0.010) and the United Kingdom (AAPC = 0.98, 95% CI [0.26 to 1.70], p value = 0.014). By contrast, seven countries had decreasing trends, with Brazil (AAPC = -14.10, 95% CI [-18.72, -9.21], p value < 0.001), Costa Rica (AAPC = -7.18, 95% CI [-11.13, -3.06], p value = 0.004), and the Philippines (AAPC = -3.76, 95% CI [-5.34, -2.15], p value = 0.001) reported the most drastic decreases. Among women, only the United Kingdom (AAPC = 1.23, 95% CI [0.26 to 2.20], p value = 0.019) reported an increasing trend in incidence. Conversely, nine countries reported decreasing trend, as Brazil (AAPC = -16.04, 95% CI [-25.94, -4.82], p value = 0.012), Costa Rica (AAPC = -7.45, 95% CI [-11.67,-3.03], p value = 0.005), and Cyprus (AAPC = -6.36, 95% CI [-9.97, -2.61], p = 0.005) showed the most significant decrease.

### Mortality Trends of Individuals Aged 0-85+

**Figure 5:**
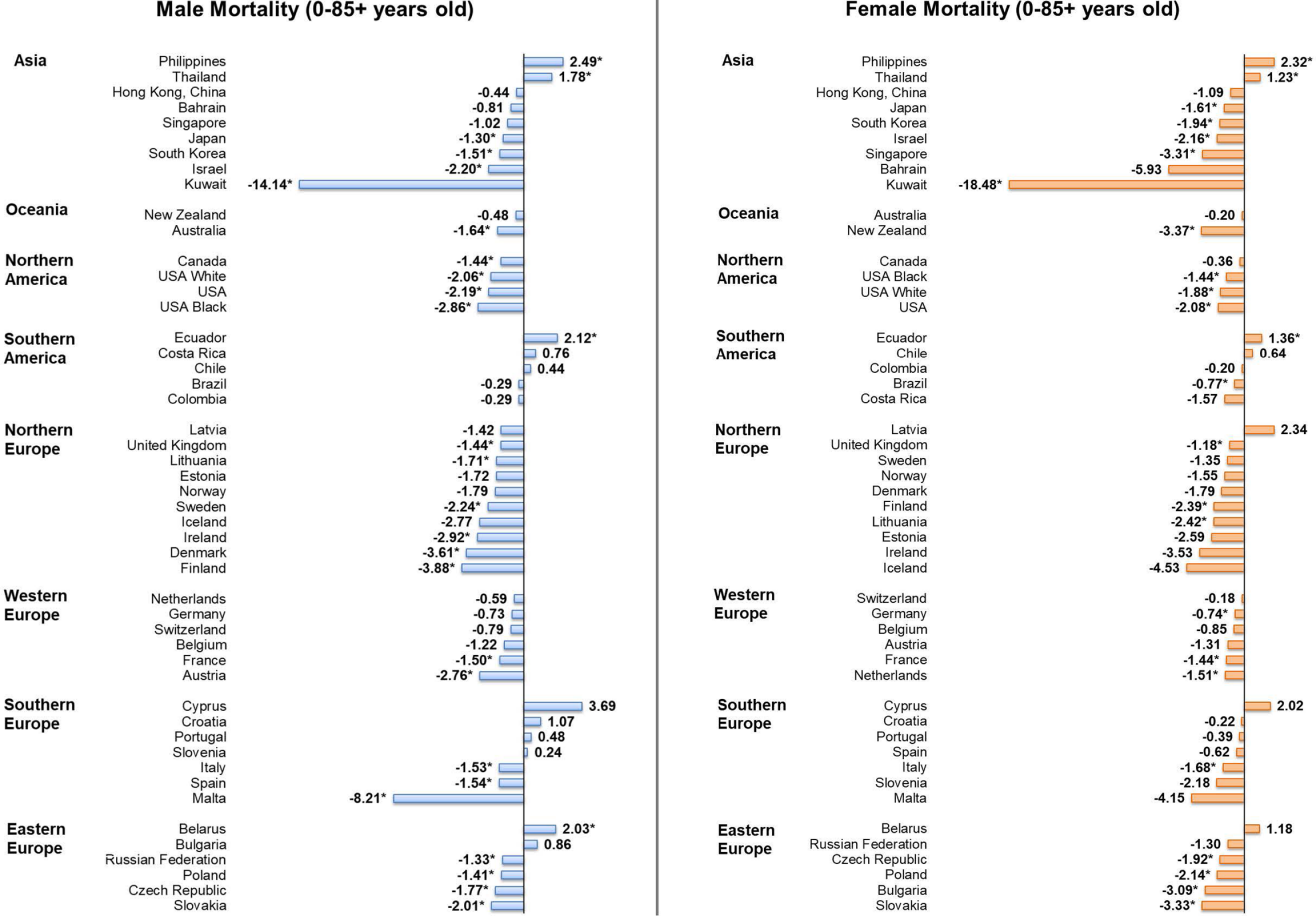
AAPC of mortality of leukaemia in individuals aged 0 – 85+ years old. * p values < 0.05.

Considering male patients, 22 countries, including 15 European countries, showed significant decreasing trends in mortality (**Figure 5**). Countries with the most significant decrease were Kuwait (AAPC = -14.14, 95% CI [-24.51, -2.36], p = 0.026), Malta (AAPC = -8.21, 95% CI [-14.30 to -1.68], p value = 0.021) and Finland (AAPC = -3.88, 95% CI [-5.80, -1.93], p value = 0.002). In contrast, four countries reported significant increasing trends, which included the Philippines (AAPC = 2.49, 95% CI [1.03, 3.97], p value = 0.004), Ecuador (AAPC = 2.12, 95% CI [0.55, 3.72], p value = 0.014), Belarus (AAPC = 2.03, 95% CI [0.58, 3.50], p value = 0.012), and Thailand (AAPC = 1.78, 95% CI [1.07, 2.49], p value < 0.001). Considering female patients, 19 countries showed significant decreasing trends in mortality and only three countries showed significant increasing trends. Kuwait (AAPC = -18.48, 95% CI [-26.76, -9.26], p = 0.002), New Zealand (AAPC = -3.37, 95% CI [-6.31, -0.35], p = 0.033) and Slovakia (AAPC = -3.33, 95% CI [-5.20, -1.43], p value = 0.004) were the countries showing the most drastic decrease, while the increasing trends were reported in the Philippines (AAPC = 2.32, 95% CI [0.94, 3.72], p value = 0.005), Ecuador (AAPC = 1.36, 95% CI [0.04, 2.69], p value = 0.045), and Thailand (AAPC = 1.23, 95% CI [0.45, 2.02], p value = 0.007).

### Incidence Trends of Individuals in Specific Age Groups

For men aged 50 or above, five countries showed significant increases in incidence and eight countries showed significant decreases, with India (AAPC = 6.59, 95% CI [2.84, 10.49], p value = 0.003) showing the largest increase and Brazil (AAPC = -16.04, 95% CI [-23.35, -8.04], p value < 0.001) showing the largest decrease (**Supplementary Figure 3**). For the younger men aged between 15-49, four countries reported significant decreases and no countries reported significant increases, Brazil (AAPC = -17.70; 95% CI [-26.62, -7.71]; p value = 0.004) showed the largest decrease (**Supplementary Figure 4**). For the youngest age group of boys aged 14 or below, New Zealand (AAPC = 7.38; 95% CI [4.54, 10.29]; p value < 0.001) and Korea (AAPC = 3.11; 95% CI [0.89, 5.39]; p value = 0.012) reported significant increasing trends while decreasing trends were found in Brazil (AAPC = -8.96; 95% CI [-15.7, -1.68]; p value = 0.023) and Philippines (AAPC = -4.52; 95% CI [-7.78, -1.15]; p value = 0.015) (**Supplementary Figure 5**). For women aged 50 or above, only the United Kingdom (AAPC = 1.97, 95% CI [1.09, 2.86], p value = 0.001) reported a significant increase in incidence and 11 countries reported significant decreases, with Costa Rica showing the most significant decrease (AAPC = -15.50, 95% CI [-22.03, -8.42], p value = 0.001); for the younger women aged between 15-49, four countries and two countries reported significant increases and decreases respectively, in which Uganda (AAPC = 9.12; 95% CI [1.12, 17.75]; p value = 0.03) had the largest increase and Brazil (AAPC = -18.76; 95% CI [-33.92, -0.12]; p value = 0.049) had the largest decrease. For girls aged 14 or below, only Belarus (AAPC = 5.71; 95% CI [2.02, 9.54]; p value = 0.007) showed a significant increasing trend whereas decreasing trends were observed in Bahrain (AAPC = -11.87; 95% CI [-22.16, -0.23]; p value = 0.047) and three other countries.

## Discussion

### Summary of Major Findings

This analysis provides the most updated evaluation of the global burden, risk factors, and epidemiologic trends of leukaemia by age, sex, and country using data from cancer registries. We have several major findings: 1) the highest incidence and mortality rates of leukaemia were observed in countries with higher income and among males; 2) higher incidence and mortality of leukaemia were associated with a higher HDI, GDP per capita, prevalence of smoking, inactivity, overweight, obesity, and hypercholesterolaemia; 3) there was an overall decreasing trend of leukaemia for the recent past decade, while an increasing incidence and mortality were observed in some populations, including men and younger individuals.

### Explanation of Findings and Relationship With Literature

There was a disparity in the distribution of leukaemia across different regions in 2020. The study found the highest incidence of leukaemia in North America, Australia and New Zealand, Western Europe, and Northern Europe. The burden of leukaemia was associated with HDI and GDP per capita at the country level. These findings are generally consistent with a previous study which concluded regions with high Socio-demographic Index (SDI) usually had higher ASRs of leukaemia (7). The reasons behind this phenomenon remain unexplored but may be related to genetics (16). Other possible factors may include a higher prevalence of environmental and lifestyle risk factors, metabolic diseases, and level of technology and capacity of detection for leukaemia in more developed regions (19 20). Also, low- and middle- income countries (LMICs) often have a lower life expectancy, the relatively lower mortality may be attributable to the occurrence of competing causes of deaths. The incidence and mortality of leukaemia were higher in men than in women, which was likely due to a higher level of exposure to leukaemia-related risk factors among men, including lifestyle risk factors (e.g., smoking) and occupational factors (e.g., ionizing and non-ionizing radiation, hydrocarbons and pesticides) (17–21).

This study found some preventable and common lifestyle and metabolic risk factors associated with the incidence and mortality of leukaemia at a country level, including smoking, physical inactivity, overweight, obesity, and hypercholesterolaemia. The results are generally supported by the findings of previous individual-level observational research on the association between these risk factors and risk of leukaemia. For instance, a meta-analysis of 23 studies showed that current and ever smokers have 40% (RR 1.40, 95% CI 1.22–1.60) and 25% (RR 1.25, 95% CI 1.15–1.36) increased risk of developing AML when compared with non-smokers (22). A large study of 1.44 million participants concluded high vs low levels of leisure-time physical activity were associated with lower risks of myeloid leukaemia (HR, 0.80; 95% CI, 0.70-0.92) (23). Another meta-analysis found the RRs of leukaemia were 1.14 [95% confidence interval (CI), 1.03–1.25] for overweight participants and 1.39 (95% CI, 1.25–1.54) for obese participants (24). Evidence also suggested there is a high incidence of hypercholesterolemia in CLL patients (25).

For the recent past decade, there was an overall decreasing trend of leukaemia incidence and mortality. The attributable factors to this favourable trend may include: 1) progress in therapies for leukaemia and their associated treatment‐related prognosis; 2) reduction in exposure to environmental risk factors and smoking; 3) decrease in childhood leukaemia; 4) increase in intake of folate and vitamin supplementation during pregnancy; and 5) expanded genetic screening for high-risk germline mutations (5, 26–31). Nevertheless, we also observed a significant increase in leukaemia incidence and mortality in some populations. The incidence increases in more developed countries may be likely due to continuous improvement in the technology and capacity of detection for leukaemia so that more leukaemia cases were diagnosed and recorded. On the contrary, the mortality increases in less developed countries are more concerning since this unfavourable trend may be driven by the increasing prevalence of risk factors for leukaemia in these regions. More intensive risk modifications are therefore required for these countries.

### Limitations

There are some limitations to the current study. Firstly, there could be under-reporting of the incidence and mortality of leukaemia in the developing countries due to the underdevelopment of infrastructure and mechanism of cancer reporting in these regions. Secondly, numbers might have been overestimated for some countries since their figures were represented by cancer registries of the major cities. Thirdly, a direct comparison between different countries could be difficult as the cancer registration might have changed over time. However, this limitation is of less concern when we compared the incidence and mortality of leukaemia according to age and sex groups within the same region. Furthermore, there was a lack of analysis on the trend of the different subtypes of leukaemia. As the geographical distribution, risk factors, and epidemic trends could vary by different subtypes of leukaemia, this information bare important implications for diseases prevention. Lastly, linking exposure at a country-level to individuals and controlling for confounders may be difficult based on the ecological epidemiological design of the study. Possible confounders may include the prolonged life expectancy in countries with higher HDI, while the association between BMI and hypercholesterolaemia and the burden of leukaemia might be confounded by the more accurate diagnostic procedures in countries with a higher HDI. Therefore, the findings between the exposure and the trends should be interpreted with caution.

### Conclusions

The incidence and mortality of leukaemia has been decreasing for the past decade likely due to the recent development of novel therapeutic strategies and targeted drugs for leukaemia. However, an increasing trend of leukaemia incidence was found in Germany, Korea, Japan, Canada and the United Kingdom while mortality increased in Ecuador, Belarus, Thailand, and the Philippines. Intensive lifestyle modifications including further smoking reduction, physical activity, weight control, and optimal management of hypercholesterolaemia might be beneficial to reduce the risk of leukaemia, especially among men and younger individuals. It is also important to improve early detection, treatment, surveillance, and quality of life for patients with leukaemia. Lastly, further longitudinal research is required to explore the reasons behind these epidemiologic trends observed and give more insights into the specific aetiology and prognosis of leukaemia by different subtypes. Study should be done to confirm the association between the lifestyle factors and the risk of leukaemia at an individual level.

## Data Availability Statement

The original contributions presented in the study are included in the article/**Supplementary Material**. Further inquiries can be directed to the corresponding authors.

## Ethics Statement

This study was approved by the Survey and Behavioural Research Ethics Committee, the Chinese University of Hong Kong (No. SBRE-20-332).

## Author Contributions

JH and MCSW participated in the conception of the research ideas, study design, interpretation of the findings, writing of the first draft of the manuscript, and provided intellectual input to the translational aspects of the study. SC, CN, and VL retrieved information from the relevant databases, performed the statistical analysis, and presented the methodology and results. MW, LZ, DL-P, WX, Z-JZ, and EE made critical revisions on the manuscripts and provided expert opinions on implications of the study findings. All authors contributed to the article and approved the submitted version.

## Conflict of Interest

The authors declare that the research was conducted in the absence of any commercial or financial relationships that could be construed as a potential conflict of interest.

## Publisher’s Note

All claims expressed in this article are solely those of the authors and do not necessarily represent those of their affiliated organizations, or those of the publisher, the editors and the reviewers. Any product that may be evaluated in this article, or claim that may be made by its manufacturer, is not guaranteed or endorsed by the publisher.
