# Supplementary material for: Disease Burden, Risk Factors, and Trends of Leukaemia: A Global Analysis
Source: Front Oncol. 2022 Jul 22;12:904292. doi: 10.3389/fonc.2022.904292 (PMC9355717; doi:10.3389/fonc.2022.904292)
Supplement: Supplementary file 1 [file DataSheet_1.pdf]

Supplementary Legends

Supplementary Figure 1. Incidence and mortality trends for individual countries

Supplementary Figure 2. Results of joinpoint regression for individual countries

Supplementary Figure 3. AAPC of incidence of leukaemia aged 50 years and older

Supplementary Figure 4. AAPC of incidence of leukaemia aged between 15-49 years old

Supplementary Figure 5. AAPC of incidence of leukaemia aged < 15 years old

Supplementary Figure 1: The plots of incidence and mortality trends for each country

Male

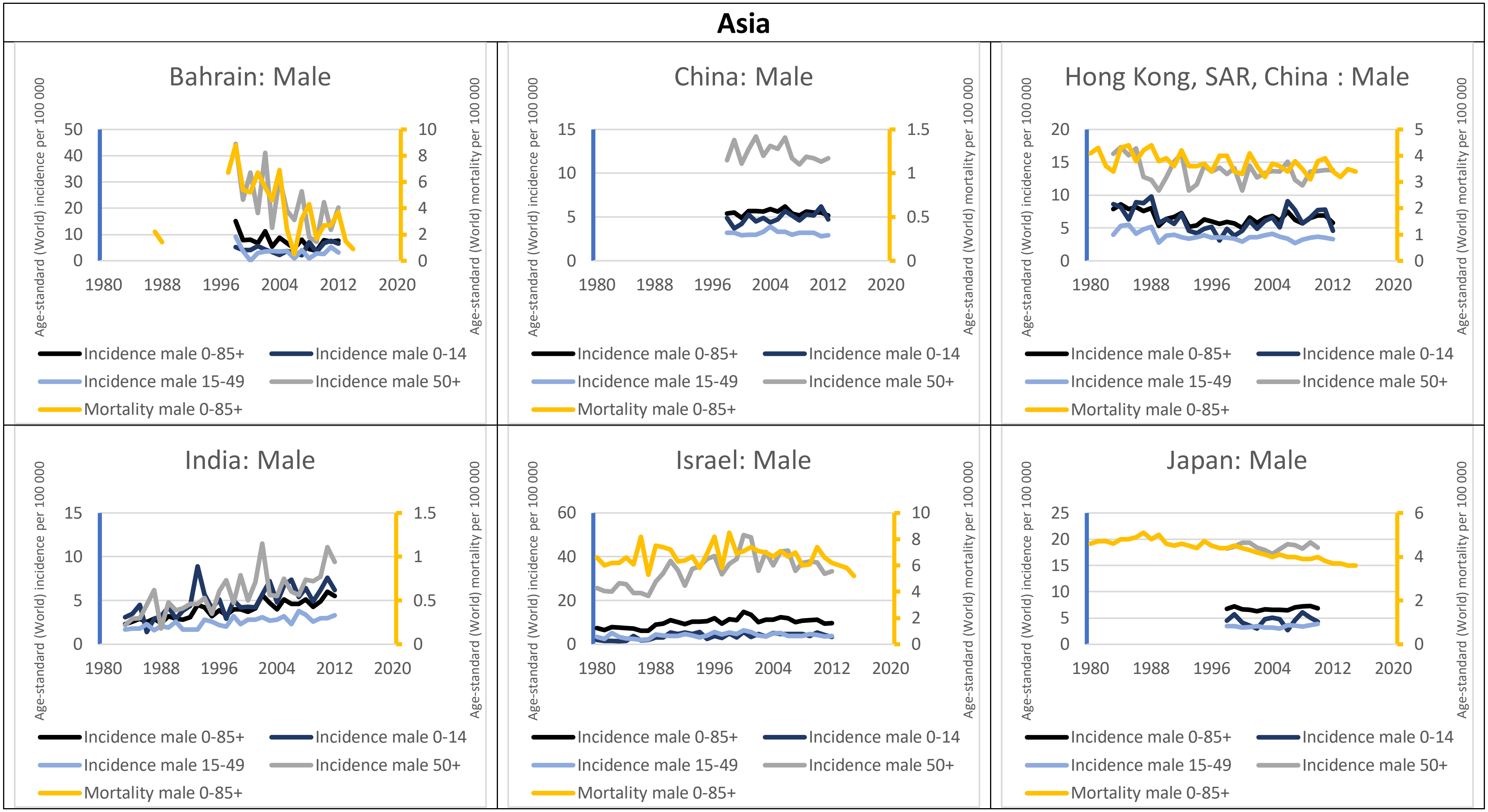

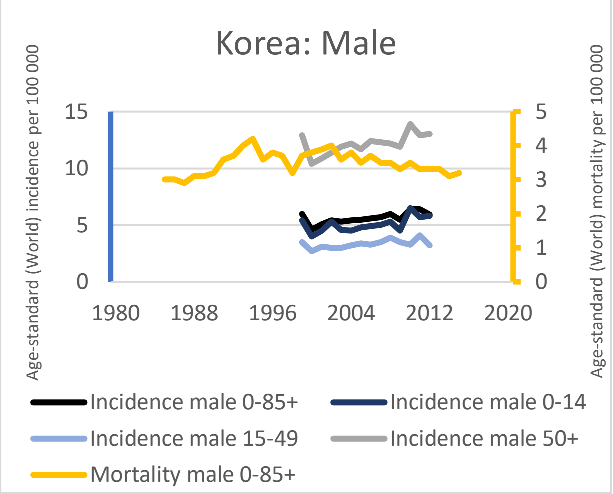
Korea: Male
Age-standard (World) incidence per 100 000
Age-standard (World) mortality per 100 000
15
10
5
0
5
4
3
2
1
0
1980
1988
1996
2004
2012
2020
Incidence male 0-85+
Incidence male 0-14
Incidence male 15-49
Incidence male 50+
Mortality male 0-85+

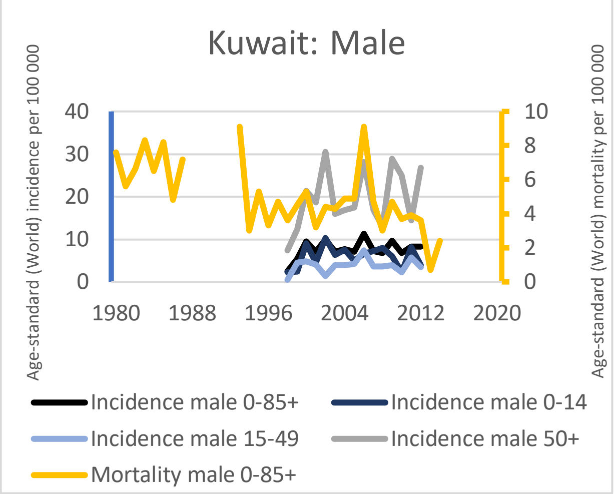
Kuwait: Male
Age-standard (World) incidence per 100 000
Age-standard (World) mortality per 100 000
40
30
20
10
0
10
8
6
4
2
0
1980
1988
1996
2004
2012
2020
Incidence male 0-85+
Incidence male 0-14
Incidence male 15-49
Incidence male 50+
Mortality male 0-85+

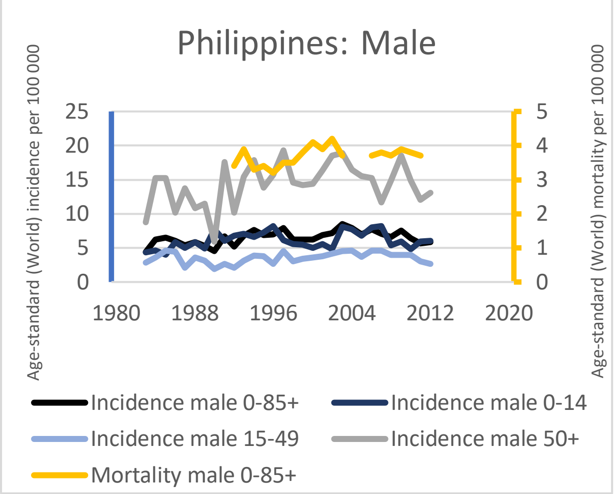
Philippines: Male
Age-standard (World) incidence per 100 000
Age-standard (World) mortality per 100 000
25
20
15
10
5
0
5
4
3
2
1
0
1980
1988
1996
2004
2012
2020
Incidence male 0-85+
Incidence male 0-14
Incidence male 15-49
Incidence male 50+
Mortality male 0-85+

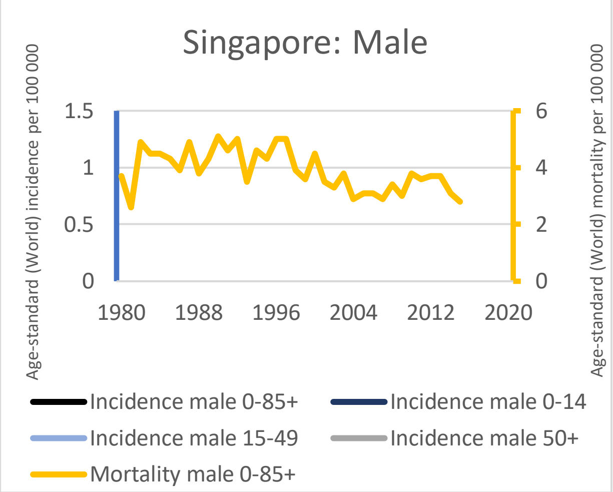
Singapore: Male
Age-standard (World) incidence per 100 000
Age-standard (World) mortality per 100 000
1.5
1
0.5
0
6
4
2
0
1980
1988
1996
2004
2012
2020
Incidence male 0-85+
Incidence male 0-14
Incidence male 15-49
Incidence male 50+
Mortality male 0-85+

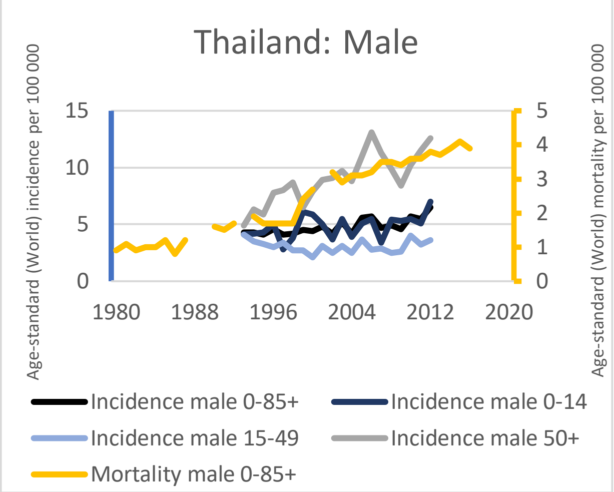
Thailand: Male
Age-standard (World) incidence per 100 000
Age-standard (World) mortality per 100 000
15
10
5
0
5
4
3
2
1
0
1980
1988
1996
2004
2012
2020
Incidence male 0-85+
Incidence male 0-14
Incidence male 15-49
Incidence male 50+
Mortality male 0-85+

Oceania

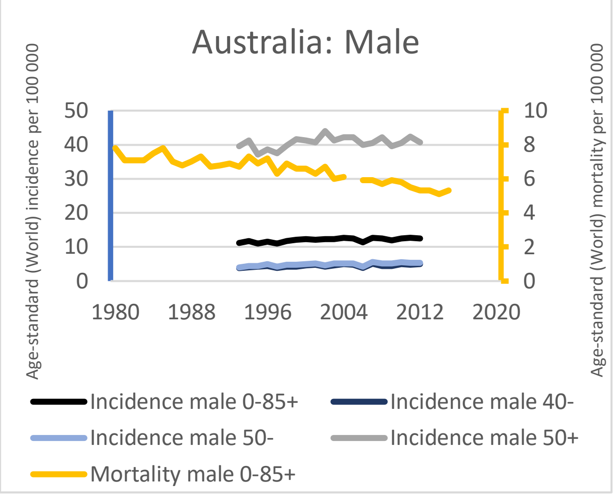

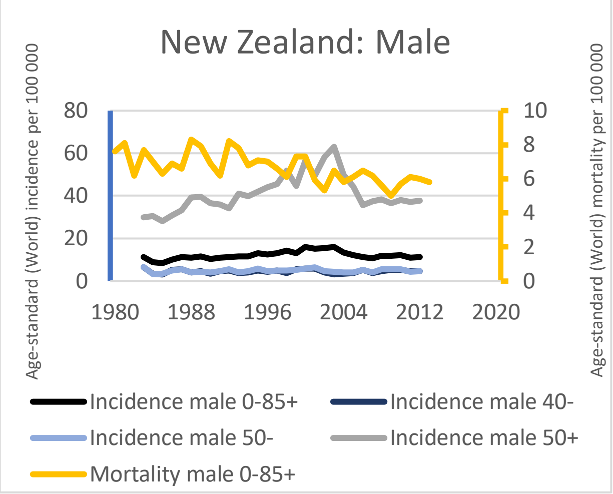

Northern America

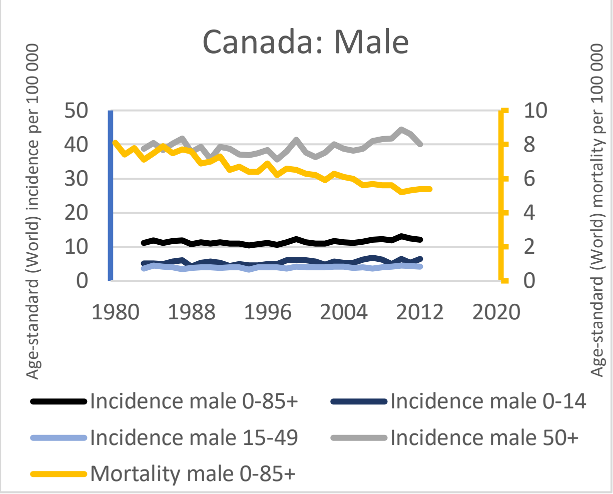

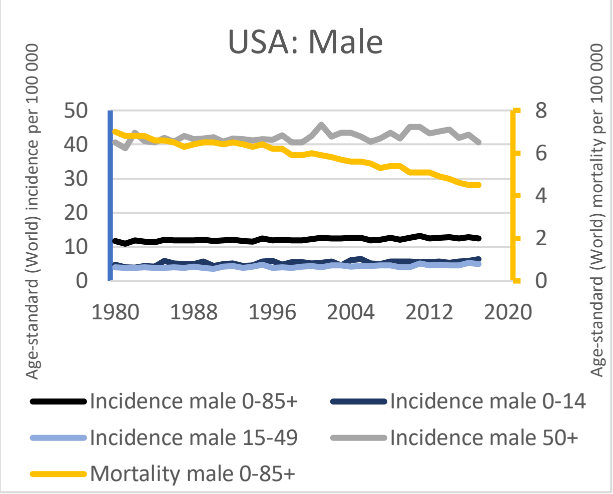

Southern America

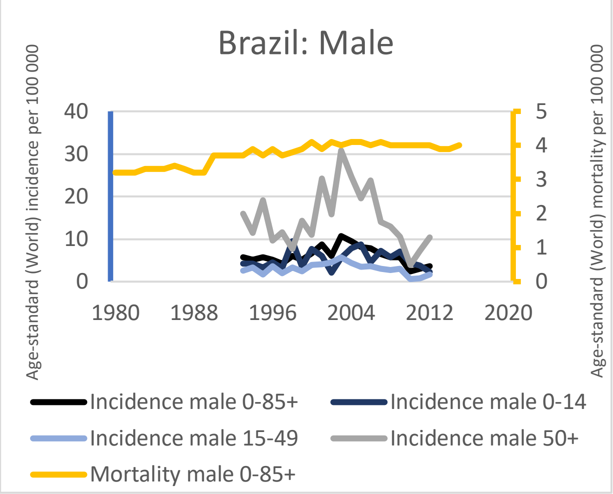

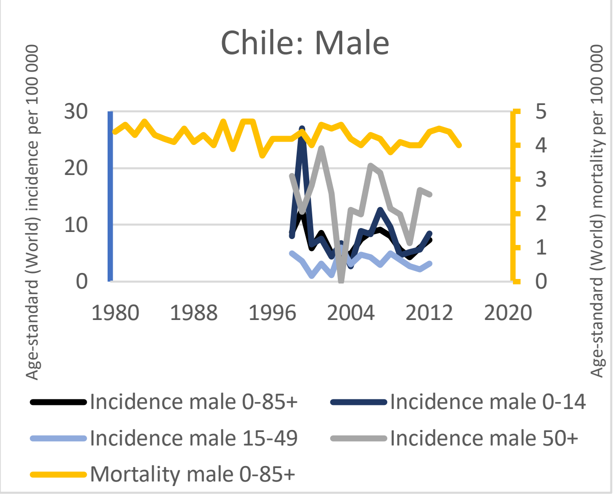

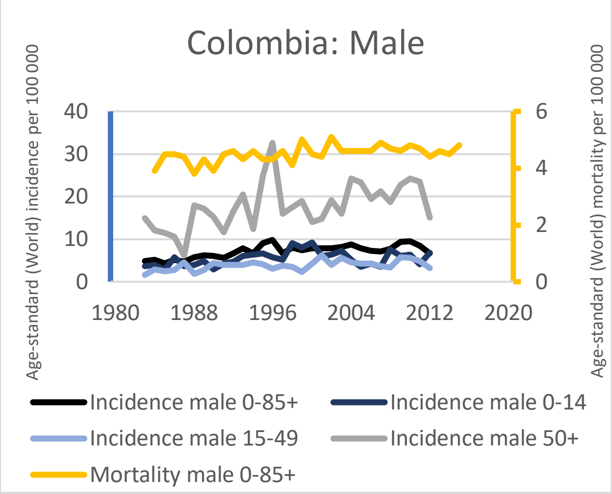

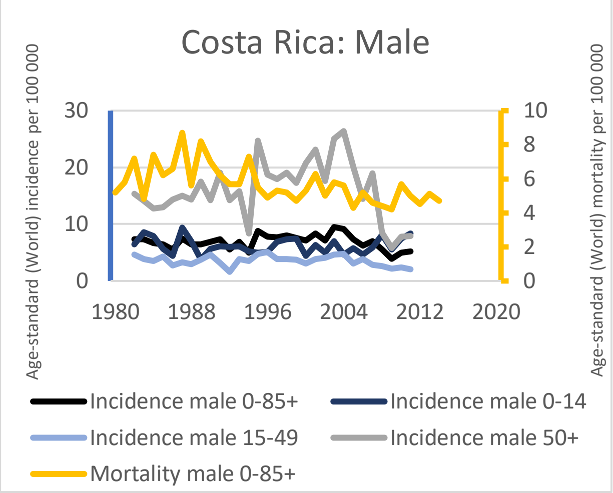

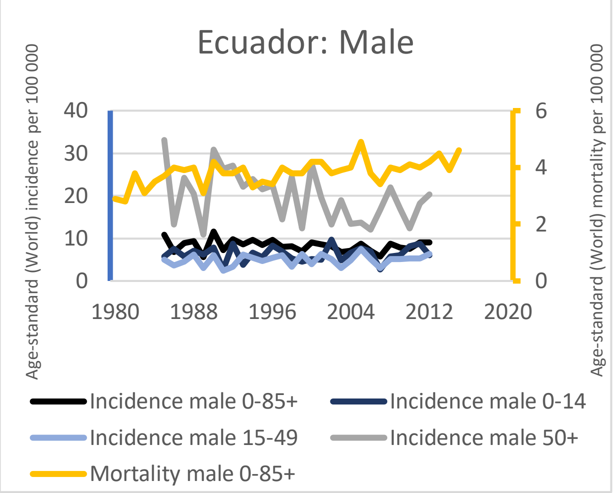

Northern Europe

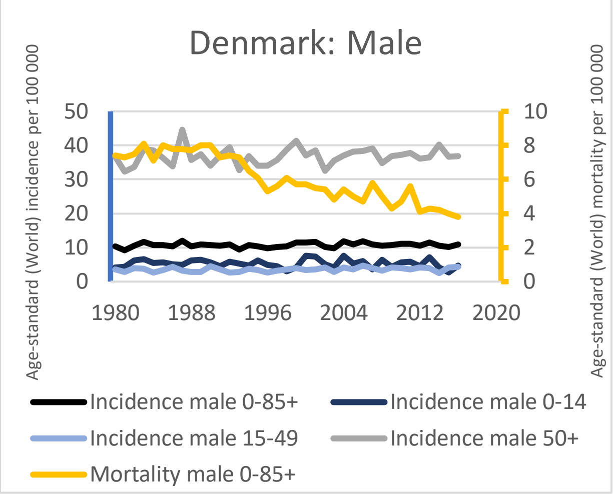
Denmark: Male
Age-standard (World) incidence per 100 000
Age-standard (World) mortality per 100 000
0 10 20 30 40 50
0 2 4 6 8 10
1980 1988 1996 2004 2012 2020
Incidence male 0-85+
Incidence male 0-14
Incidence male 15-49
Incidence male 50+
Mortality male 0-85+

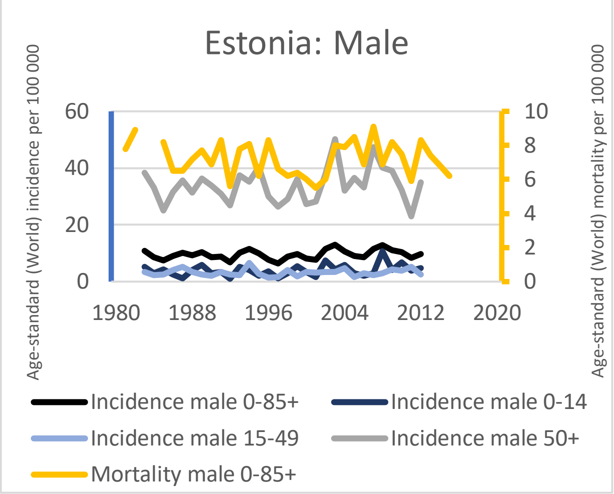
Estonia: Male
Age-standard (World) incidence per 100 000
Age-standard (World) mortality per 100 000
0 20 40 60
0 2 4 6 8 10
1980 1988 1996 2004 2012 2020
Incidence male 0-85+
Incidence male 0-14
Incidence male 15-49
Incidence male 50+
Mortality male 0-85+

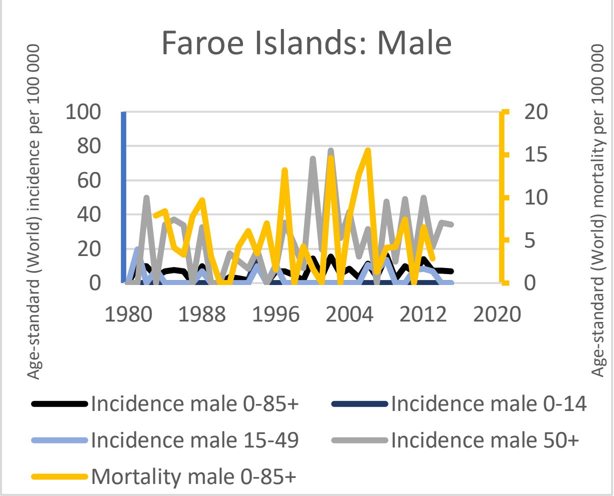
Faroe Islands: Male
Age-standard (World) incidence per 100 000
Age-standard (World) mortality per 100 000
0 20 40 60 80 100
0 5 10 15 20
1980 1988 1996 2004 2012 2020
Incidence male 0-85+
Incidence male 0-14
Incidence male 15-49
Incidence male 50+
Mortality male 0-85+

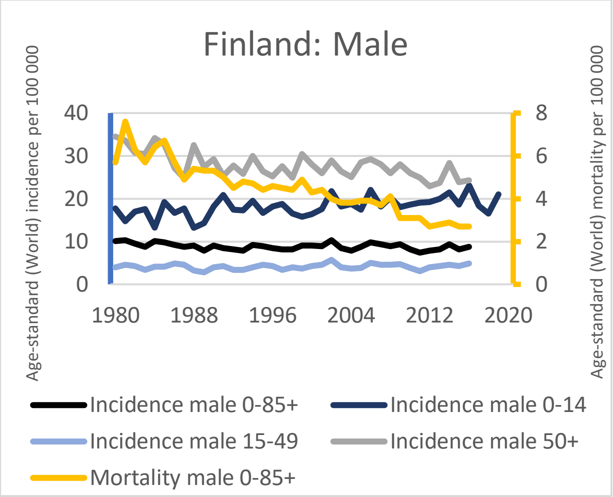
Finland: Male
Age-standard (World) incidence per 100 000
Age-standard (World) mortality per 100 000
0 10 20 30 40
0 2 4 6 8
1980 1988 1996 2004 2012 2020
Incidence male 0-85+
Incidence male 0-14
Incidence male 15-49
Incidence male 50+
Mortality male 0-85+

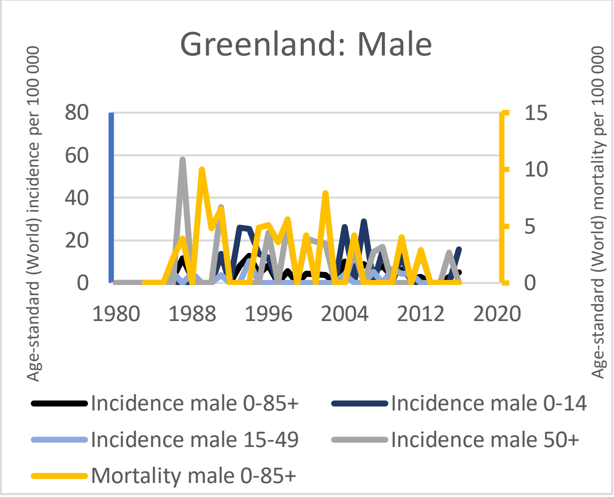
Greenland: Male
Age-standard (World) incidence per 100 000
Age-standard (World) mortality per 100 000
0 20 40 60 80
0 5 10 15
1980 1988 1996 2004 2012 2020
Incidence male 0-85+
Incidence male 0-14
Incidence male 15-49
Incidence male 50+
Mortality male 0-85+

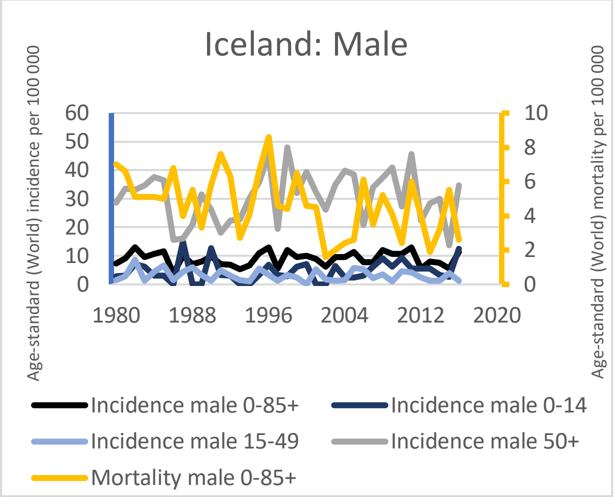
Iceland: Male
Age-standard (World) incidence per 100 000
Age-standard (World) mortality per 100 000
0 10 20 30 40 50 60
0 2 4 6 8 10
1980 1988 1996 2004 2012 2020
Incidence male 0-85+
Incidence male 0-14
Incidence male 15-49
Incidence male 50+
Mortality male 0-85+

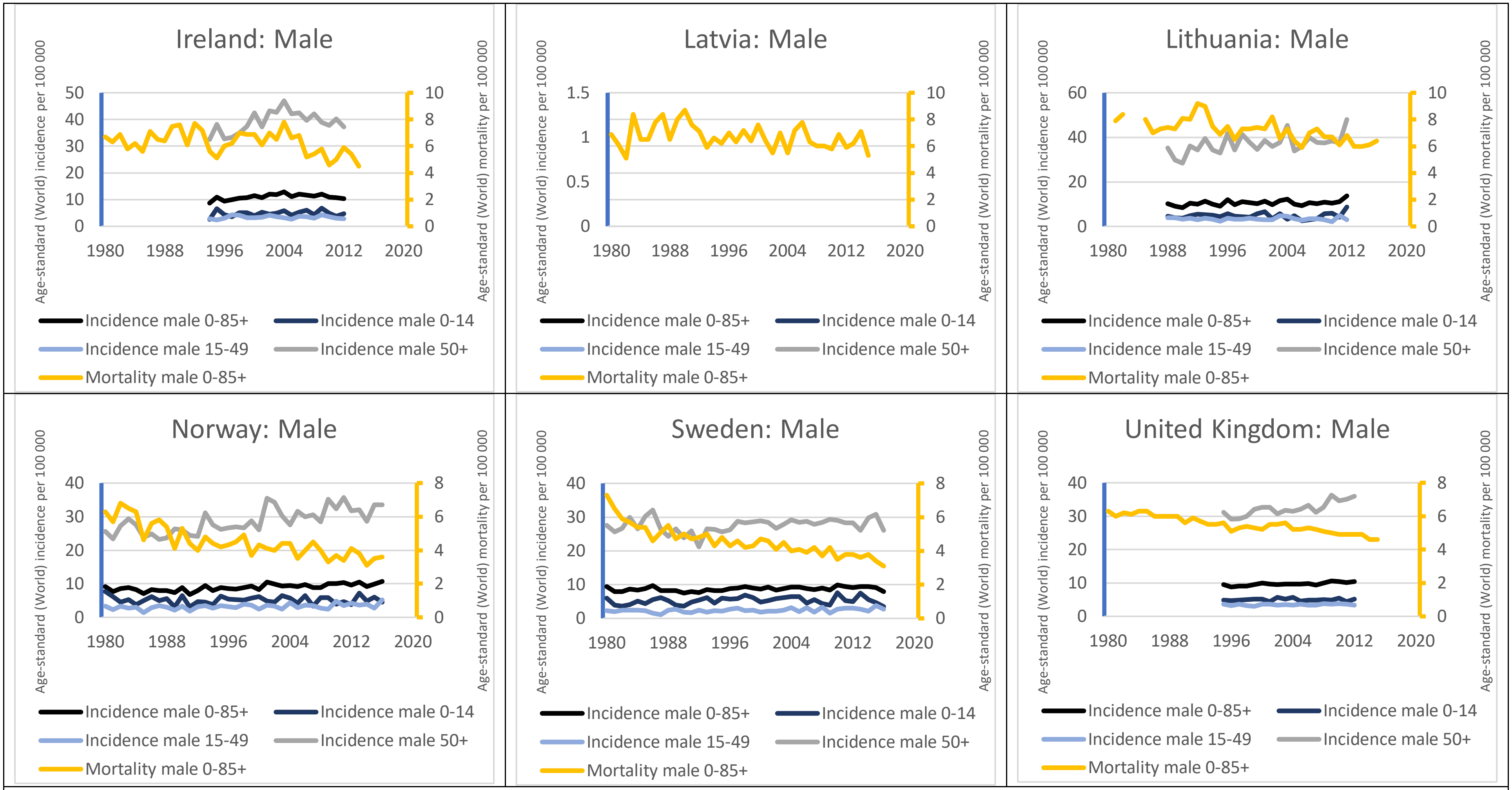

Western Europe

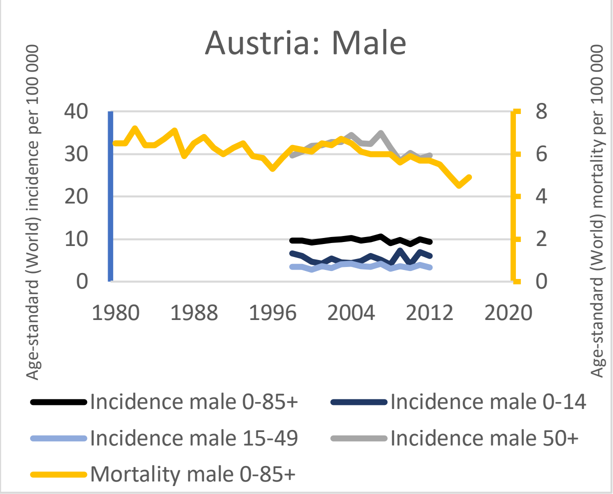

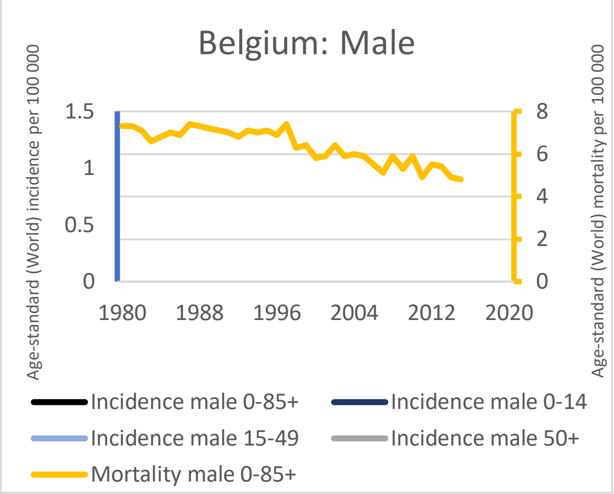

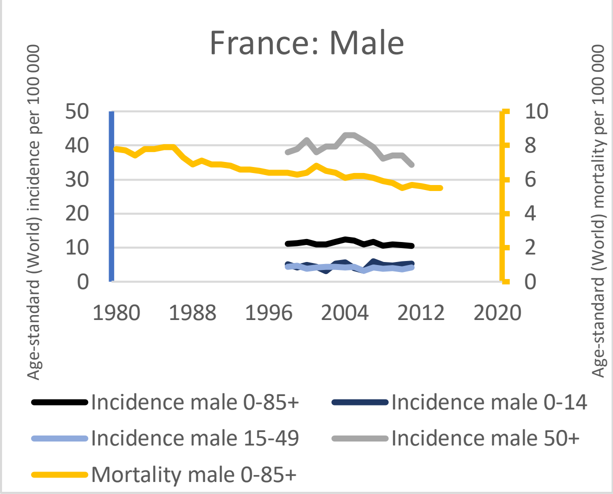

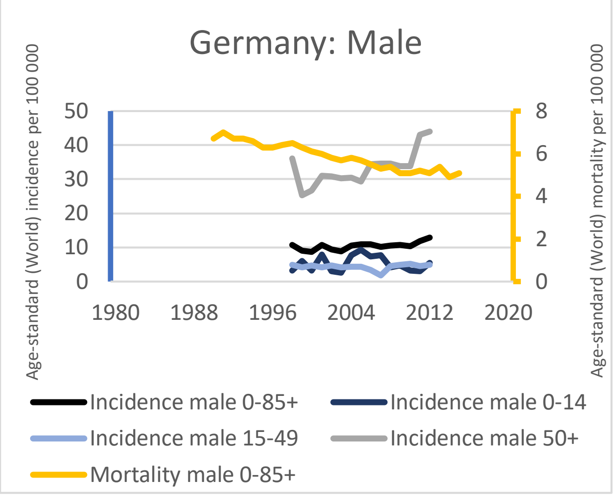

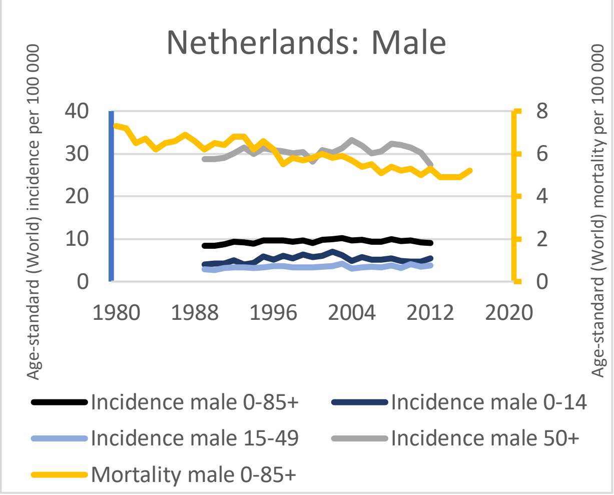

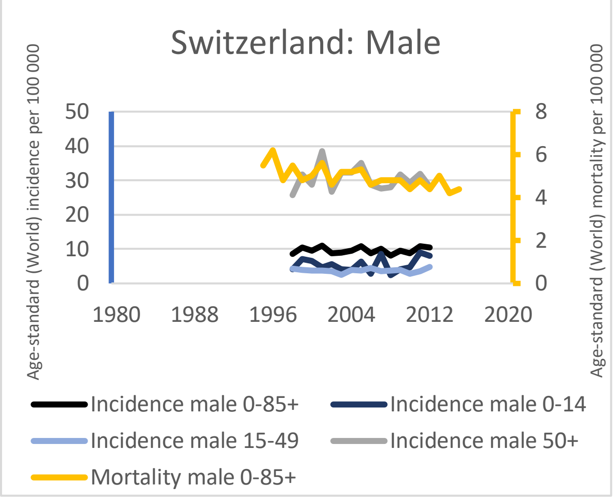

Southern Europe

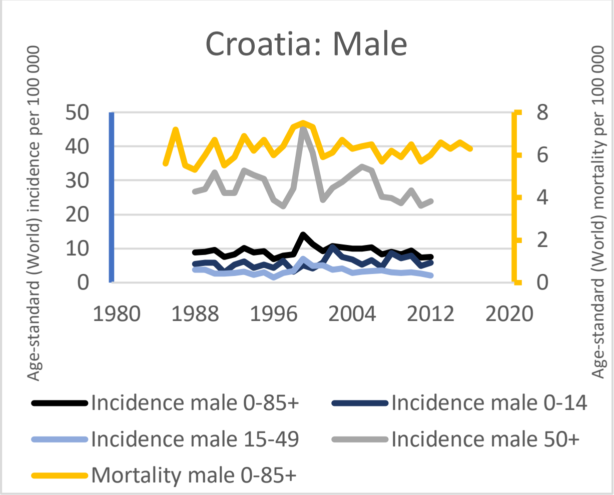
Croatia: Male
Age-standard (World) incidence per 100 000
Age-standard (World) mortality per 100 000
Incidence male 0-85+
Incidence male 0-14
Incidence male 15-49
Incidence male 50+
Mortality male 0-85+

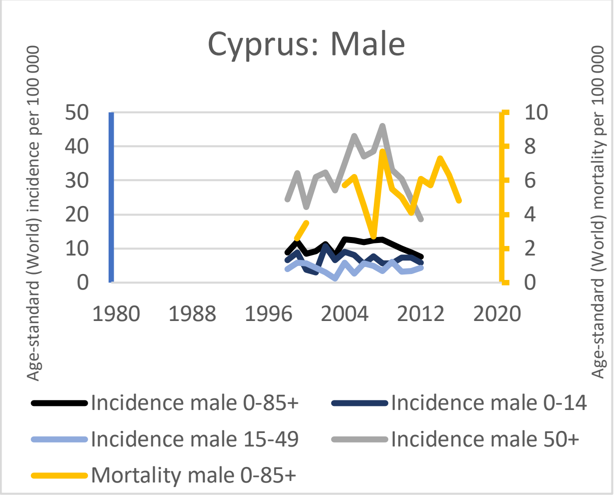
Cyprus: Male
Age-standard (World) incidence per 100 000
Age-standard (World) mortality per 100 000
Incidence male 0-85+
Incidence male 0-14
Incidence male 15-49
Incidence male 50+
Mortality male 0-85+

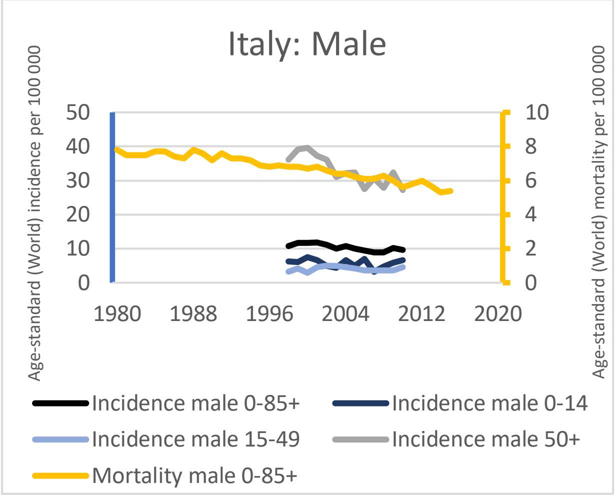
Italy: Male
Age-standard (World) incidence per 100 000
Age-standard (World) mortality per 100 000
Incidence male 0-85+
Incidence male 0-14
Incidence male 15-49
Incidence male 50+
Mortality male 0-85+

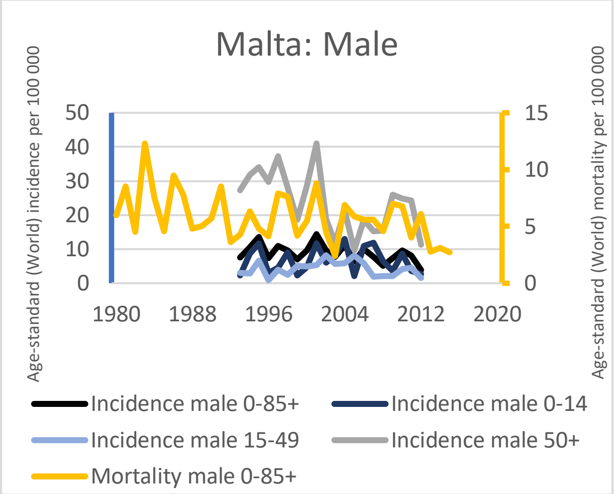
Malta: Male
Age-standard (World) incidence per 100 000
Age-standard (World) mortality per 100 000
Incidence male 0-85+
Incidence male 0-14
Incidence male 15-49
Incidence male 50+
Mortality male 0-85+

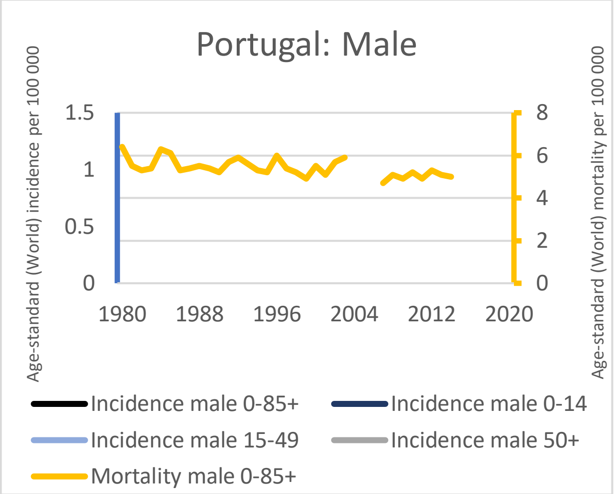
Portugal: Male
Age-standard (World) incidence per 100 000
Age-standard (World) mortality per 100 000
Incidence male 0-85+
Incidence male 0-14
Incidence male 15-49
Incidence male 50+
Mortality male 0-85+

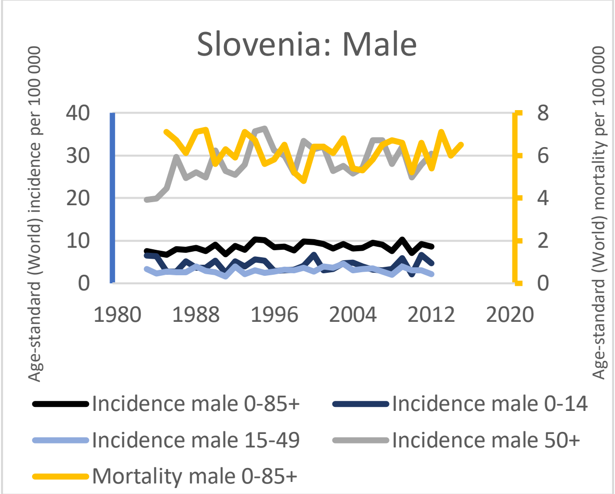
Slovenia: Male
Age-standard (World) incidence per 100 000
Age-standard (World) mortality per 100 000
Incidence male 0-85+
Incidence male 0-14
Incidence male 15-49
Incidence male 50+
Mortality male 0-85+

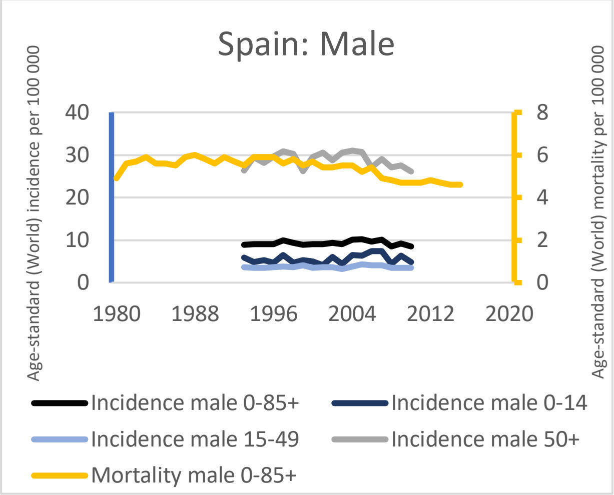
Spain: Male
Age-standard (World) incidence per 100 000
Age-standard (World) mortality per 100 000
40
30
20
10
0
8
6
4
2
0
1980
1988
1996
2004
2012
2020
Incidence male 0-85+
Incidence male 0-14
Incidence male 15-49
Incidence male 50+
Mortality male 0-85+

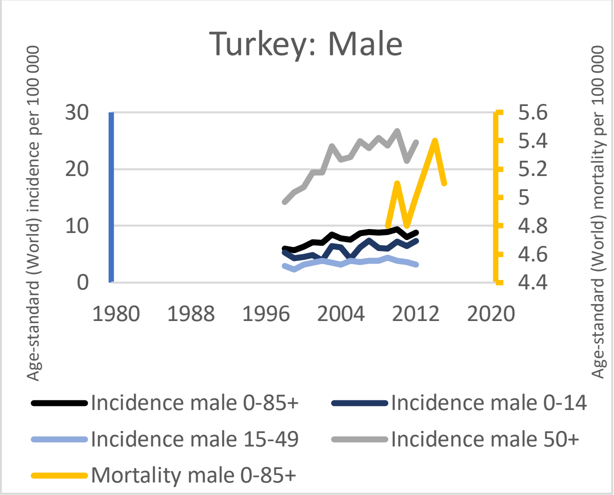
Turkey: Male
Age-standard (World) incidence per 100 000
Age-standard (World) mortality per 100 000
30
20
10
0
5.6
5.4
5.2
5
4.8
4.6
4.4
1980
1988
1996
2004
2012
2020
Incidence male 0-85+
Incidence male 0-14
Incidence male 15-49
Incidence male 50+
Mortality male 0-85+

Eastern Europe

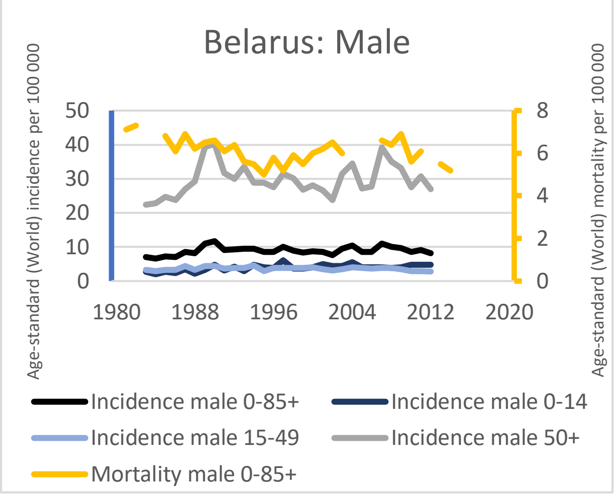

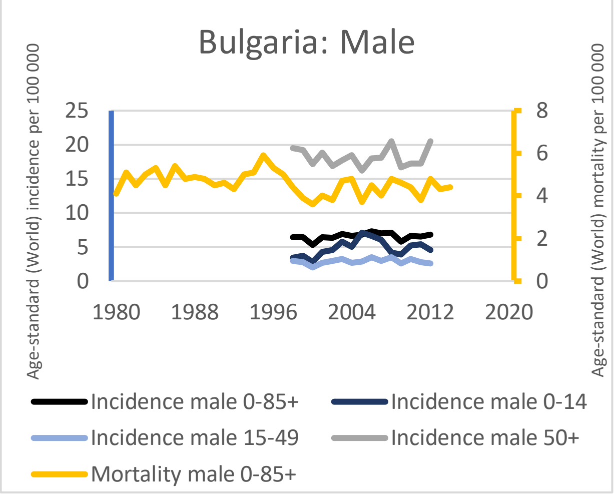

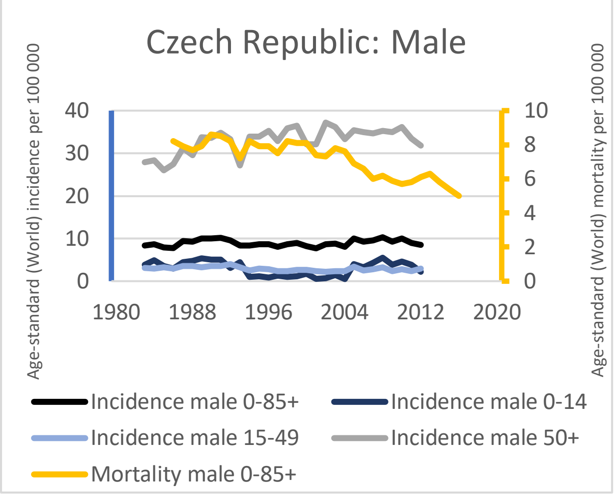

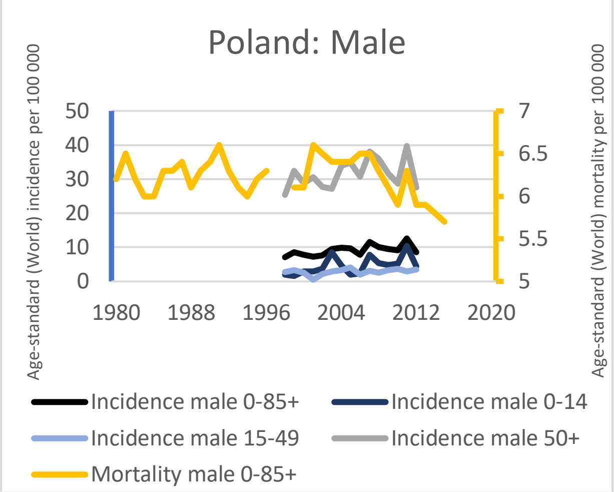

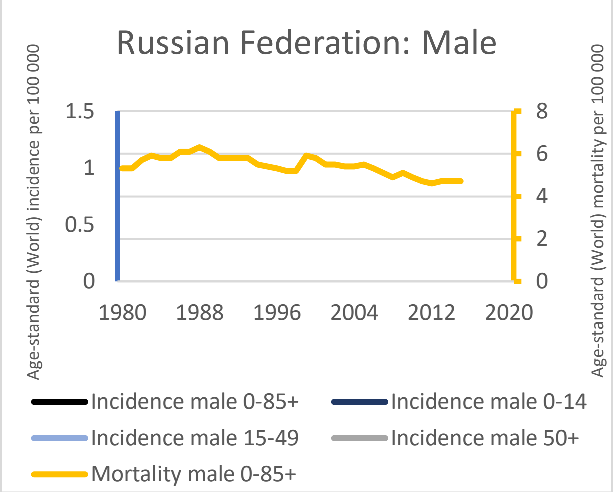

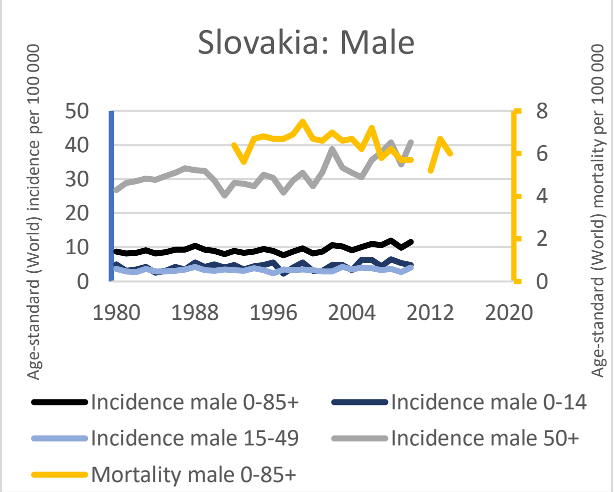

Africa		
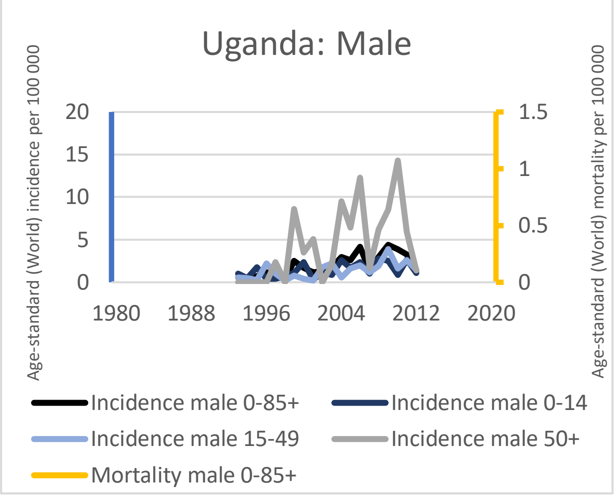 Uganda: Male<br>Age-standard (World) incidence per 100 000<br>20<br>15<br>10<br>5<br>0<br>1980<br>1988<br>1996<br>2004<br>2012<br>2020<br>1.5<br>1<br>0.5<br>0<br>Age-standard (World) mortality per 100 000<br>Incidence male 0-85+<br>Incidence male 0-14<br>Incidence male 15-49<br>Incidence male 50+<br>Mortality male 0-85+		

Female

Asia

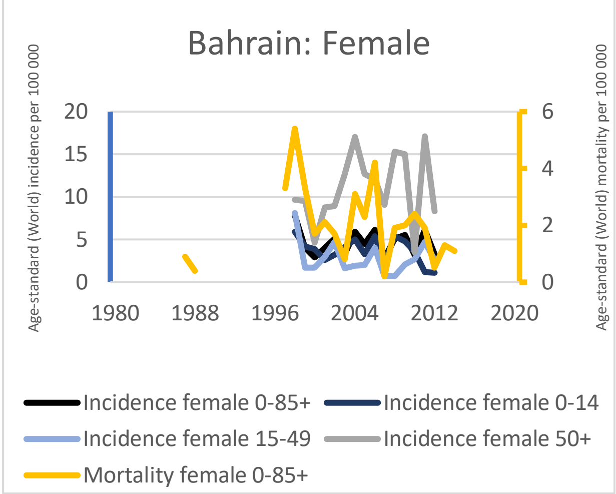
Bahrain: Female
Age-standard (World) incidence per 100 000
Age-standard (World) mortality per 100 000
0 5 10 15 20
0 2 4 6
1980 1988 1996 2004 2012 2020
Incidence female 0-85+
Incidence female 0-14
Incidence female 15-49
Incidence female 50+
Mortality female 0-85+

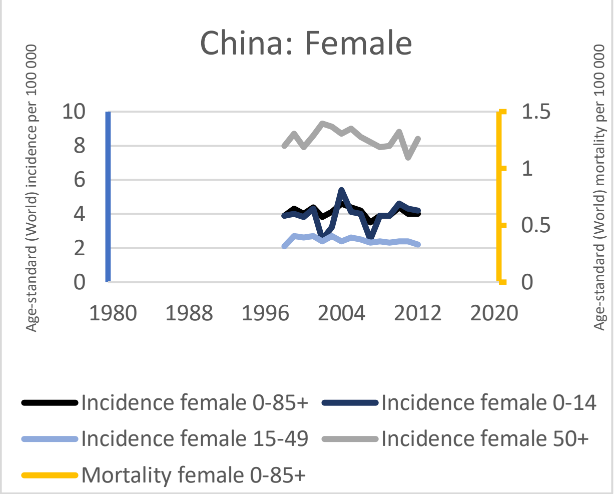
China: Female
Age-standard (World) incidence per 100 000
Age-standard (World) mortality per 100 000
0 2 4 6 8 10
0 0.5 1 1.5
1980 1988 1996 2004 2012 2020
Incidence female 0-85+
Incidence female 0-14
Incidence female 15-49
Incidence female 50+
Mortality female 0-85+

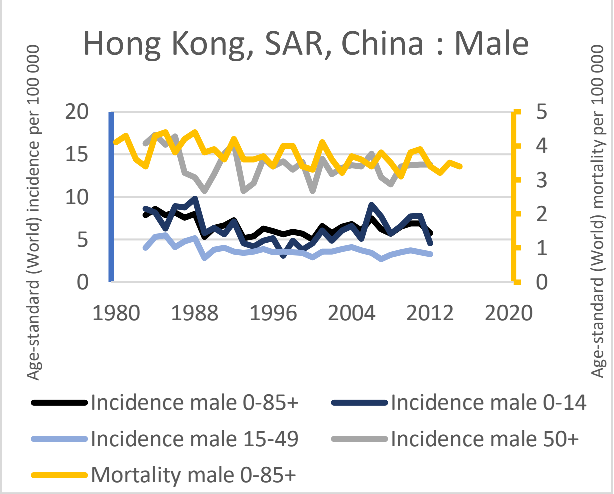
Hong Kong, SAR, China : Male
Age-standard (World) incidence per 100 000
Age-standard (World) mortality per 100 000
0 5 10 15 20
0 1 2 3 4 5
1980 1988 1996 2004 2012 2020
Incidence male 0-85+
Incidence male 0-14
Incidence male 15-49
Incidence male 50+
Mortality male 0-85+

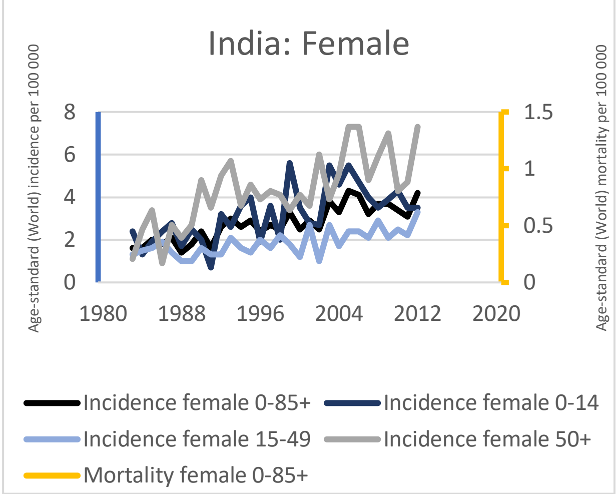
India: Female
Age-standard (World) incidence per 100 000
Age-standard (World) mortality per 100 000
0 2 4 6 8
0 0.5 1 1.5
1980 1988 1996 2004 2012 2020
Incidence female 0-85+
Incidence female 0-14
Incidence female 15-49
Incidence female 50+
Mortality female 0-85+

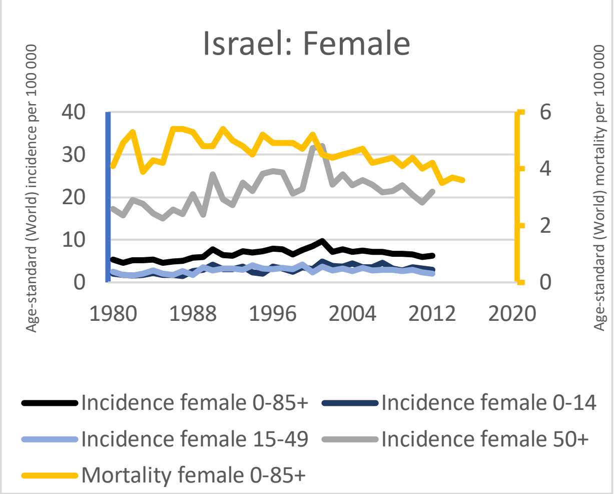
Israel: Female
Age-standard (World) incidence per 100 000
Age-standard (World) mortality per 100 000
0 10 20 30 40
0 2 4 6
1980 1988 1996 2004 2012 2020
Incidence female 0-85+
Incidence female 0-14
Incidence female 15-49
Incidence female 50+
Mortality female 0-85+

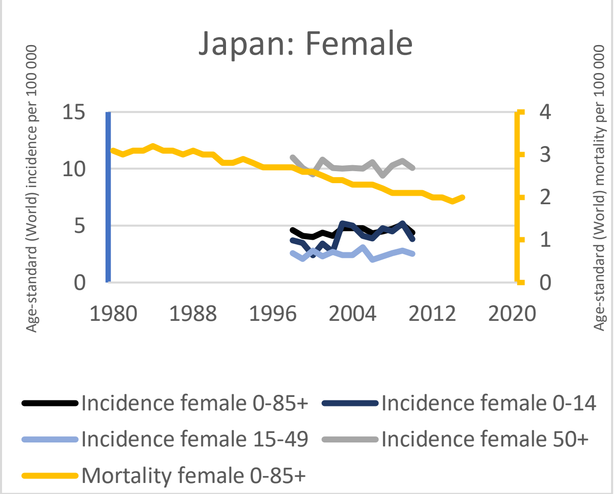
Japan: Female
Age-standard (World) incidence per 100 000
Age-standard (World) mortality per 100 000
0 5 10 15
0 1 2 3 4
1980 1988 1996 2004 2012 2020
Incidence female 0-85+
Incidence female 0-14
Incidence female 15-49
Incidence female 50+
Mortality female 0-85+

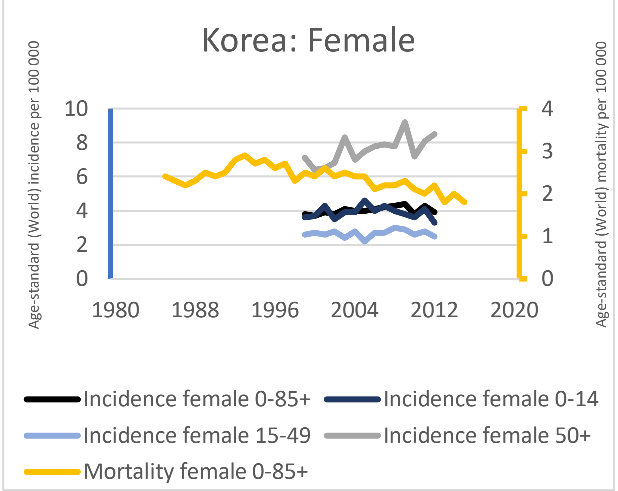
Korea: Female
Age-standard (World) incidence per 100 000
Age-standard (World) mortality per 100 000
0 2 4 6 8 10
0 1 2 3 4
1980 1988 1996 2004 2012 2020
Incidence female 0-85+
Incidence female 0-14
Incidence female 15-49
Incidence female 50+
Mortality female 0-85+

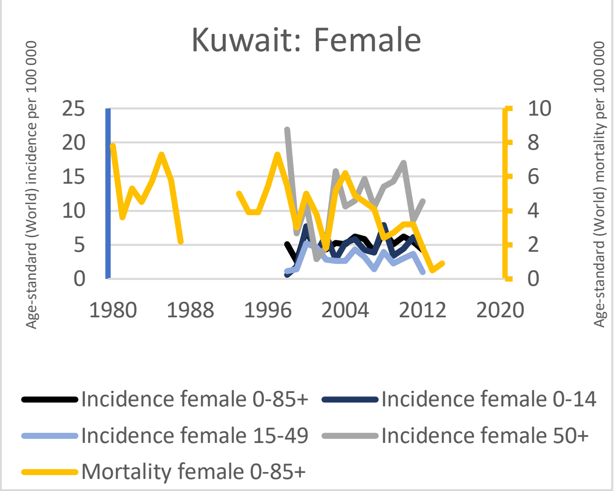
Kuwait: Female
Age-standard (World) incidence per 100 000
Age-standard (World) mortality per 100 000
0 5 10 15 20 25
0 2 4 6 8 10
1980 1988 1996 2004 2012 2020
Incidence female 0-85+
Incidence female 0-14
Incidence female 15-49
Incidence female 50+
Mortality female 0-85+

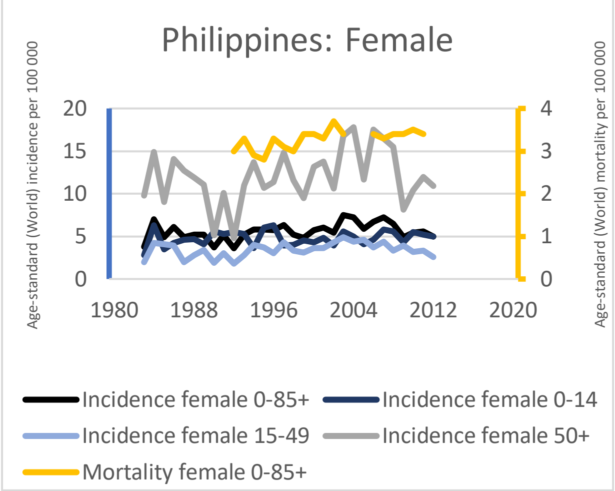
Philippines: Female
Age-standard (World) incidence per 100 000
Age-standard (World) mortality per 100 000
0 5 10 15 20
0 1 2 3 4
1980 1988 1996 2004 2012 2020
Incidence female 0-85+
Incidence female 0-14
Incidence female 15-49
Incidence female 50+
Mortality female 0-85+

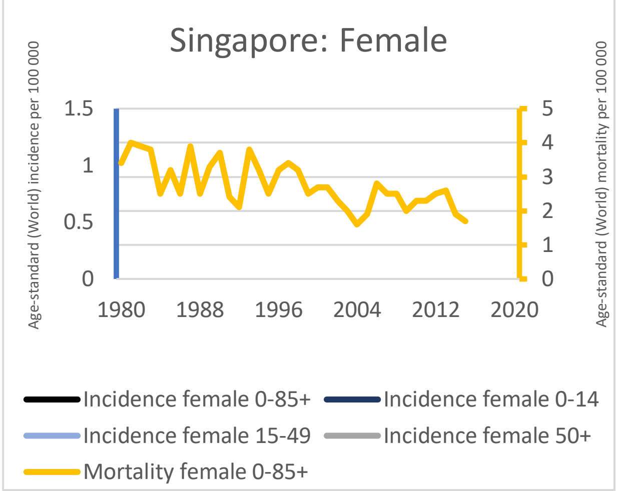
Singapore: Female
Age-standard (World) incidence per 100 000
Age-standard (World) mortality per 100 000
0 0.5 1 1.5
0 1 2 3 4 5
1980 1988 1996 2004 2012 2020
Incidence female 0-85+
Incidence female 0-14
Incidence female 15-49
Incidence female 50+
Mortality female 0-85+

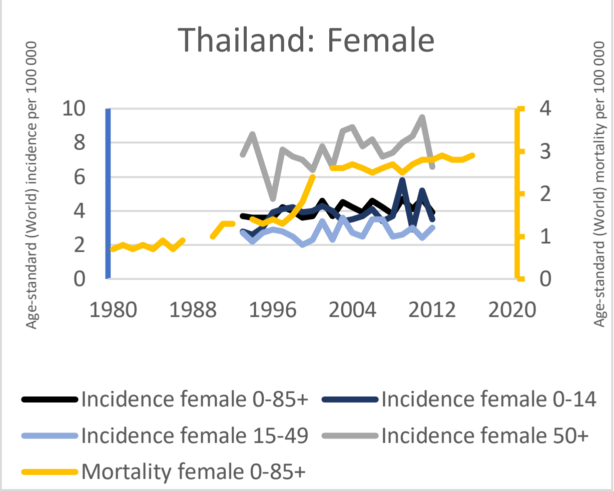
Thailand: Female
Age-standard (World) incidence per 100 000
Age-standard (World) mortality per 100 000
0 2 4 6 8 10
0 1 2 3 4
1980 1988 1996 2004 2012 2020
Incidence female 0-85+
Incidence female 0-14
Incidence female 15-49
Incidence female 50+
Mortality female 0-85+

Oceania

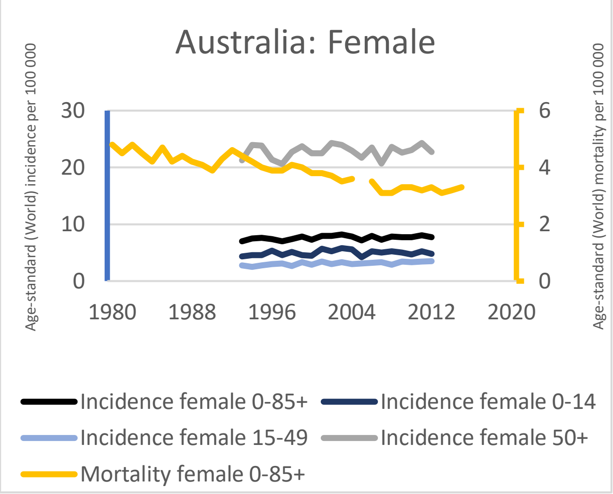

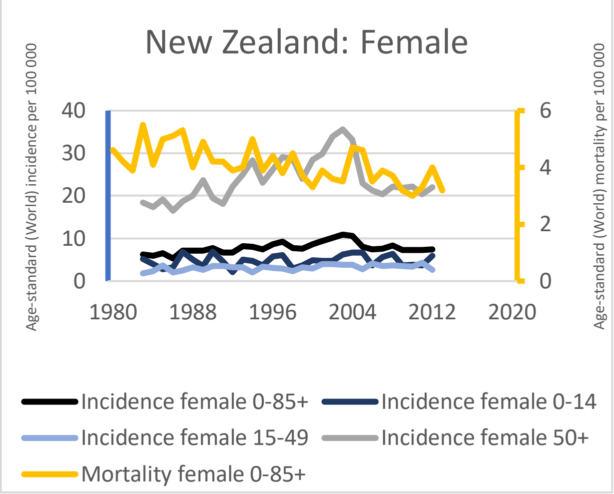

Northern America

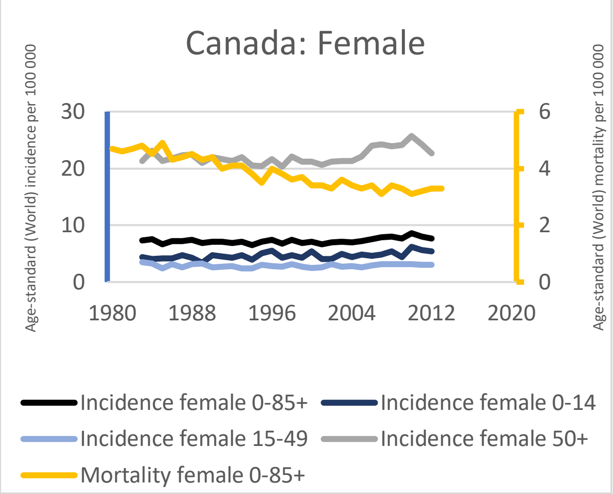

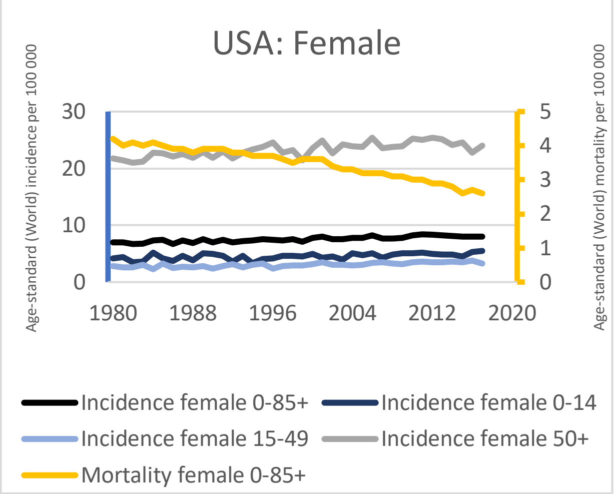

Southern America

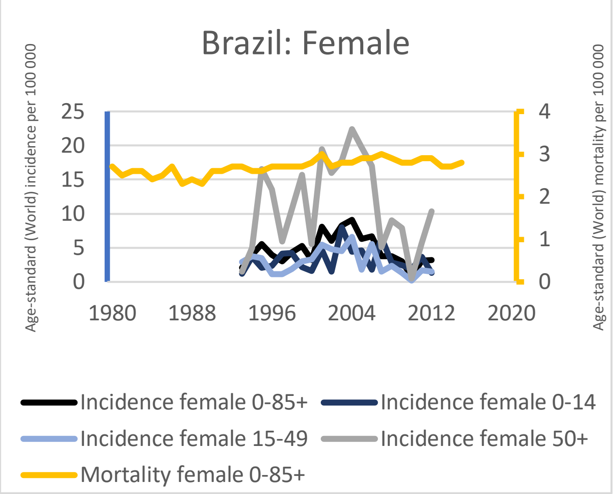

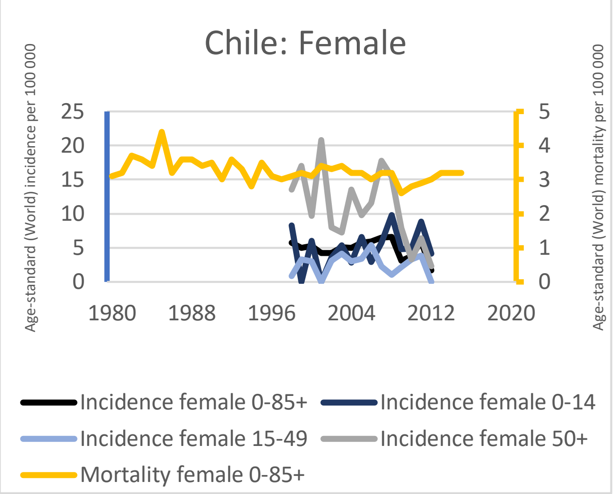

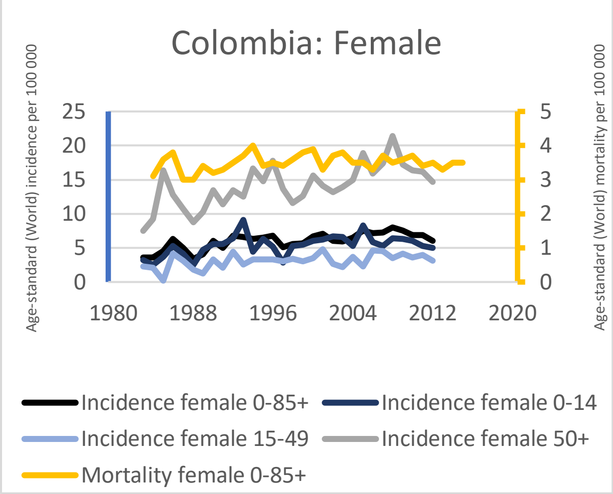

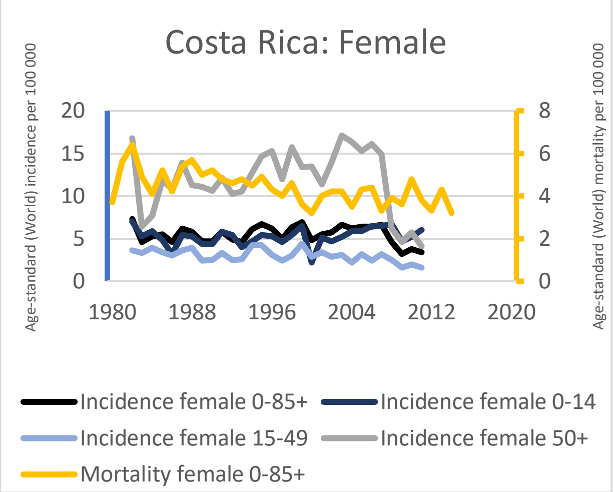

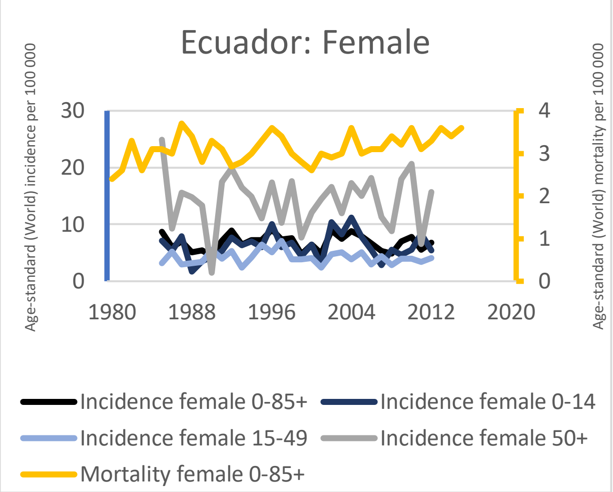

Northern Europe

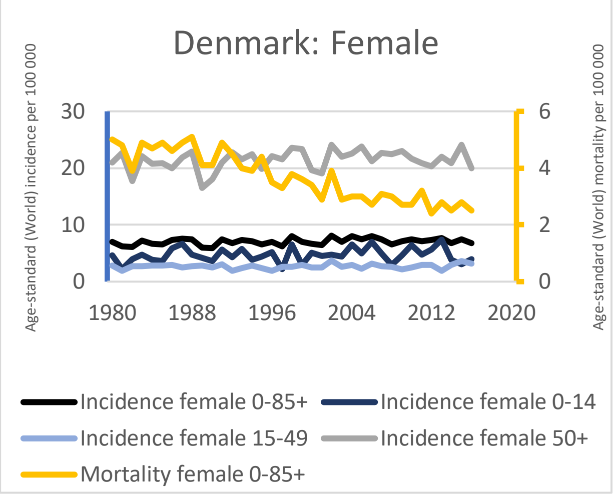
Denmark: Female
Age-standard (World) incidence per 100 000
Age-standard (World) mortality per 100 000
30
20
10
0
6
4
2
0
1980
1988
1996
2004
2012
2020
Incidence female 0-85+
Incidence female 0-14
Incidence female 15-49
Incidence female 50+
Mortality female 0-85+

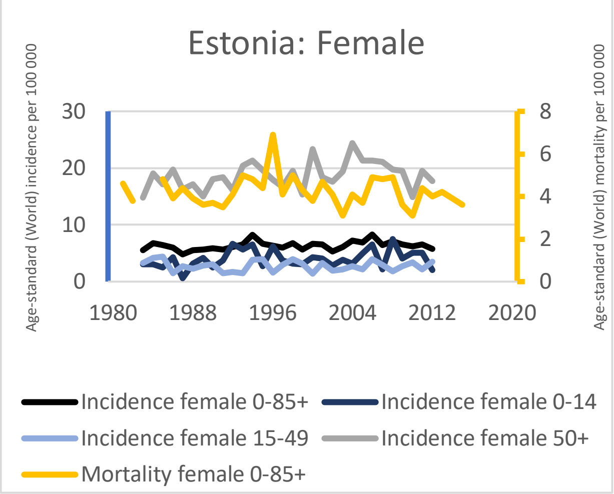
Estonia: Female
Age-standard (World) incidence per 100 000
Age-standard (World) mortality per 100 000
30
20
10
0
8
6
4
2
0
1980
1988
1996
2004
2012
2020
Incidence female 0-85+
Incidence female 0-14
Incidence female 15-49
Incidence female 50+
Mortality female 0-85+

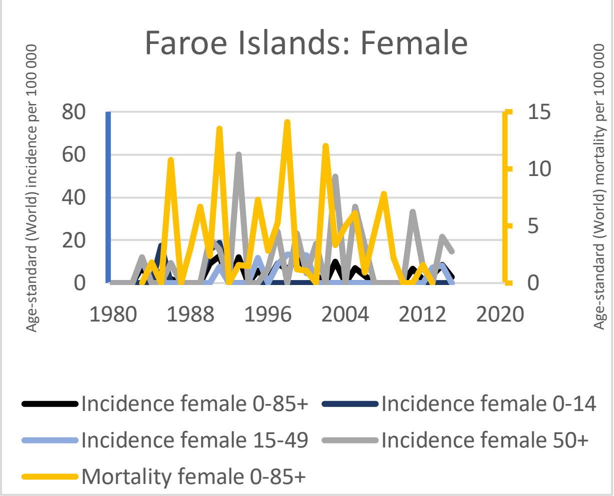
Faroe Islands: Female
Age-standard (World) incidence per 100 000
Age-standard (World) mortality per 100 000
80
60
40
20
0
15
10
5
0
1980
1988
1996
2004
2012
2020
Incidence female 0-85+
Incidence female 0-14
Incidence female 15-49
Incidence female 50+
Mortality female 0-85+

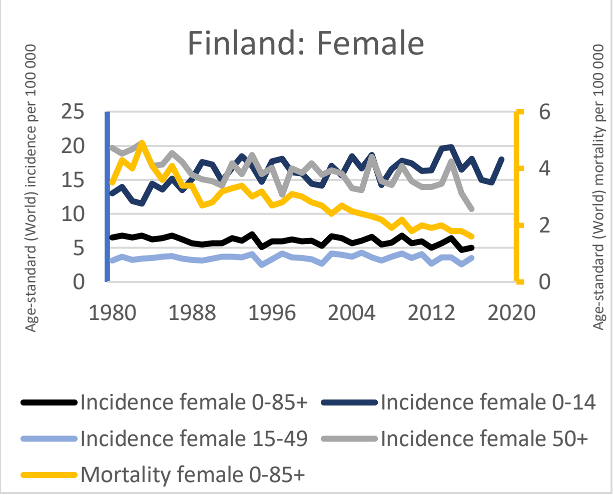
Finland: Female
Age-standard (World) incidence per 100 000
Age-standard (World) mortality per 100 000
25
20
15
10
5
0
6
4
2
0
1980
1988
1996
2004
2012
2020
Incidence female 0-85+
Incidence female 0-14
Incidence female 15-49
Incidence female 50+
Mortality female 0-85+

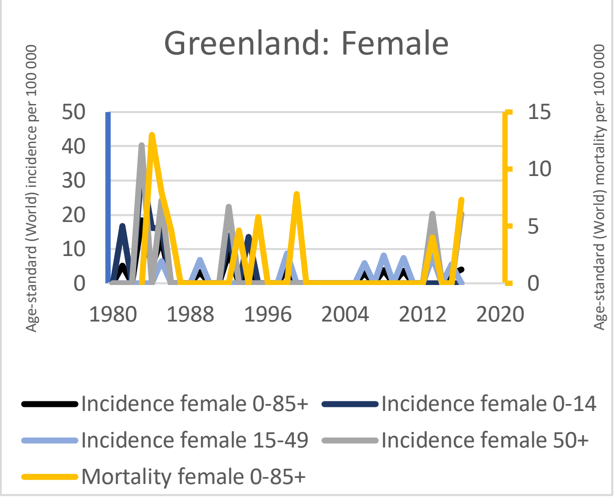
Greenland: Female
Age-standard (World) incidence per 100 000
Age-standard (World) mortality per 100 000
50
40
30
20
10
0
15
10
5
0
1980
1988
1996
2004
2012
2020
Incidence female 0-85+
Incidence female 0-14
Incidence female 15-49
Incidence female 50+
Mortality female 0-85+

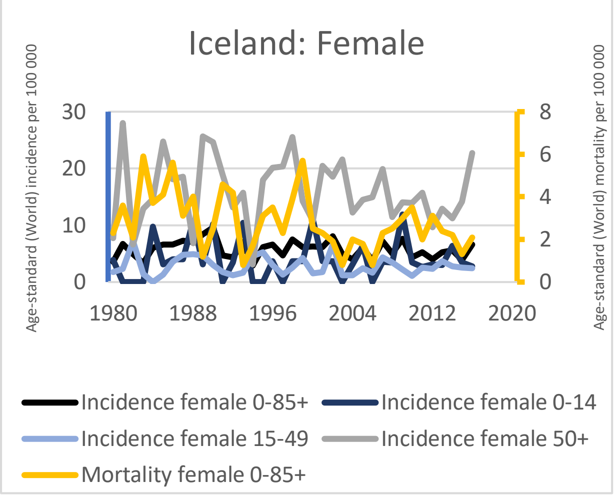
Iceland: Female
Age-standard (World) incidence per 100 000
Age-standard (World) mortality per 100 000
30
20
10
0
8
6
4
2
0
1980
1988
1996
2004
2012
2020
Incidence female 0-85+
Incidence female 0-14
Incidence female 15-49
Incidence female 50+
Mortality female 0-85+

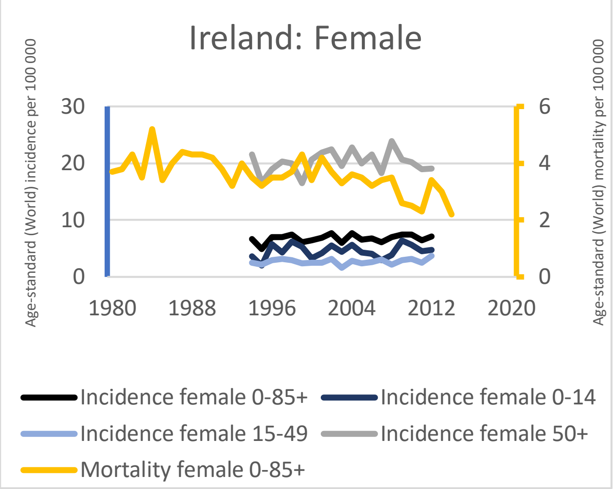

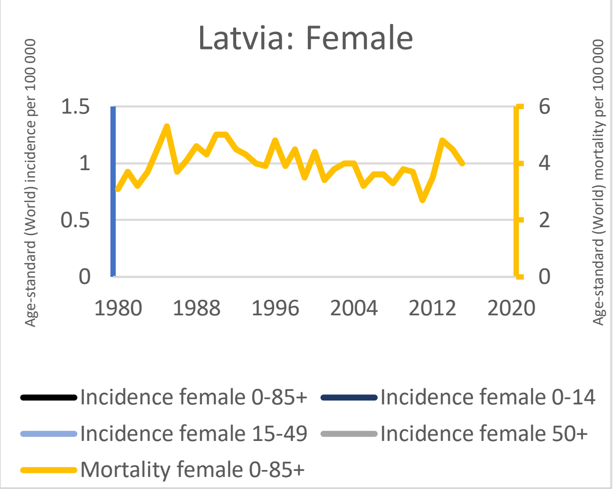

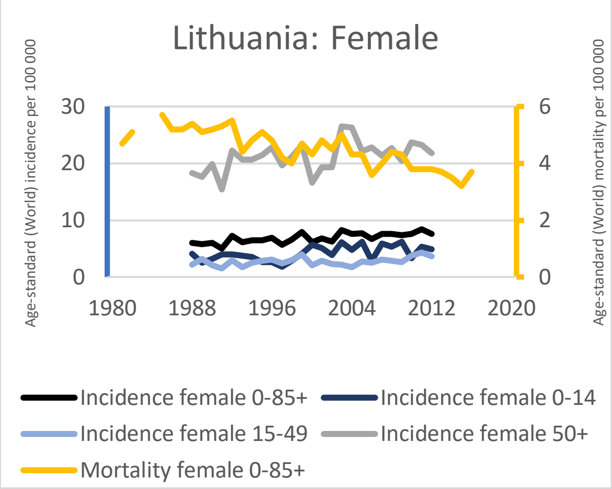

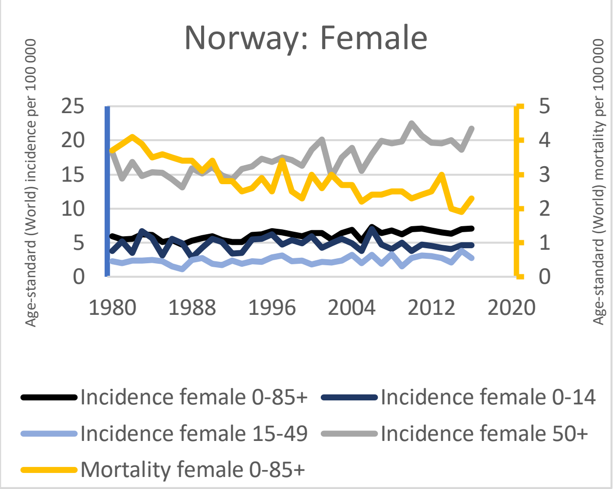

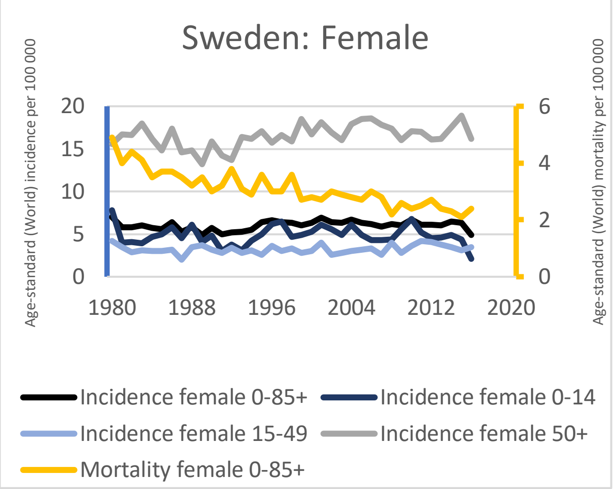

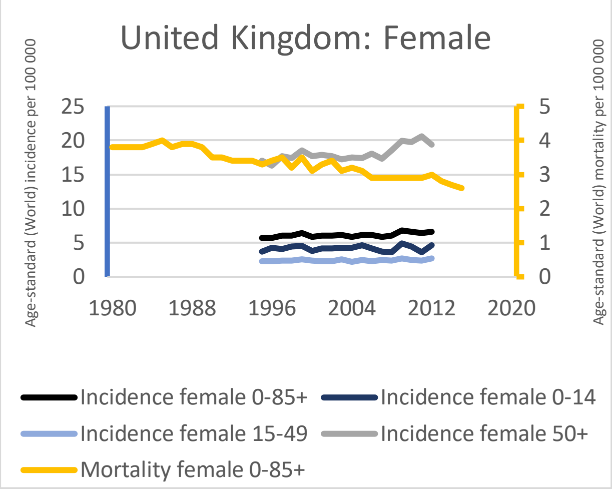

Western Europe

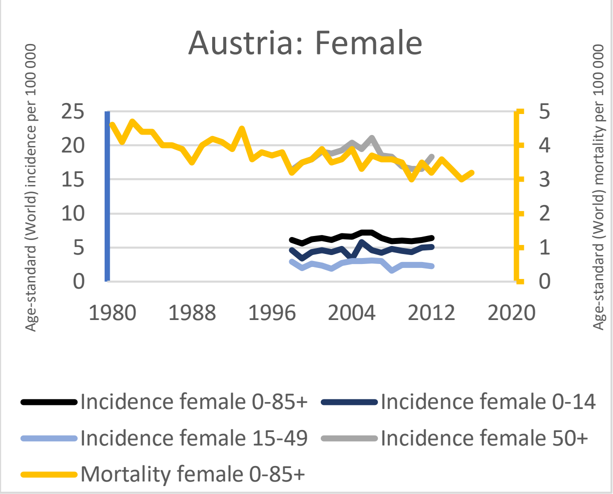

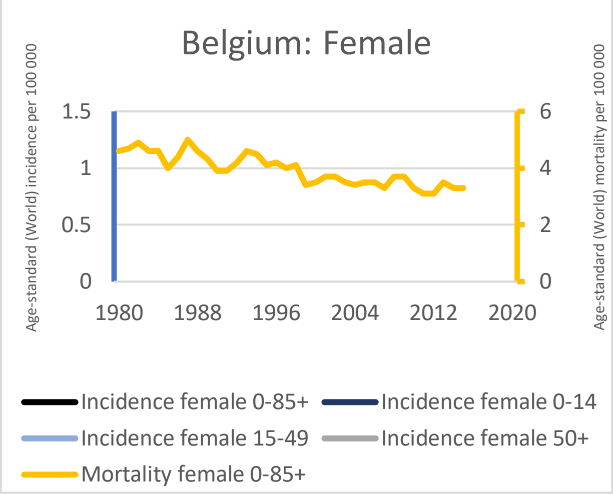

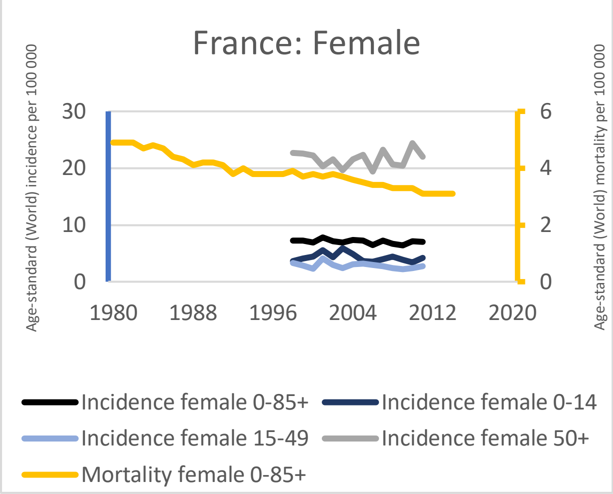

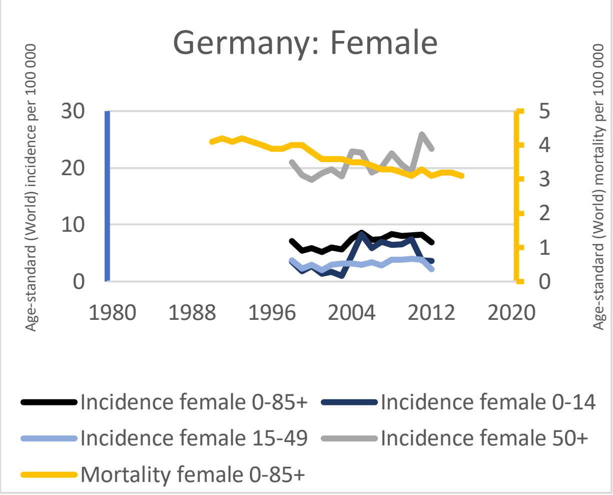

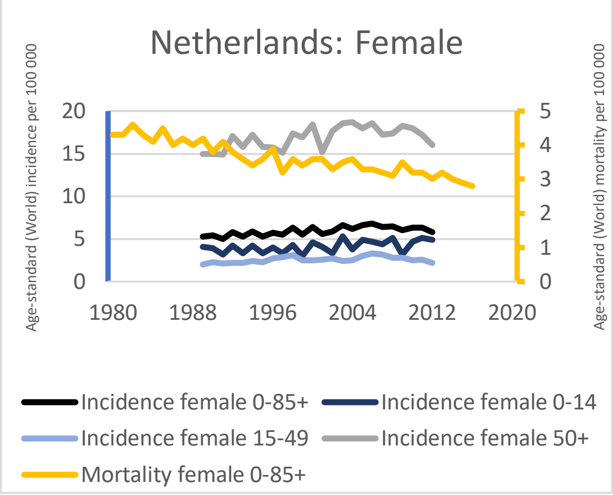

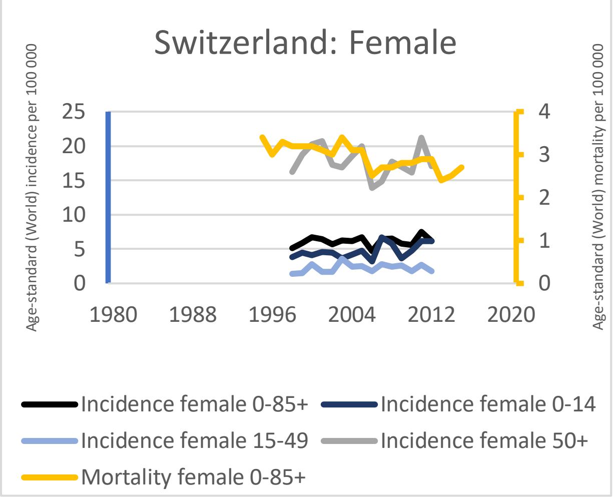

Southern Europe

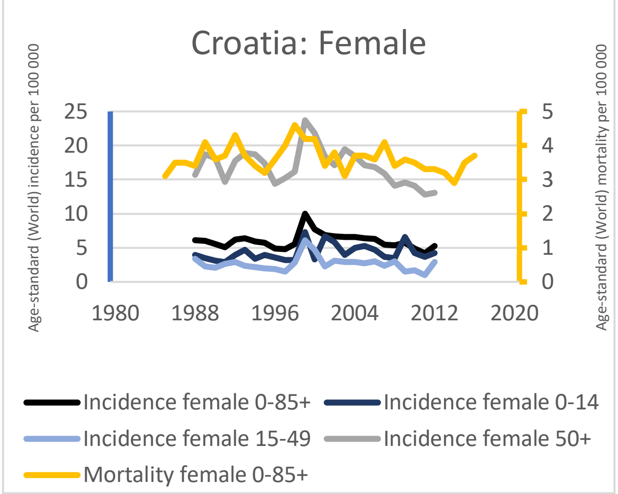
Croatia: Female
Age-standard (World) incidence per 100 000
Age-standard (World) mortality per 100 000
Incidence female 0-85+
Incidence female 0-14
Incidence female 15-49
Incidence female 50+
Mortality female 0-85+

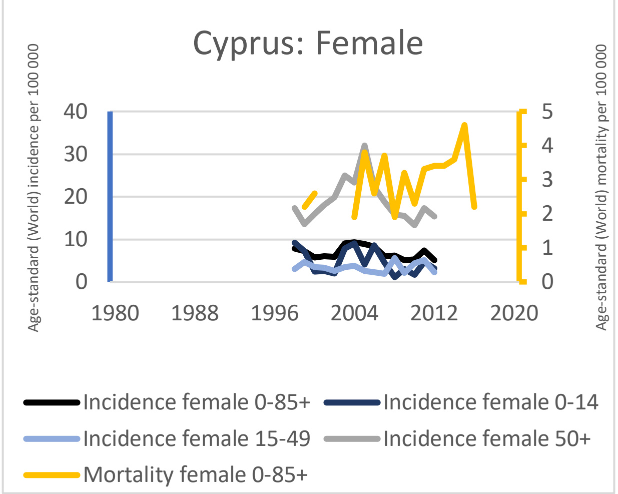
Cyprus: Female
Age-standard (World) incidence per 100 000
Age-standard (World) mortality per 100 000
Incidence female 0-85+
Incidence female 0-14
Incidence female 15-49
Incidence female 50+
Mortality female 0-85+

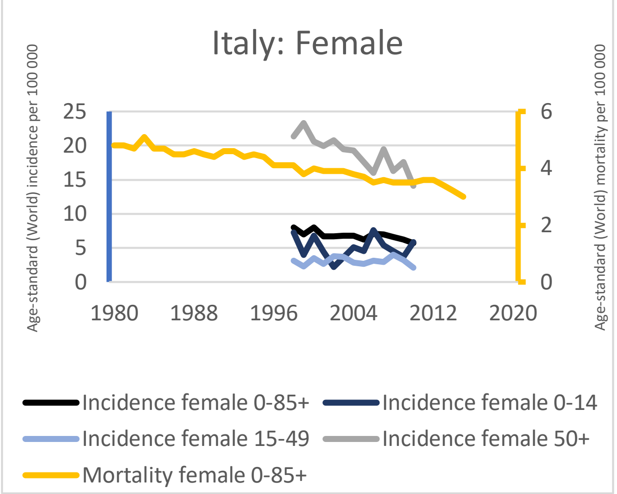
Italy: Female
Age-standard (World) incidence per 100 000
Age-standard (World) mortality per 100 000
Incidence female 0-85+
Incidence female 0-14
Incidence female 15-49
Incidence female 50+
Mortality female 0-85+

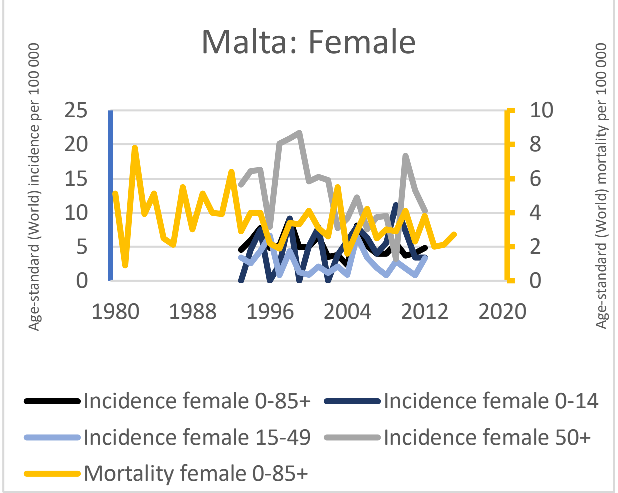
Malta: Female
Age-standard (World) incidence per 100 000
Age-standard (World) mortality per 100 000
Incidence female 0-85+
Incidence female 0-14
Incidence female 15-49
Incidence female 50+
Mortality female 0-85+

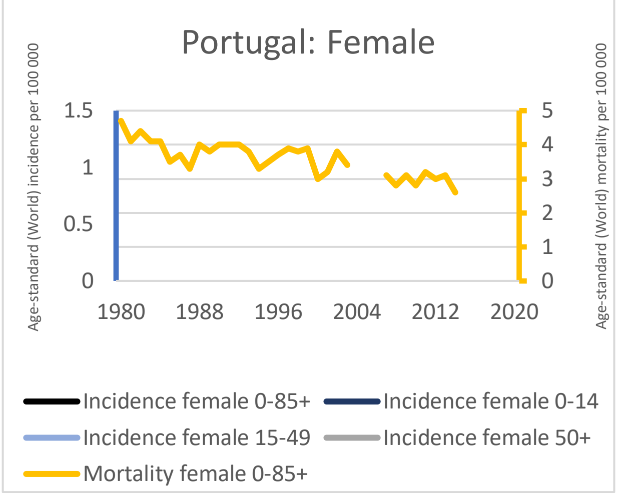
Portugal: Female
Age-standard (World) incidence per 100 000
Age-standard (World) mortality per 100 000
Incidence female 0-85+
Incidence female 0-14
Incidence female 15-49
Incidence female 50+
Mortality female 0-85+

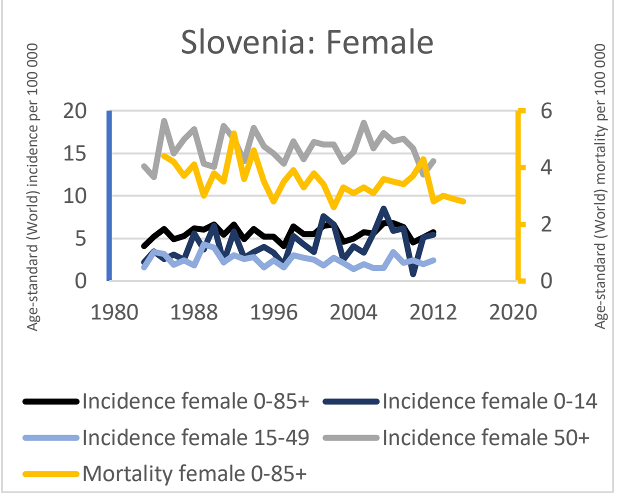
Slovenia: Female
Age-standard (World) incidence per 100 000
Age-standard (World) mortality per 100 000
Incidence female 0-85+
Incidence female 0-14
Incidence female 15-49
Incidence female 50+
Mortality female 0-85+

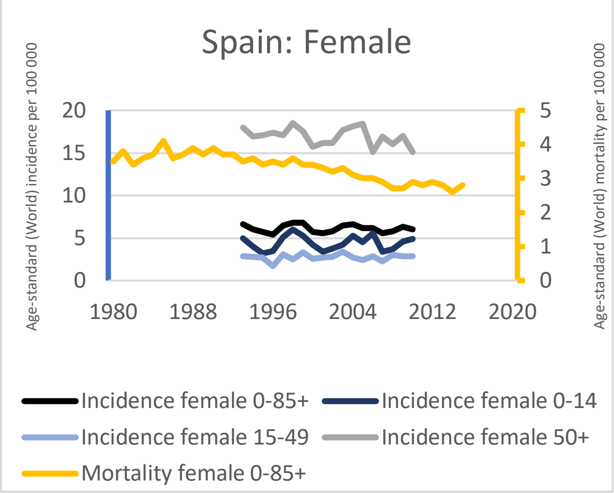
Spain: Female
Age-standard (World) incidence per 100 000
20
15
10
5
0
1980
1988
1996
2004
2012
2020
5
4
3
2
1
0
Age-standard (World) mortality per 100 000
Incidence female 0-85+
Incidence female 0-14
Incidence female 15-49
Incidence female 50+
Mortality female 0-85+

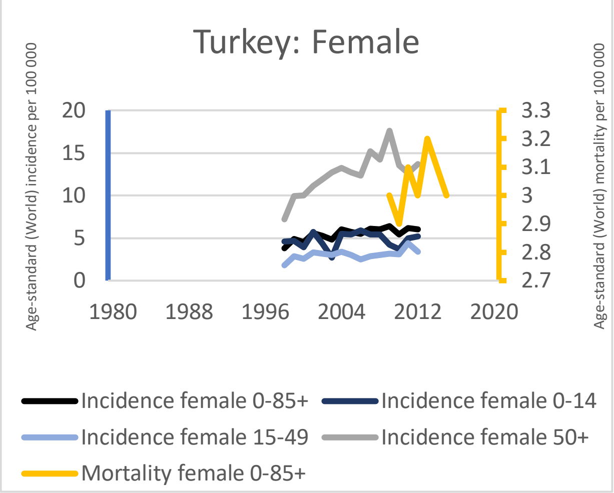
Turkey: Female
Age-standard (World) incidence per 100 000
20
15
10
5
0
1980
1988
1996
2004
2012
2020
3.3
3.2
3.1
3
2.9
2.8
2.7
Age-standard (World) mortality per 100 000
Incidence female 0-85+
Incidence female 0-14
Incidence female 15-49
Incidence female 50+
Mortality female 0-85+

Eastern Europe

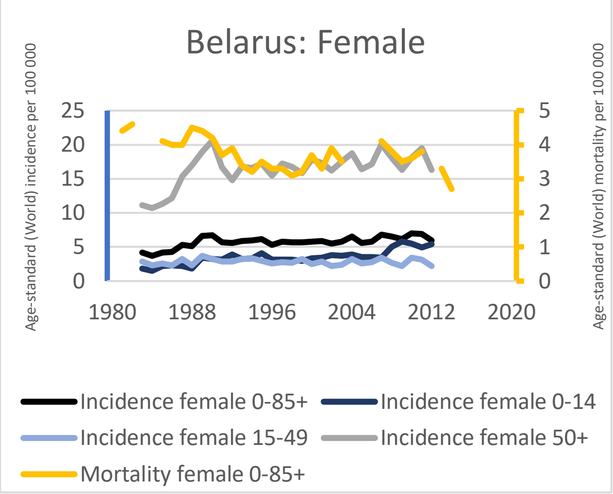

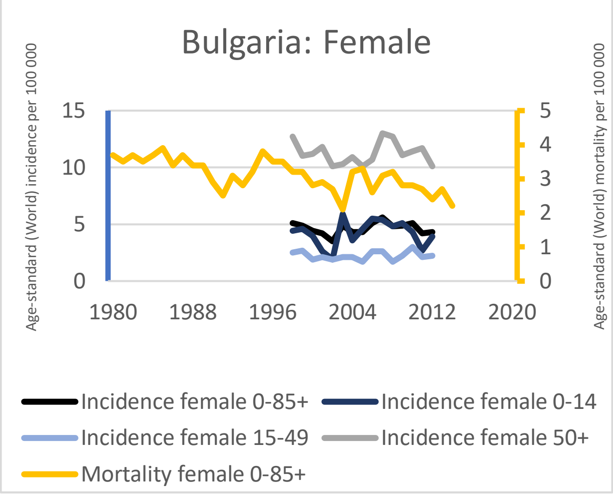

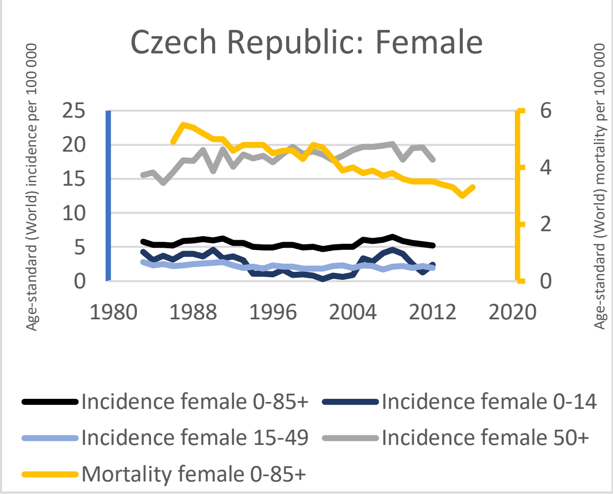

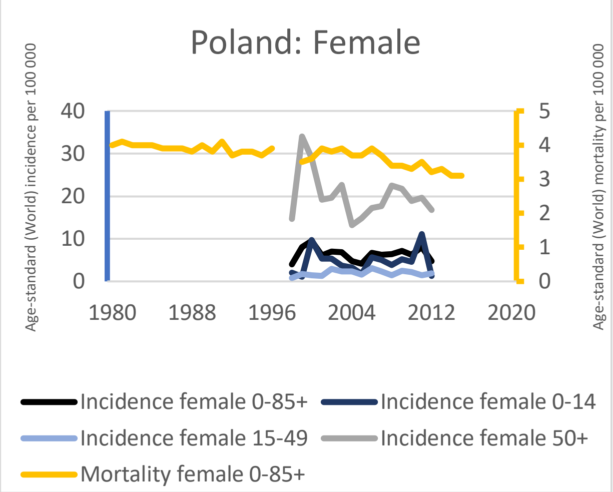

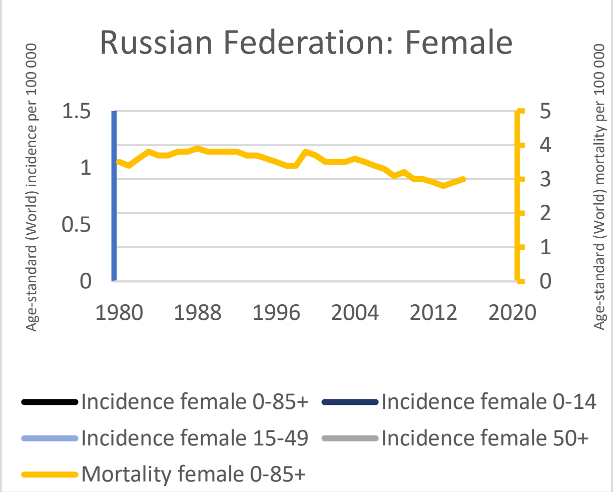

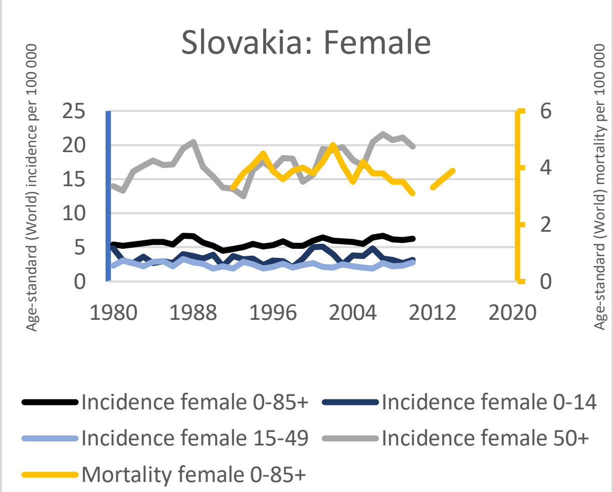

Africa		
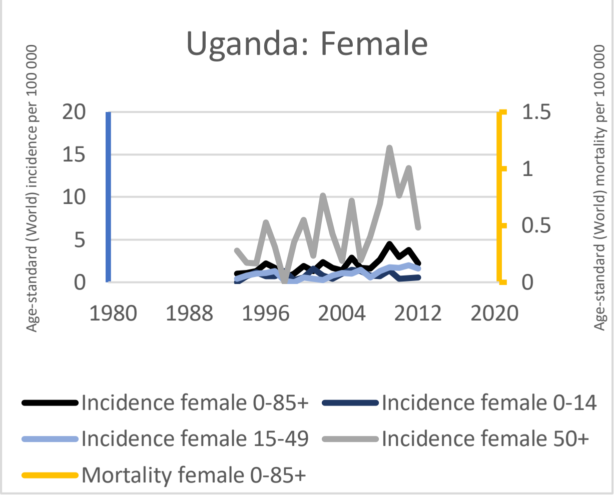		

Uganda: Female
Age-standard (World) incidence per 100 000
Age-standard (World) mortality per 100 000
20
15
10
5
0
1.5
1
0.5
0
1980
1988
1996
2004
2012
2020
Incidence female 0-85+
Incidence female 0-14
Incidence female 15-49
Incidence female 50+
Mortality female 0-85+

Supplementary Figure 2: The graphs of the joinpoint regression output

a.) Incidence male all ages

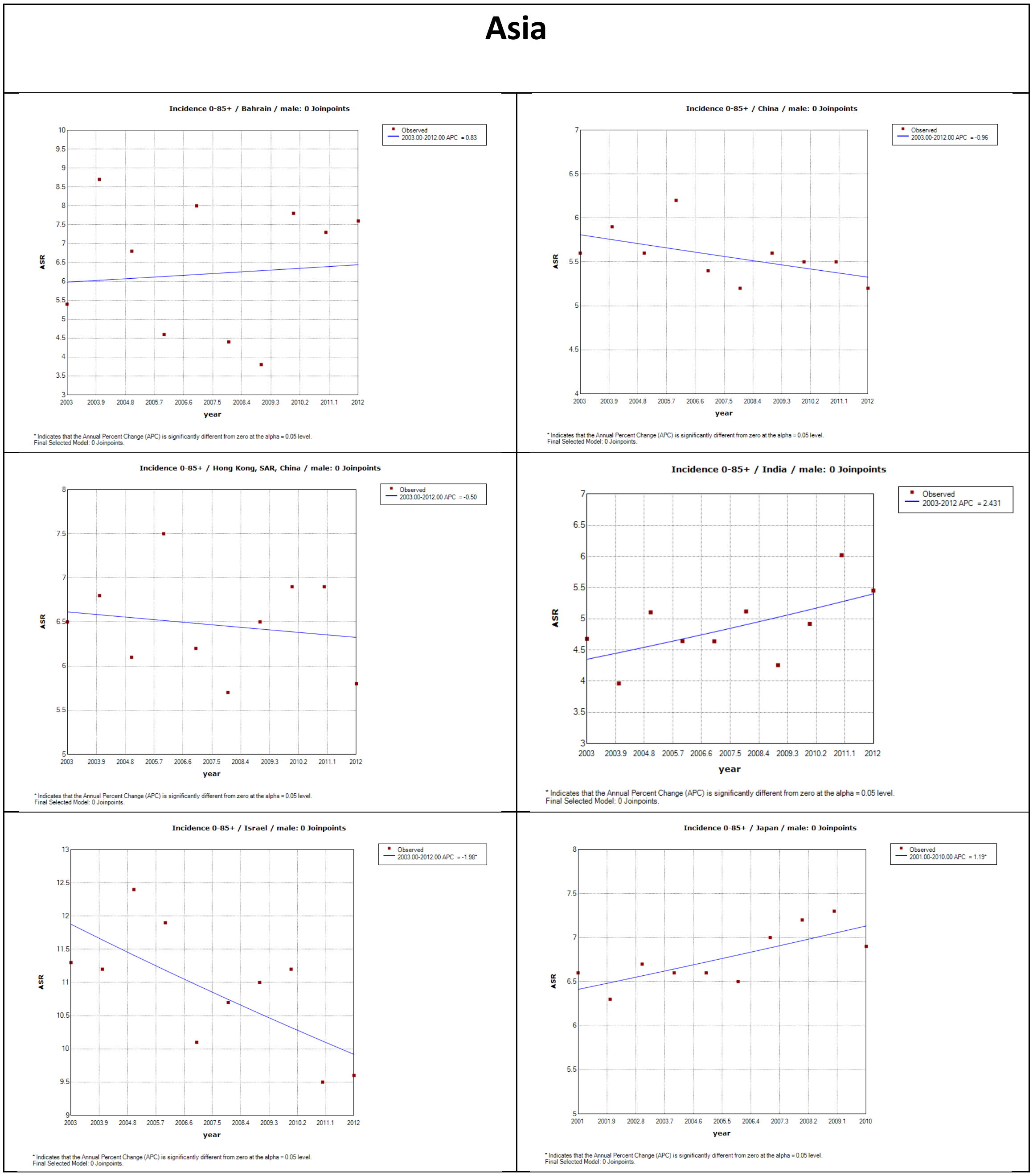

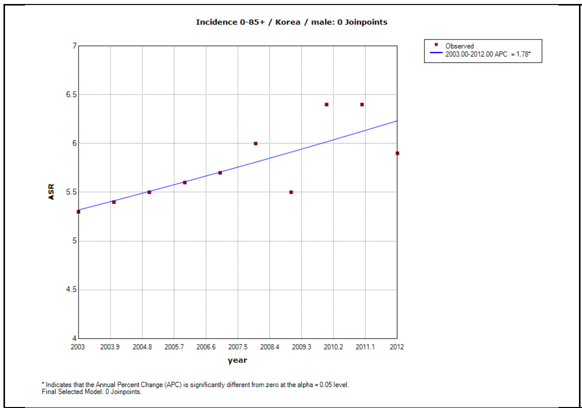
Incidence 0-85+ / Korea / male: 0 Joinpoints
Observed
2003.00-2012.00 APC = 1.78*
ASR
year
* Indicates that the Annual Percent Change (APC) is significantly different from zero at the alpha = 0.05 level.
Final Selected Model: 0 Joinpoints.

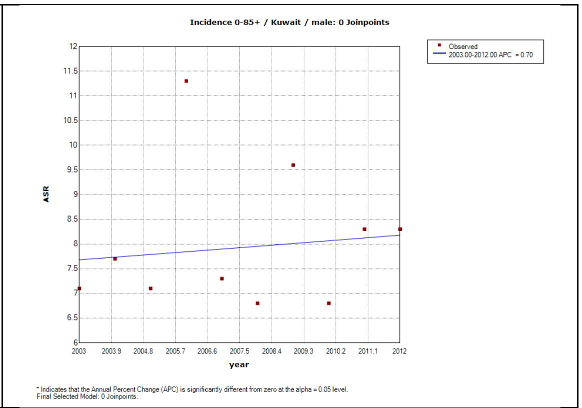
Incidence 0-85+ / Kuwait / male: 0 Joinpoints
Observed
2003.00-2012.00 APC = 0.70
ASR
year
* Indicates that the Annual Percent Change (APC) is significantly different from zero at the alpha = 0.05 level.
Final Selected Model: 0 Joinpoints.

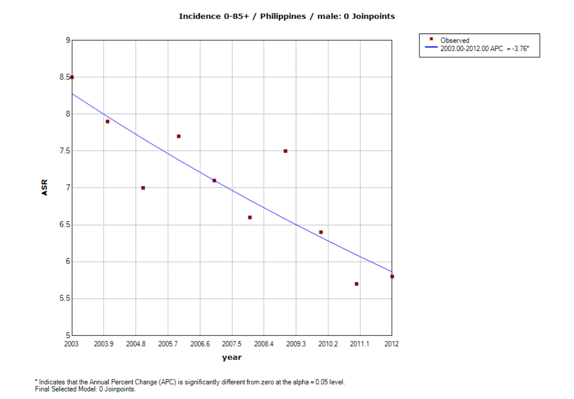
Incidence 0-85+ / Philippines / male: 0 Joinpoints
Observed
2003.00-2012.00 APC = -3.76*
ASR
year
* Indicates that the Annual Percent Change (APC) is significantly different from zero at the alpha = 0.05 level.
Final Selected Model: 0 Joinpoints.

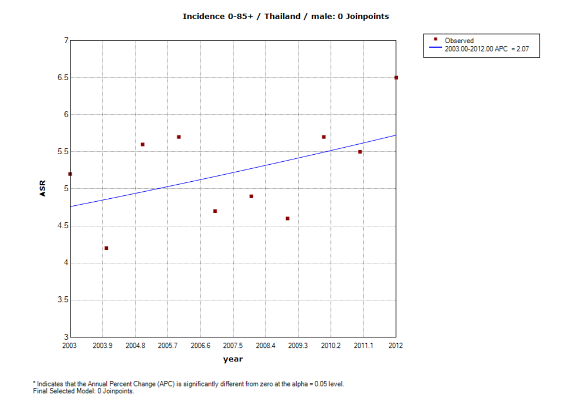
Incidence 0-85+ / Thailand / male: 0 Joinpoints
Observed
2003.00-2012.00 APC = 2.07
ASR
year
* Indicates that the Annual Percent Change (APC) is significantly different from zero at the alpha = 0.05 level.
Final Selected Model: 0 Joinpoints.

Oceania

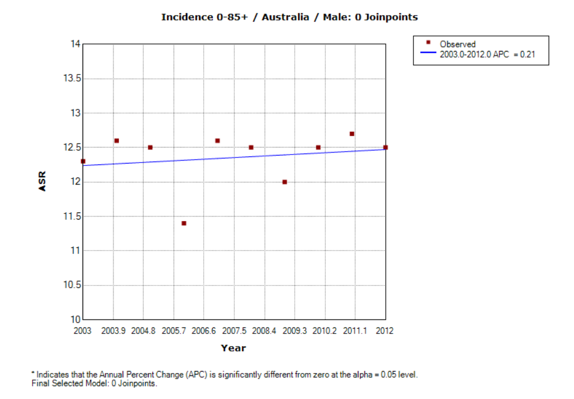
Incidence 0-85+ / Australia / Male: 0 Joinpoints
Observed
2003.0-2012.0 APC = 0.21
ASR
Year
* Indicates that the Annual Percent Change (APC) is significantly different from zero at the alpha = 0.05 level.
Final Selected Model: 0 Joinpoints.

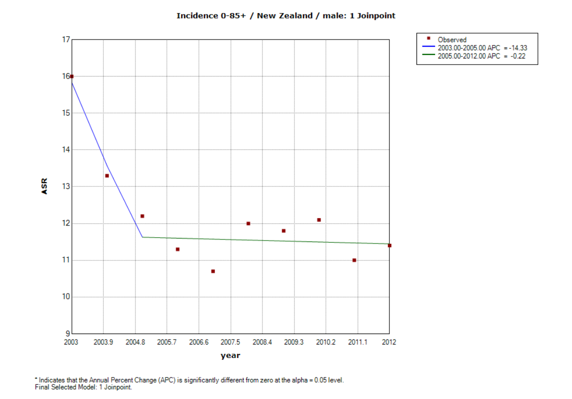
Incidence 0-85+ / New Zealand / male: 1 Joinpoint
Observed
2003.00-2005.00 APC = -14.33
2005.00-2012.00 APC = -0.22
ASR
year
* Indicates that the Annual Percent Change (APC) is significantly different from zero at the alpha = 0.05 level.
Final Selected Model: 1 Joinpoint.

Northern America

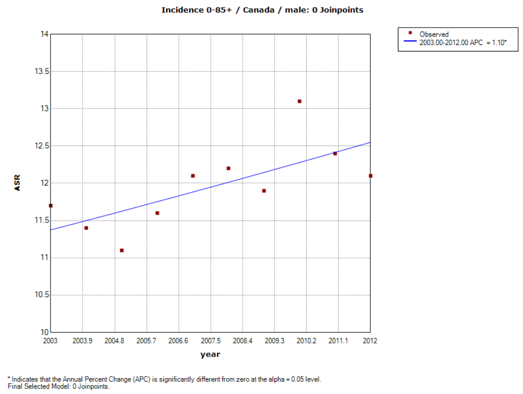

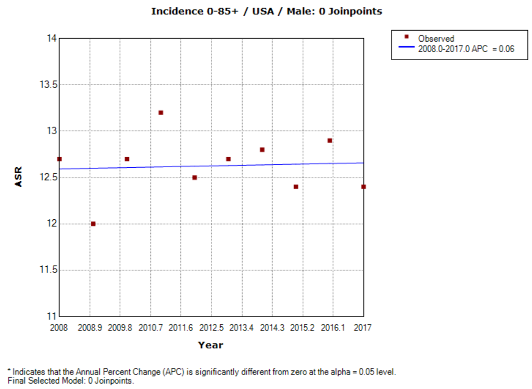

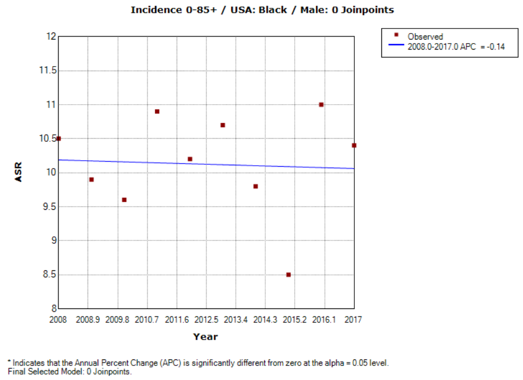

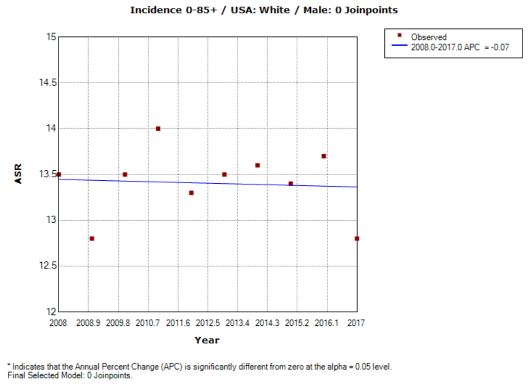

Southern America

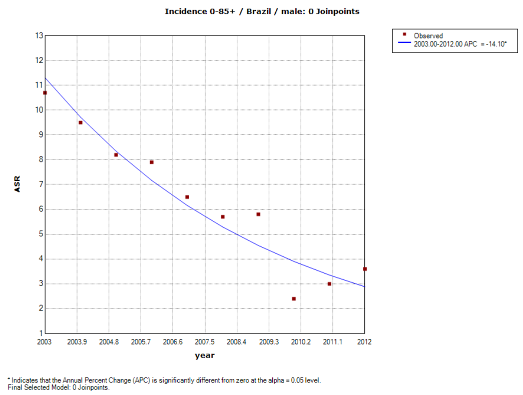

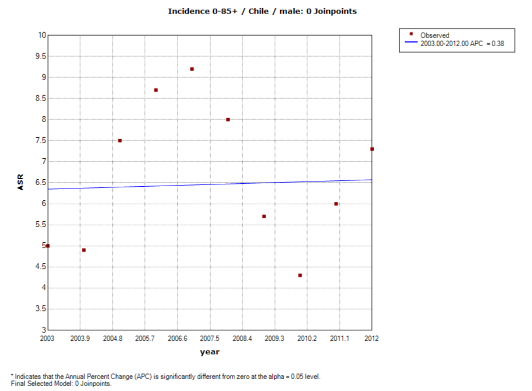

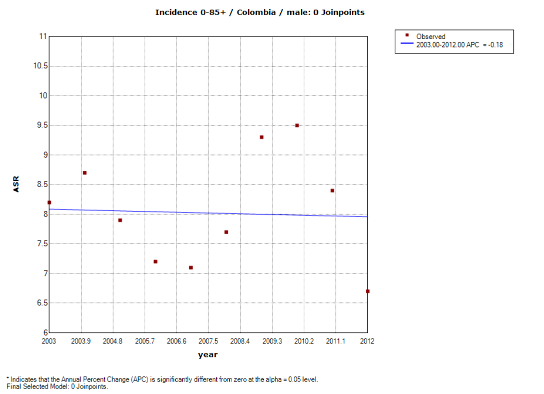

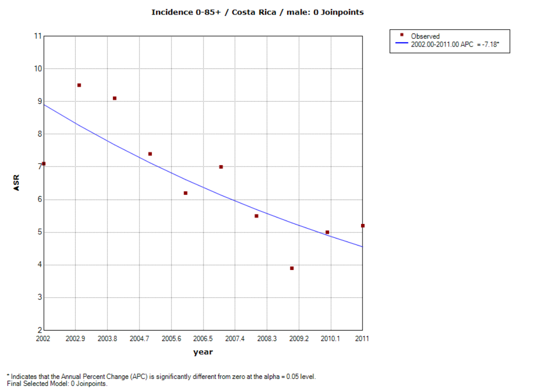

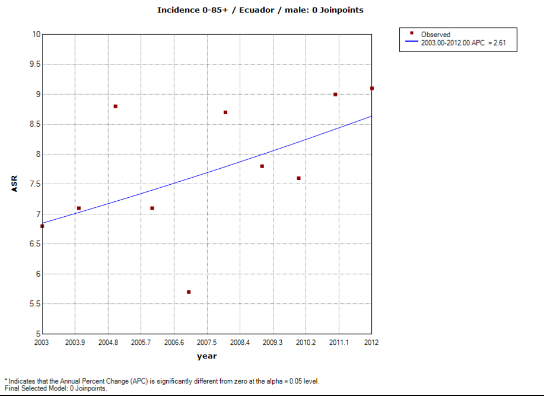

Northern Europe

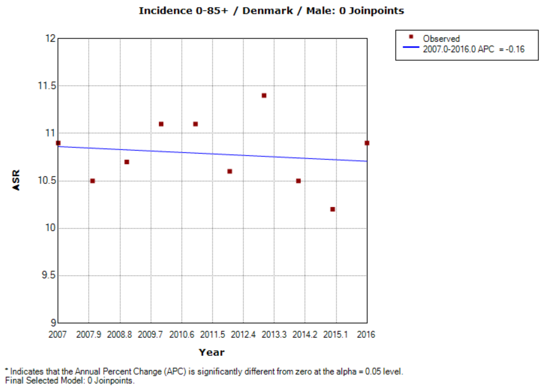

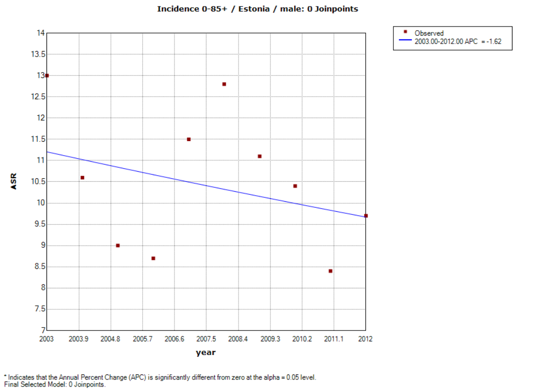

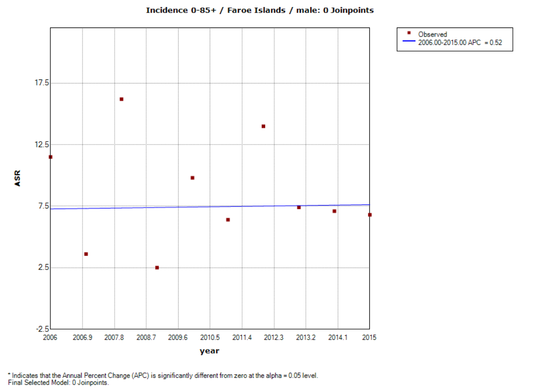

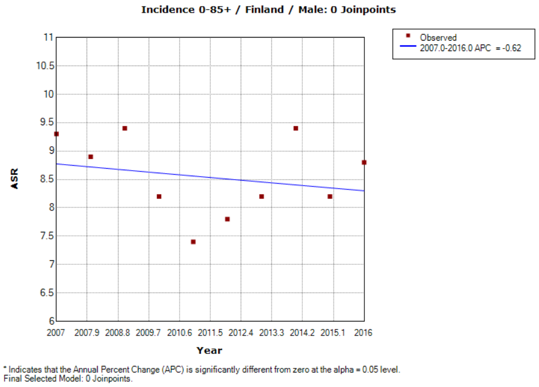

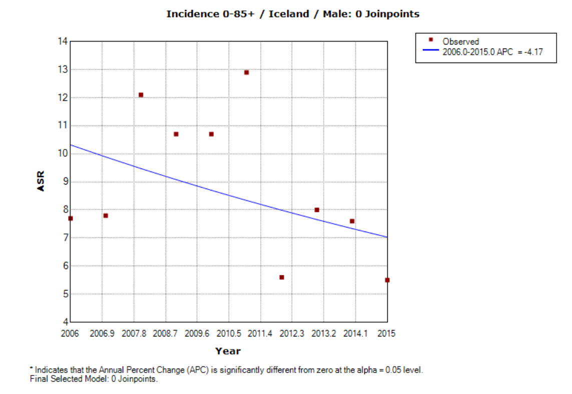

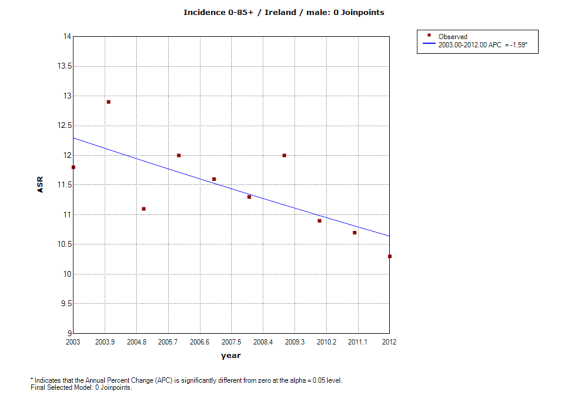

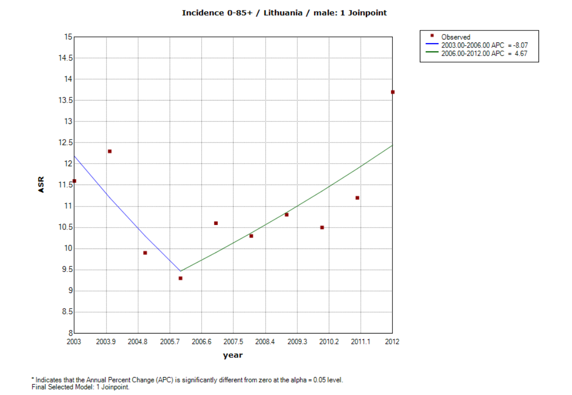

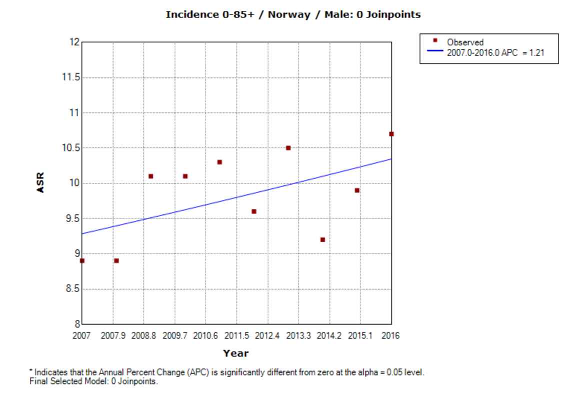

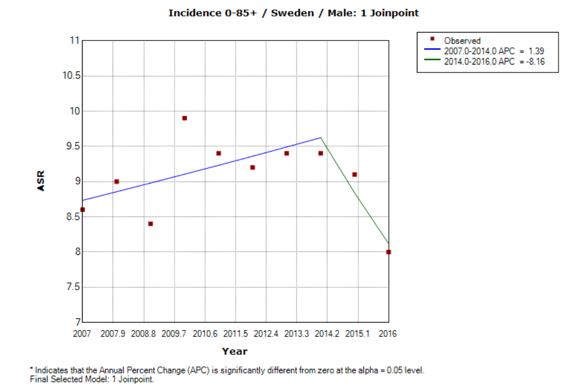

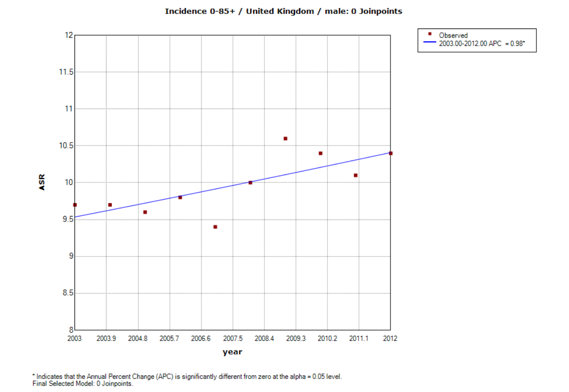

Western Europe

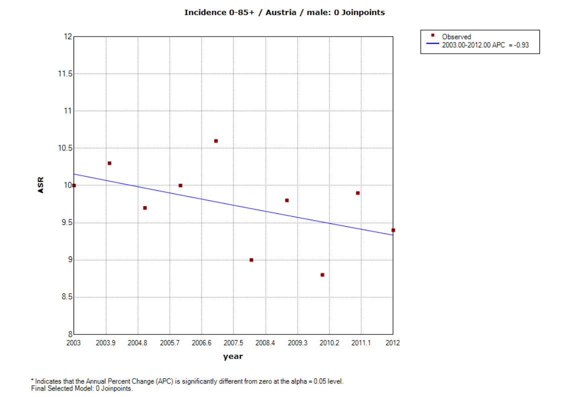

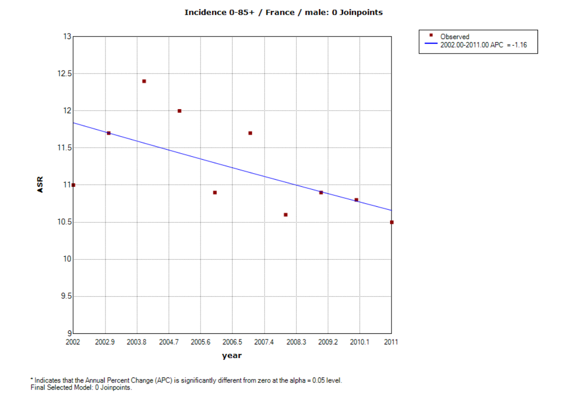

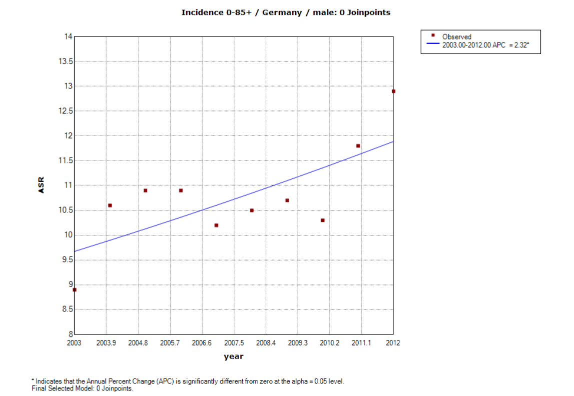

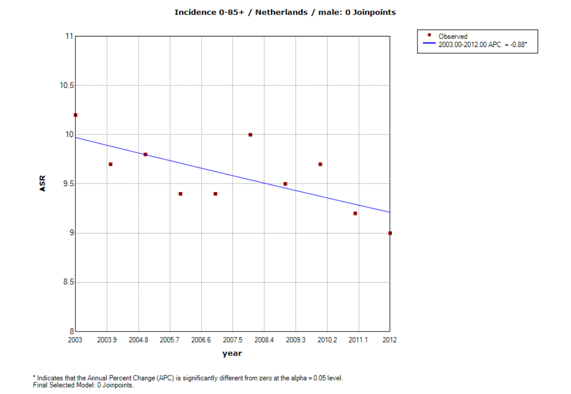

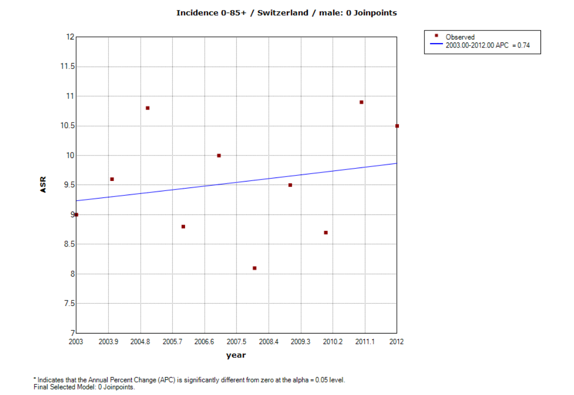

Southern Europe

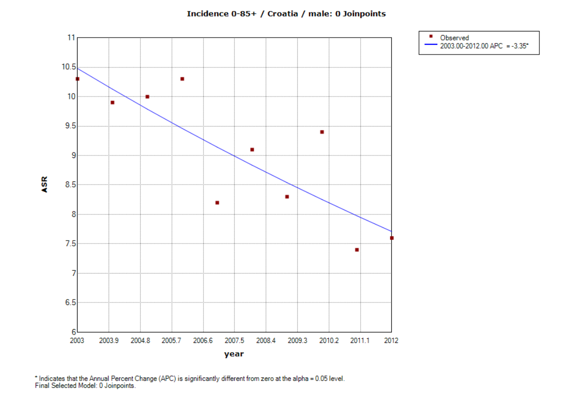

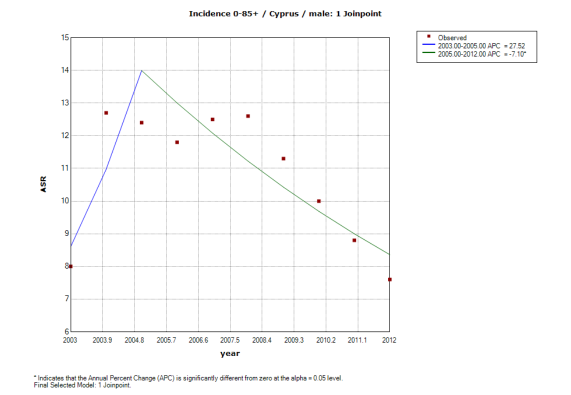

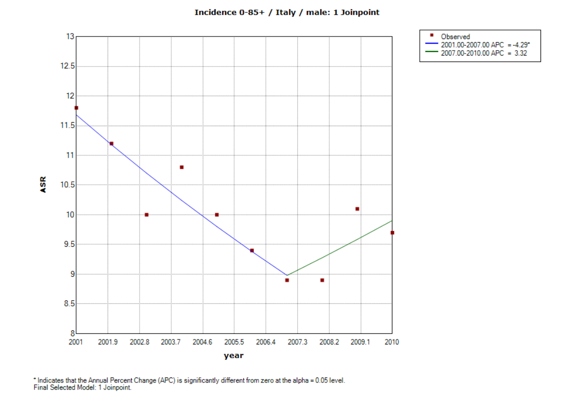

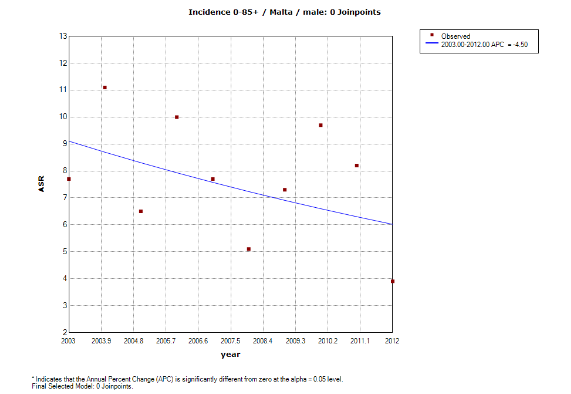

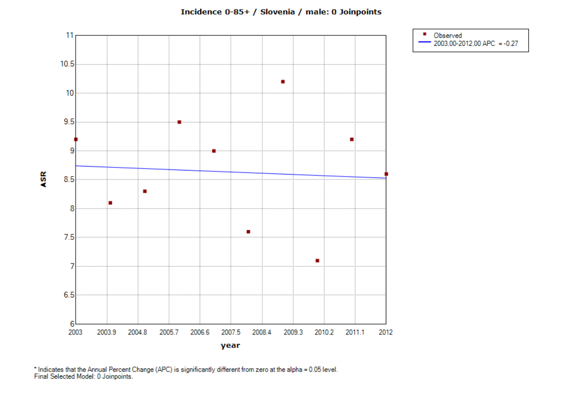

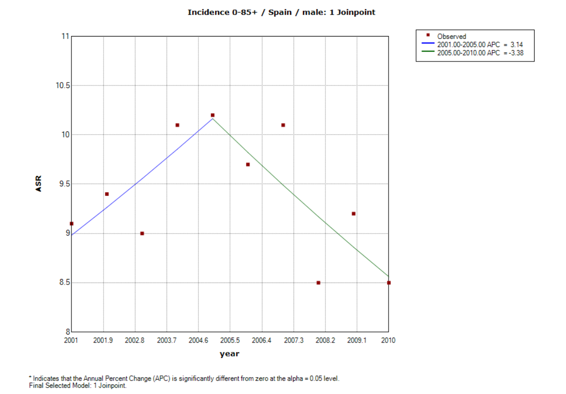

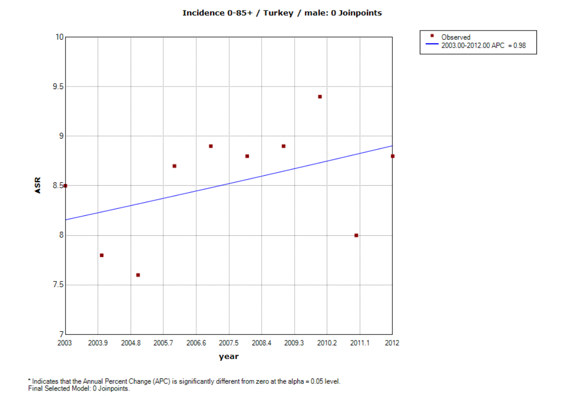

Eastern Europe

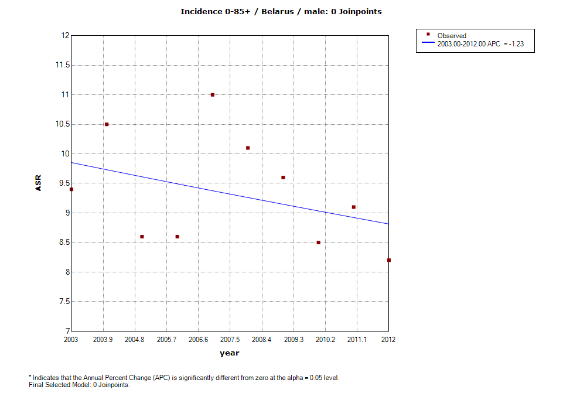

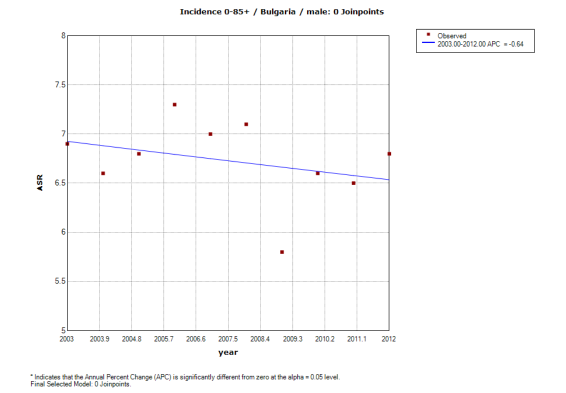

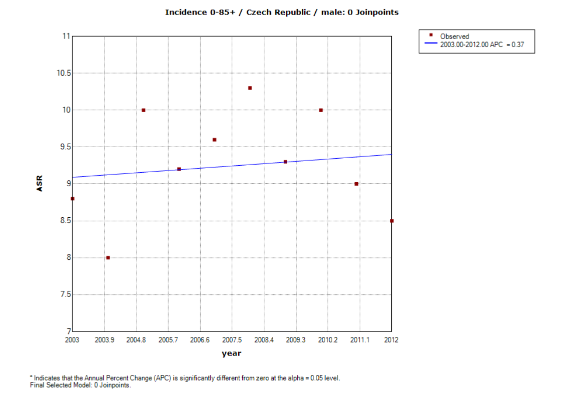

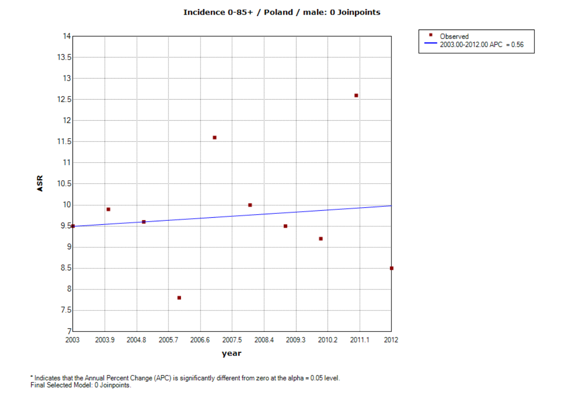

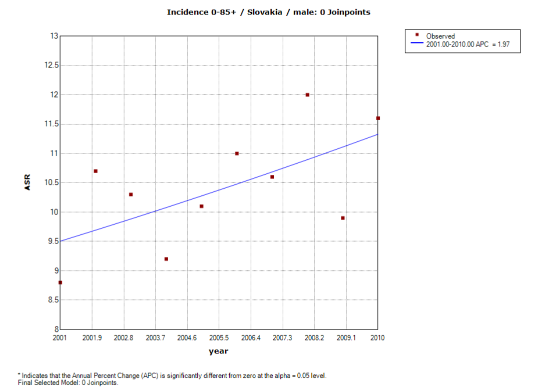
Incidence 0-85+ / Slovakia / male: 0 Joinpoints
Observed
2001.00-2010.00 APC = 1.97
ASR
year
* Indicates that the Annual Percent Change (APC) is significantly different from zero at the alpha = 0.05 level.
Final Selected Model: 0 Joinpoints.

Africa

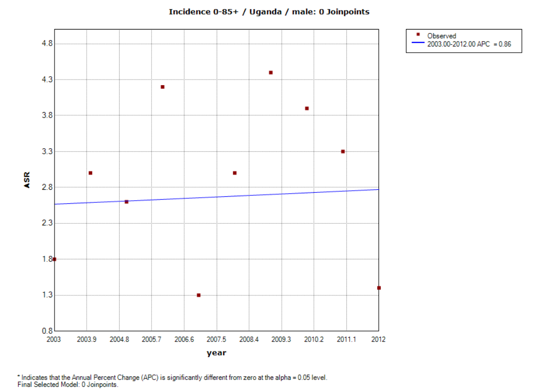
Incidence 0-85+ / Uganda / male: 0 Joinpoints
Observed
2003.00-2012.00 APC = 0.86
ASR
year
* Indicates that the Annual Percent Change (APC) is significantly different from zero at the alpha = 0.05 level.
Final Selected Model: 0 Joinpoints.

b.) Incidence female all ages

Asia

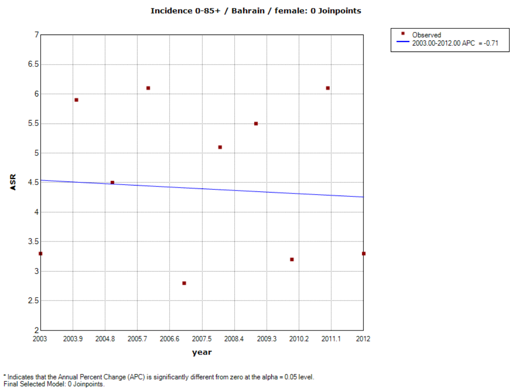

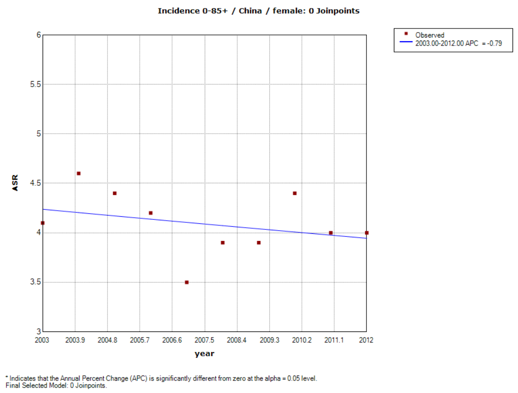

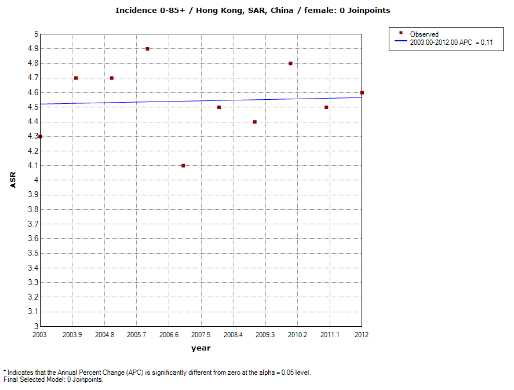

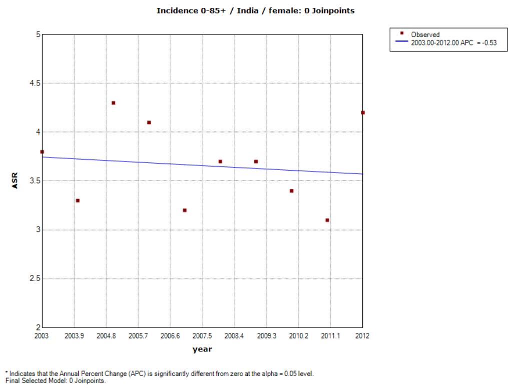

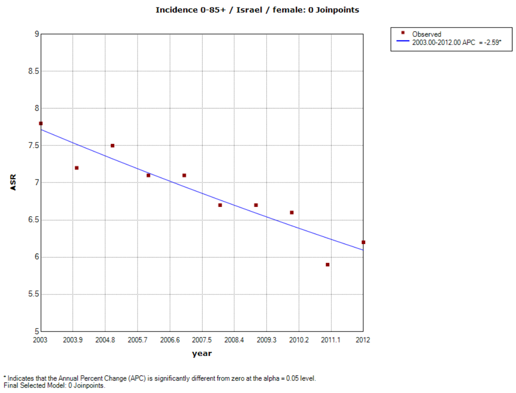

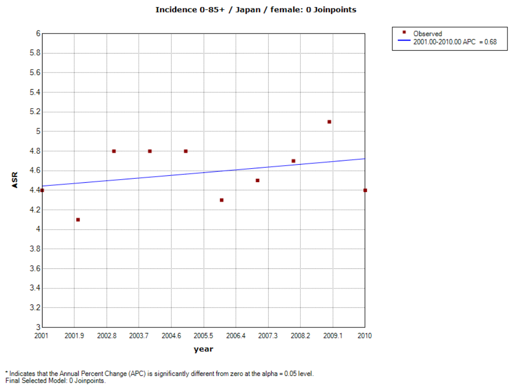

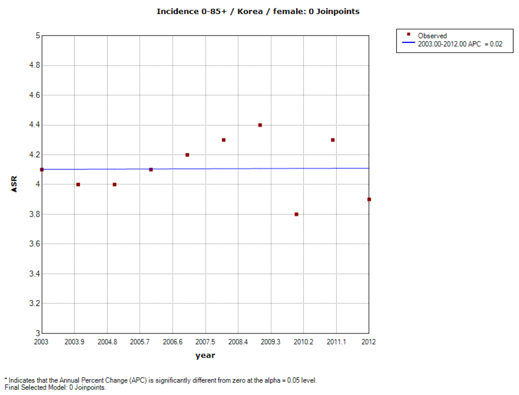
Incidence 0-85+ / Korea / female: 0 Joinpoints
Observed
2003.00-2012.00 APC = 0.02
ASR
year
* Indicates that the Annual Percent Change (APC) is significantly different from zero at the alpha = 0.05 level.
Final Selected Model: 0 Joinpoints.

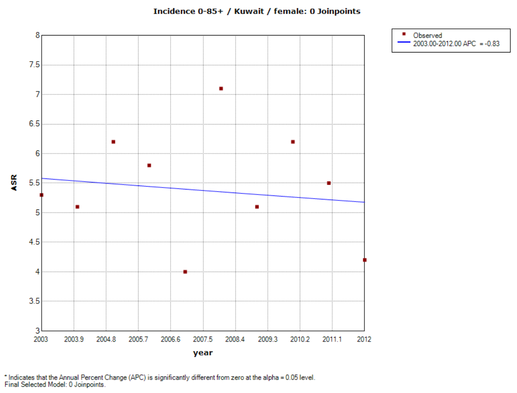
Incidence 0-85+ / Kuwait / female: 0 Joinpoints
Observed
2003.00-2012.00 APC = -0.83
ASR
year
* Indicates that the Annual Percent Change (APC) is significantly different from zero at the alpha = 0.05 level.
Final Selected Model: 0 Joinpoints.

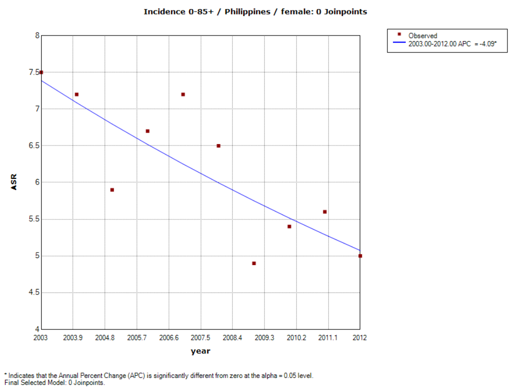
Incidence 0-85+ / Philippines / female: 0 Joinpoints
Observed
2003.00-2012.00 APC = -4.09*
ASR
year
* Indicates that the Annual Percent Change (APC) is significantly different from zero at the alpha = 0.05 level.
Final Selected Model: 0 Joinpoints.

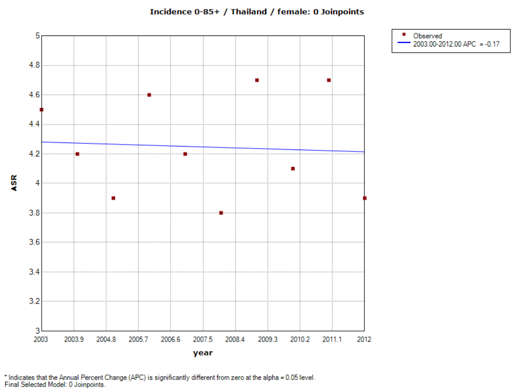
Incidence 0-85+ / Thailand / female: 0 Joinpoints
Observed
2003.00-2012.00 APC = -0.17
ASR
year
* Indicates that the Annual Percent Change (APC) is significantly different from zero at the alpha = 0.05 level.
Final Selected Model: 0 Joinpoints.

Oceania

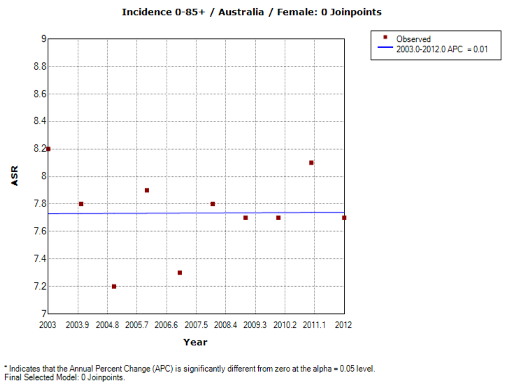
Incidence 0-85+ / Australia / Female: 0 Joinpoints
Observed
2003.0-2012.0 APC = 0.01
ASR
Year
* Indicates that the Annual Percent Change (APC) is significantly different from zero at the alpha = 0.05 level.
Final Selected Model: 0 Joinpoints.

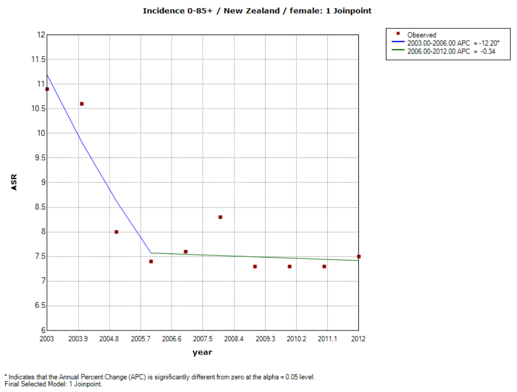
Incidence 0-85+ / New Zealand / female: 1 Joinpoint
Observed
2003.00-2006.00 APC = -12.20*
2006.00-2012.00 APC = -0.34
ASR
year
* Indicates that the Annual Percent Change (APC) is significantly different from zero at the alpha = 0.05 level.
Final Selected Model: 1 Joinpoint.

Northern America

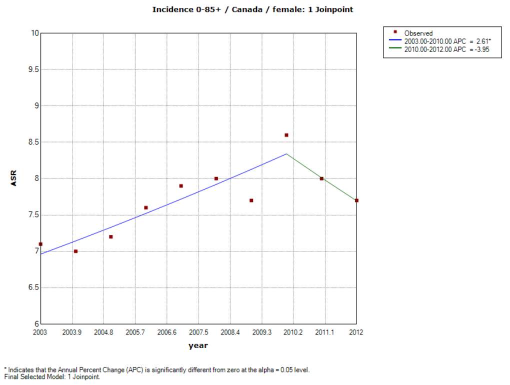

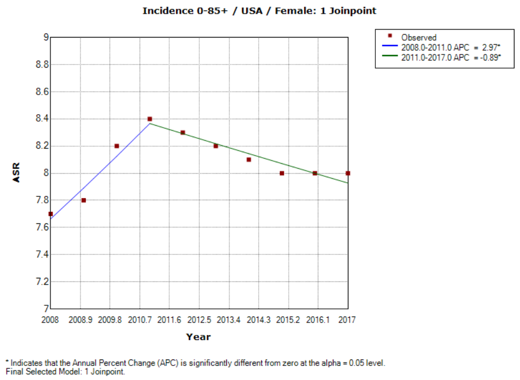

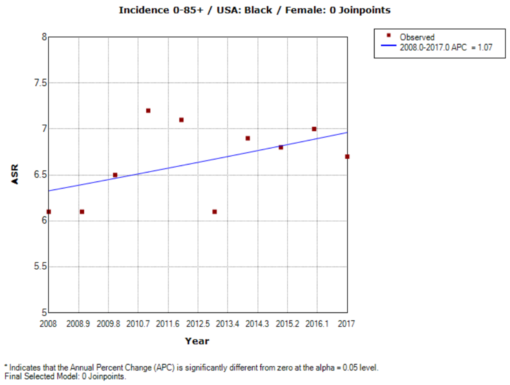

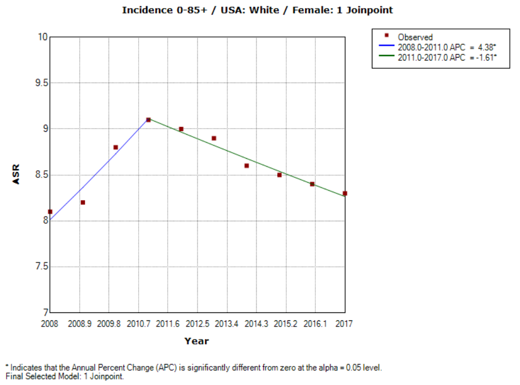

Southern America

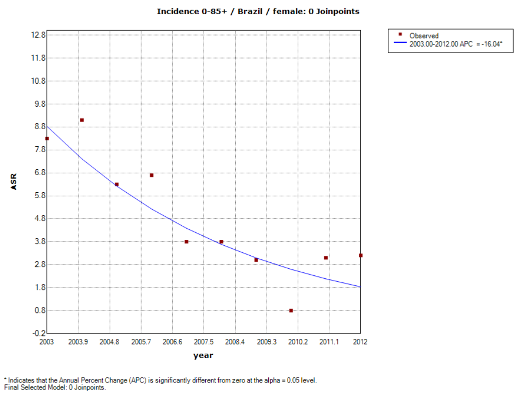

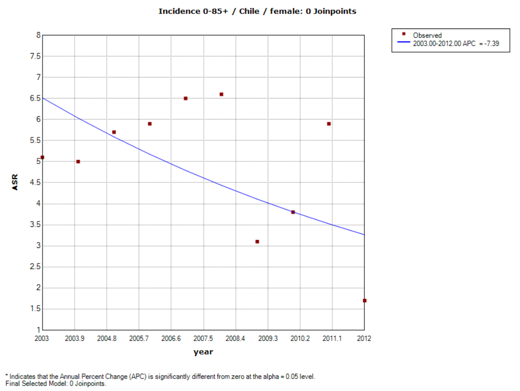

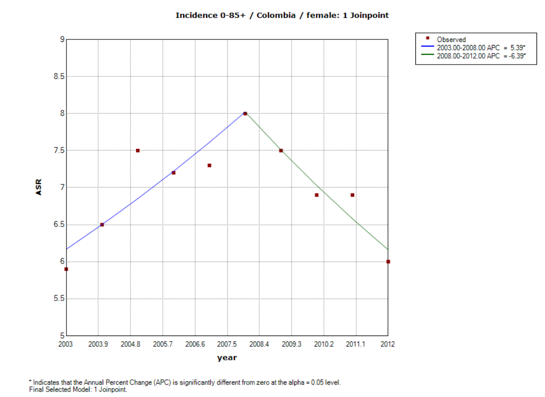

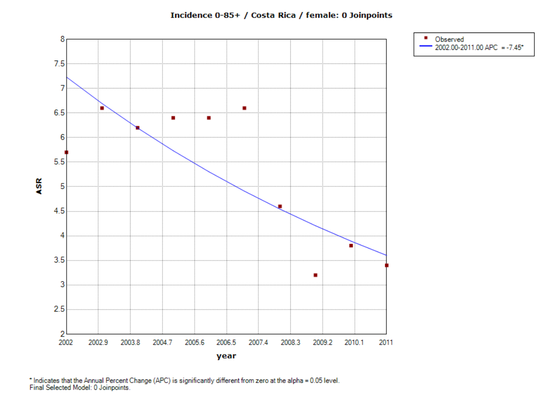

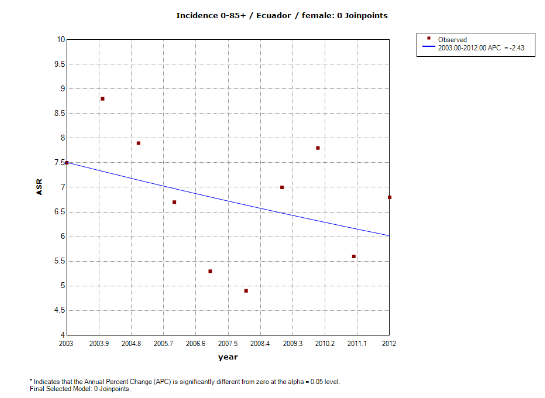

Northern Europe

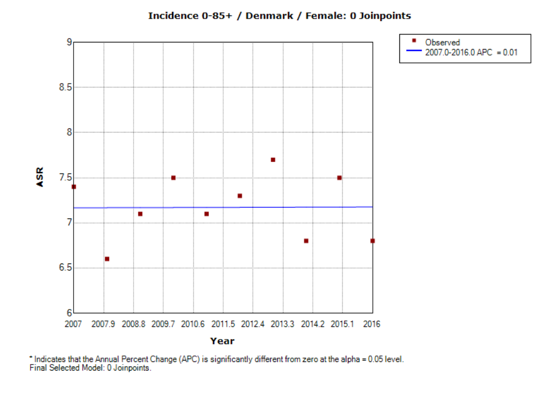

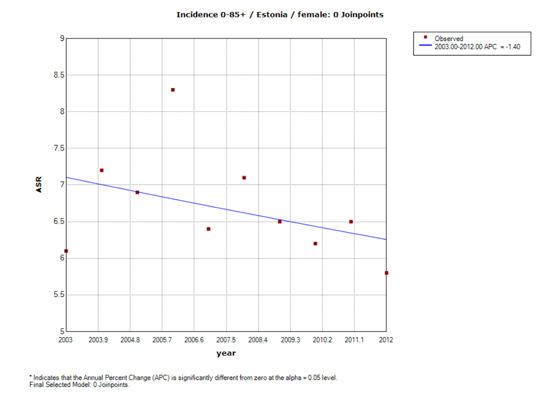

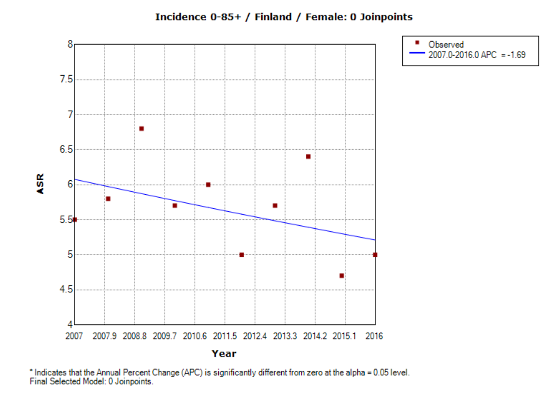

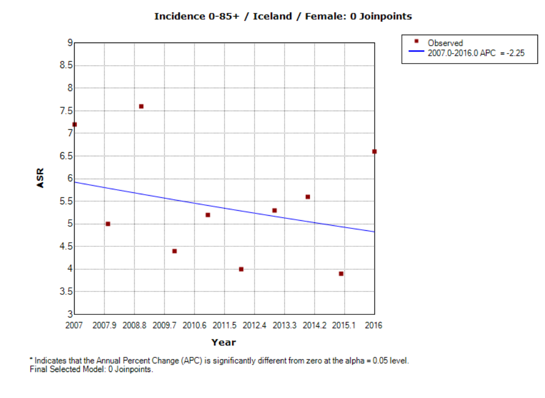

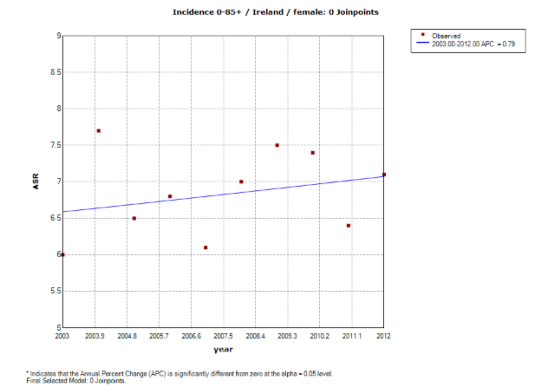

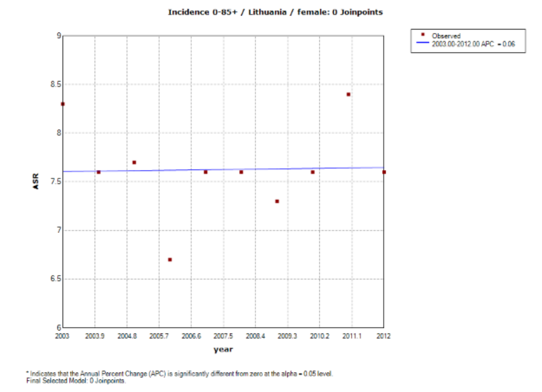

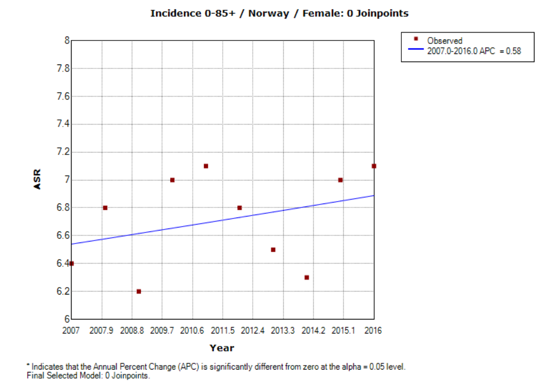

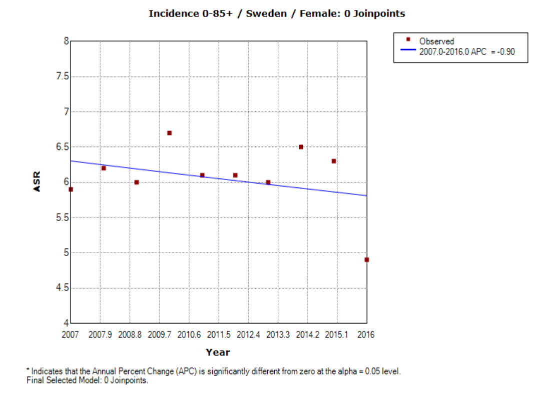

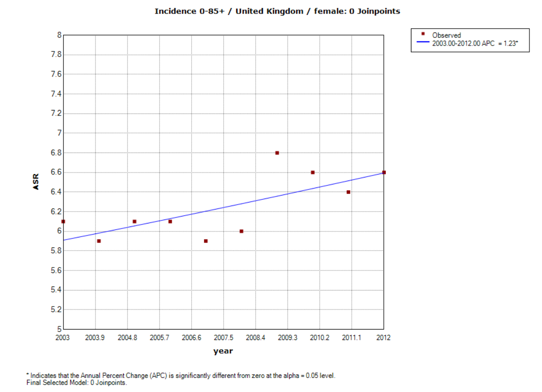

Western Europe

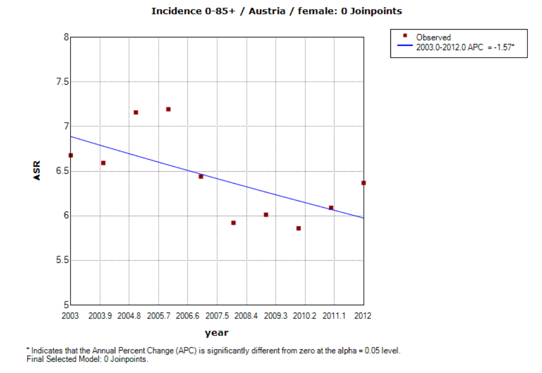

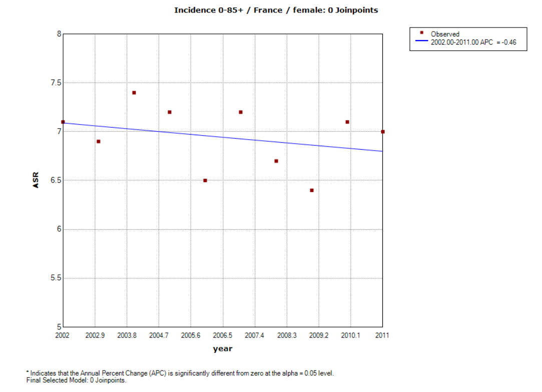

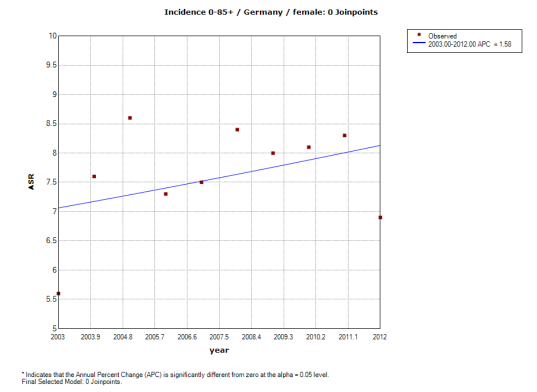

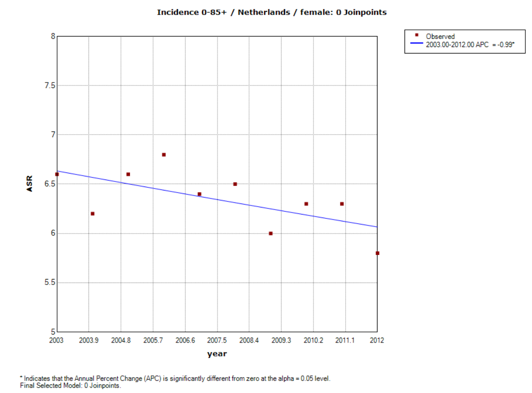

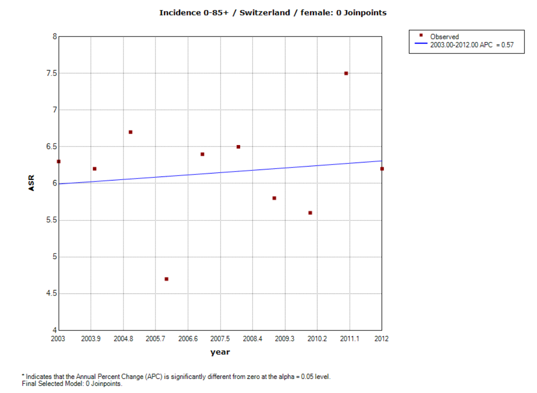

Southern Europe

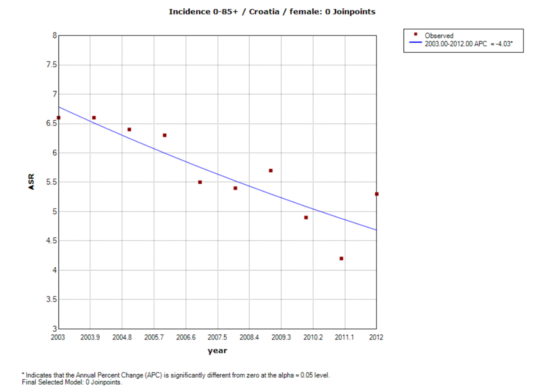

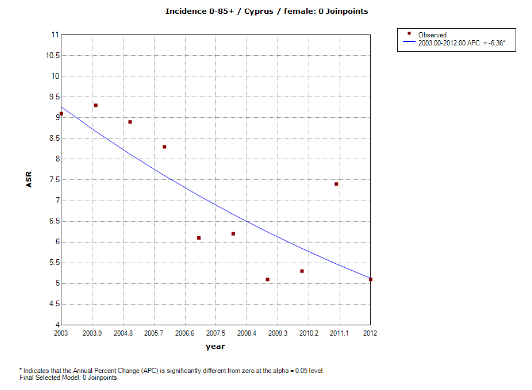

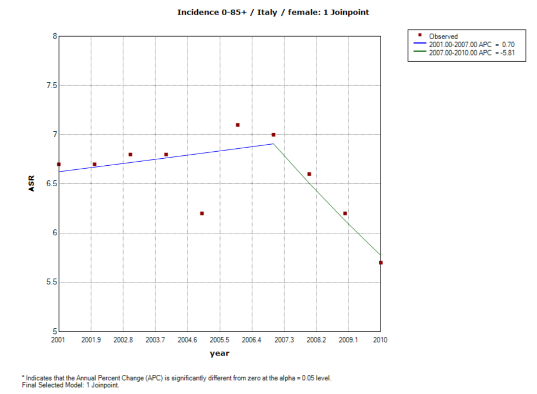

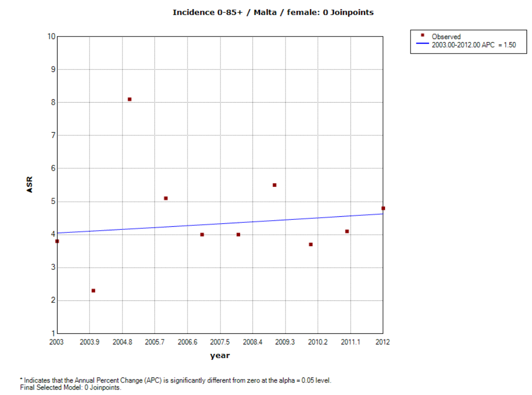

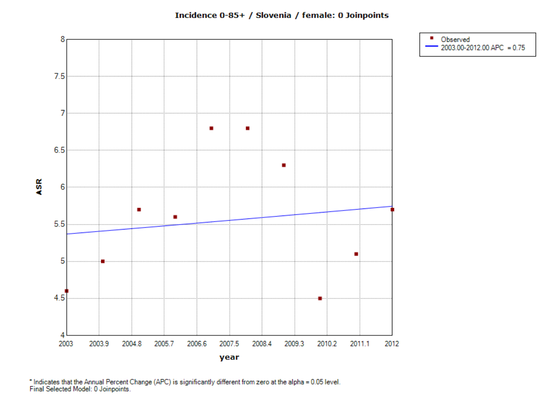

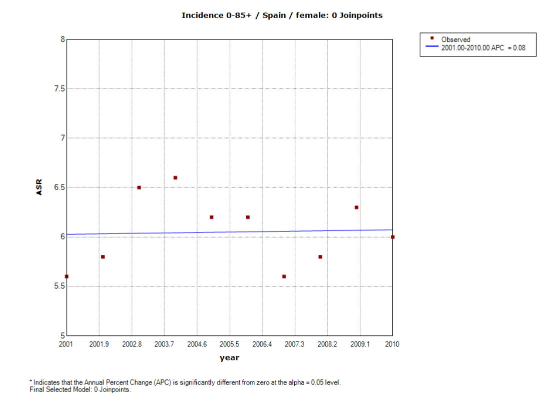

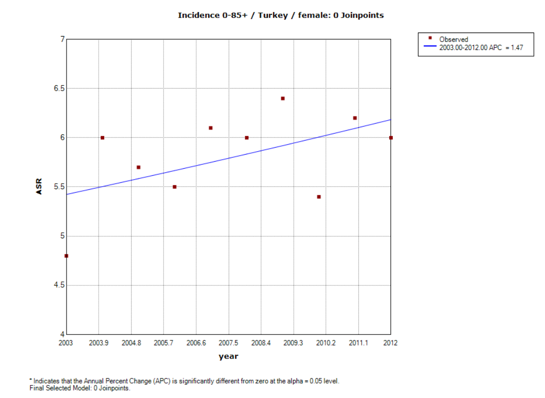

Eastern Europe

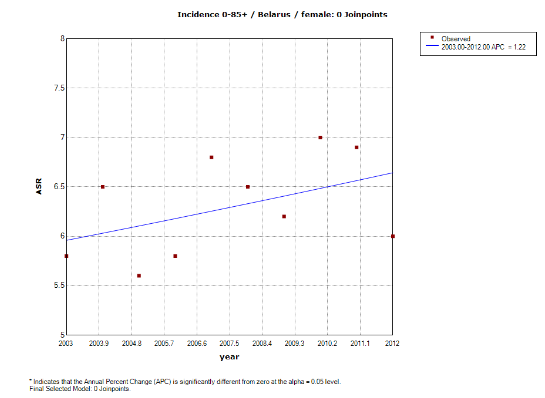

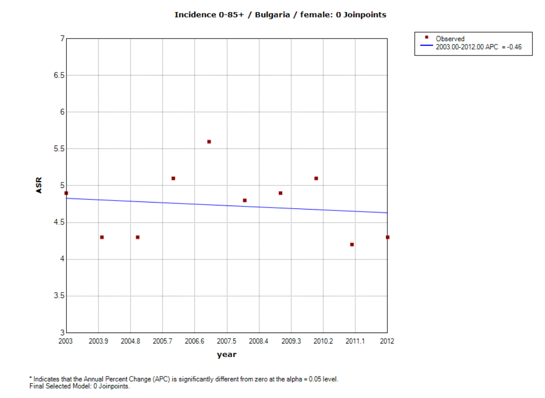

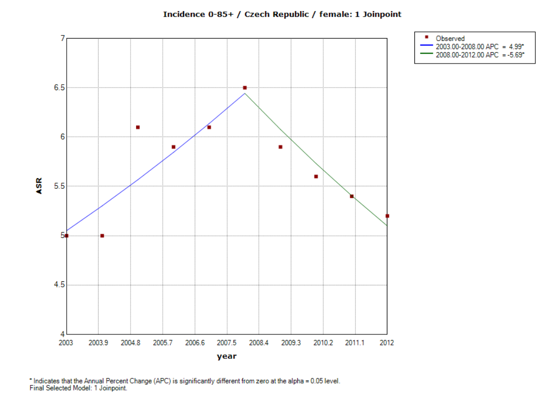

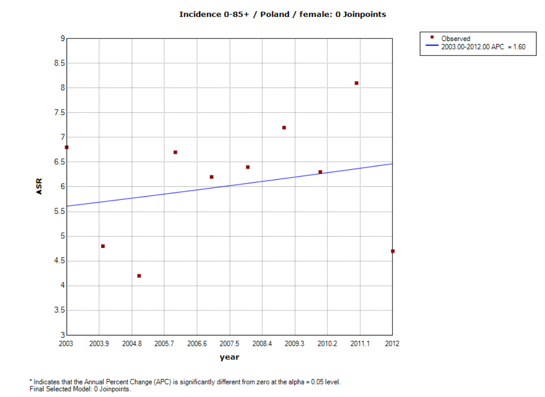

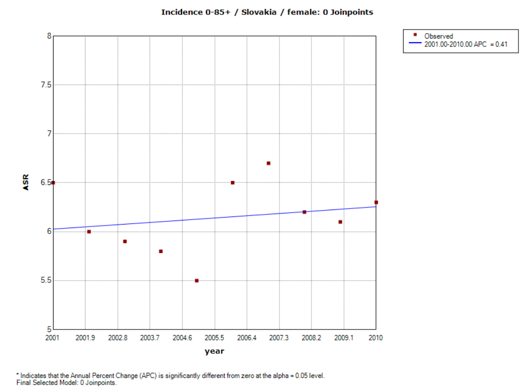
Incidence 0-85+ / Slovakia / female: 0 Joinpoints
Observed
2001.00-2010.00 APC = 0.41
ASR
year
* Indicates that the Annual Percent Change (APC) is significantly different from zero at the alpha = 0.05 level.
Final Selected Model: 0 Joinpoints.

Africa

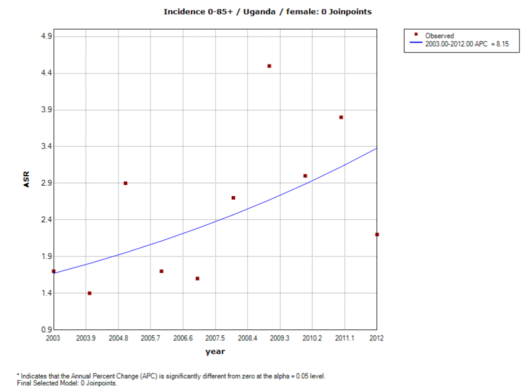
Incidence 0-85+ / Uganda / female: 0 Joinpoints
Observed
2003.00-2012.00 APC = 8.15
ASR
year
* Indicates that the Annual Percent Change (APC) is significantly different from zero at the alpha = 0.05 level.
Final Selected Model: 0 Joinpoints.

c.) Incidence male below 40 years old

Asia

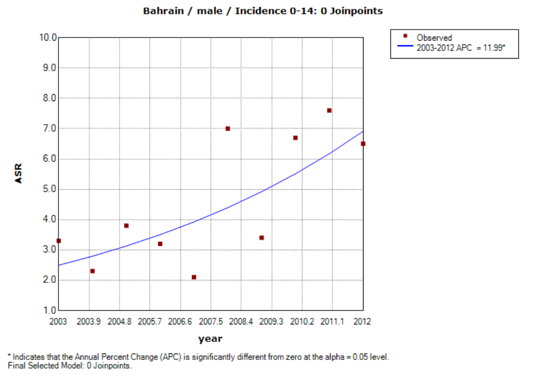

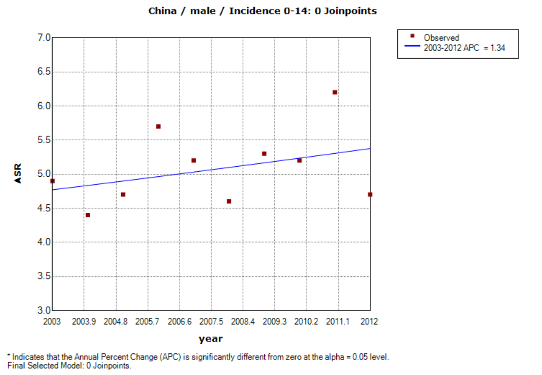

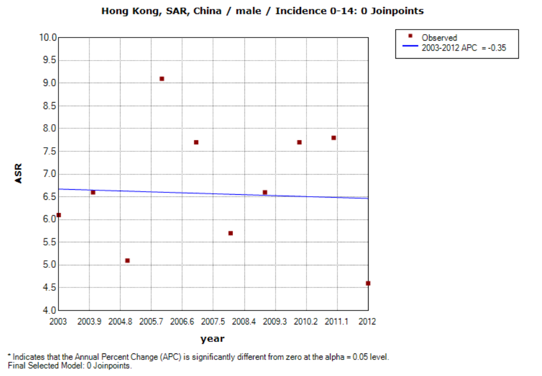

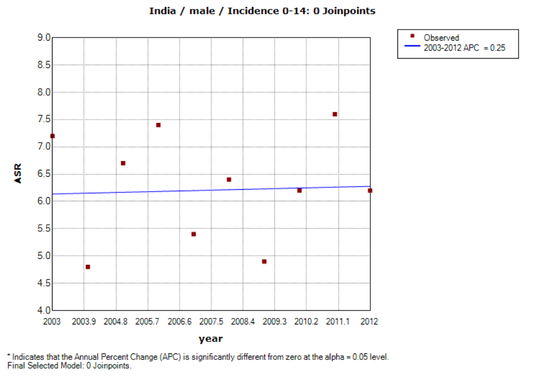

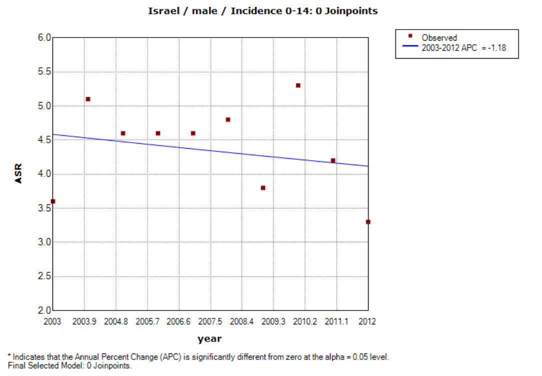

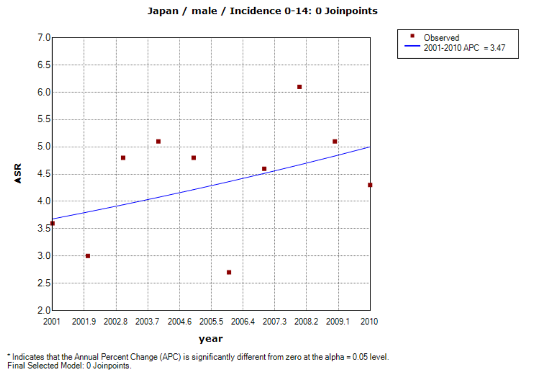

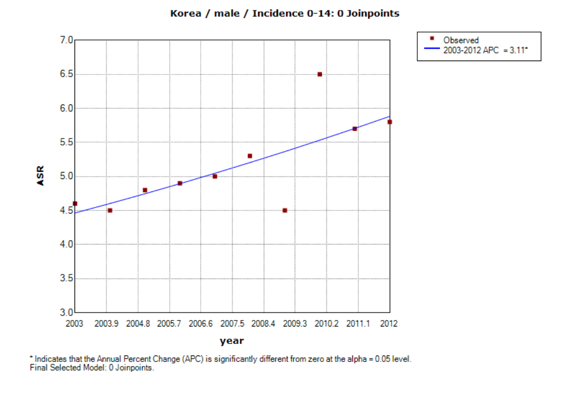
Korea / male / Incidence 0-14: 0 Joinpoints
Observed
2003-2012 APC = 3.11*
ASR
year
* Indicates that the Annual Percent Change (APC) is significantly different from zero at the alpha = 0.05 level.
Final Selected Model: 0 Joinpoints.

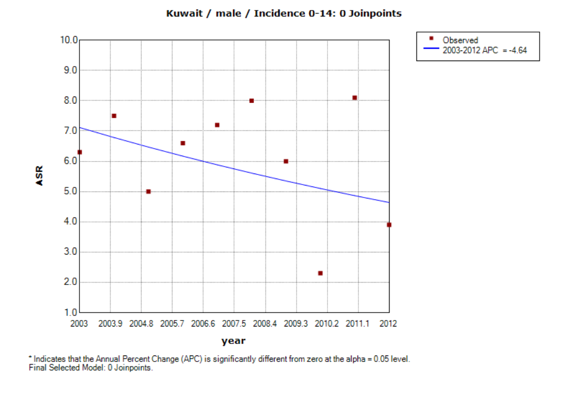
Kuwait / male / Incidence 0-14: 0 Joinpoints
Observed
2003-2012 APC = -4.64
ASR
year
* Indicates that the Annual Percent Change (APC) is significantly different from zero at the alpha = 0.05 level.
Final Selected Model: 0 Joinpoints.

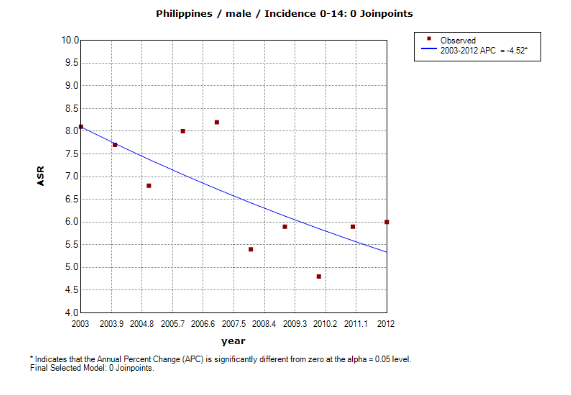
Philippines / male / Incidence 0-14: 0 Joinpoints
Observed
2003-2012 APC = -4.52*
ASR
year
* Indicates that the Annual Percent Change (APC) is significantly different from zero at the alpha = 0.05 level.
Final Selected Model: 0 Joinpoints.

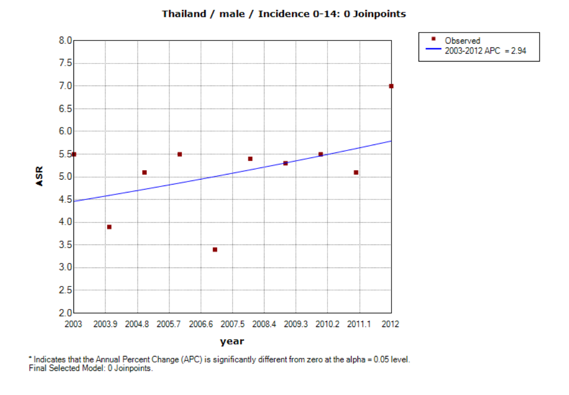
Thailand / male / Incidence 0-14: 0 Joinpoints
Observed
2003-2012 APC = 2.94
ASR
year
* Indicates that the Annual Percent Change (APC) is significantly different from zero at the alpha = 0.05 level.
Final Selected Model: 0 Joinpoints.

Oceania

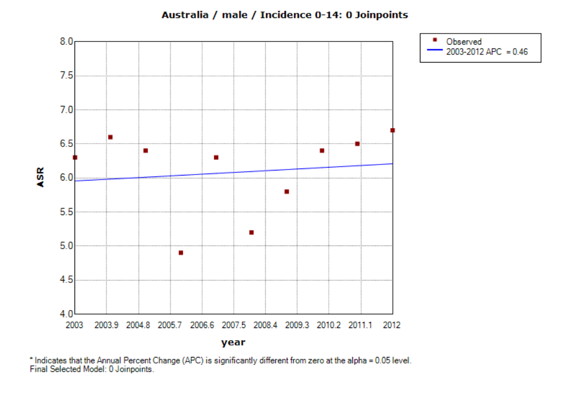
Australia / male / Incidence 0-14: 0 Joinpoints
Observed
2003-2012 APC = 0.46
ASR
year
* Indicates that the Annual Percent Change (APC) is significantly different from zero at the alpha = 0.05 level.
Final Selected Model: 0 Joinpoints.

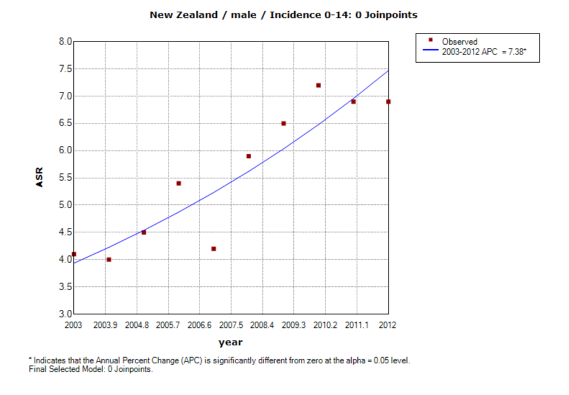
New Zealand / male / Incidence 0-14: 0 Joinpoints
Observed
2003-2012 APC = 7.38*
ASR
year
* Indicates that the Annual Percent Change (APC) is significantly different from zero at the alpha = 0.05 level.
Final Selected Model: 0 Joinpoints.

Northern America

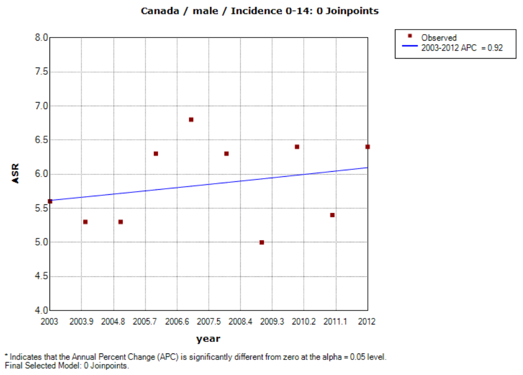

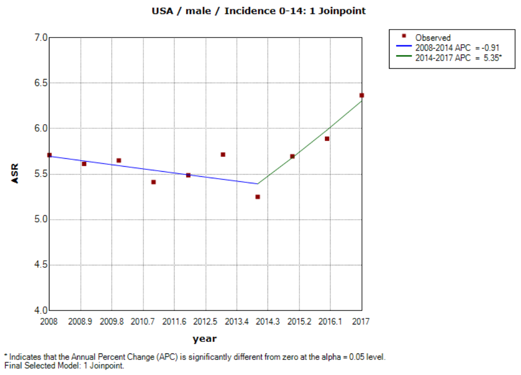

Southern America

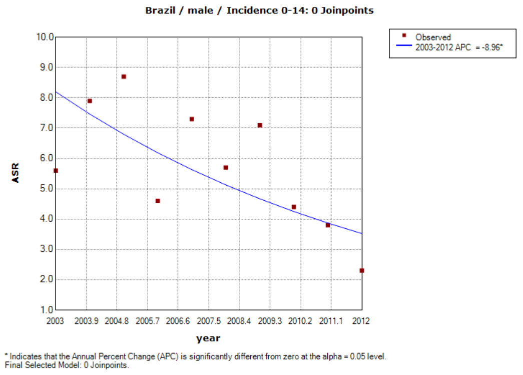

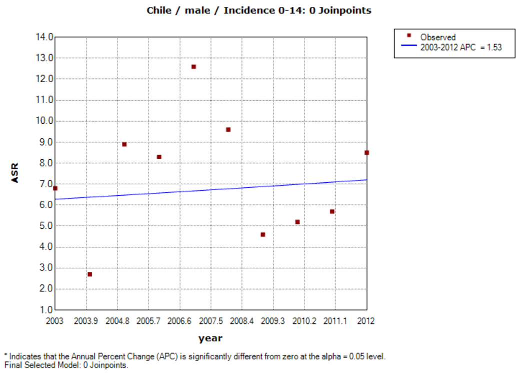

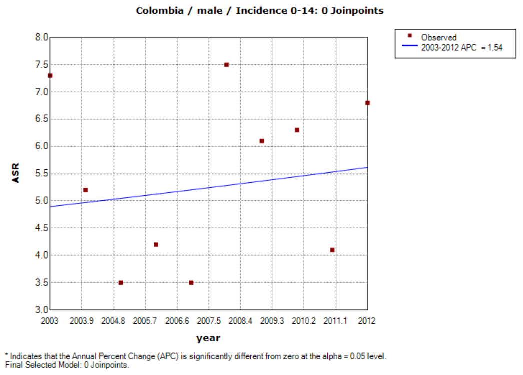

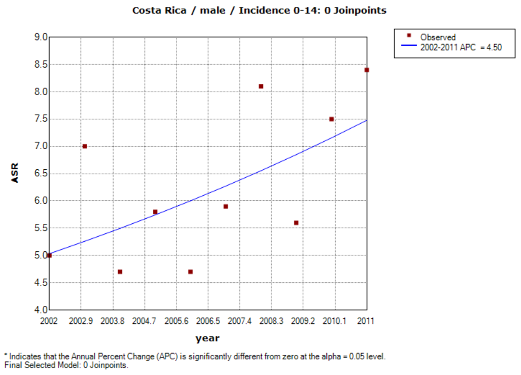

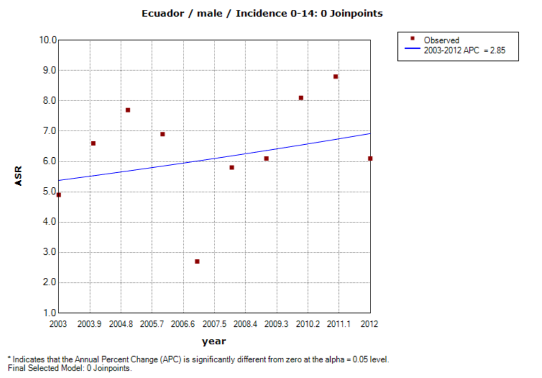

Northern Europe

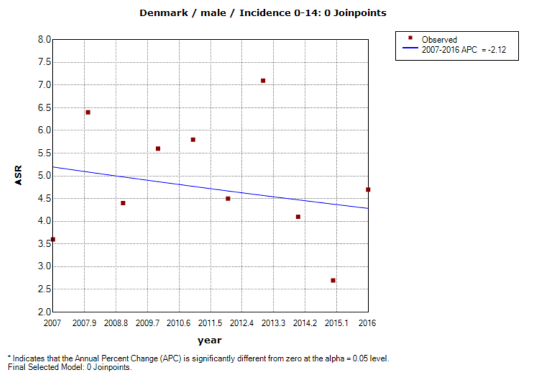

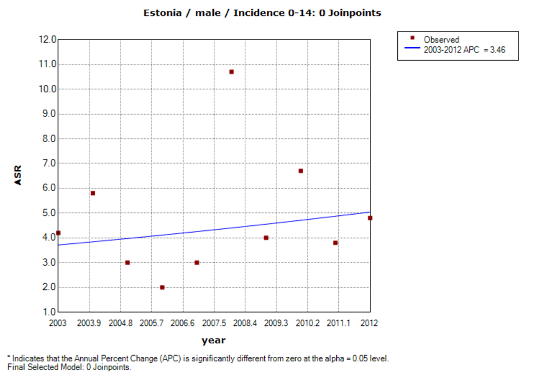

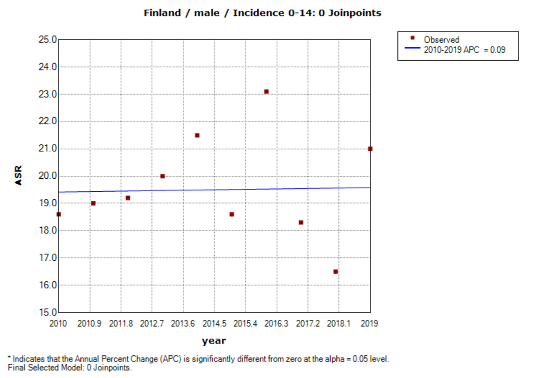

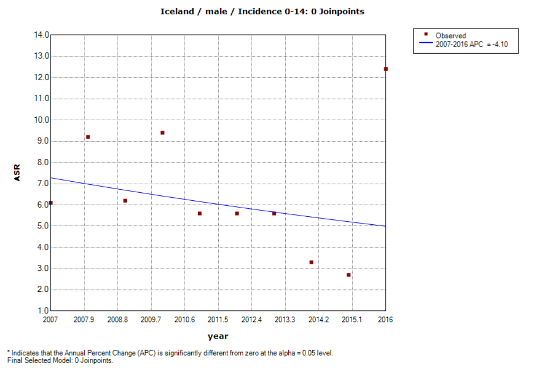

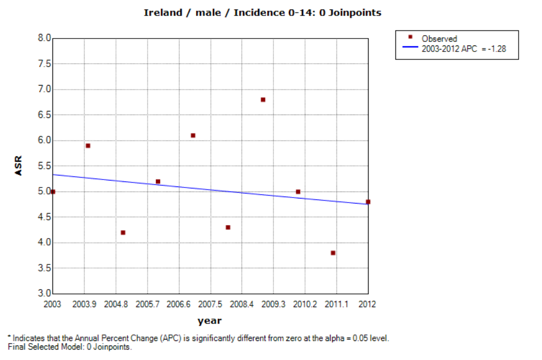

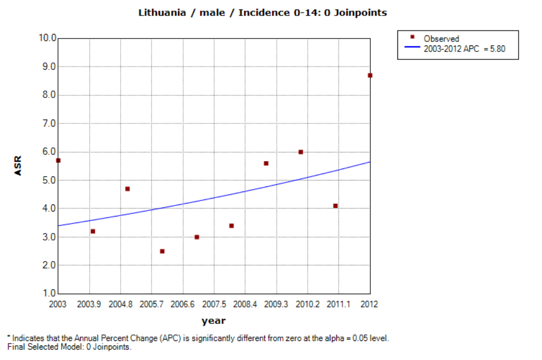

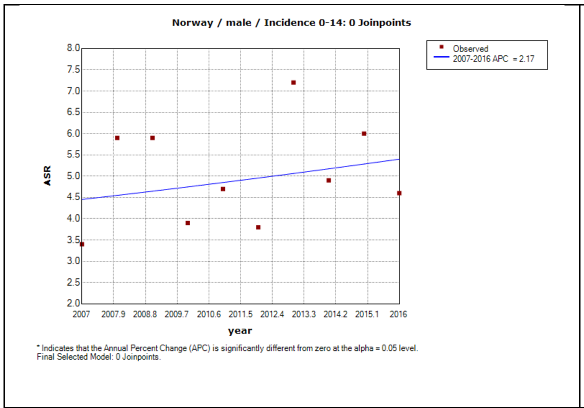

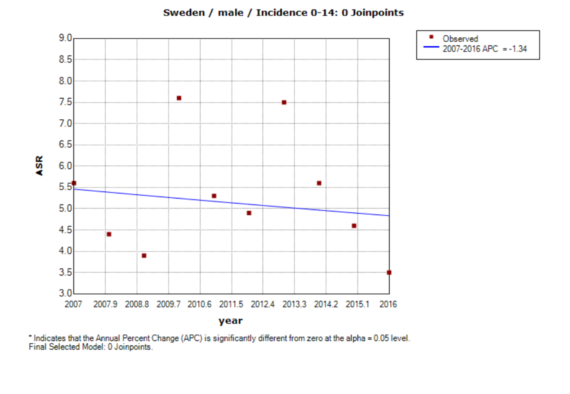

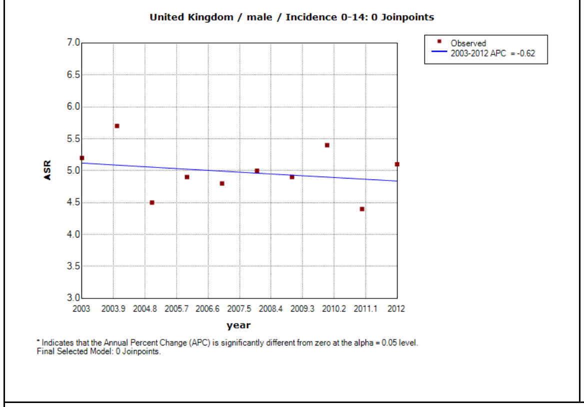

Western Europe

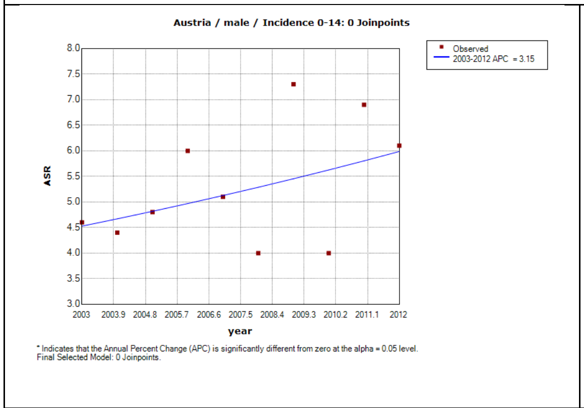

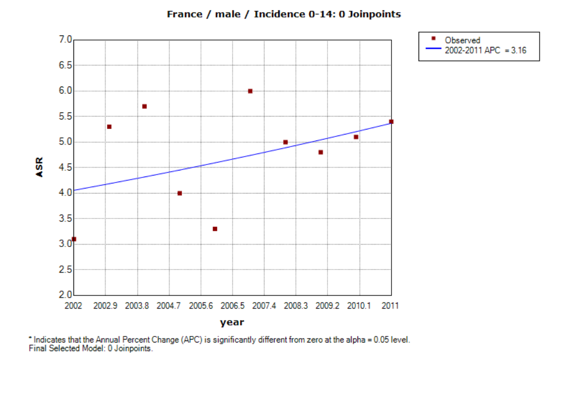

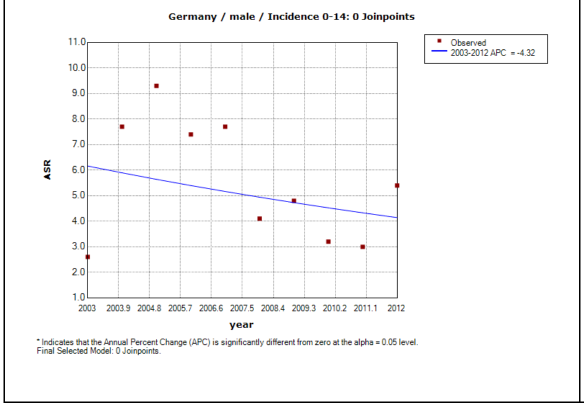

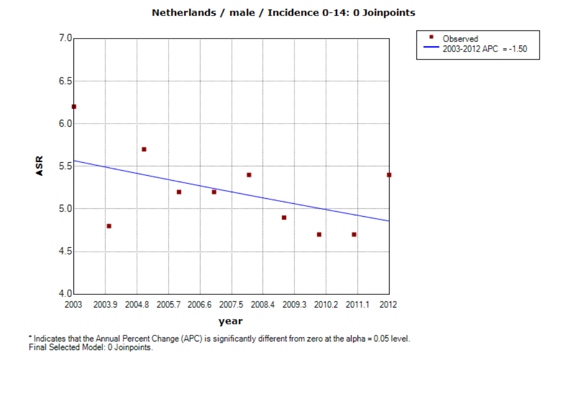

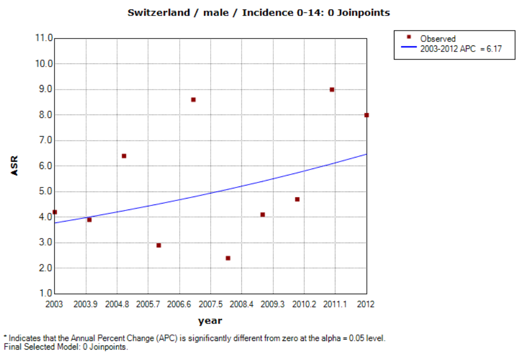

Southern Europe

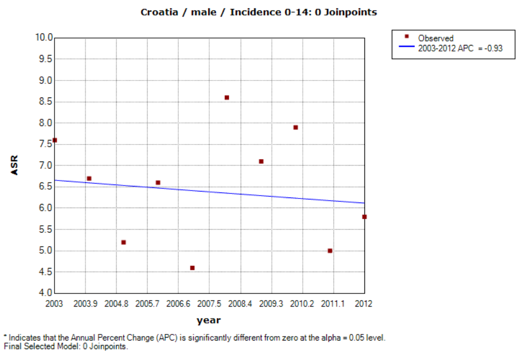

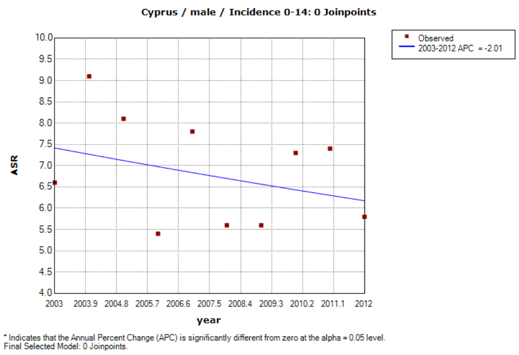

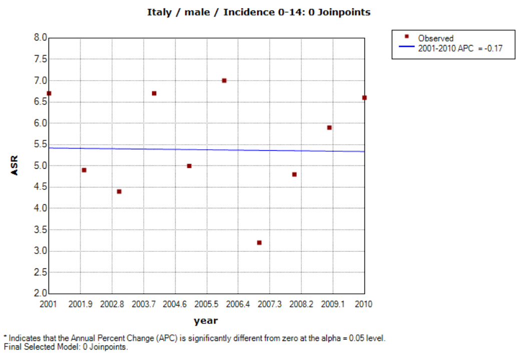

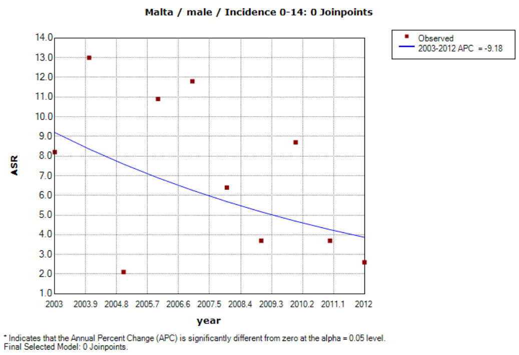

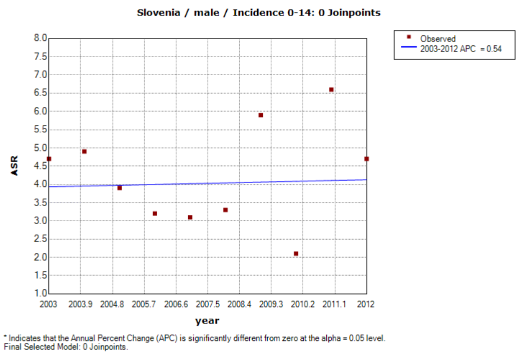

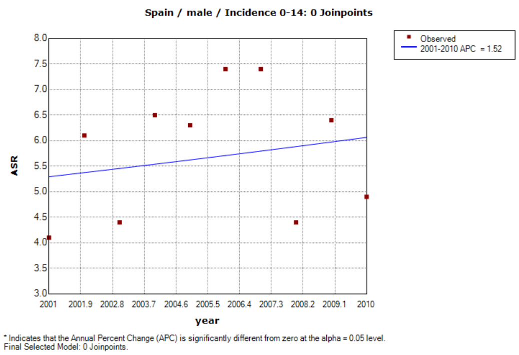

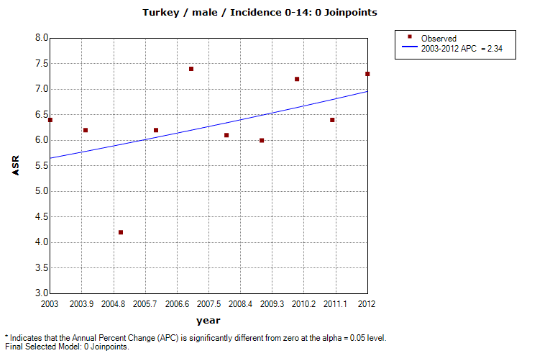

Eastern Europe

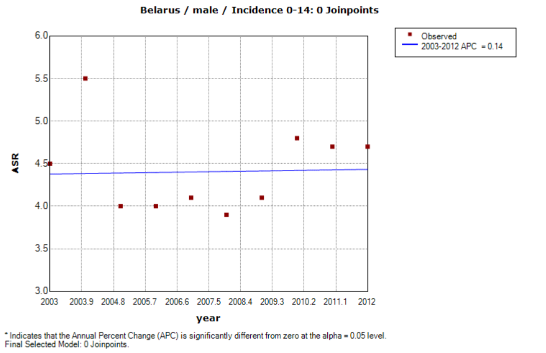

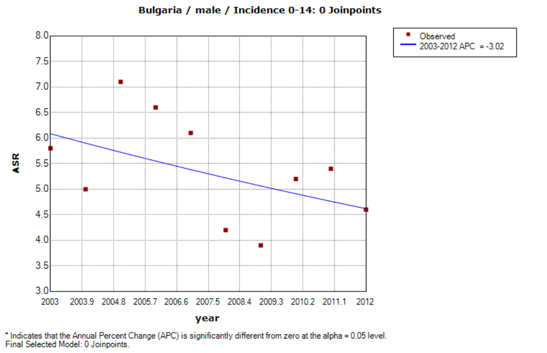

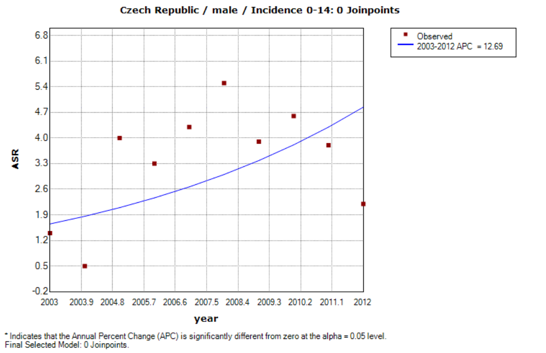

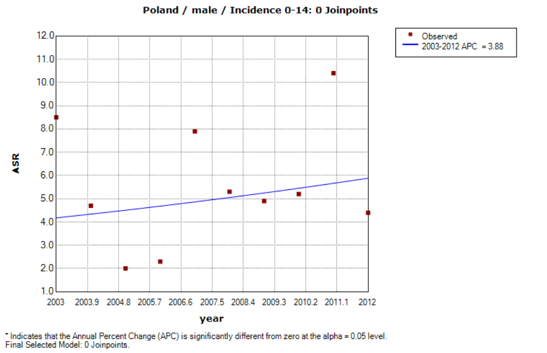

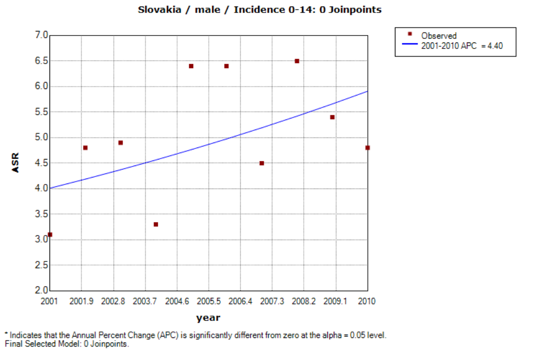

Africa	
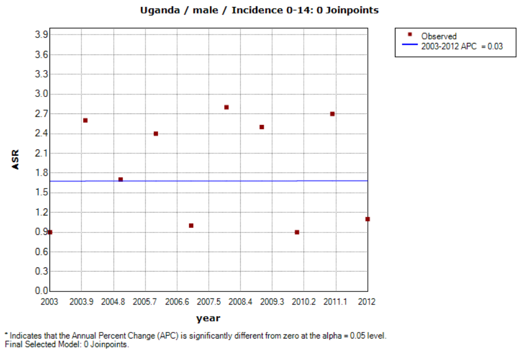Uganda / male / Incidence 0-14: 0 Joinpoints
Observed
2003-2012 APC = 0.03
ASR
year
3.9
3.6
3.3
3.0
2.7
2.4
2.1
1.8
1.5
1.2
0.9
0.6
0.3
0.0
2003
2003.9
2004.8
2005.7
2006.6
2007.5
2008.4
2009.3
2010.2
2011.1
2012
* Indicates that the Annual Percent Change (APC) is significantly different from zero at the alpha = 0.05 level.
Final Selected Model: 0 Joinpoints.	

d.) Incidence female below 15 years old

Asia

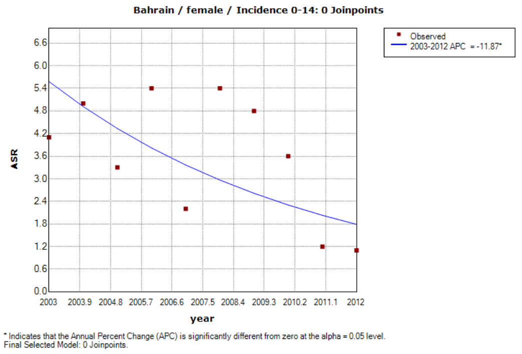

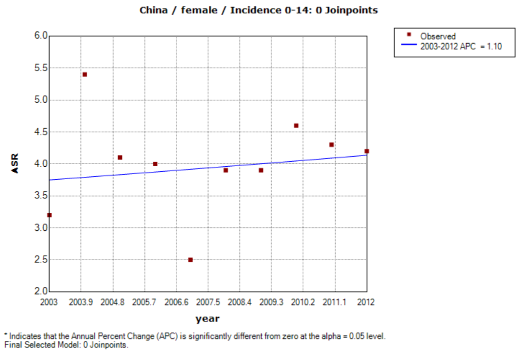

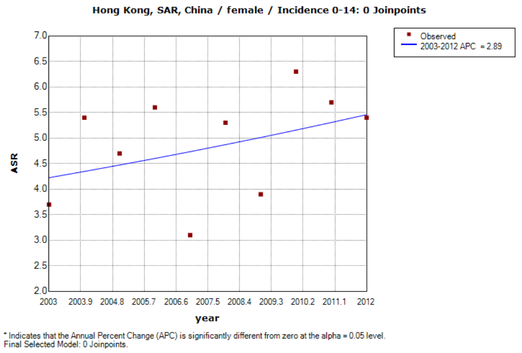

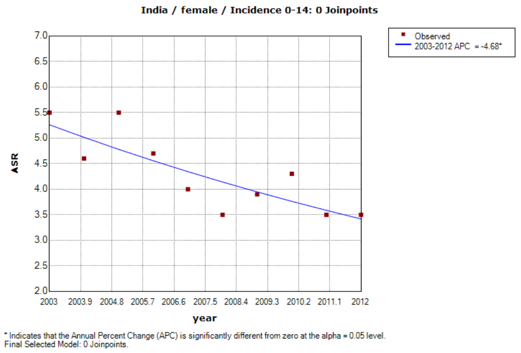

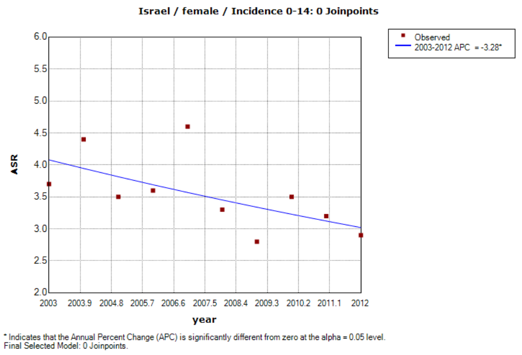

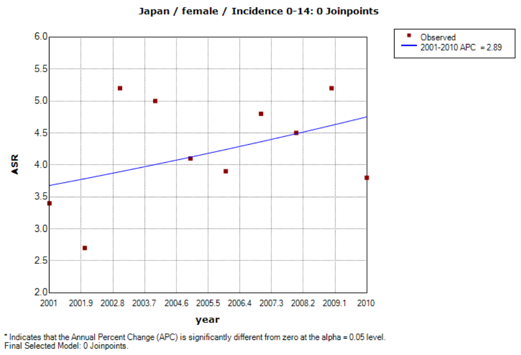

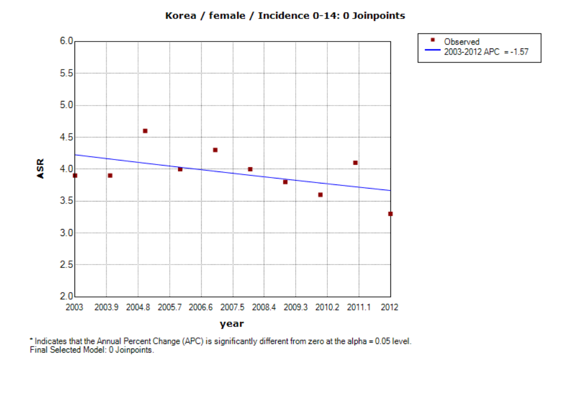
Korea / female / Incidence 0-14: 0 Joinpoints
Observed
2003-2012 APC = -1.57
ASR
year
* Indicates that the Annual Percent Change (APC) is significantly different from zero at the alpha = 0.05 level.
Final Selected Model: 0 Joinpoints.

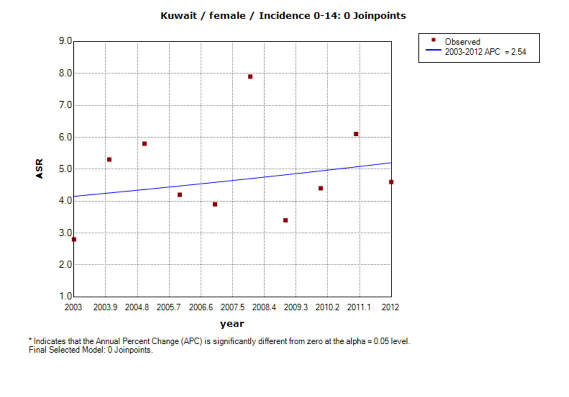
Kuwait / female / Incidence 0-14: 0 Joinpoints
Observed
2003-2012 APC = 2.54
ASR
year
* Indicates that the Annual Percent Change (APC) is significantly different from zero at the alpha = 0.05 level.
Final Selected Model: 0 Joinpoints.

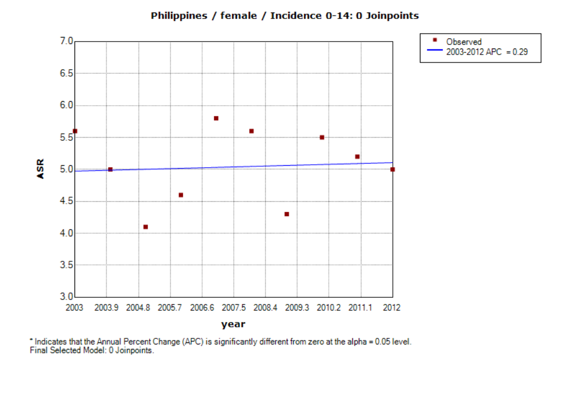
Philippines / female / Incidence 0-14: 0 Joinpoints
Observed
2003-2012 APC = 0.29
ASR
year
* Indicates that the Annual Percent Change (APC) is significantly different from zero at the alpha = 0.05 level.
Final Selected Model: 0 Joinpoints.

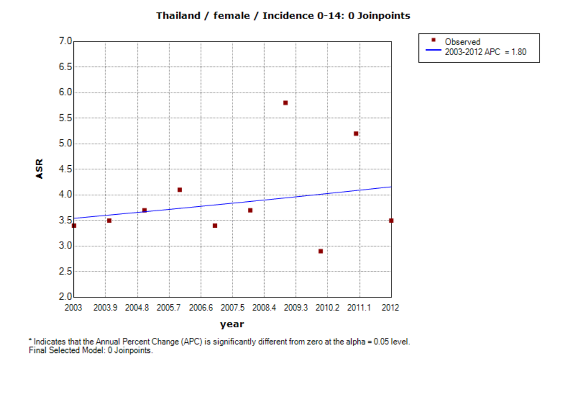
Thailand / female / Incidence 0-14: 0 Joinpoints
Observed
2003-2012 APC = 1.80
ASR
year
* Indicates that the Annual Percent Change (APC) is significantly different from zero at the alpha = 0.05 level.
Final Selected Model: 0 Joinpoints.

Oceania

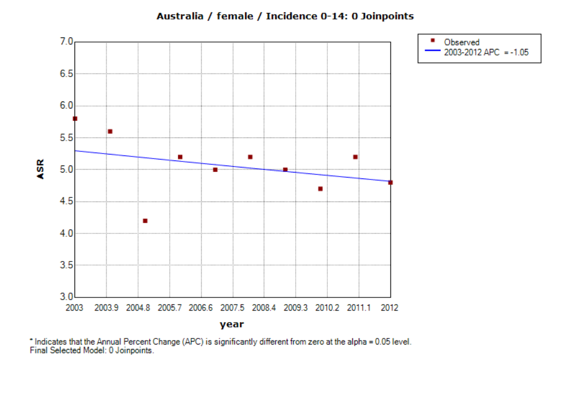
Australia / female / Incidence 0-14: 0 Joinpoints
Observed
2003-2012 APC = -1.05
ASR
year
* Indicates that the Annual Percent Change (APC) is significantly different from zero at the alpha = 0.05 level.
Final Selected Model: 0 Joinpoints.

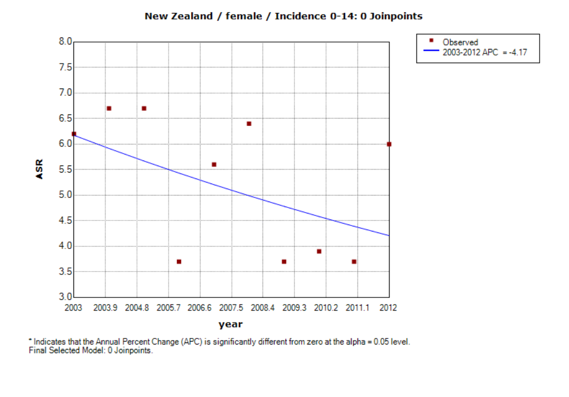
New Zealand / female / Incidence 0-14: 0 Joinpoints
Observed
2003-2012 APC = -4.17
ASR
year
* Indicates that the Annual Percent Change (APC) is significantly different from zero at the alpha = 0.05 level.
Final Selected Model: 0 Joinpoints.

Northern America

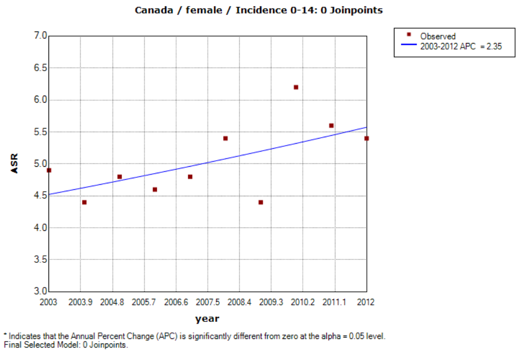

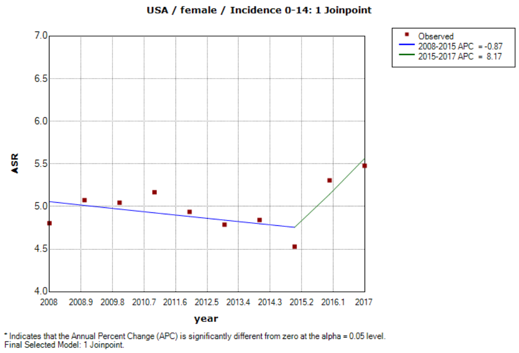

Southern America

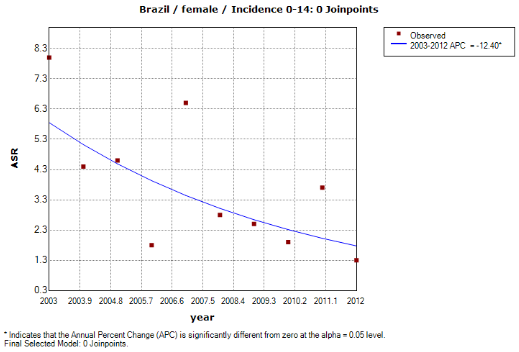

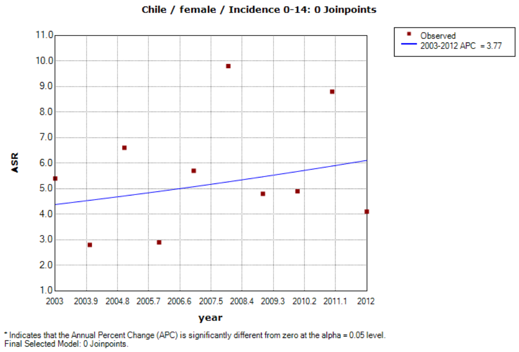

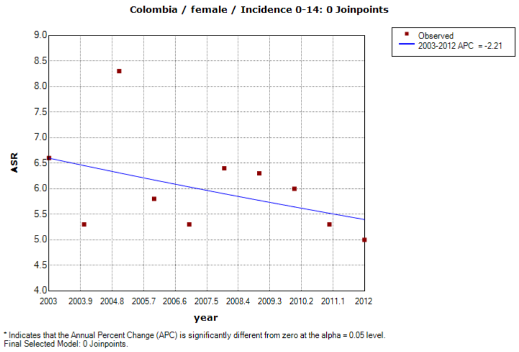

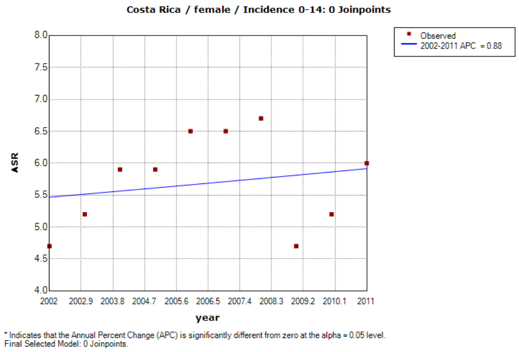

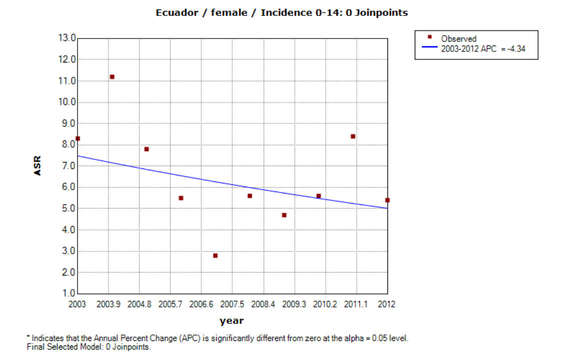

Northern Europe

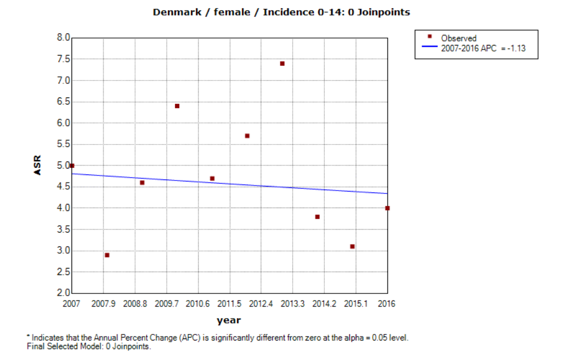

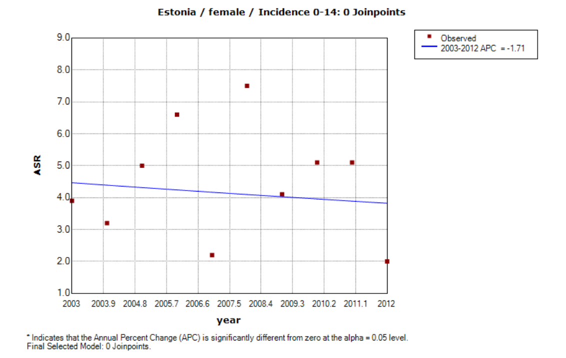

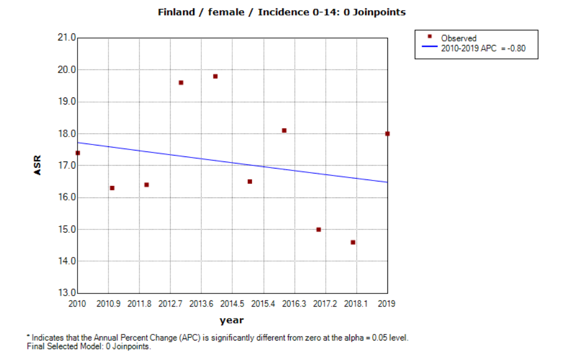

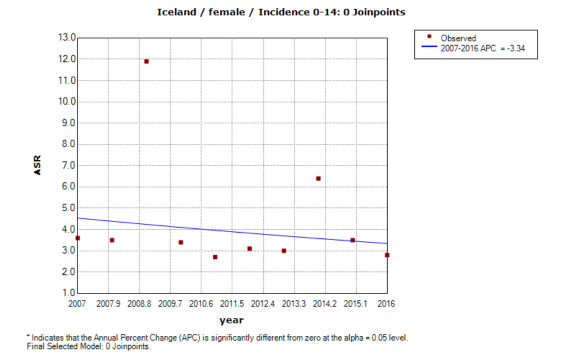

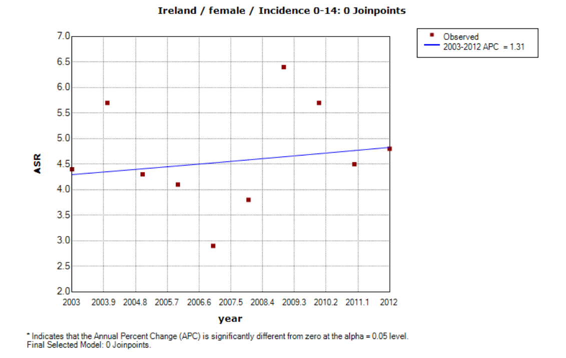

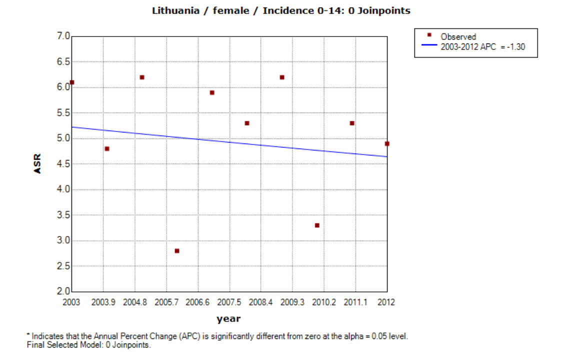

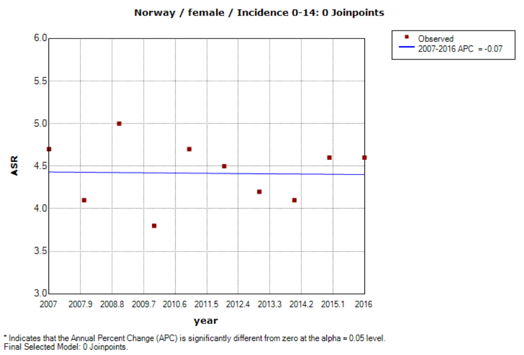

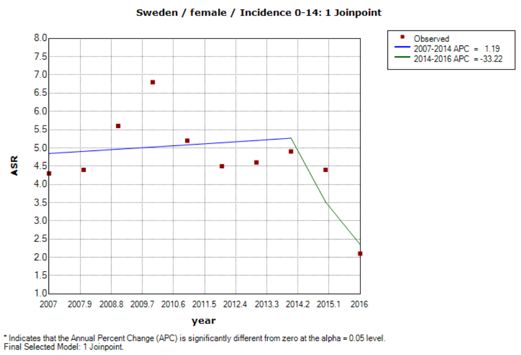

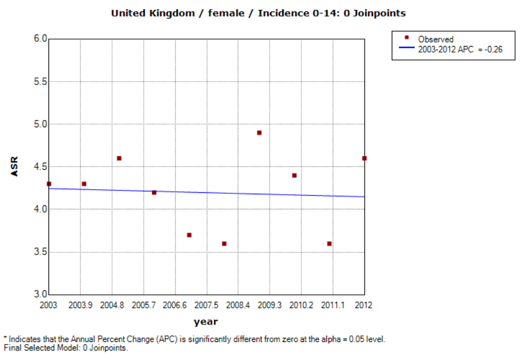

Western Europe

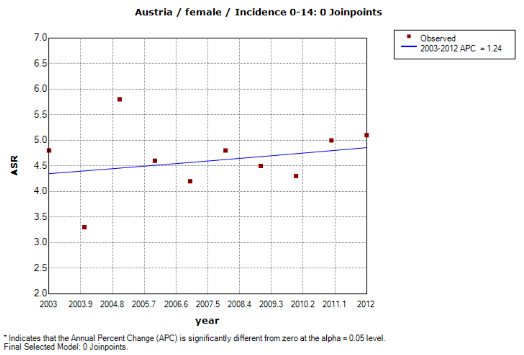

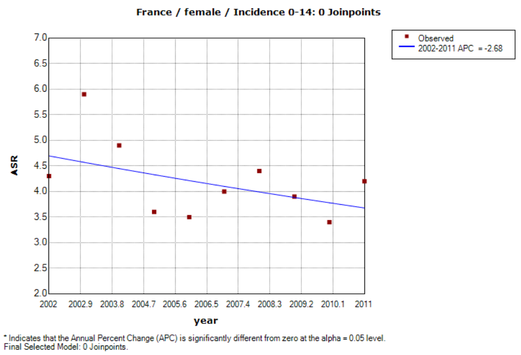

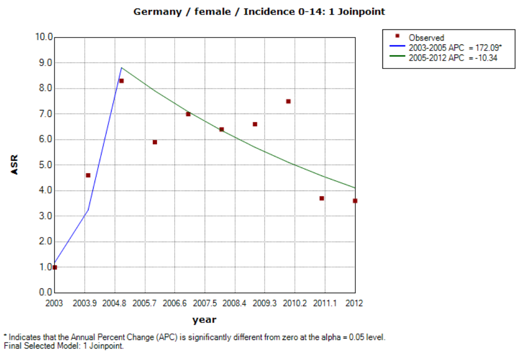

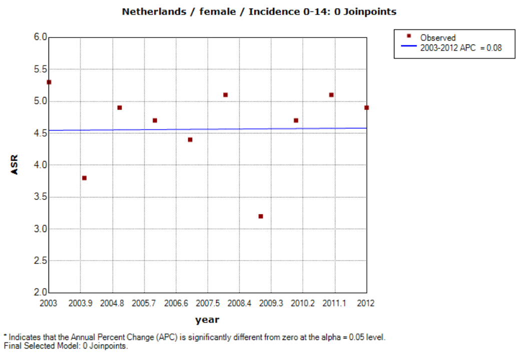

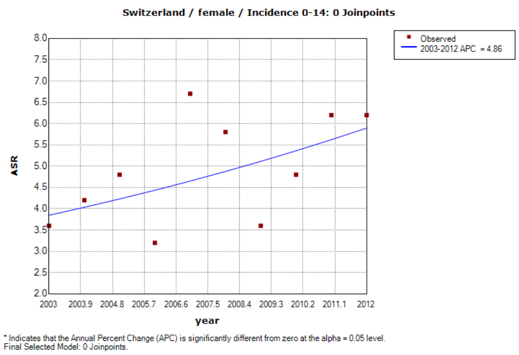

Southern Europe

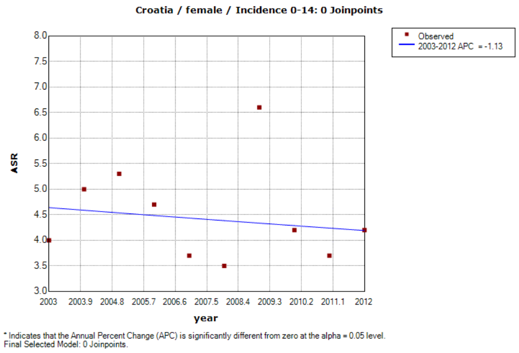

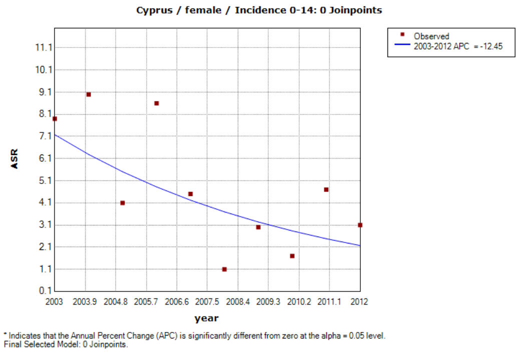

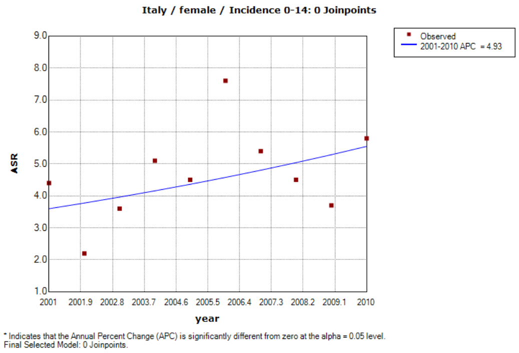

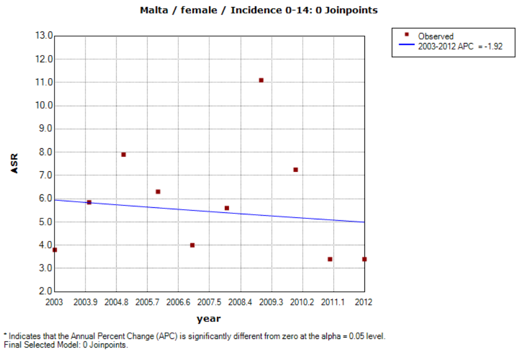

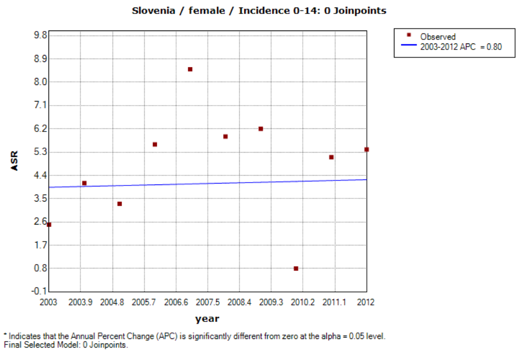

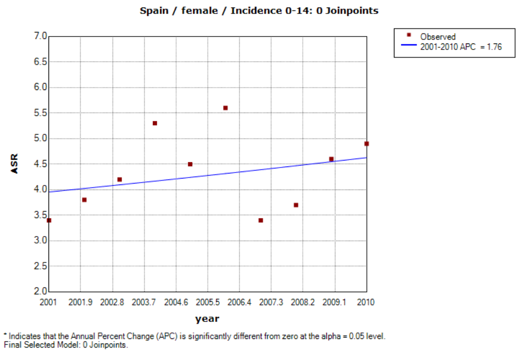

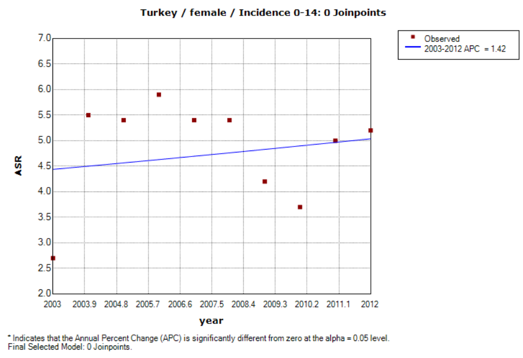

Eastern Europe

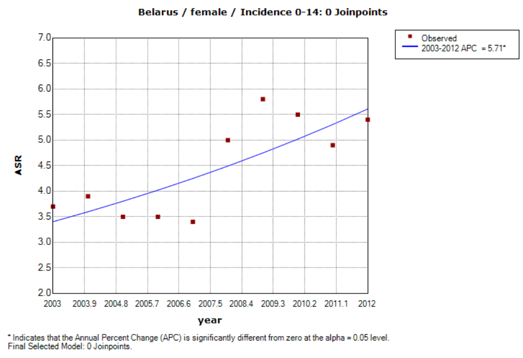

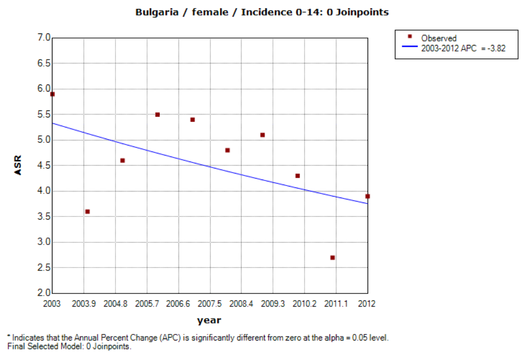

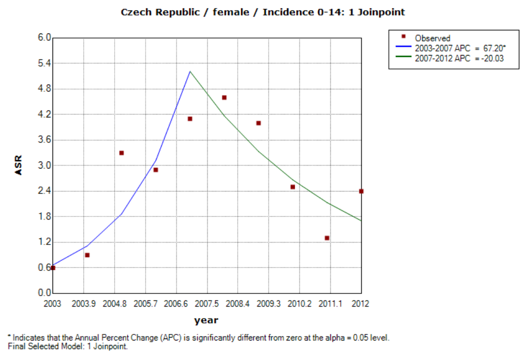

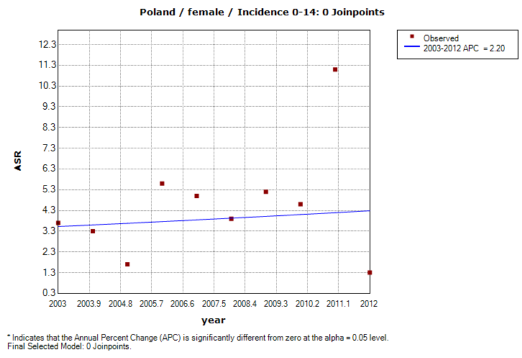

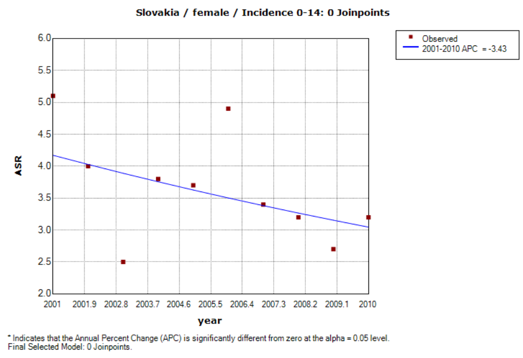

Africa

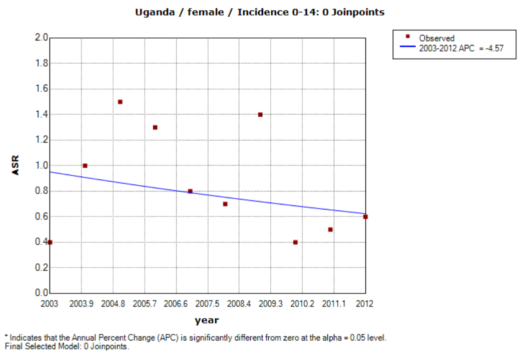
Uganda / female / Incidence 0-14: 0 Joinpoints
Observed
2003-2012 APC = -4.57
ASR
year
2.0
1.8
1.6
1.4
1.2
1.0
0.8
0.6
0.4
0.2
0.0
2003
2003.9
2004.8
2005.7
2006.6
2007.5
2008.4
2009.3
2010.2
2011.1
2012
* Indicates that the Annual Percent Change (APC) is significantly different from zero at the alpha = 0.05 level.
Final Selected Model: 0 Joinpoints.

e.) Incidence male between 15-49 years old

Asia

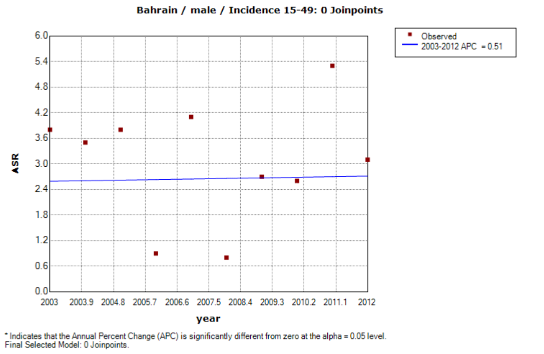

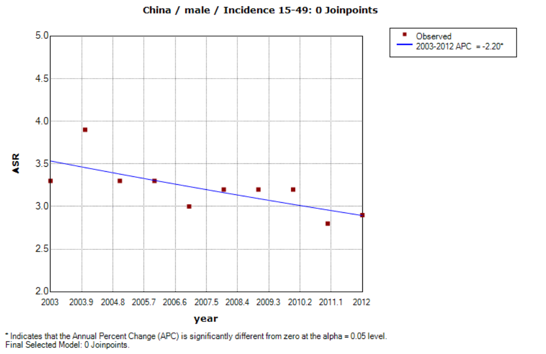

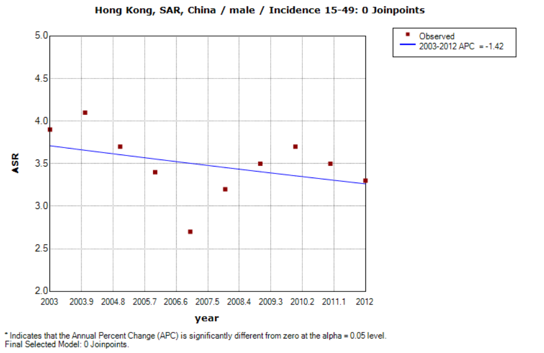

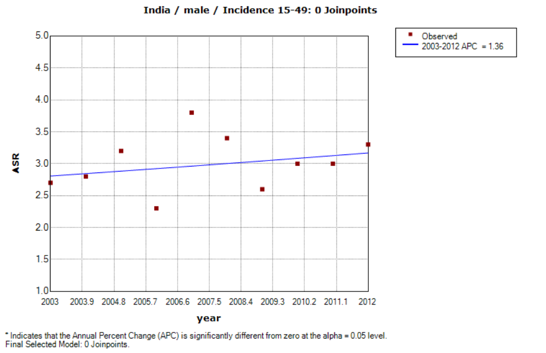

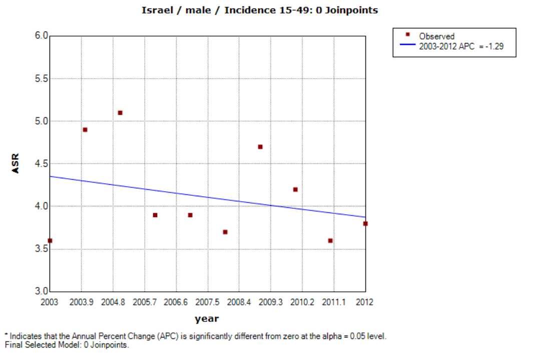

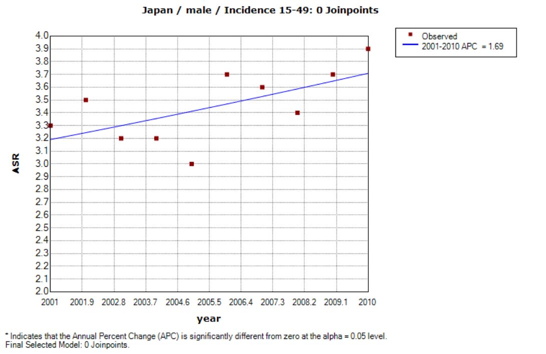

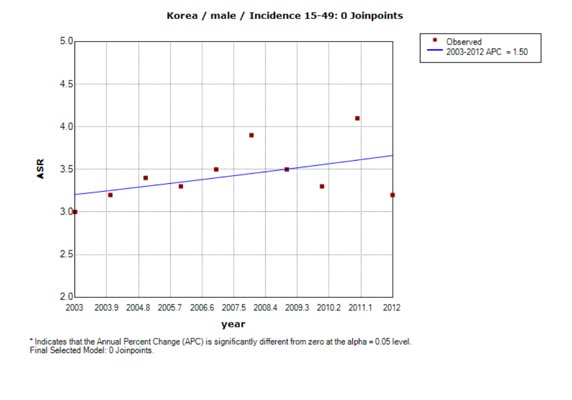
Korea / male / Incidence 15-49: 0 Joinpoints
Observed
2003-2012 APC = 1.50
ASR
year
* Indicates that the Annual Percent Change (APC) is significantly different from zero at the alpha = 0.05 level.
Final Selected Model: 0 Joinpoints.

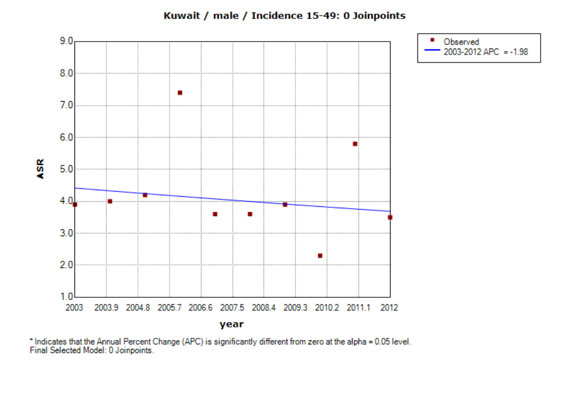
Kuwait / male / Incidence 15-49: 0 Joinpoints
Observed
2003-2012 APC = -1.98
ASR
year
* Indicates that the Annual Percent Change (APC) is significantly different from zero at the alpha = 0.05 level.
Final Selected Model: 0 Joinpoints.

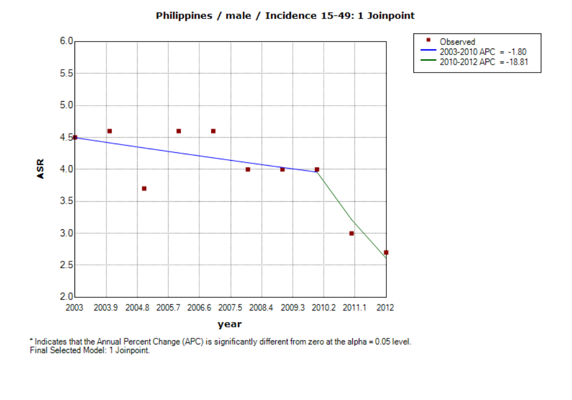
Philippines / male / Incidence 15-49: 1 Joinpoint
Observed
2003-2010 APC = -1.80
2010-2012 APC = -18.81
ASR
year
* Indicates that the Annual Percent Change (APC) is significantly different from zero at the alpha = 0.05 level.
Final Selected Model: 1 Joinpoint.

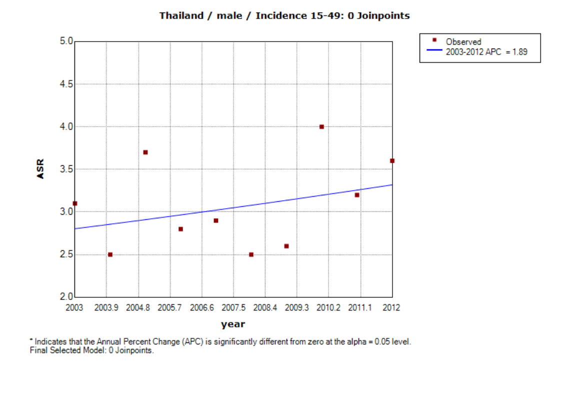
Thailand / male / Incidence 15-49: 0 Joinpoints
Observed
2003-2012 APC = 1.89
ASR
year
* Indicates that the Annual Percent Change (APC) is significantly different from zero at the alpha = 0.05 level.
Final Selected Model: 0 Joinpoints.

Oceania

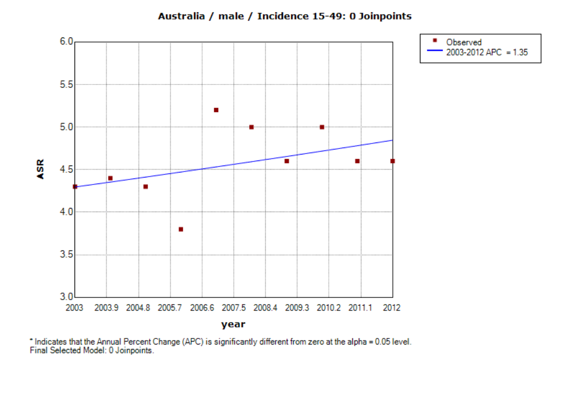
Australia / male / Incidence 15-49: 0 Joinpoints
Observed
2003-2012 APC = 1.35
ASR
year
* Indicates that the Annual Percent Change (APC) is significantly different from zero at the alpha = 0.05 level.
Final Selected Model: 0 Joinpoints.

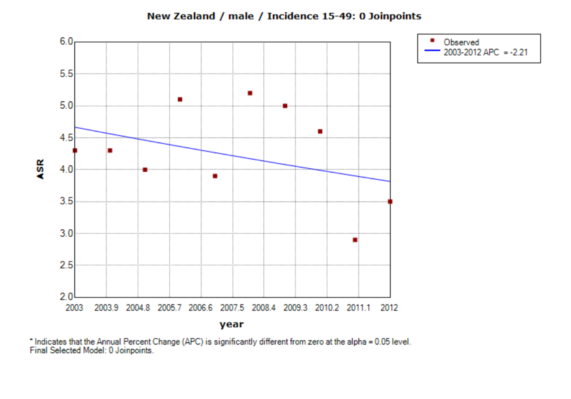
New Zealand / male / Incidence 15-49: 0 Joinpoints
Observed
2003-2012 APC = -2.21
ASR
year
* Indicates that the Annual Percent Change (APC) is significantly different from zero at the alpha = 0.05 level.
Final Selected Model: 0 Joinpoints.

Northern America

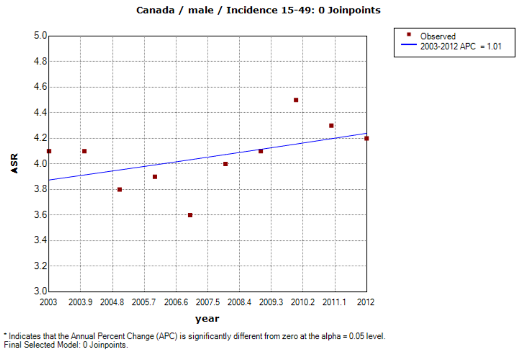

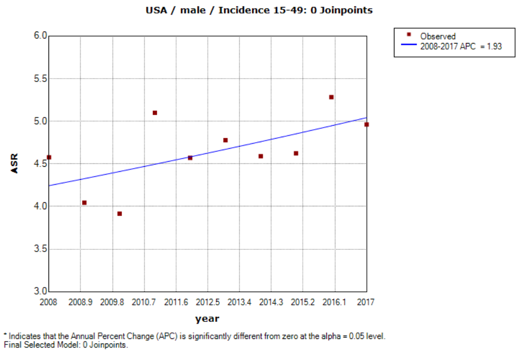

Southern America

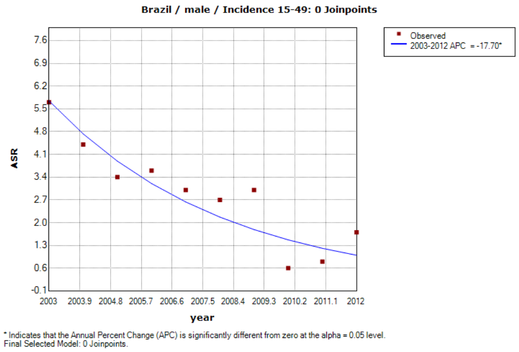

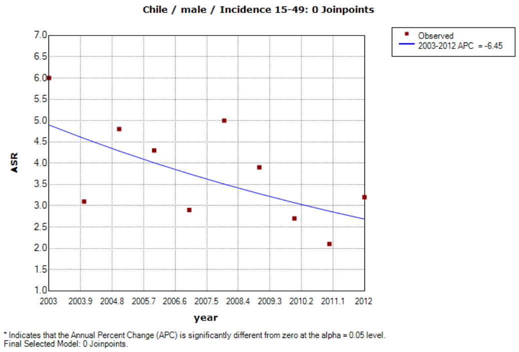

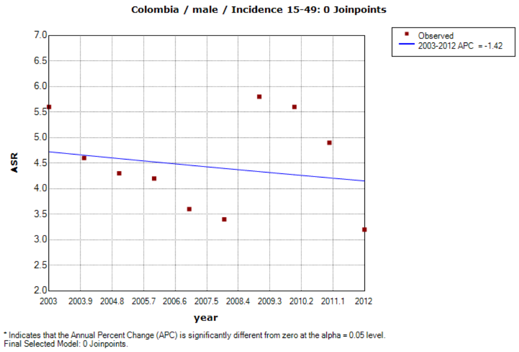

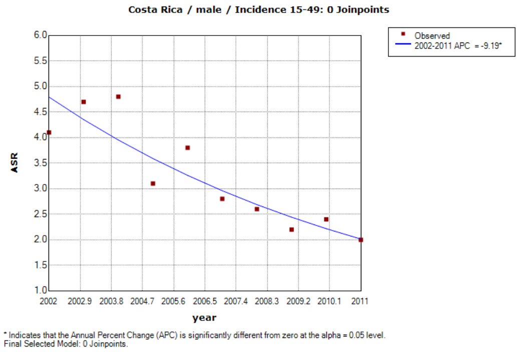

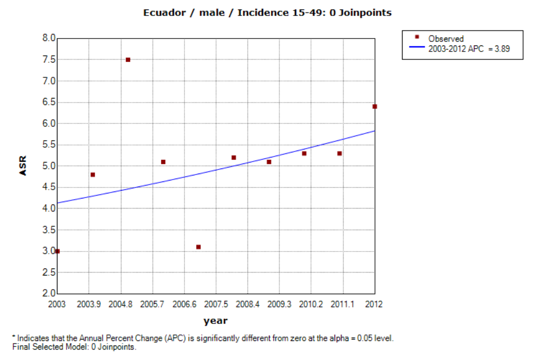

Northern Europe

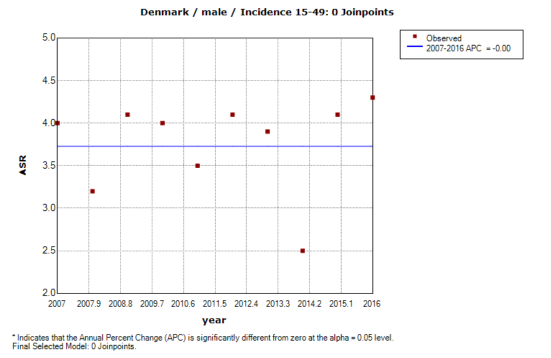

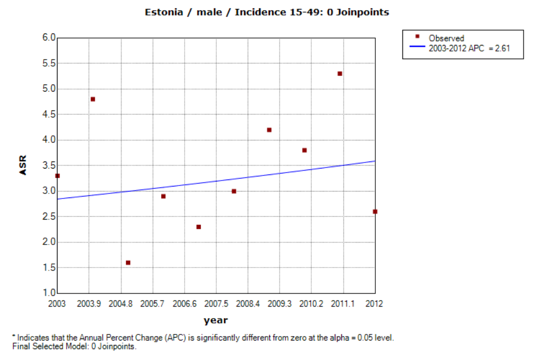

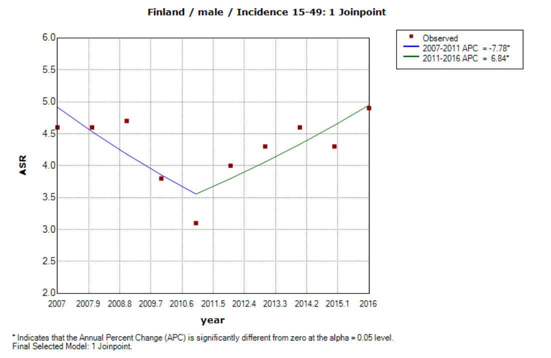

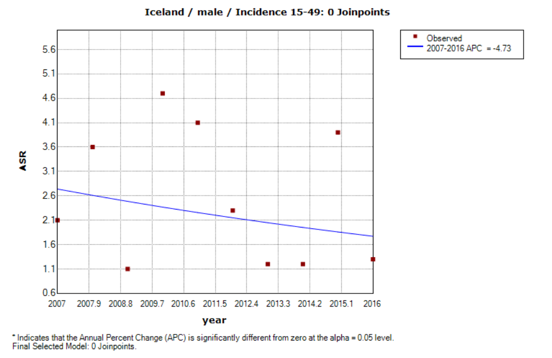

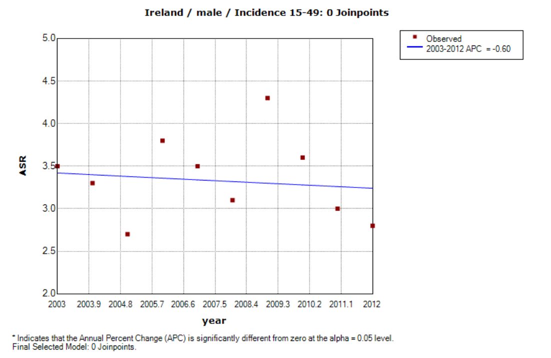

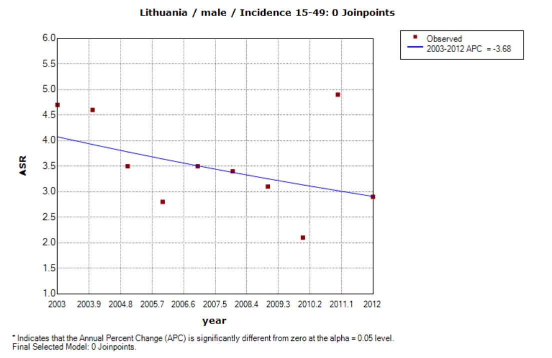

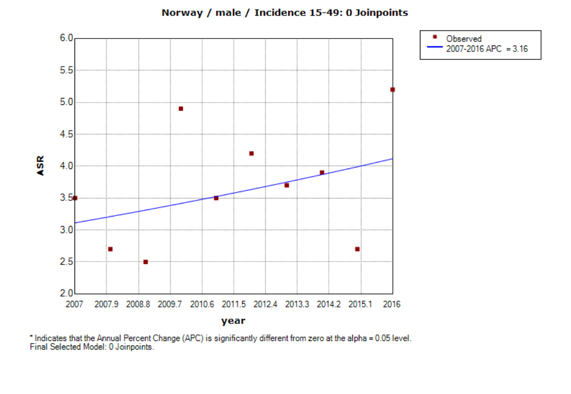

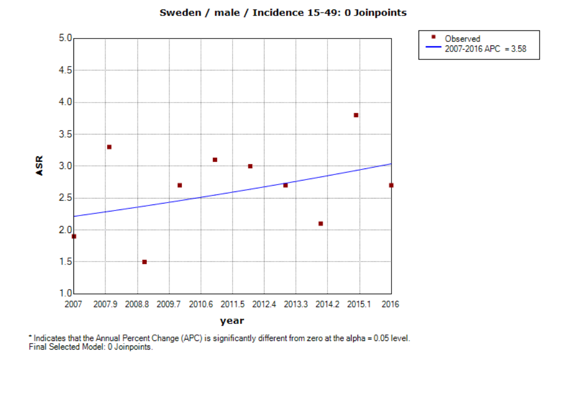

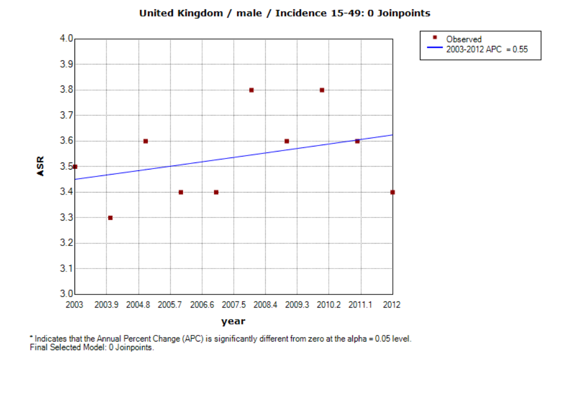

Western Europe

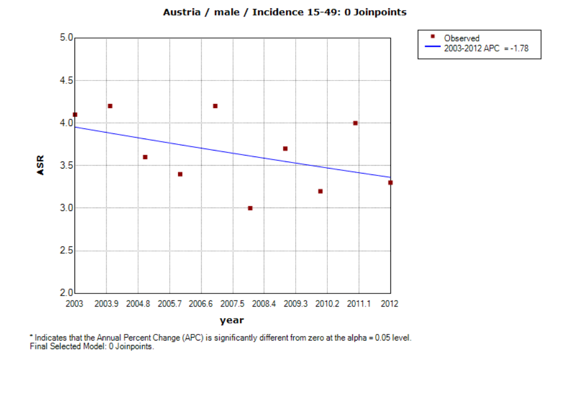

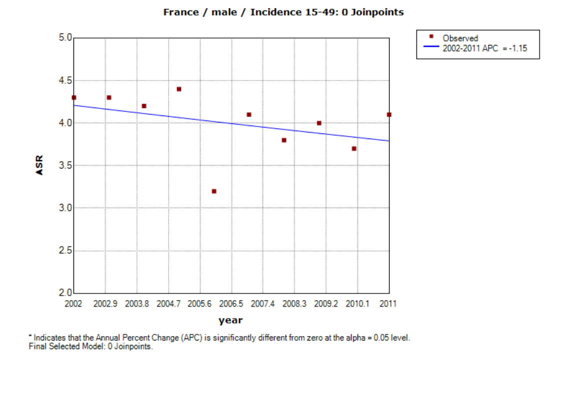

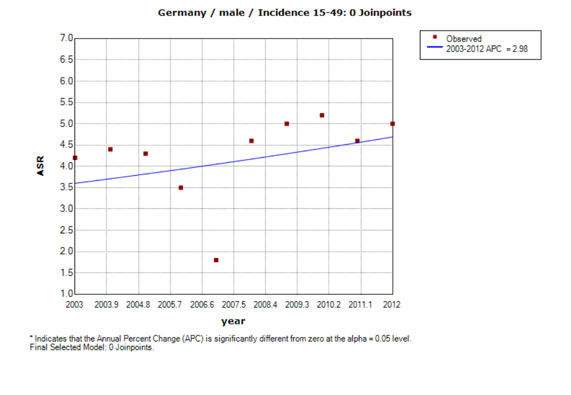

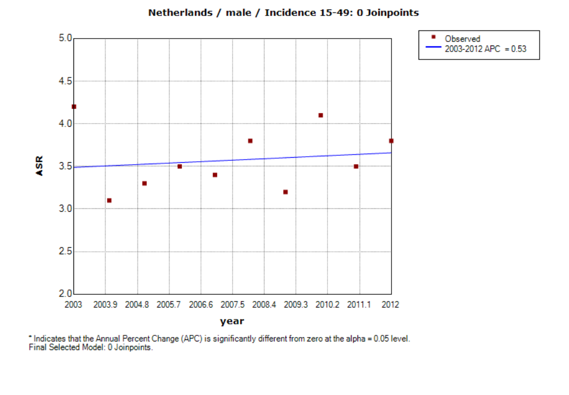

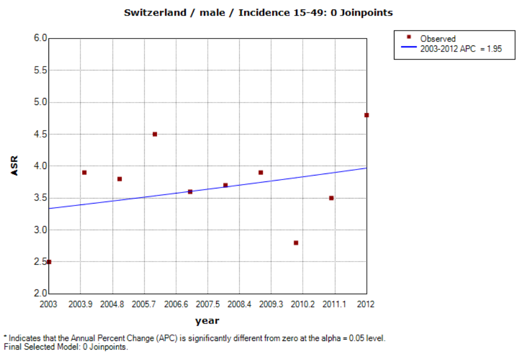

Southern Europe

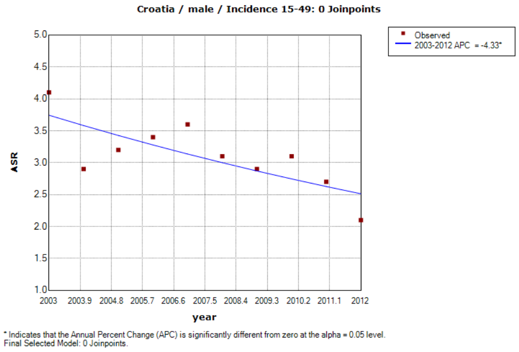

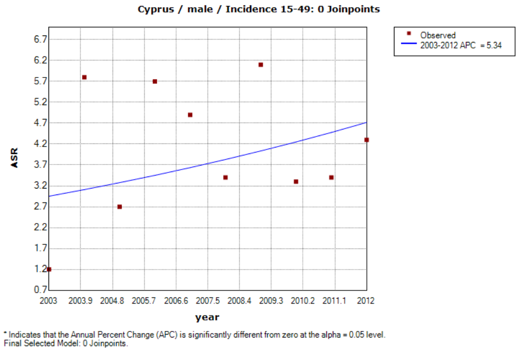

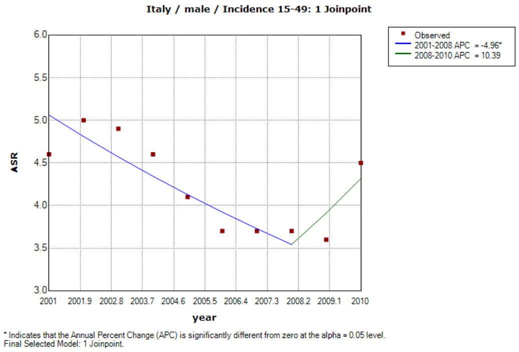

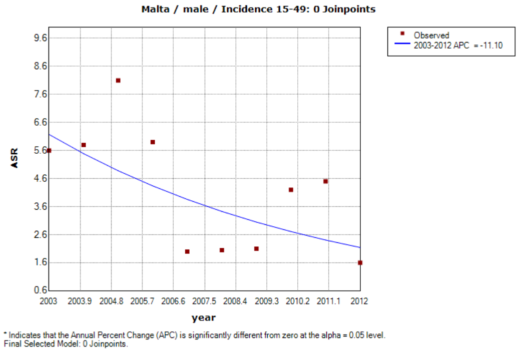

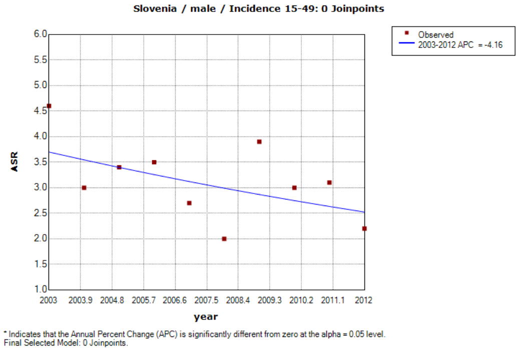

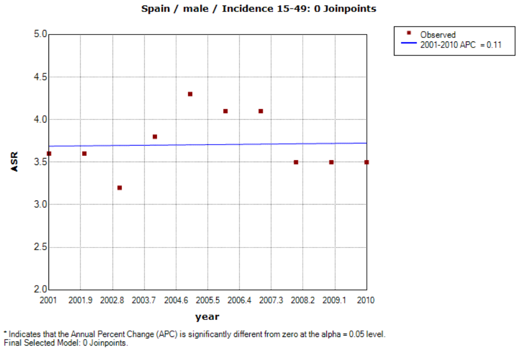

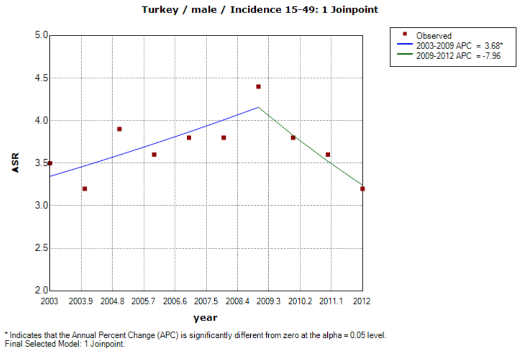

Eastern Europe

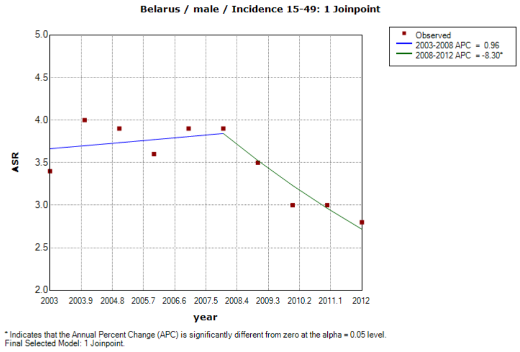

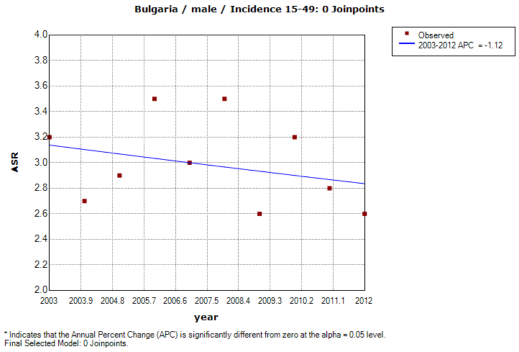

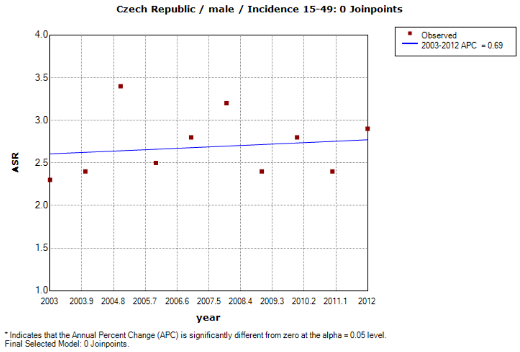

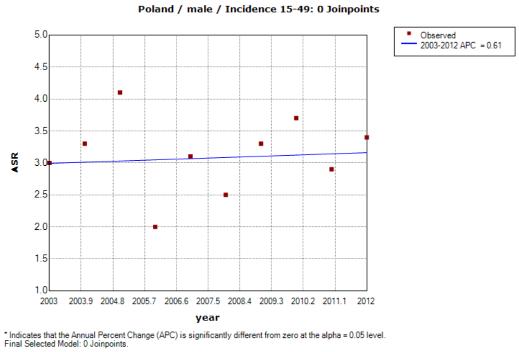

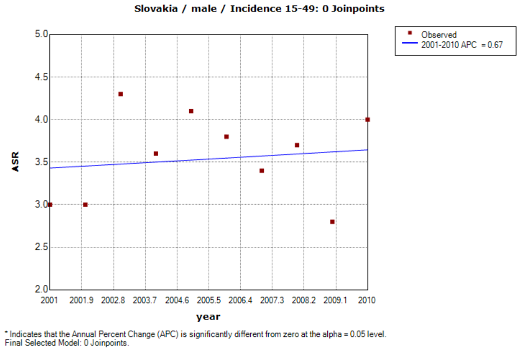

Africa

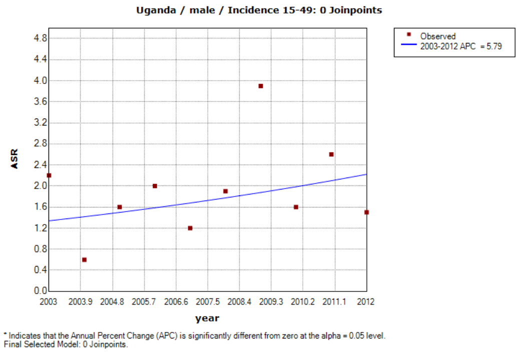
Uganda / male / Incidence 15-49: 0 Joinpoints
Observed
2003-2012 APC = 5.79
ASR
year
* Indicates that the Annual Percent Change (APC) is significantly different from zero at the alpha = 0.05 level.
Final Selected Model: 0 Joinpoints.

f.) Incidence female between 15-49 years old

Asia

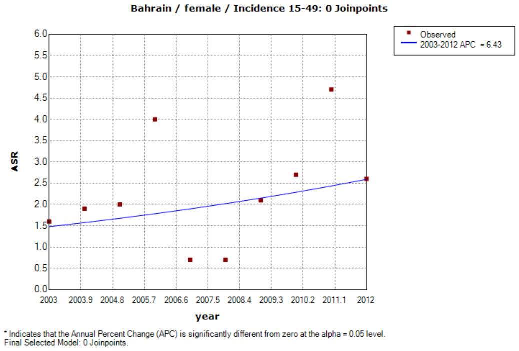

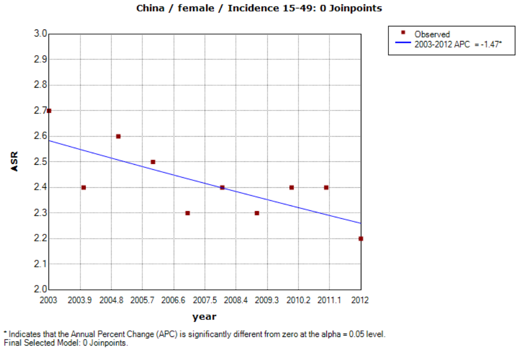

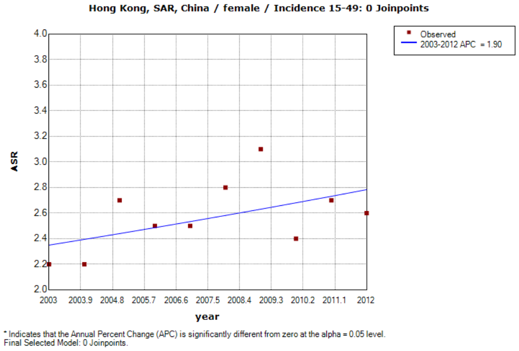

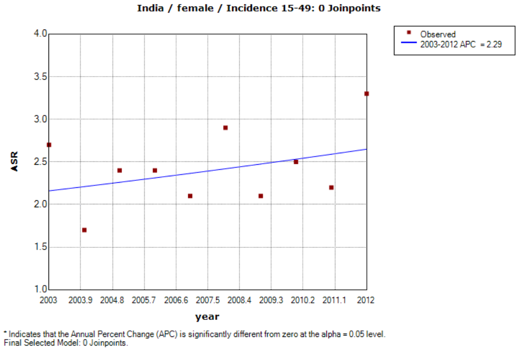

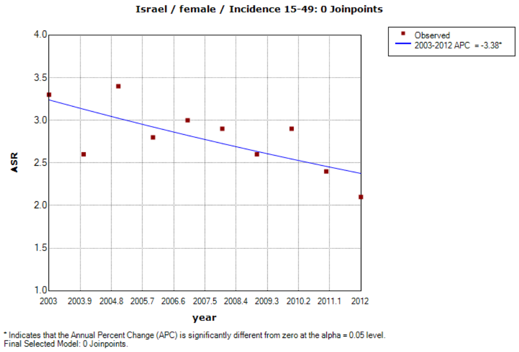

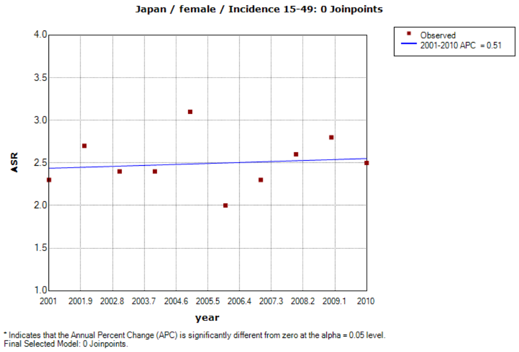

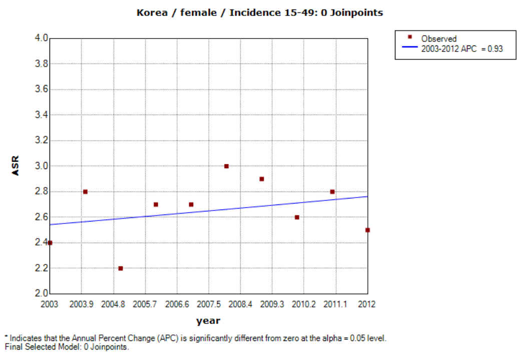
Korea / female / Incidence 15-49: 0 Joinpoints
Observed
2003-2012 APC = 0.93
ASR
year
* Indicates that the Annual Percent Change (APC) is significantly different from zero at the alpha = 0.05 level.
Final Selected Model: 0 Joinpoints.

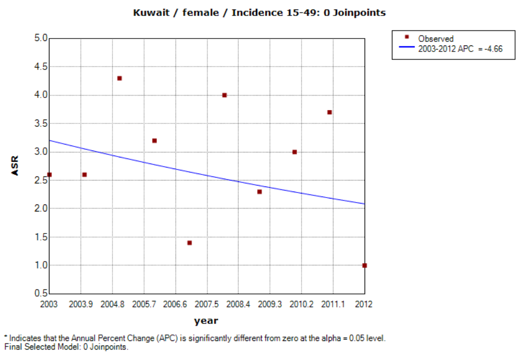
Kuwait / female / Incidence 15-49: 0 Joinpoints
Observed
2003-2012 APC = -4.66
ASR
year
* Indicates that the Annual Percent Change (APC) is significantly different from zero at the alpha = 0.05 level.
Final Selected Model: 0 Joinpoints.

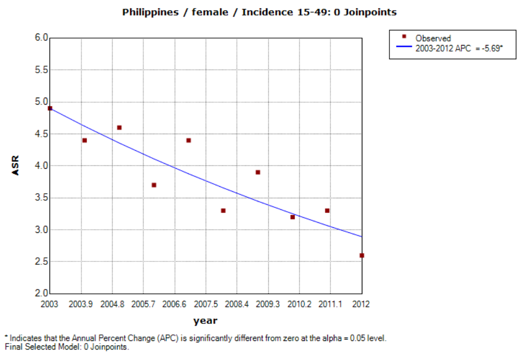
Philippines / female / Incidence 15-49: 0 Joinpoints
Observed
2003-2012 APC = -5.69*
ASR
year
* Indicates that the Annual Percent Change (APC) is significantly different from zero at the alpha = 0.05 level.
Final Selected Model: 0 Joinpoints.

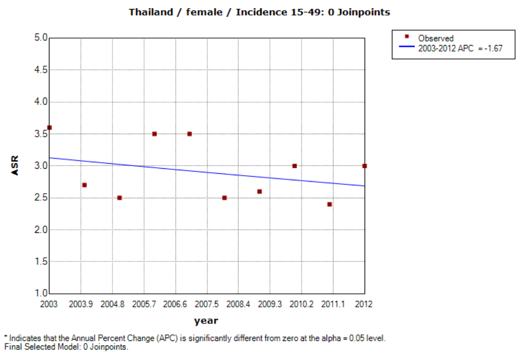
Thailand / female / Incidence 15-49: 0 Joinpoints
Observed
2003-2012 APC = -1.67
ASR
year
* Indicates that the Annual Percent Change (APC) is significantly different from zero at the alpha = 0.05 level.
Final Selected Model: 0 Joinpoints.

Oceania

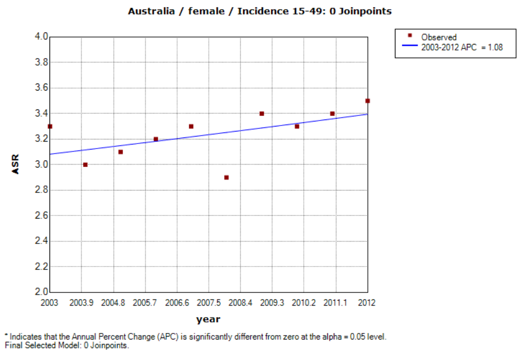
Australia / female / Incidence 15-49: 0 Joinpoints
Observed
2003-2012 APC = 1.08
ASR
year
* Indicates that the Annual Percent Change (APC) is significantly different from zero at the alpha = 0.05 level.
Final Selected Model: 0 Joinpoints.

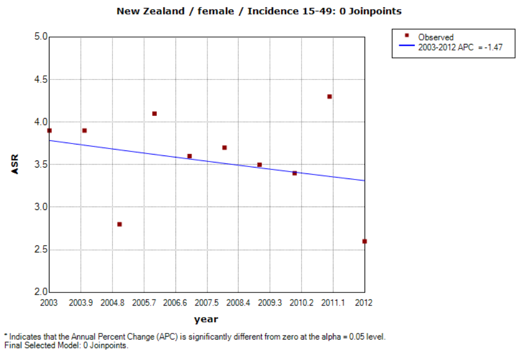
New Zealand / female / Incidence 15-49: 0 Joinpoints
Observed
2003-2012 APC = -1.47
ASR
year
* Indicates that the Annual Percent Change (APC) is significantly different from zero at the alpha = 0.05 level.
Final Selected Model: 0 Joinpoints.

Northern America

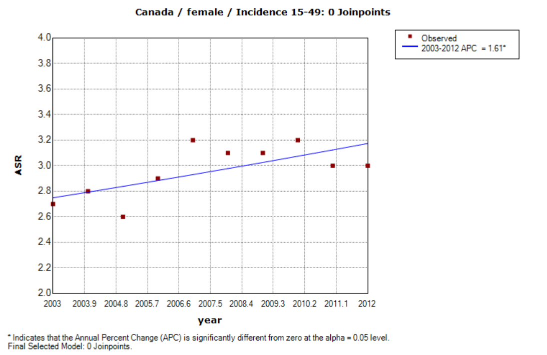

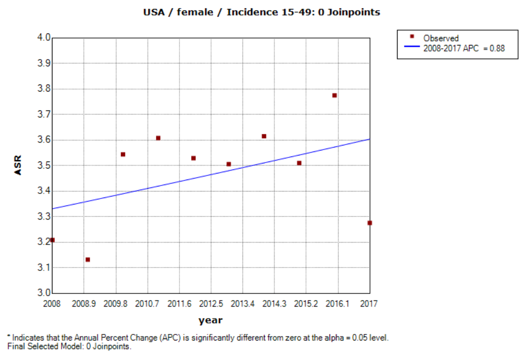

Southern America

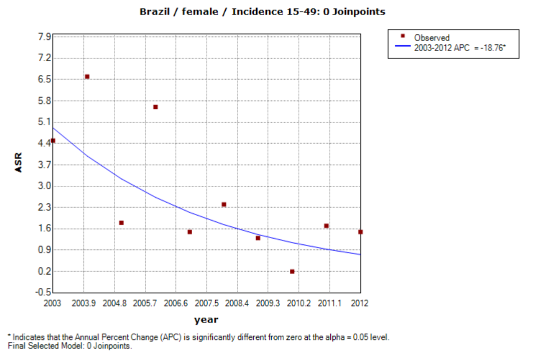

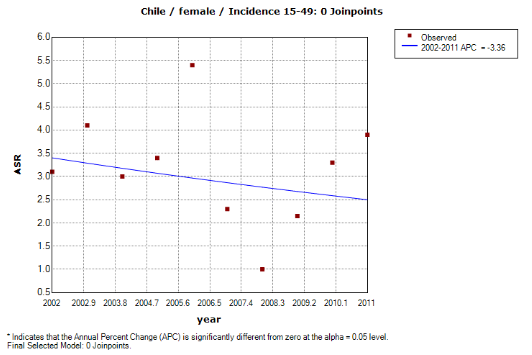

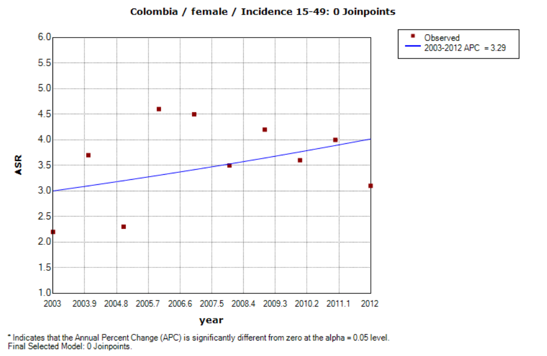

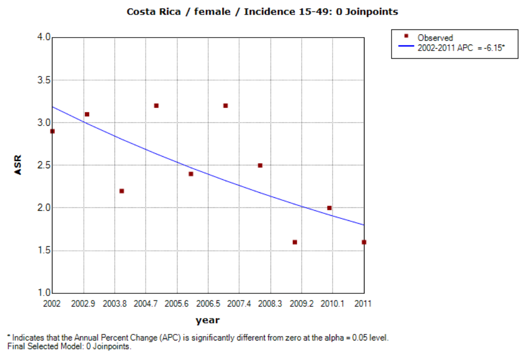

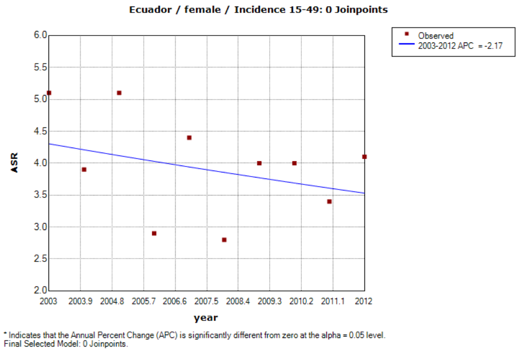

Northern Europe

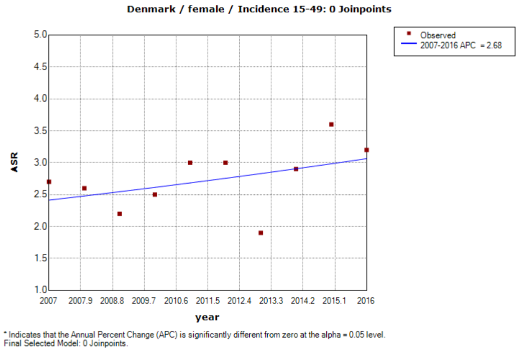

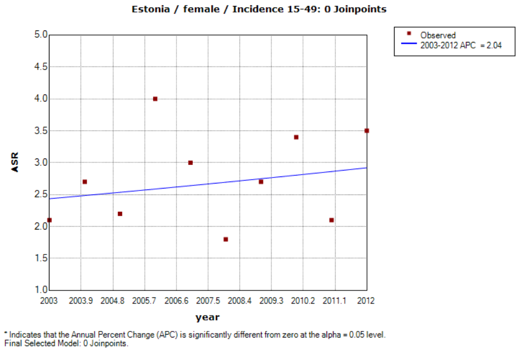

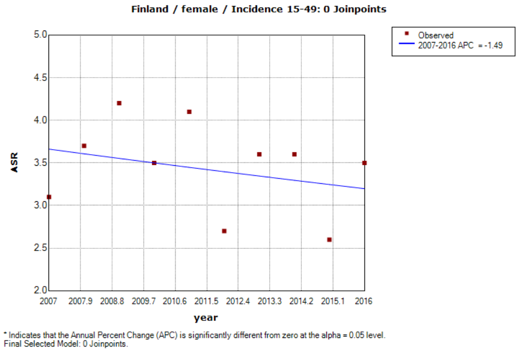

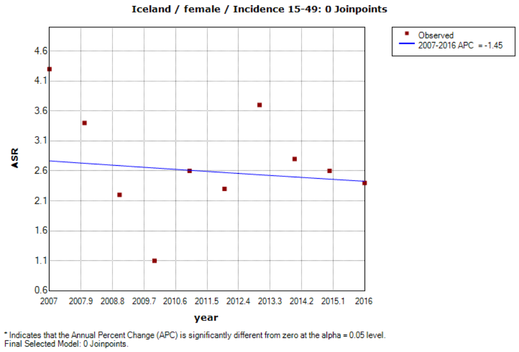

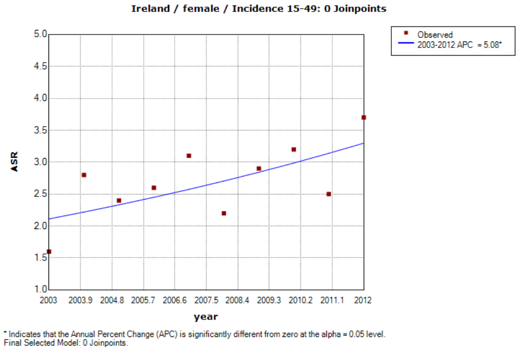

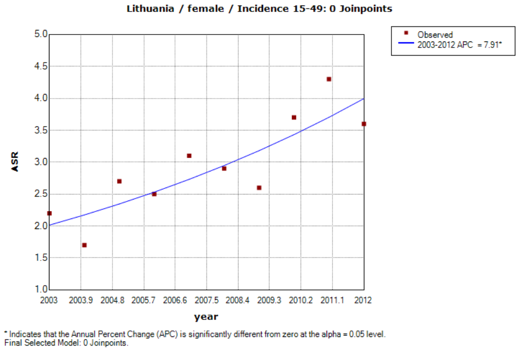

Norway / female / Incidence 15-49: 0 Joinpoints

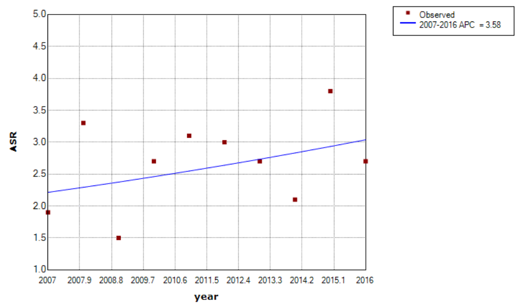

* Indicates that the Annual Percent Change (APC) is significantly different from zero at the alpha = 0.05 level.
Final Selected Model: 0 Joinpoints.

Sweden / female / Incidence 15-49: 0 Joinpoints

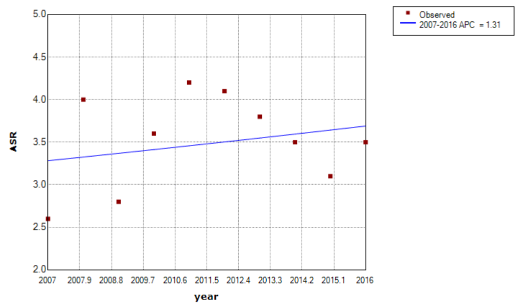

* Indicates that the Annual Percent Change (APC) is significantly different from zero at the alpha = 0.05 level.
Final Selected Model: 0 Joinpoints.

United Kingdom / female / Incidence 15-49: 0 Joinpoints

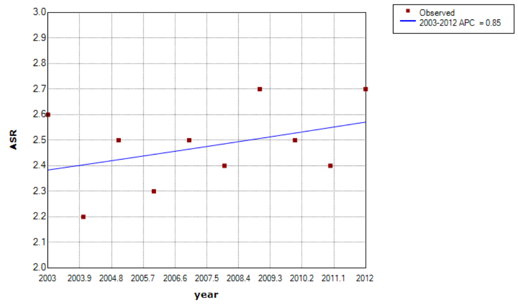

* Indicates that the Annual Percent Change (APC) is significantly different from zero at the alpha = 0.05 level.
Final Selected Model: 0 Joinpoints.

Western Europe

Austria / female / Incidence 15-49: 0 Joinpoints

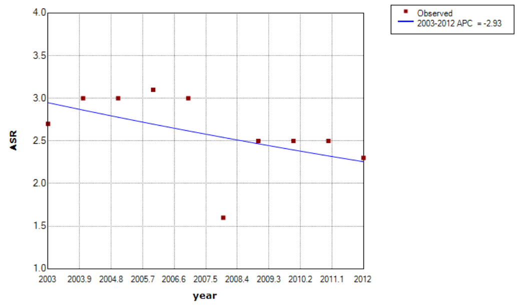

* Indicates that the Annual Percent Change (APC) is significantly different from zero at the alpha = 0.05 level.
Final Selected Model: 0 Joinpoints.

France / female / Incidence 15-49: 0 Joinpoints

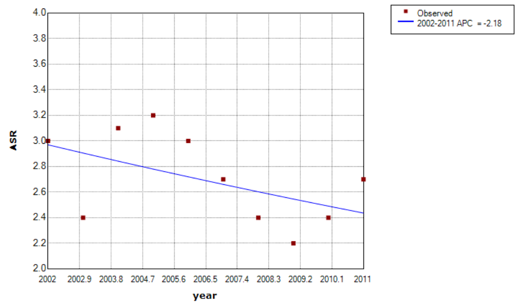

* Indicates that the Annual Percent Change (APC) is significantly different from zero at the alpha = 0.05 level.
Final Selected Model: 0 Joinpoints.

Germany / female / Incidence 15-49: 0 Joinpoints

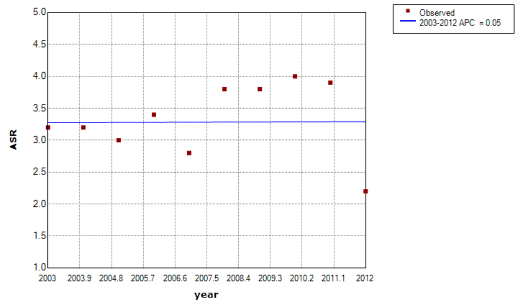

* Indicates that the Annual Percent Change (APC) is significantly different from zero at the alpha = 0.05 level.
Final Selected Model: 0 Joinpoints.

Netherlands / female / Incidence 15-49: 1 Joinpoint

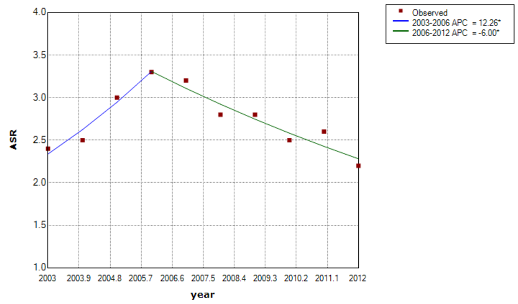

* Indicates that the Annual Percent Change (APC) is significantly different from zero at the alpha = 0.05 level.
Final Selected Model: 1 Joinpoint.

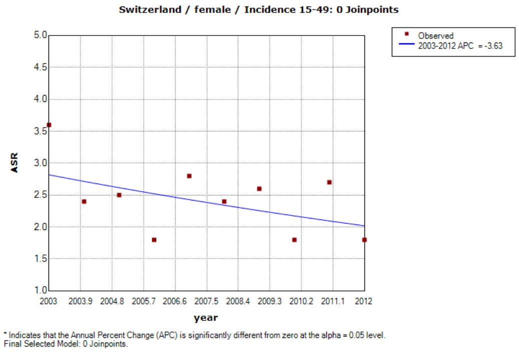

Southern Europe

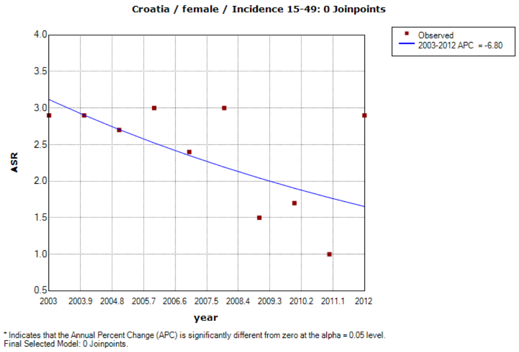

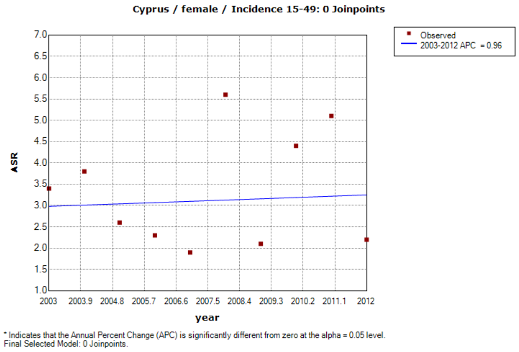

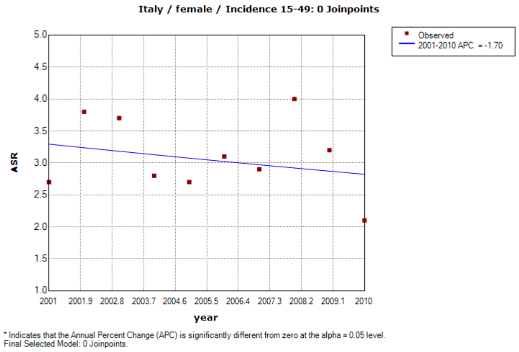

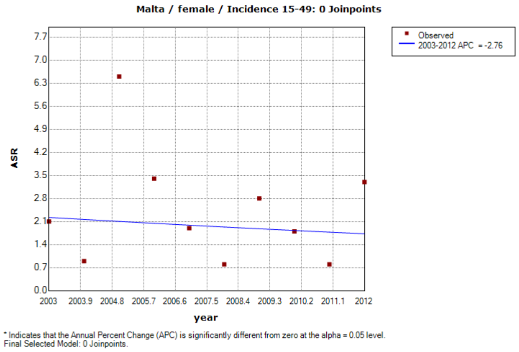

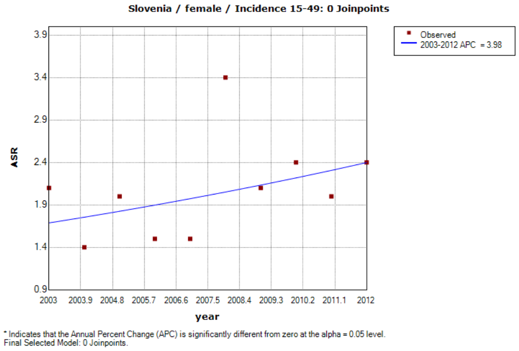

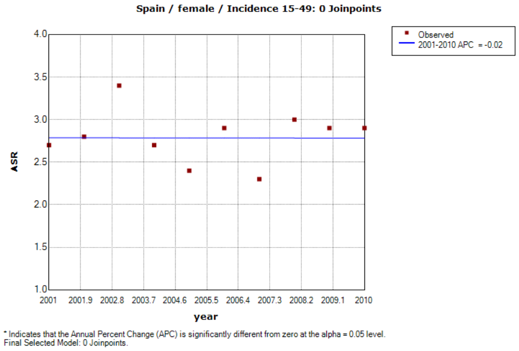

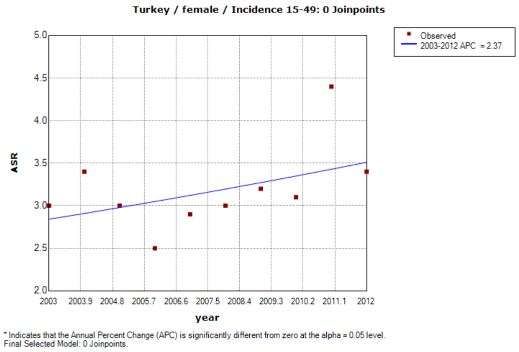

Eastern Europe

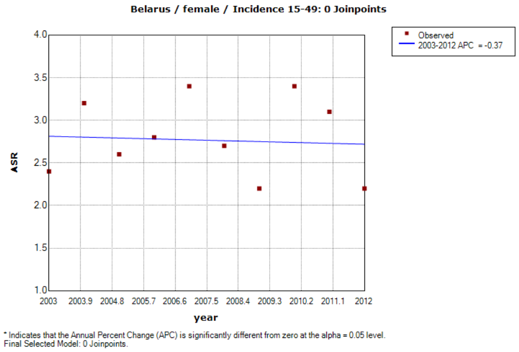

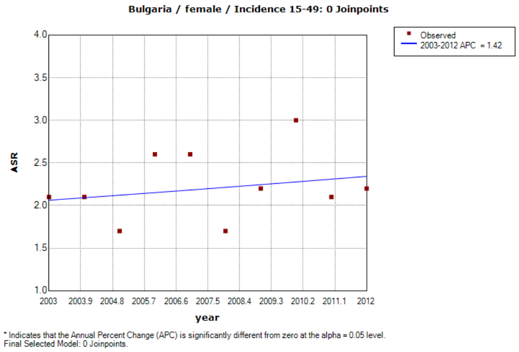

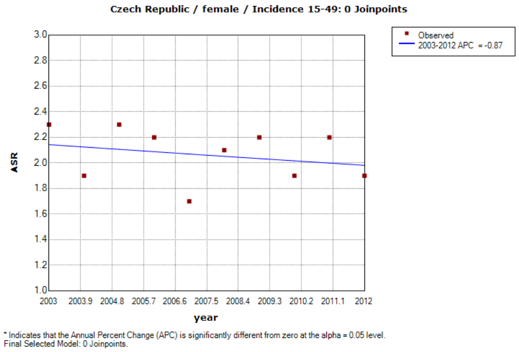

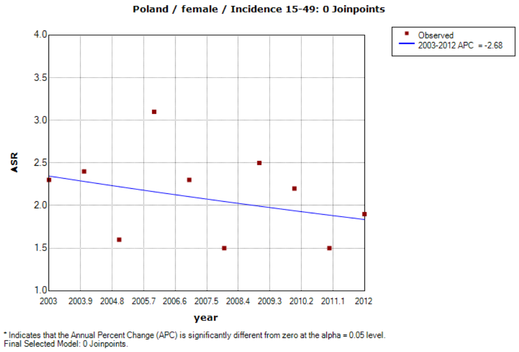

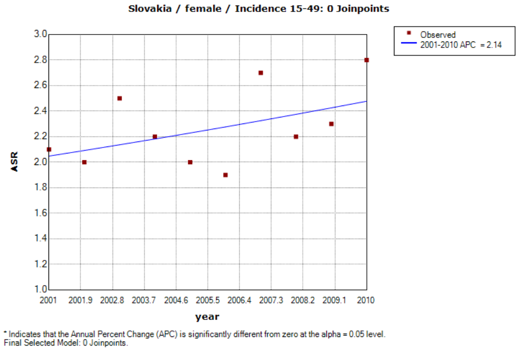

Africa	
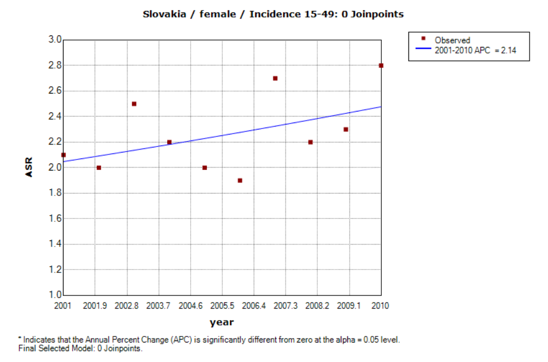Slovakia / female / Incidence 15-49: 0 Joinpoints<br>Observed<br>2001-2010 APC = 2.14<br>ASR<br>year<br>* Indicates that the Annual Percent Change (APC) is significantly different from zero at the alpha = 0.05 level.<br>Final Selected Model: 0 Joinpoints.	

g.) Incidence male above 50 years old

Asia

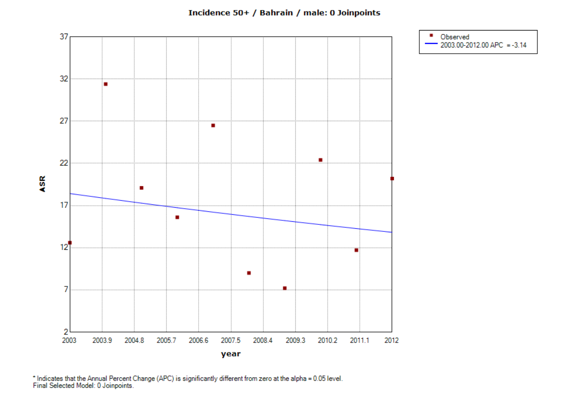

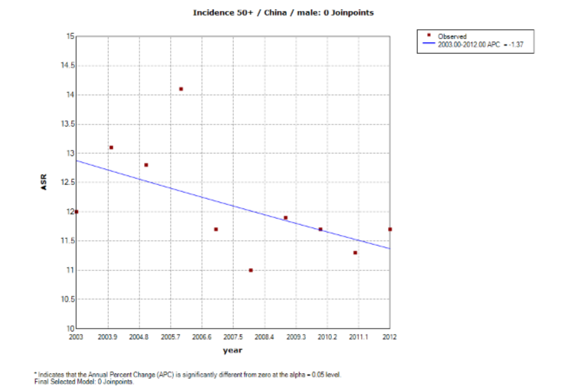

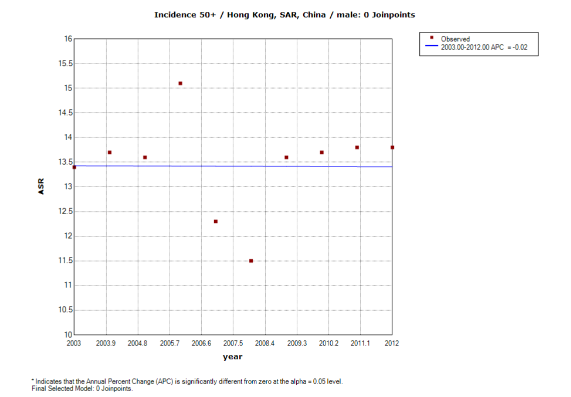

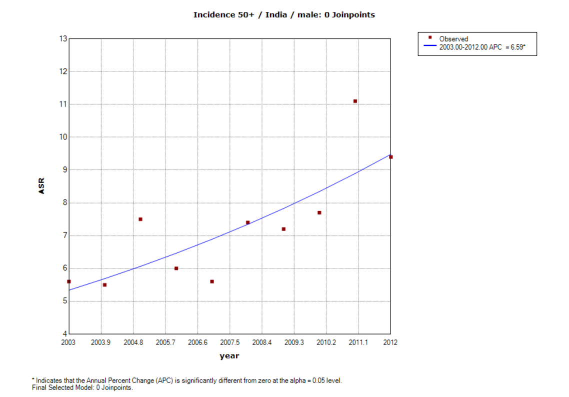

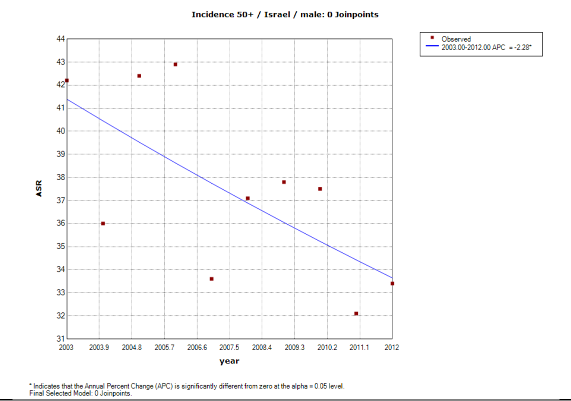

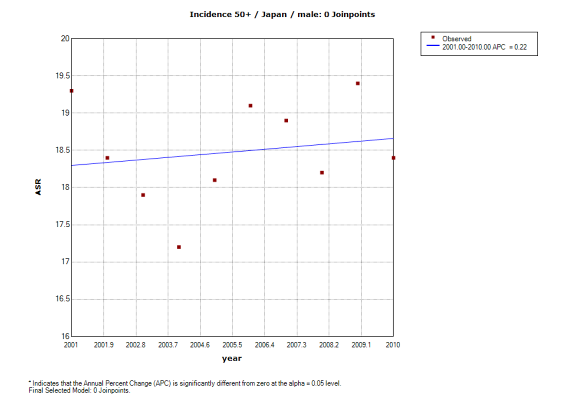

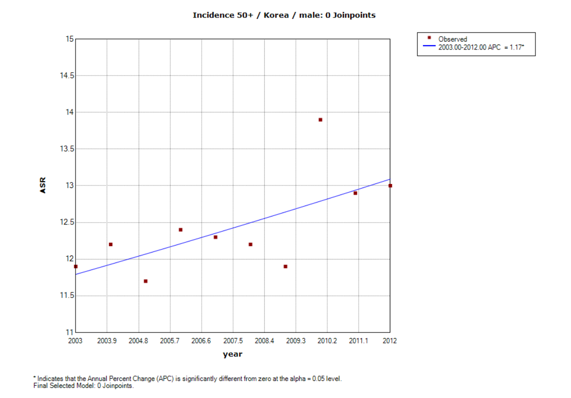
Incidence 50+ / Korea / male: 0 Joinpoints
Observed
2003.00-2012.00 APC = 1.17*
ASR
year
* Indicates that the Annual Percent Change (APC) is significantly different from zero at the alpha = 0.05 level.
Final Selected Model: 0 Joinpoints.

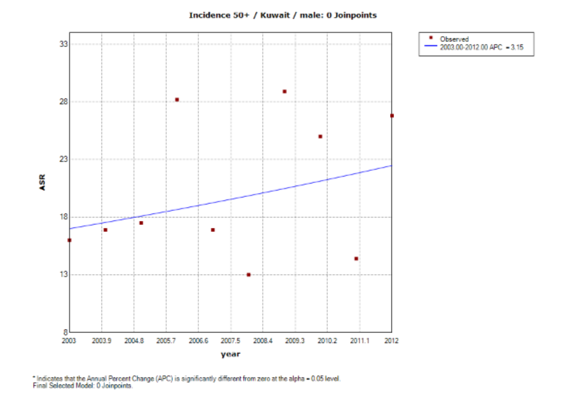
Incidence 50+ / Kuwait / male: 0 Joinpoints
Observed
2003.00-2012.00 APC = 3.15
ASR
year
* Indicates that the Annual Percent Change (APC) is significantly different from zero at the alpha = 0.05 level.
Final Selected Model: 0 Joinpoints.

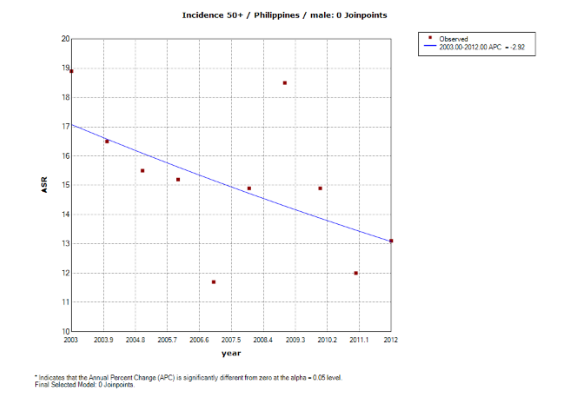
Incidence 50+ / Philippines / male: 0 Joinpoints
Observed
2003.00-2012.00 APC = -2.92
ASR
year
* Indicates that the Annual Percent Change (APC) is significantly different from zero at the alpha = 0.05 level.
Final Selected Model: 0 Joinpoints.

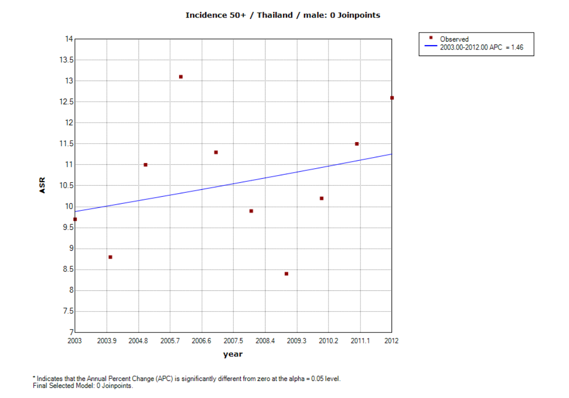
Incidence 50+ / Thailand / male: 0 Joinpoints
Observed
2003.00-2012.00 APC = 1.46
ASR
year
* Indicates that the Annual Percent Change (APC) is significantly different from zero at the alpha = 0.05 level.
Final Selected Model: 0 Joinpoints.

Oceania

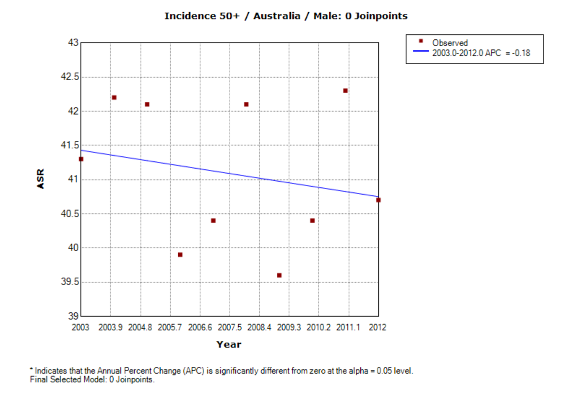
Incidence 50+ / Australia / Male: 0 Joinpoints
Observed
2003.0-2012.0 APC = -0.18
ASR
Year
* Indicates that the Annual Percent Change (APC) is significantly different from zero at the alpha = 0.05 level.
Final Selected Model: 0 Joinpoints.

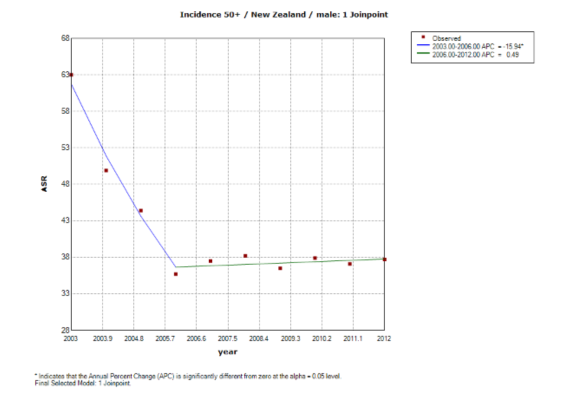
Incidence 50+ / New Zealand / male: 1 Joinpoint
Observed
2003.00-2006.00 APC = -15.94*
2006.00-2012.00 APC = 0.49
ASR
year
* Indicates that the Annual Percent Change (APC) is significantly different from zero at the alpha = 0.05 level.
Final Selected Model: 1 Joinpoint.

Northern America

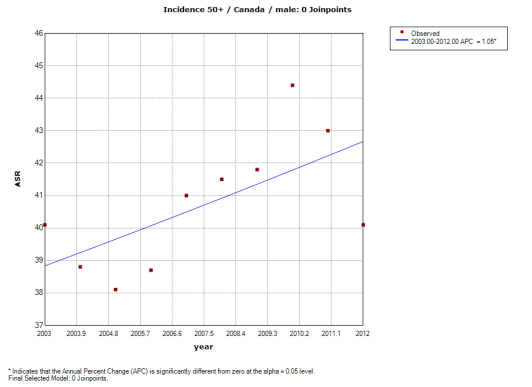

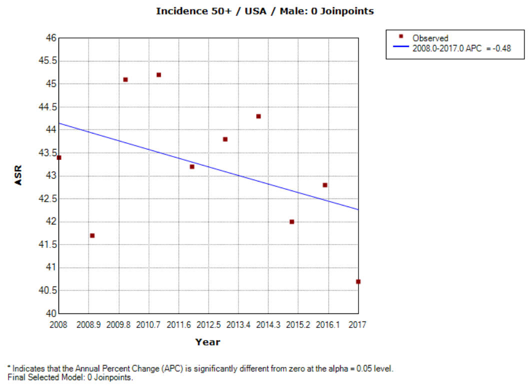

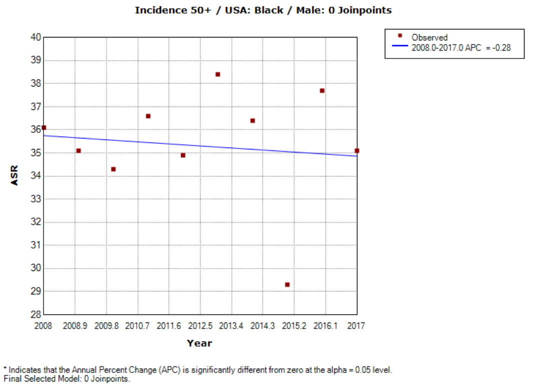

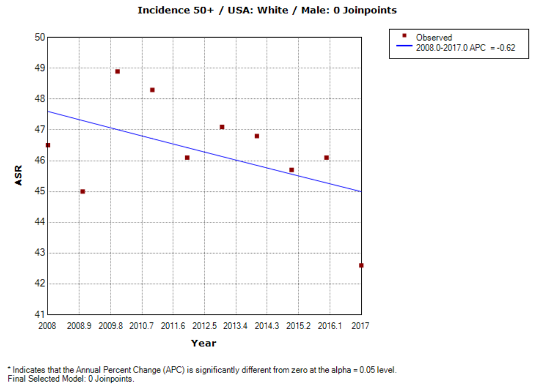

Southern America

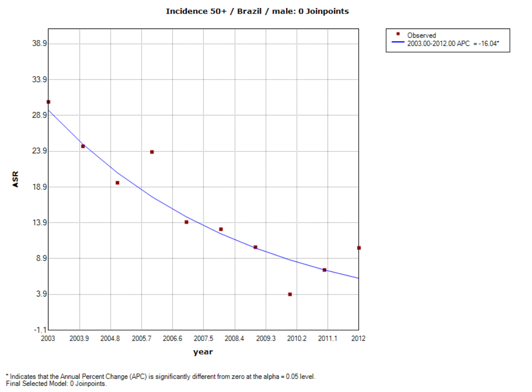

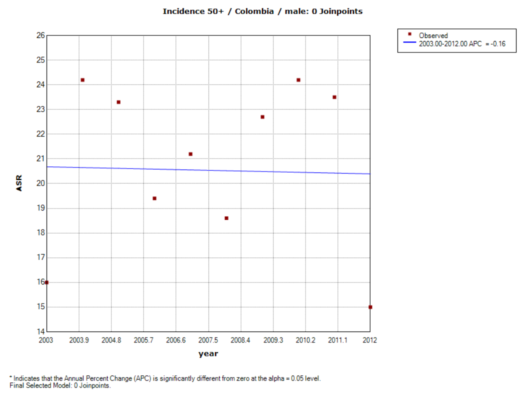

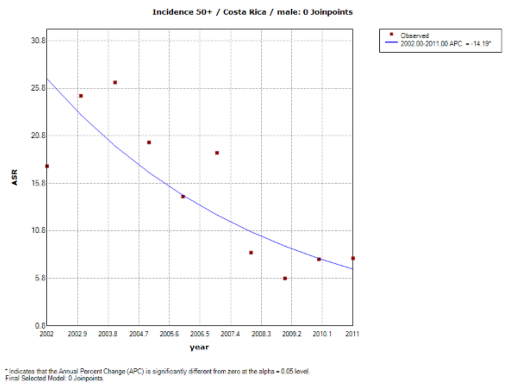

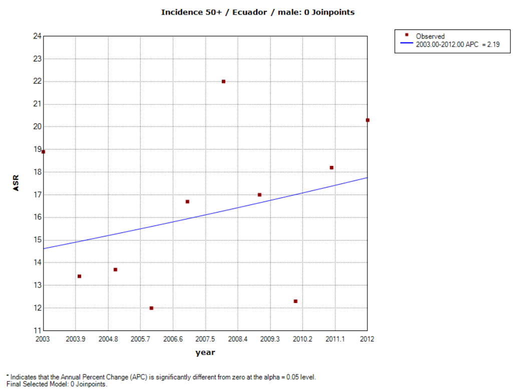

Northern Europe

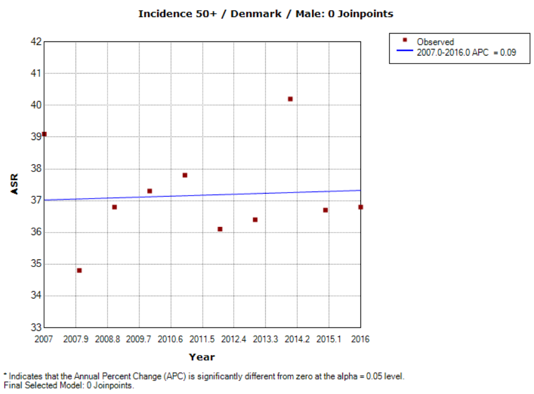

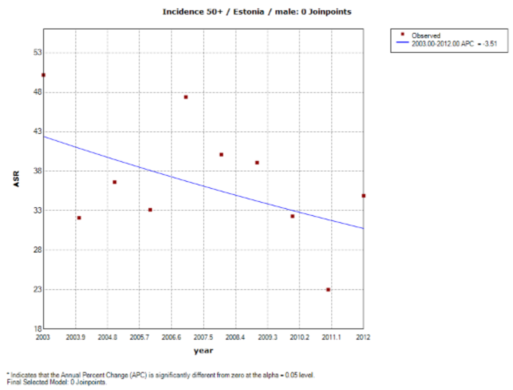

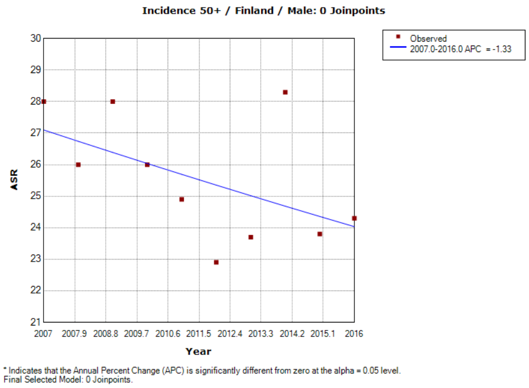

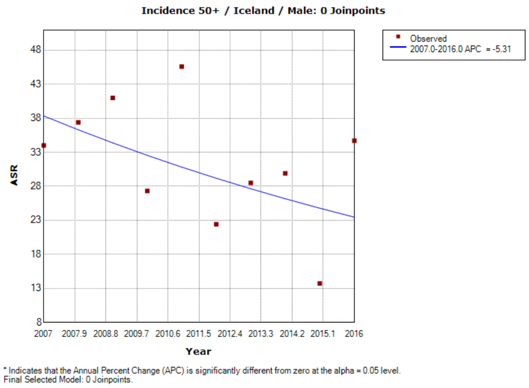

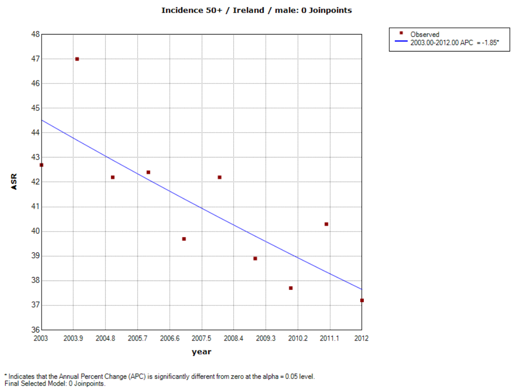

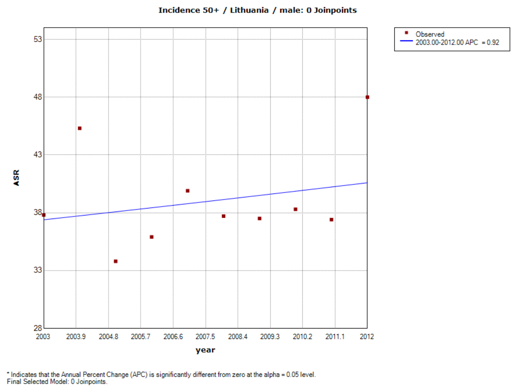

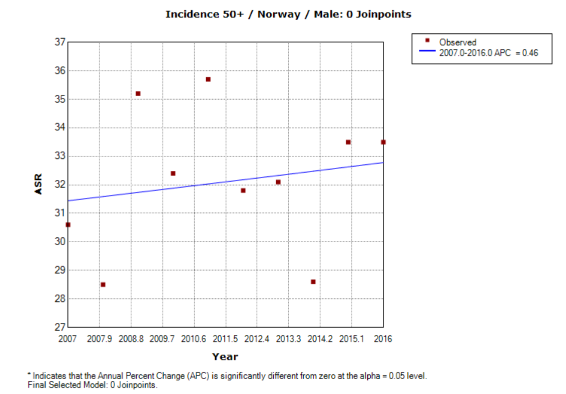

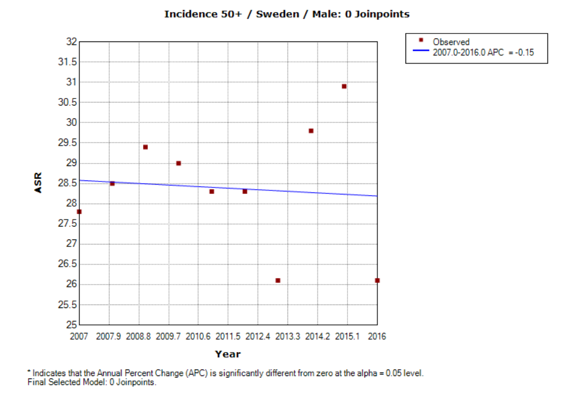

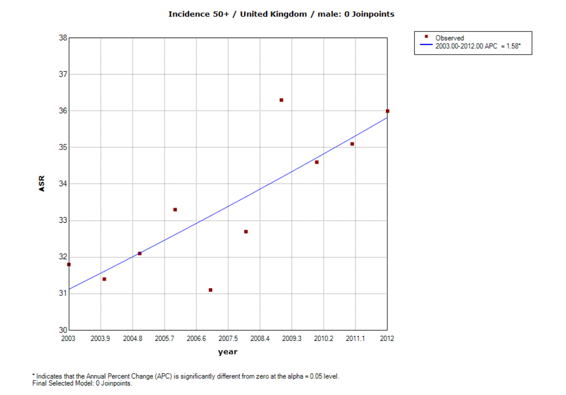

Western Europe

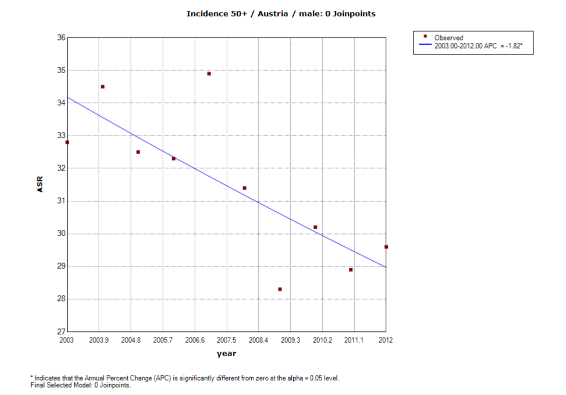

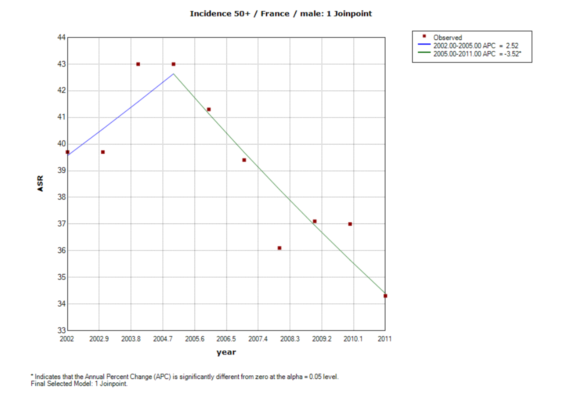

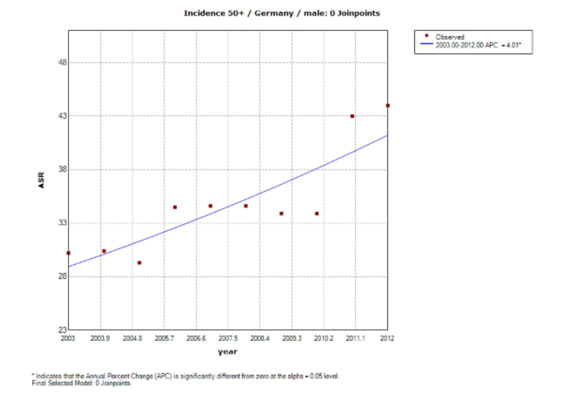

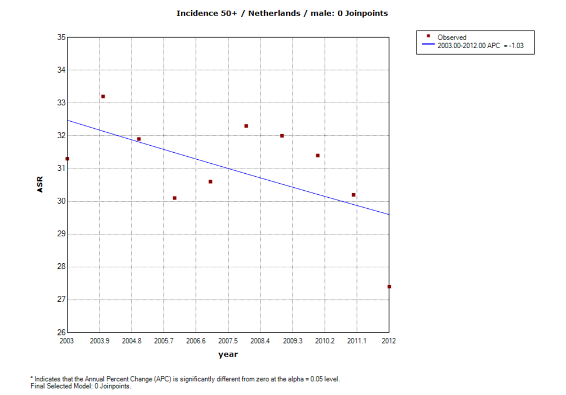

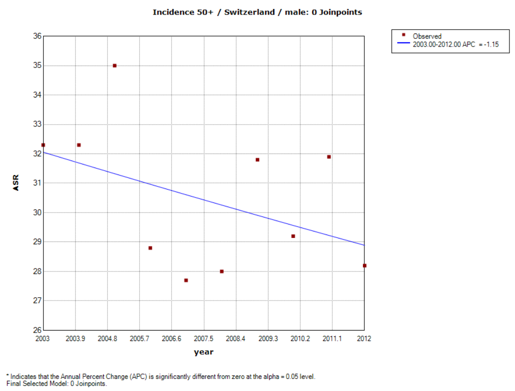

Southern Europe

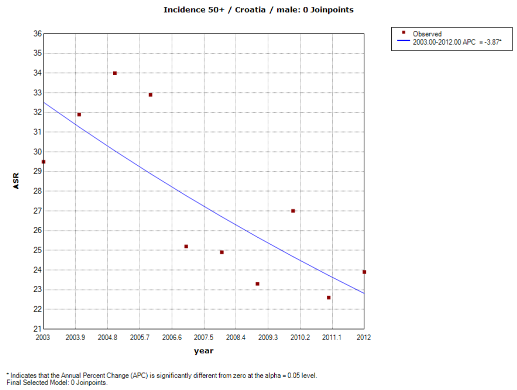

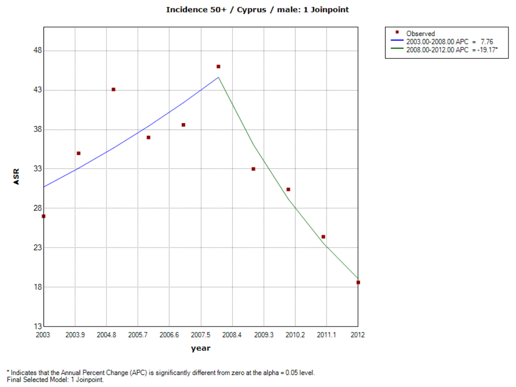

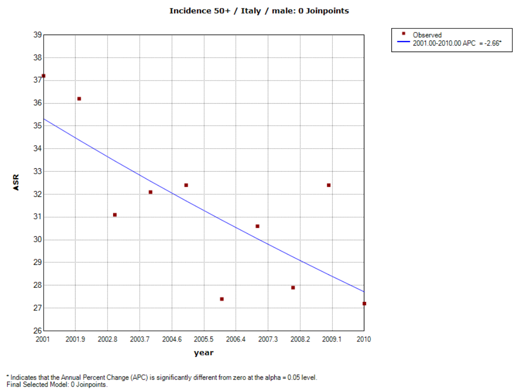

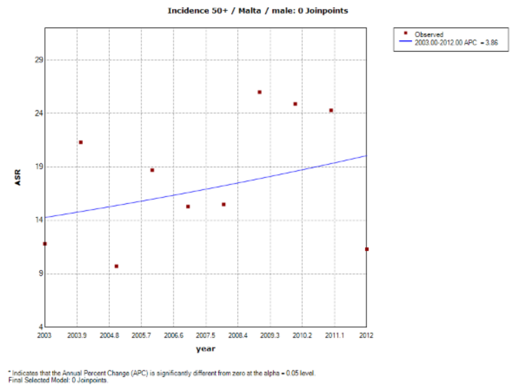

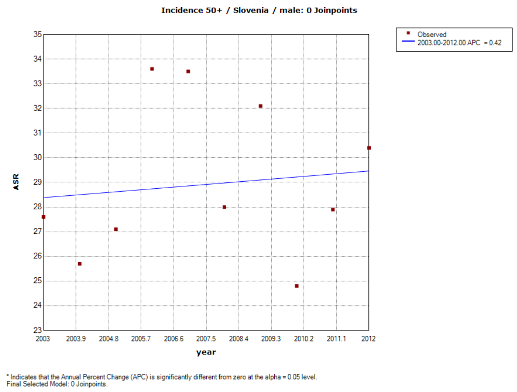

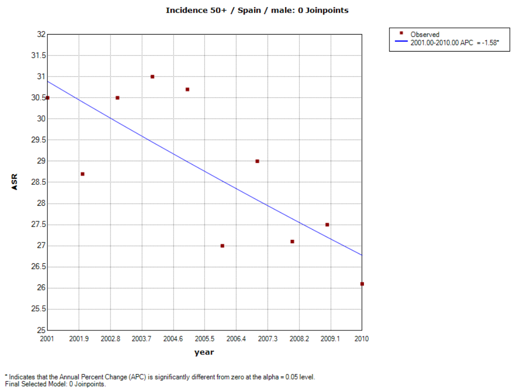

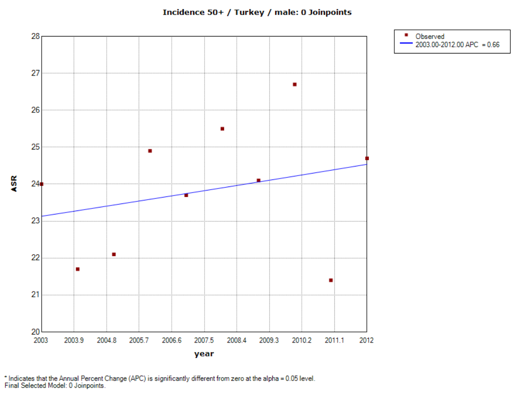

Eastern Europe

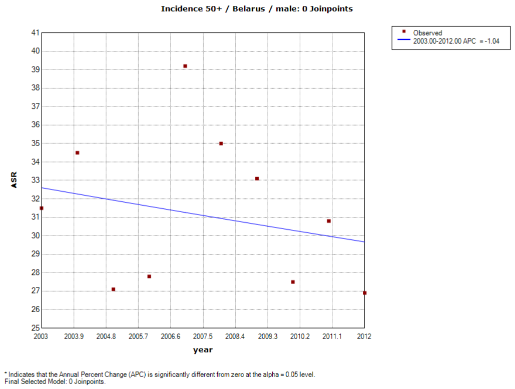

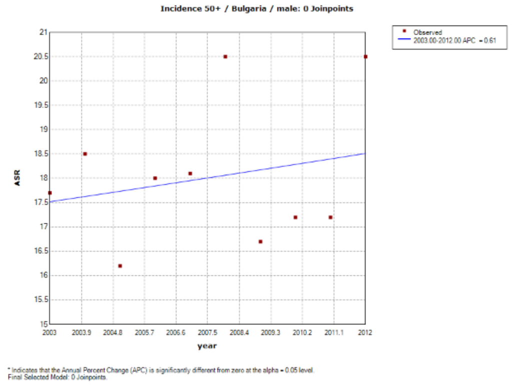

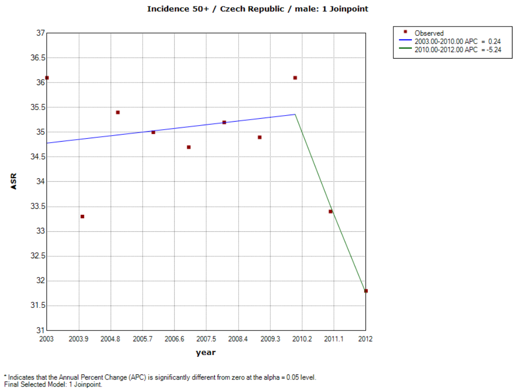

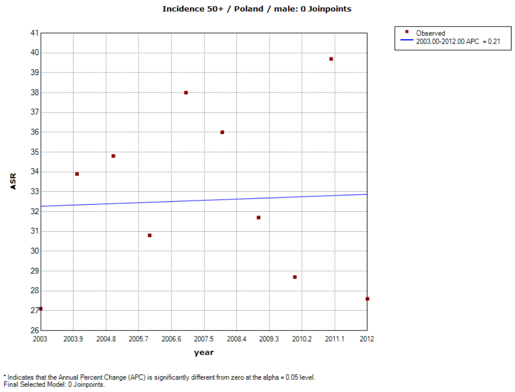

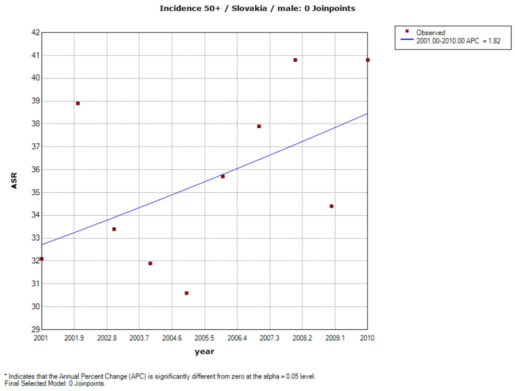

Africa

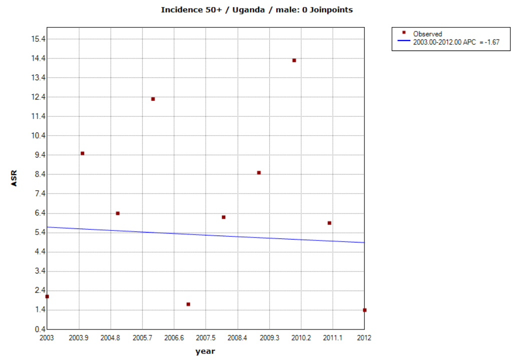

* Indicates that the Annual Percent Change (APC) is significantly different from zero at the alpha = 0.05 level.
Final Selected Model: 0 Joinpoints.

h.) Incidence female above 50 years old

Asia

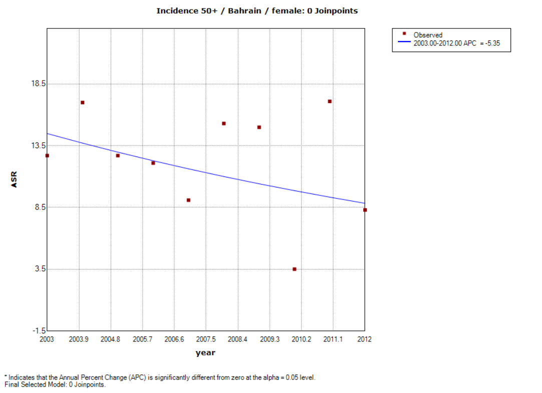

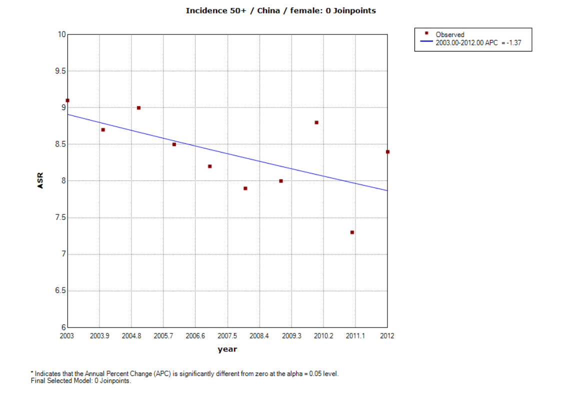

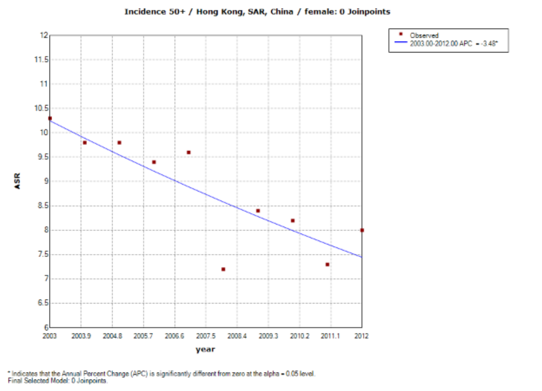

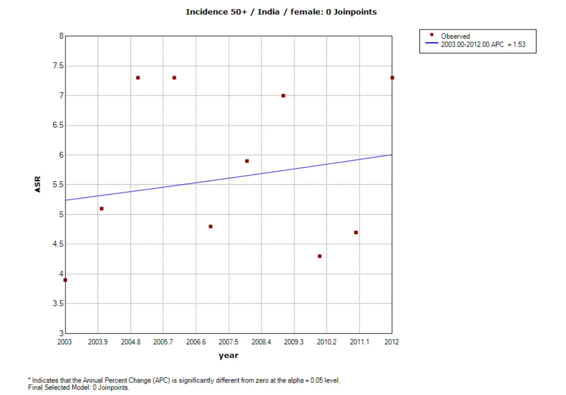

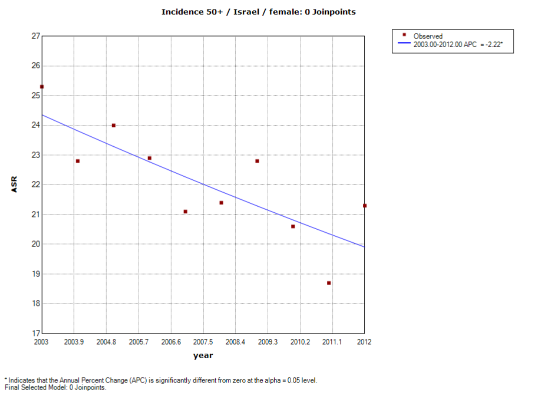

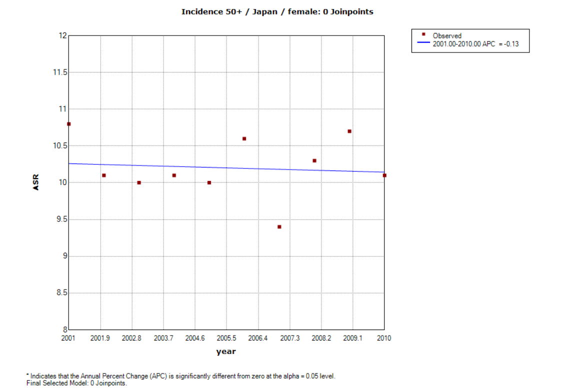

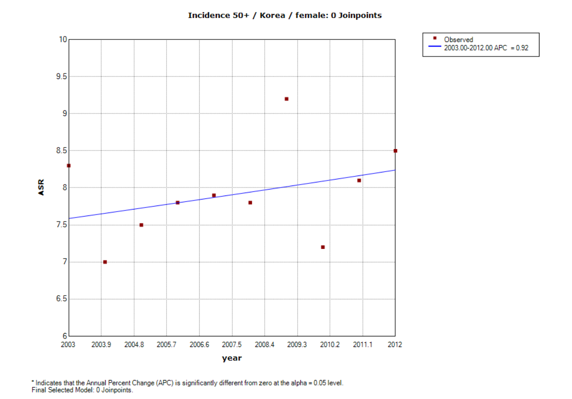
Incidence 50+ / Korea / female: 0 Joinpoints
Observed
2003.00-2012.00 APC = 0.92
ASR
year
* Indicates that the Annual Percent Change (APC) is significantly different from zero at the alpha = 0.05 level.
Final Selected Model: 0 Joinpoints.

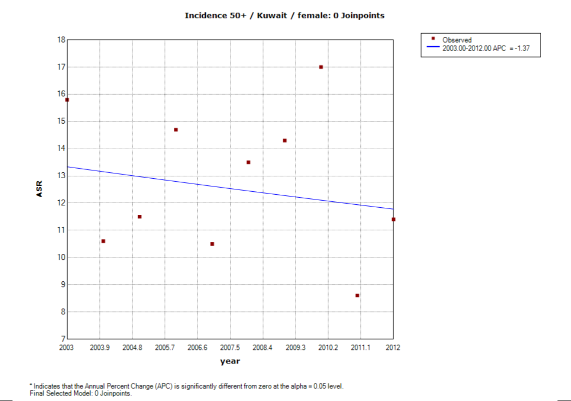
Incidence 50+ / Kuwait / female: 0 Joinpoints
Observed
2003.00-2012.00 APC = -1.37
ASR
year
* Indicates that the Annual Percent Change (APC) is significantly different from zero at the alpha = 0.05 level.
Final Selected Model: 0 Joinpoints.

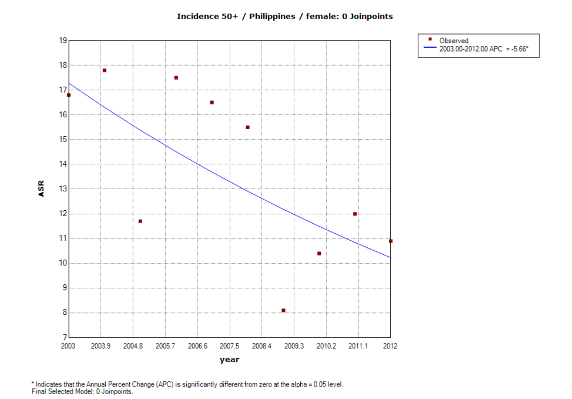
Incidence 50+ / Philippines / female: 0 Joinpoints
Observed
2003.00-2012.00 APC = -5.66*
ASR
year
* Indicates that the Annual Percent Change (APC) is significantly different from zero at the alpha = 0.05 level.
Final Selected Model: 0 Joinpoints.

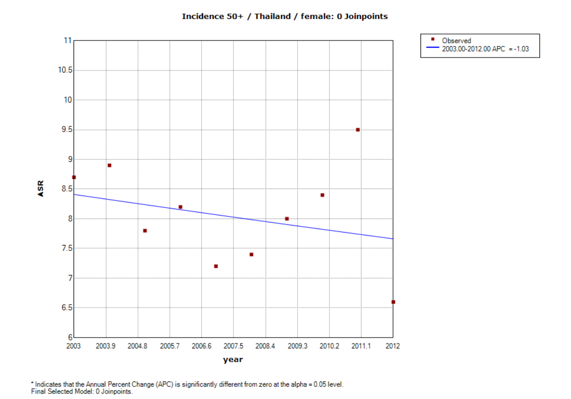
Incidence 50+ / Thailand / female: 0 Joinpoints
Observed
2003.00-2012.00 APC = -1.03
ASR
year
* Indicates that the Annual Percent Change (APC) is significantly different from zero at the alpha = 0.05 level.
Final Selected Model: 0 Joinpoints.

Oceania

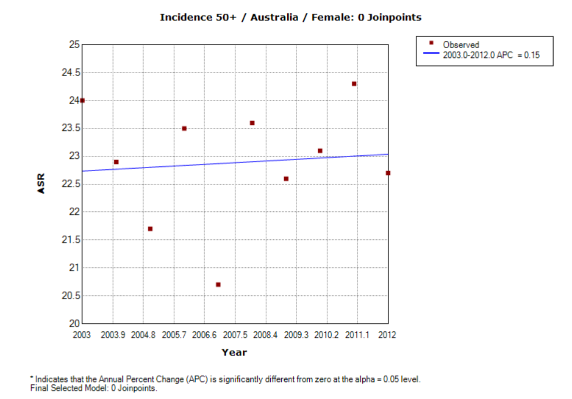
Incidence 50+ / Australia / Female: 0 Joinpoints
Observed
2003.0-2012.0 APC = 0.15
ASR
Year
* Indicates that the Annual Percent Change (APC) is significantly different from zero at the alpha = 0.05 level.
Final Selected Model: 0 Joinpoints.

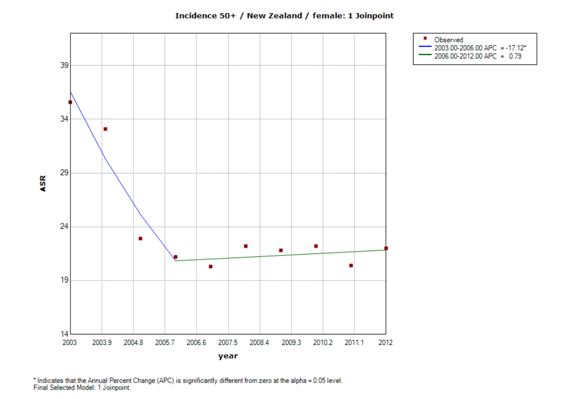
Incidence 50+ / New Zealand / female: 1 Joinpoint
Observed
2003.00-2006.00 APC = -17.12*
2006.00-2012.00 APC = 0.79
ASR
year
* Indicates that the Annual Percent Change (APC) is significantly different from zero at the alpha = 0.05 level.
Final Selected Model: 1 Joinpoint.

Northern America

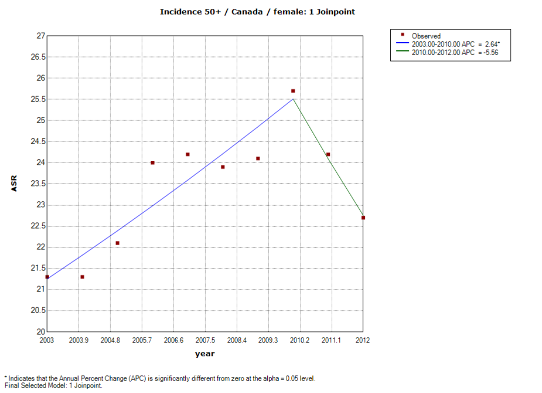

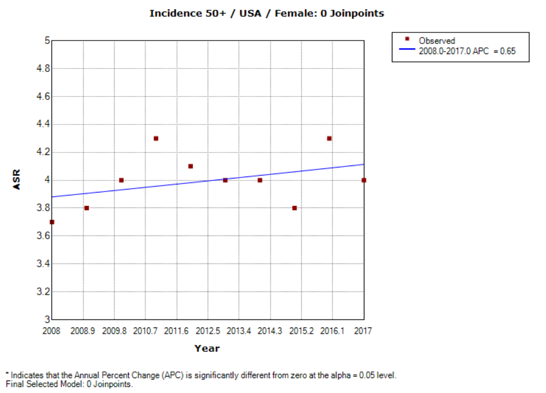

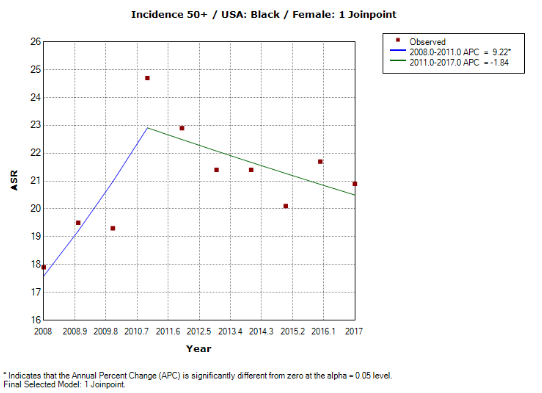

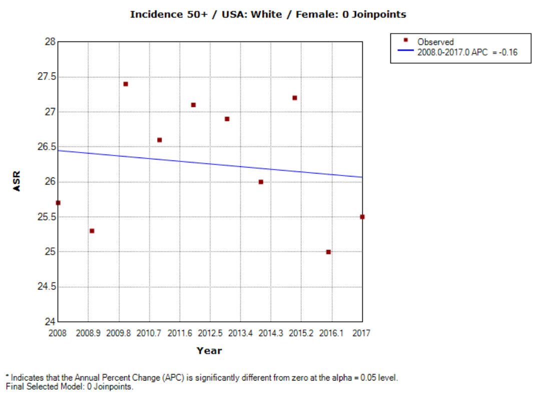

Southern America

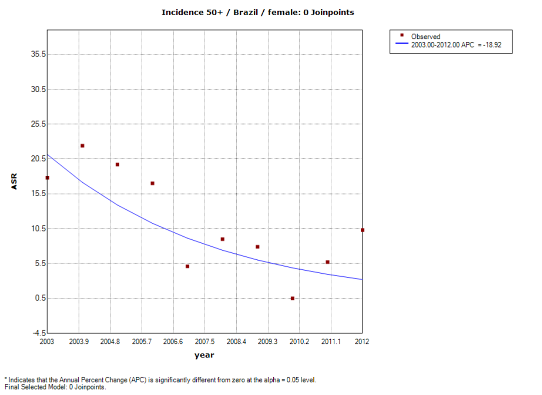

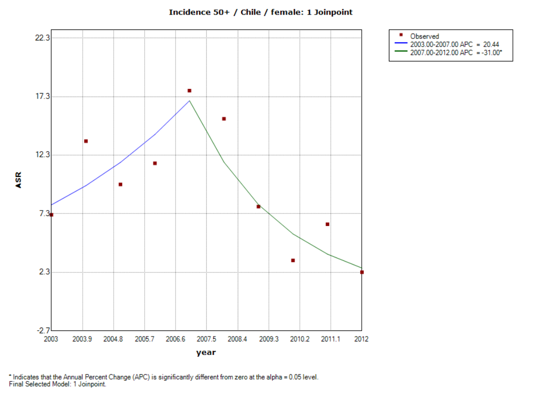

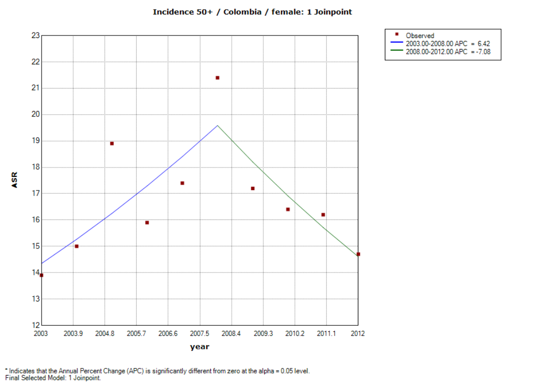

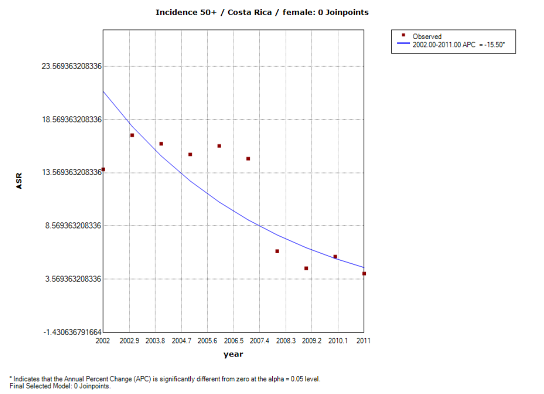

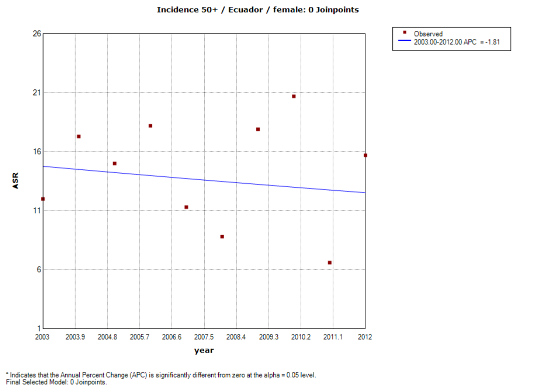

Northern Europe

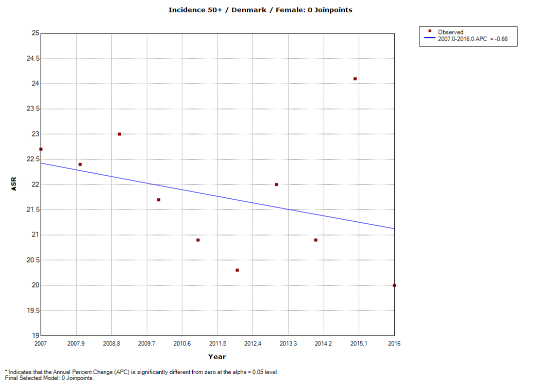

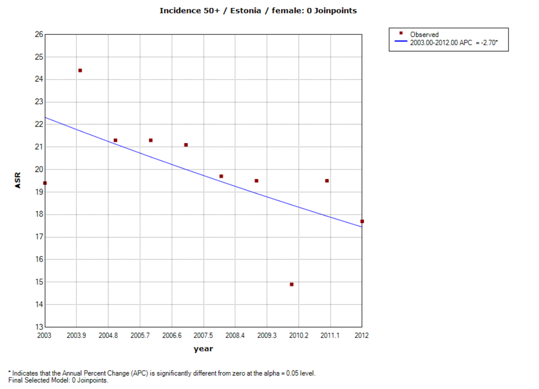

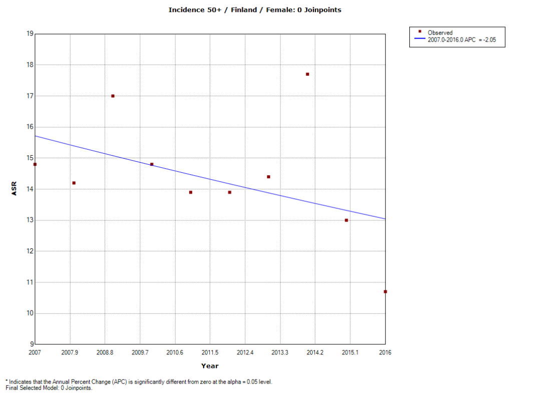

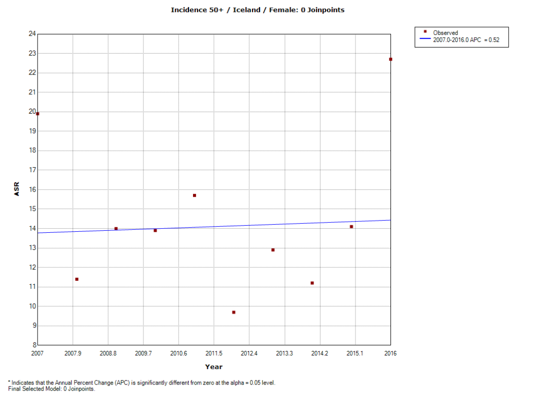

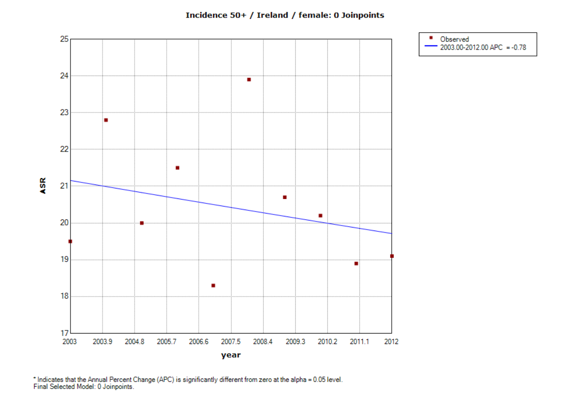

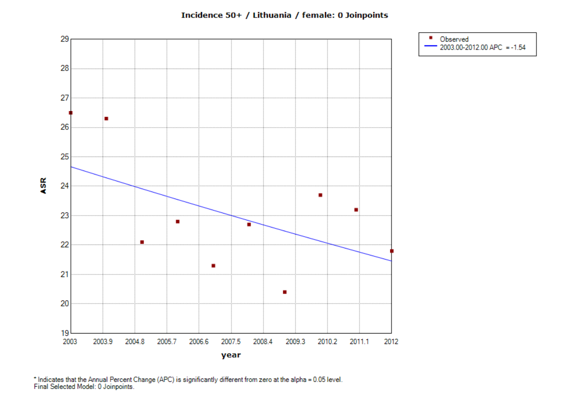

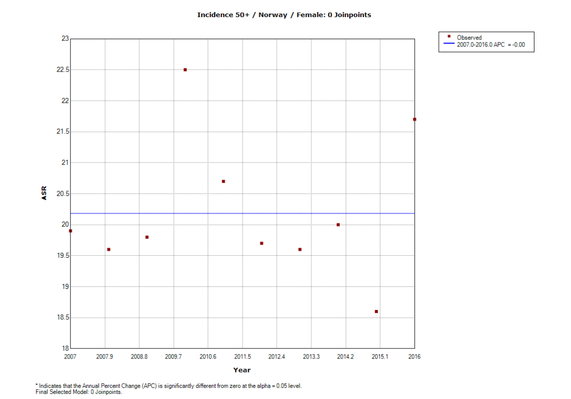

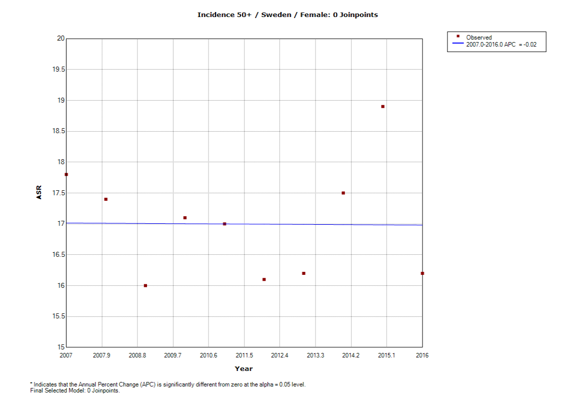

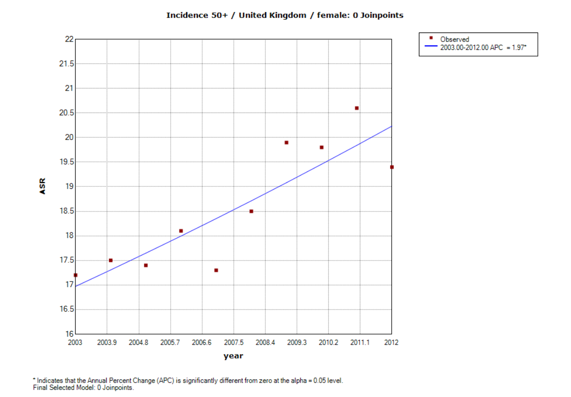

Western Europe

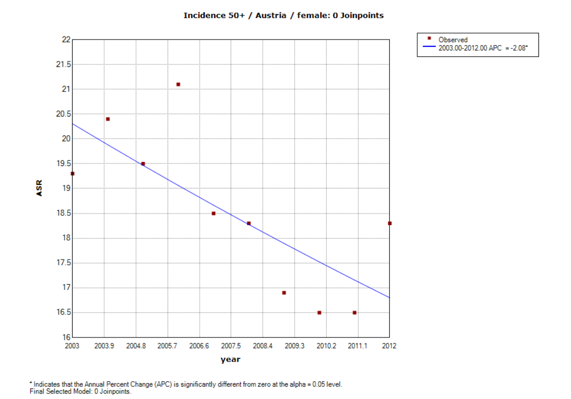

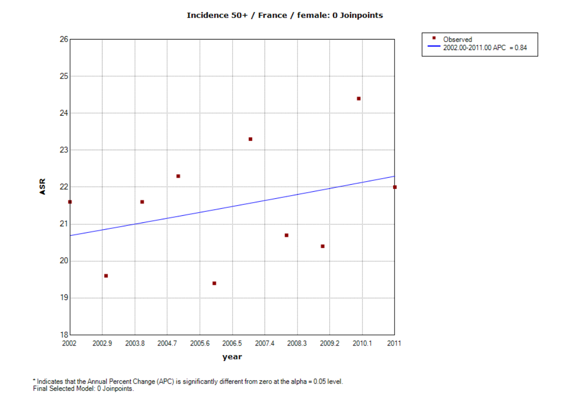

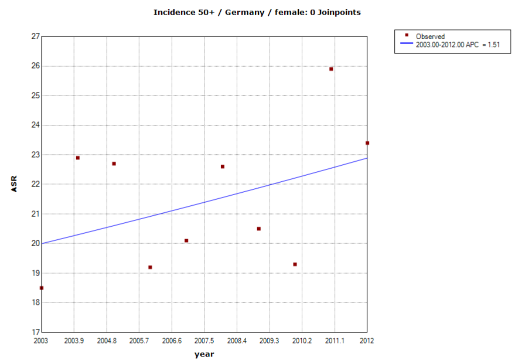

* Indicates that the Annual Percent Change (APC) is significantly different from zero at the alpha = 0.05 level.
Final Selected Model: 0 Joinpoints.

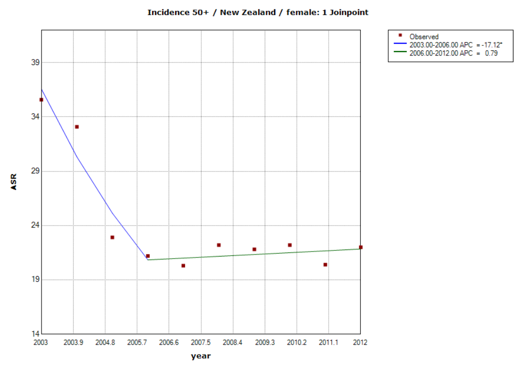

* Indicates that the Annual Percent Change (APC) is significantly different from zero at the alpha = 0.05 level.
Final Selected Model: 1 Joinpoint.

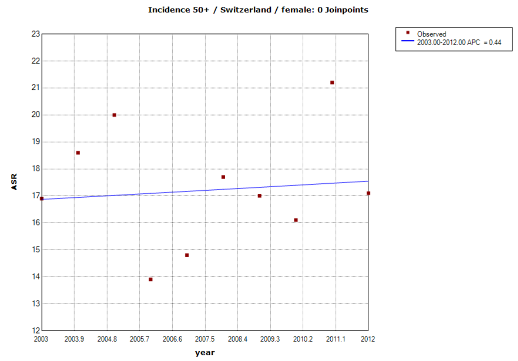

* Indicates that the Annual Percent Change (APC) is significantly different from zero at the alpha = 0.05 level.
Final Selected Model: 0 Joinpoints.

Southern Europe

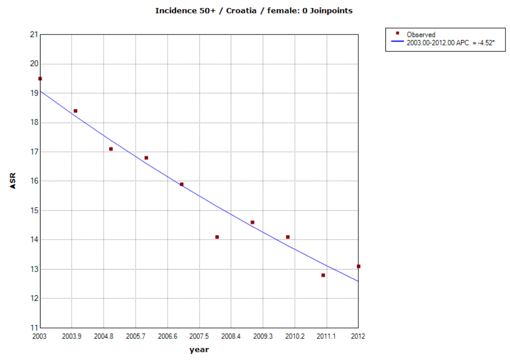

* Indicates that the Annual Percent Change (APC) is significantly different from zero at the alpha = 0.05 level.
Final Selected Model: 0 Joinpoints.

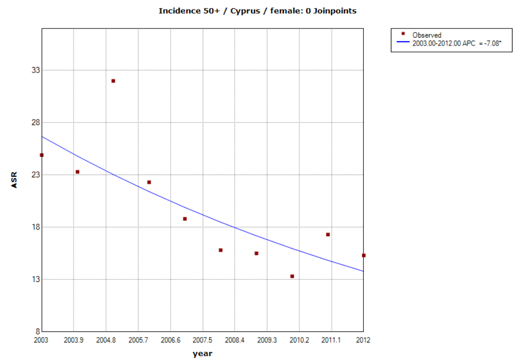

* Indicates that the Annual Percent Change (APC) is significantly different from zero at the alpha = 0.05 level.
Final Selected Model: 0 Joinpoints.

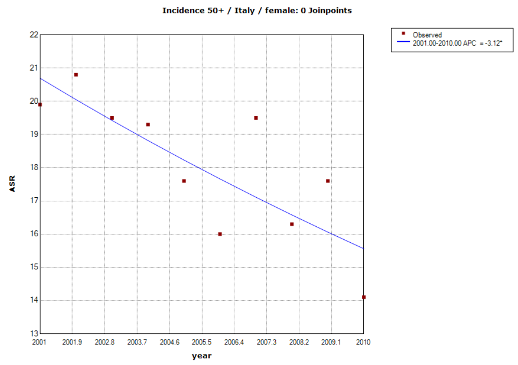

* Indicates that the Annual Percent Change (APC) is significantly different from zero at the alpha = 0.05 level.
Final Selected Model: 0 Joinpoints.

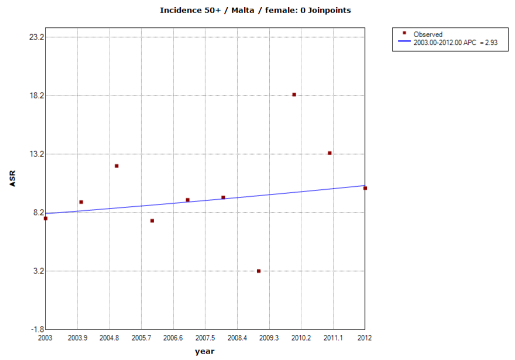

* Indicates that the Annual Percent Change (APC) is significantly different from zero at the alpha = 0.05 level.
Final Selected Model: 0 Joinpoints.

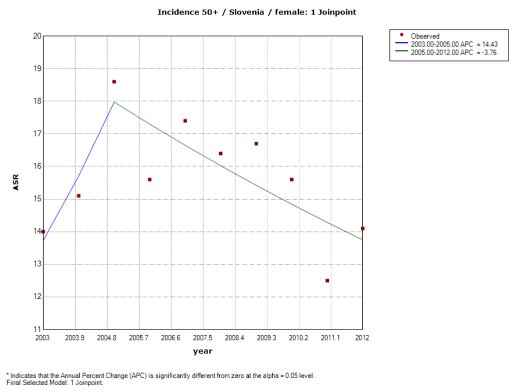

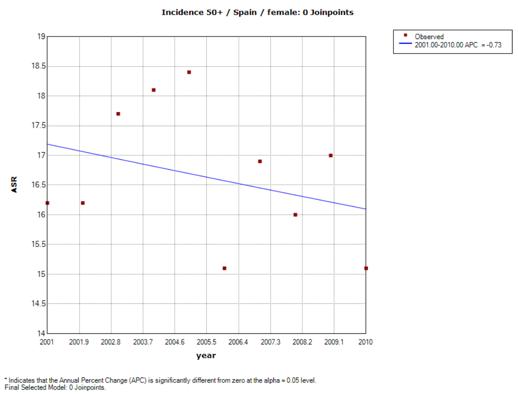

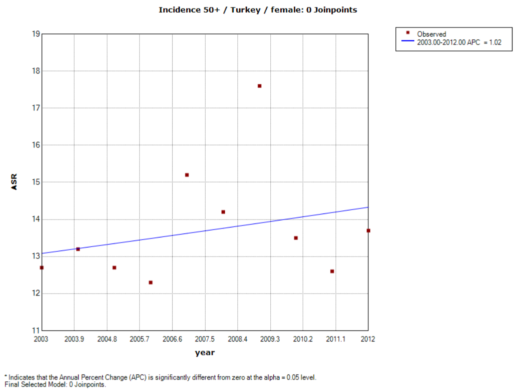

Eastern Europe

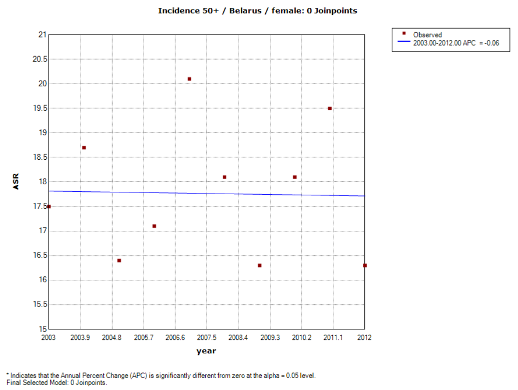

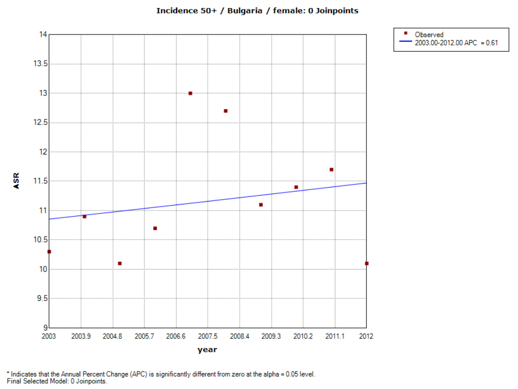

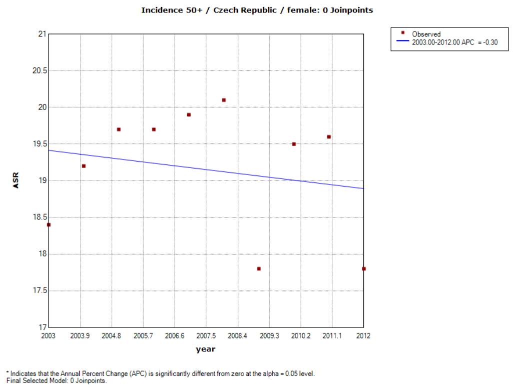

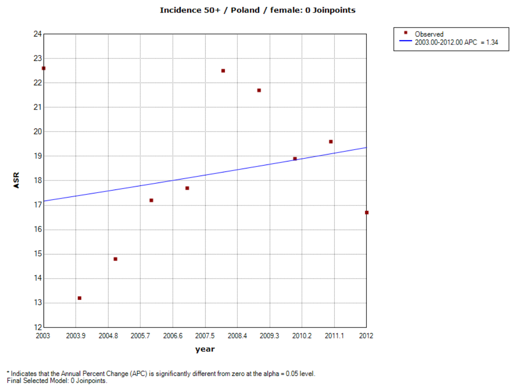

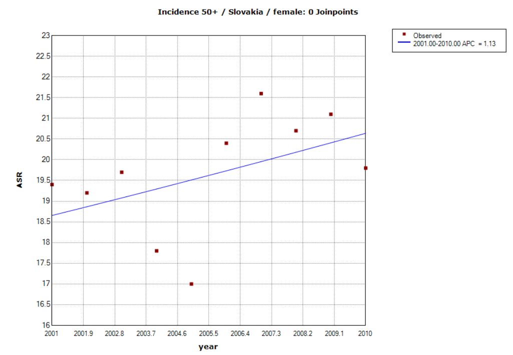

* Indicates that the Annual Percent Change (APC) is significantly different from zero at the alpha = 0.05 level.
Final Selected Model: 0 Joinpoints.

Africa

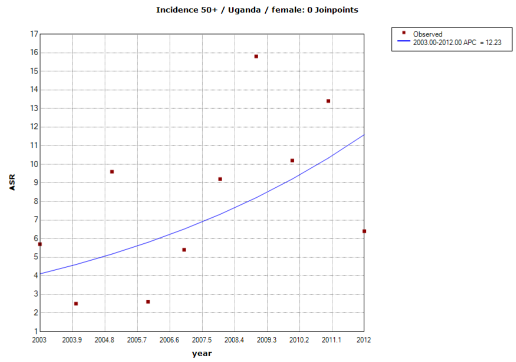

* Indicates that the Annual Percent Change (APC) is significantly different from zero at the alpha = 0.05 level.
Final Selected Model: 0 Joinpoints.

i.) Mortality male all ages

Asia

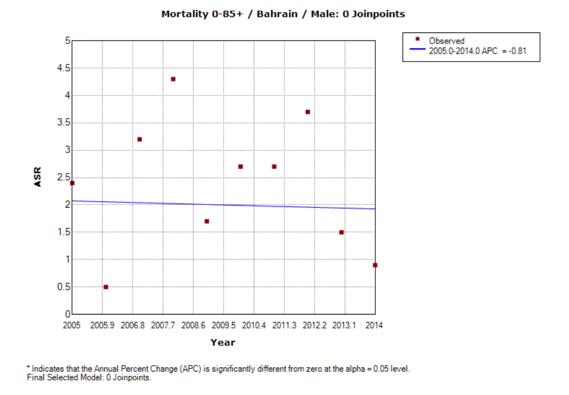

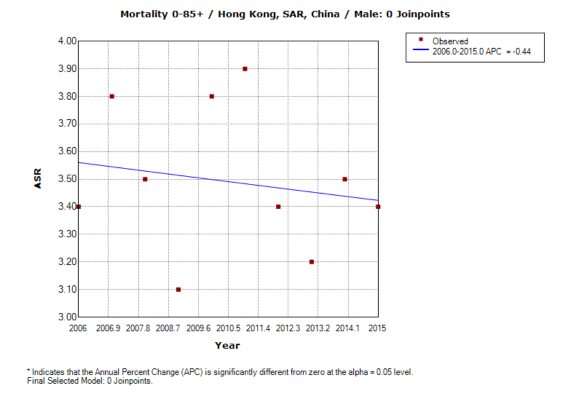

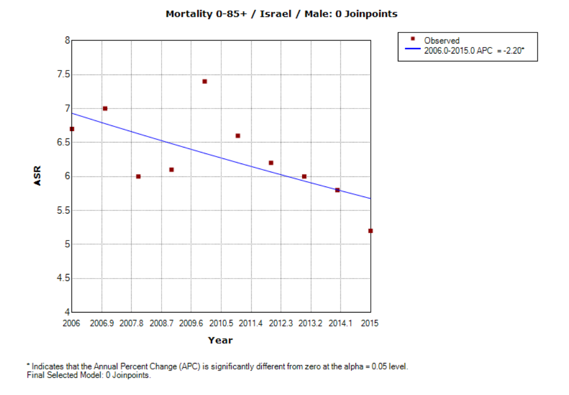

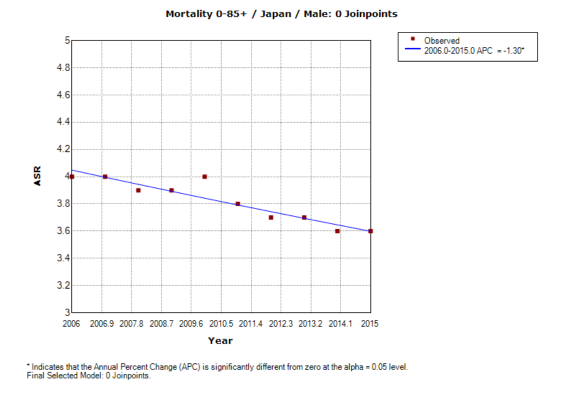

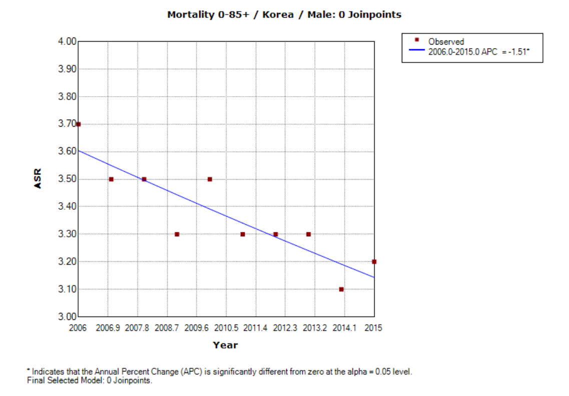

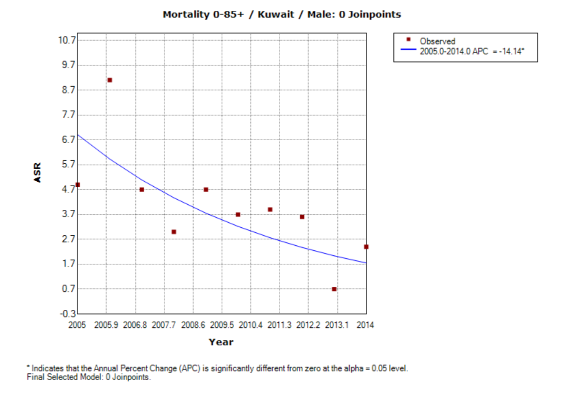

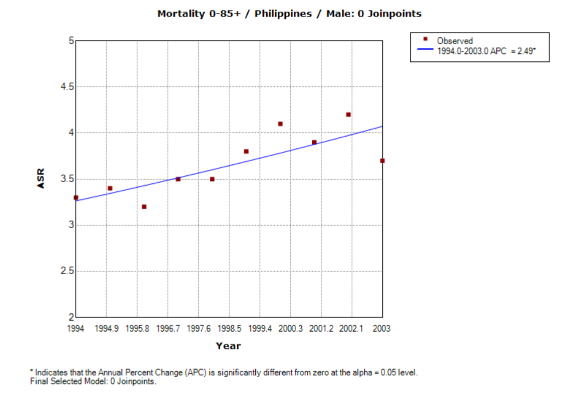
Mortality 0-85+ / Philippines / Male: 0 Joinpoints
Observed
1994.0-2003.0 APC = 2.49*
ASR
Year
* Indicates that the Annual Percent Change (APC) is significantly different from zero at the alpha = 0.05 level.
Final Selected Model: 0 Joinpoints.

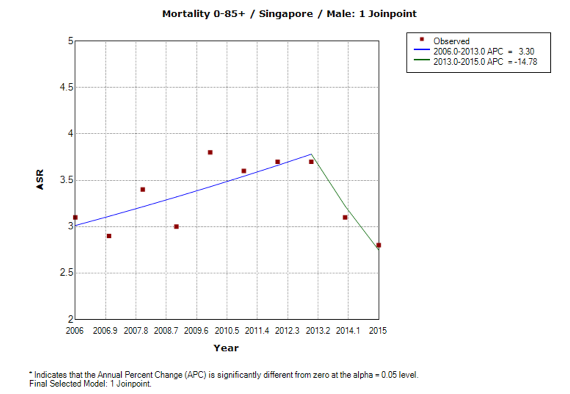
Mortality 0-85+ / Singapore / Male: 1 Joinpoint
Observed
2006.0-2013.0 APC = 3.30
2013.0-2015.0 APC = -14.78
ASR
Year
* Indicates that the Annual Percent Change (APC) is significantly different from zero at the alpha = 0.05 level.
Final Selected Model: 1 Joinpoint.

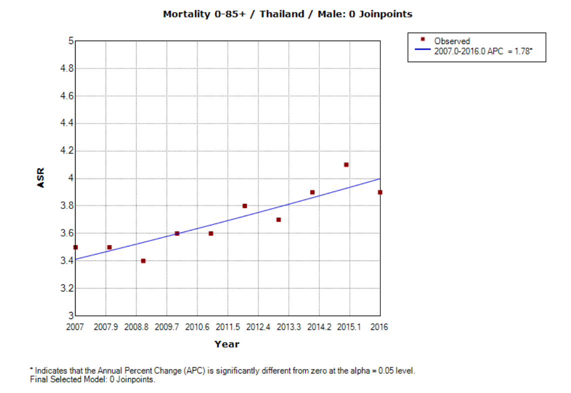
Mortality 0-85+ / Thailand / Male: 0 Joinpoints
Observed
2007.0-2016.0 APC = 1.78*
ASR
Year
* Indicates that the Annual Percent Change (APC) is significantly different from zero at the alpha = 0.05 level.
Final Selected Model: 0 Joinpoints.

Oceania

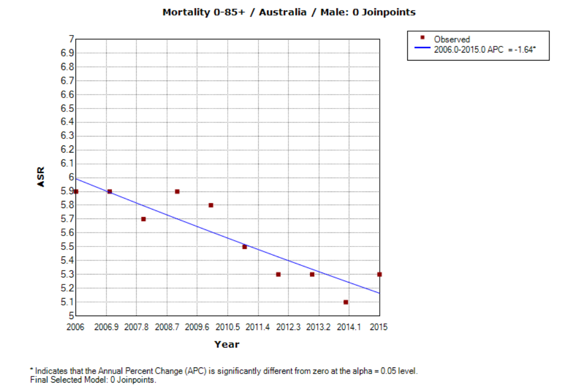
Mortality 0-85+ / Australia / Male: 0 Joinpoints
Observed
2006.0-2015.0 APC = -1.64*
ASR
Year
* Indicates that the Annual Percent Change (APC) is significantly different from zero at the alpha = 0.05 level.
Final Selected Model: 0 Joinpoints.

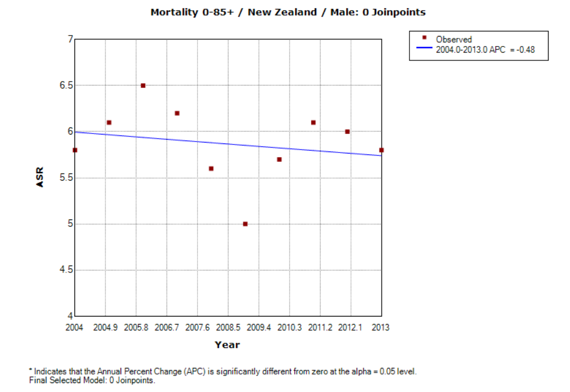
Mortality 0-85+ / New Zealand / Male: 0 Joinpoints
Observed
2004.0-2013.0 APC = -0.48
ASR
Year
* Indicates that the Annual Percent Change (APC) is significantly different from zero at the alpha = 0.05 level.
Final Selected Model: 0 Joinpoints.

Northern America

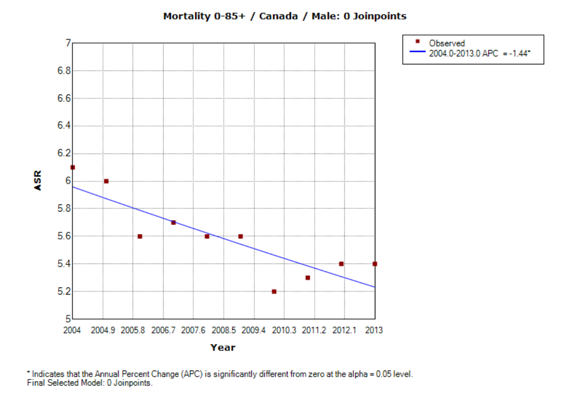

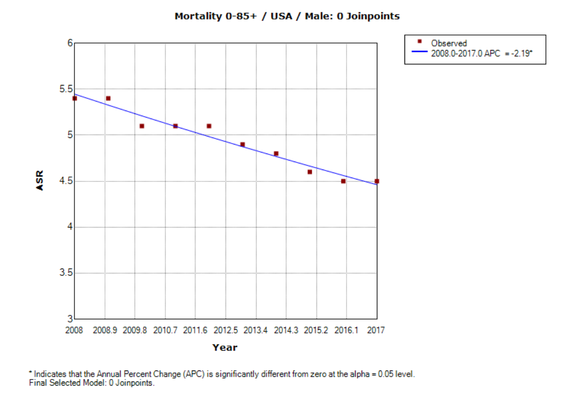

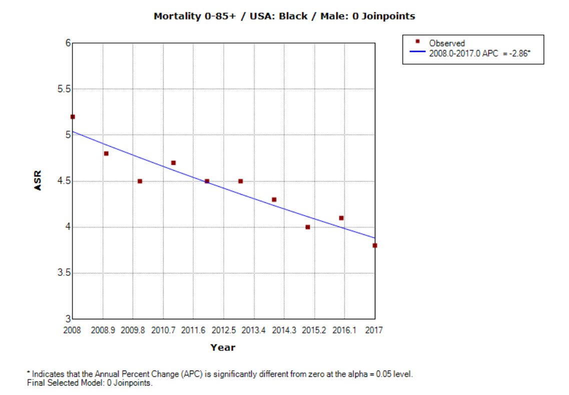

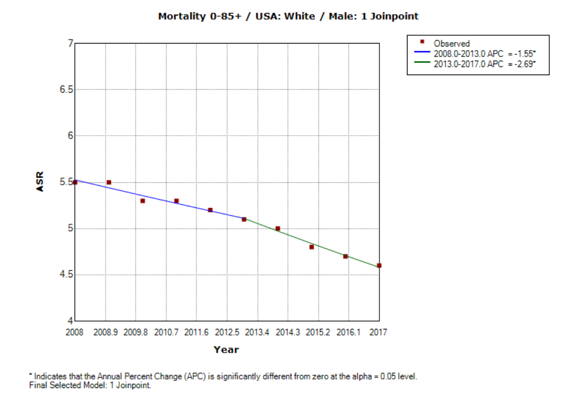

Southern America

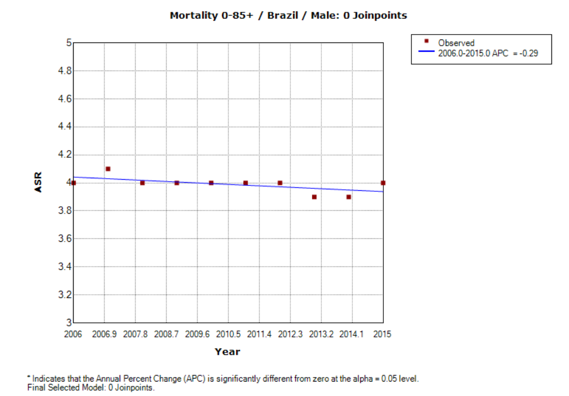

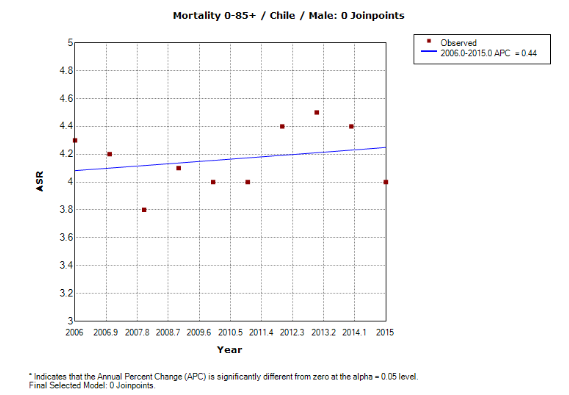

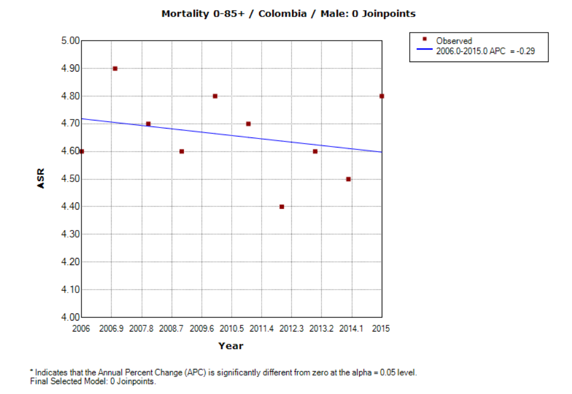

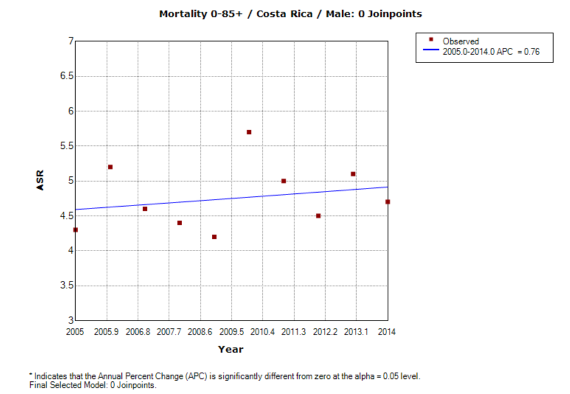

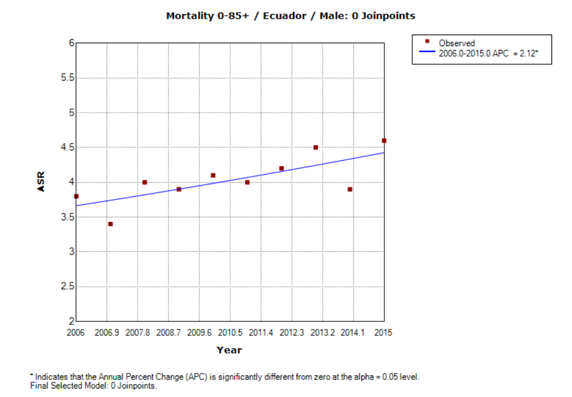

Northern Europe

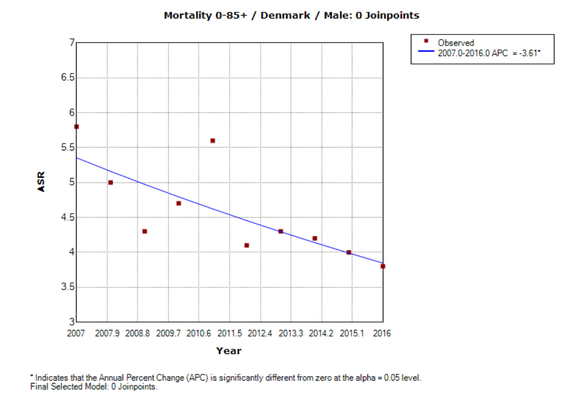

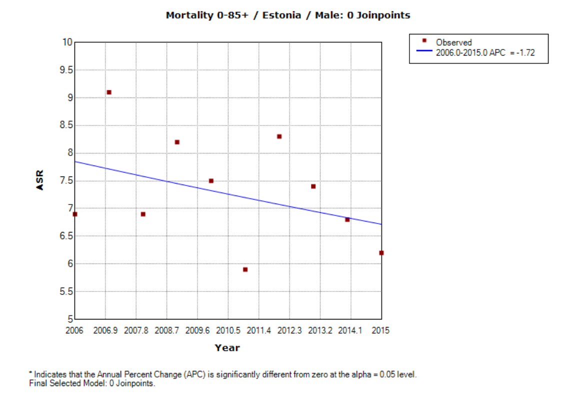

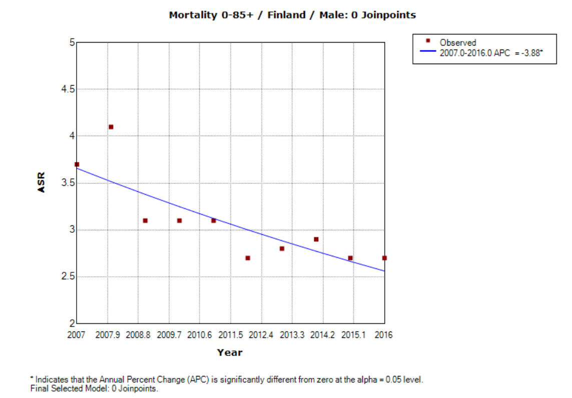

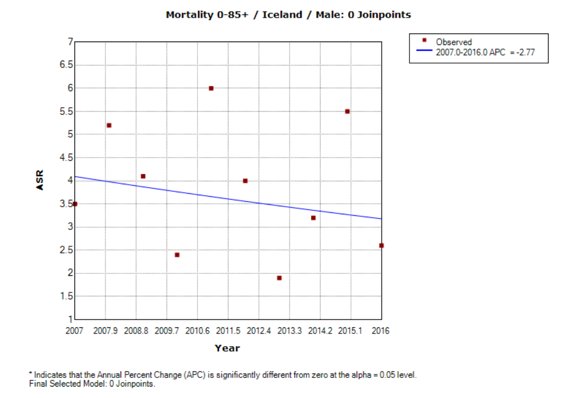

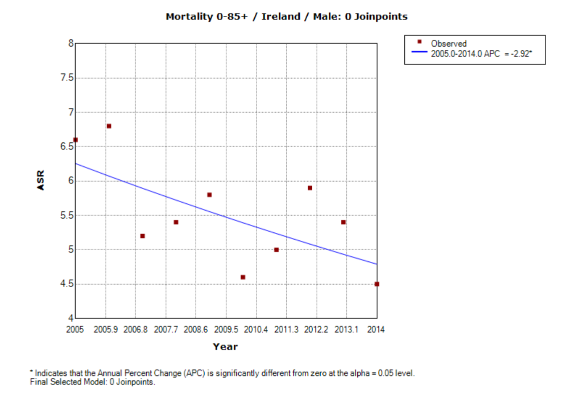

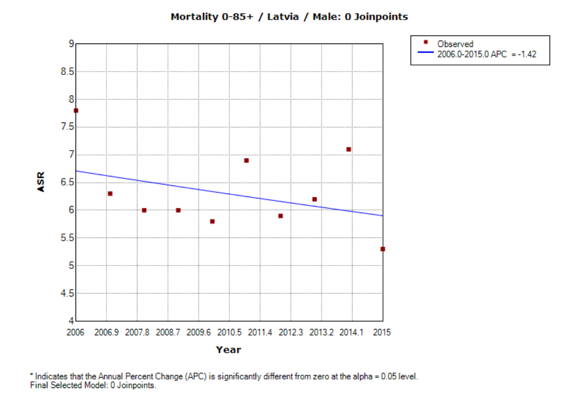

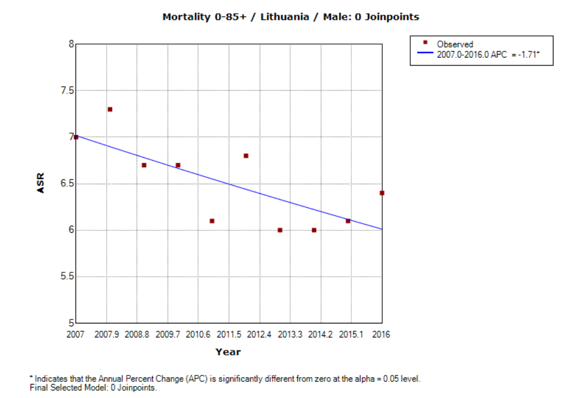

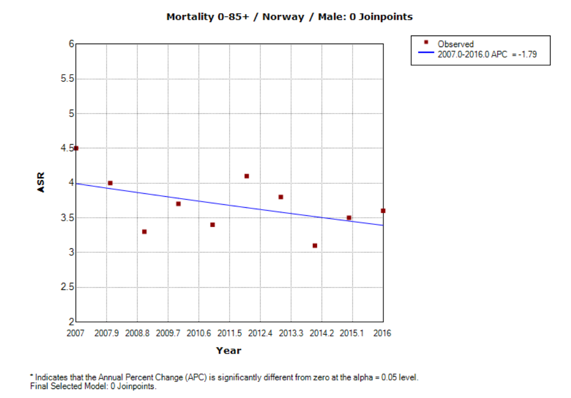

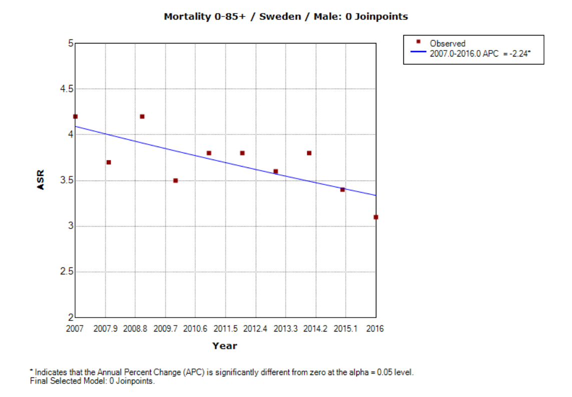

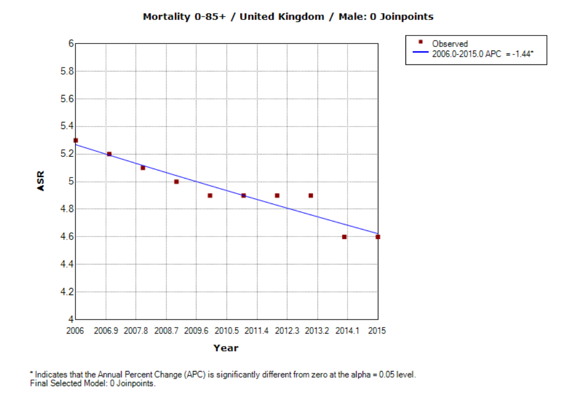

Western Europe

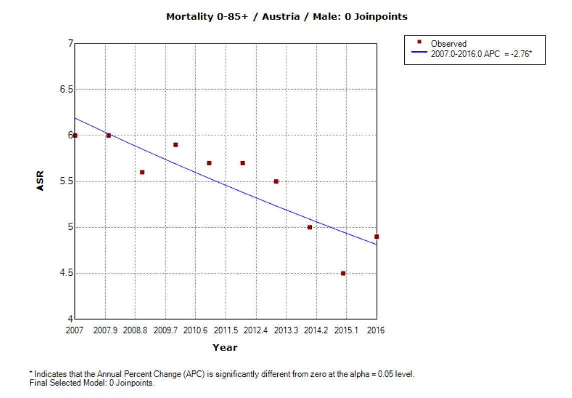

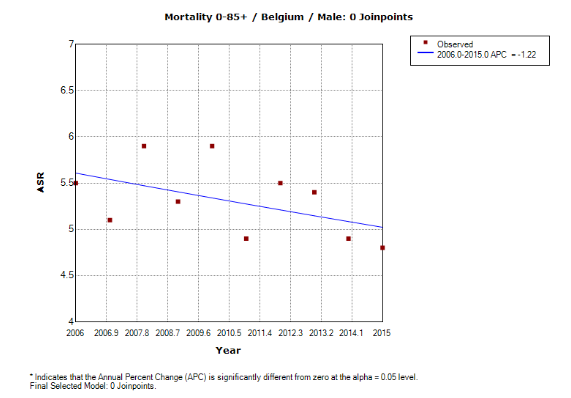

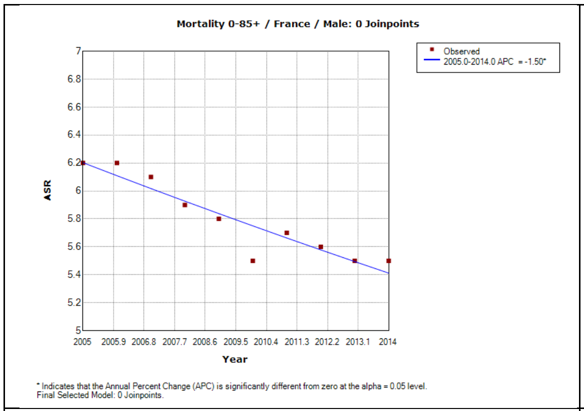

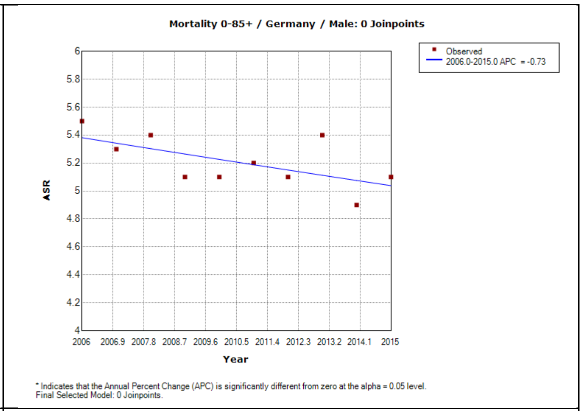

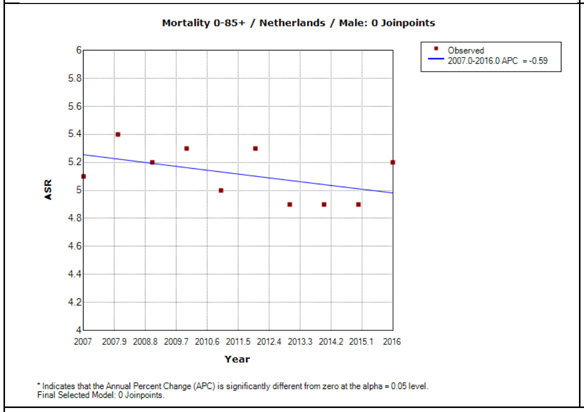

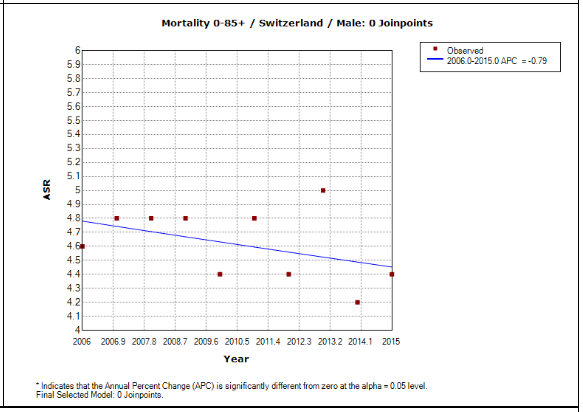

Southern Europe

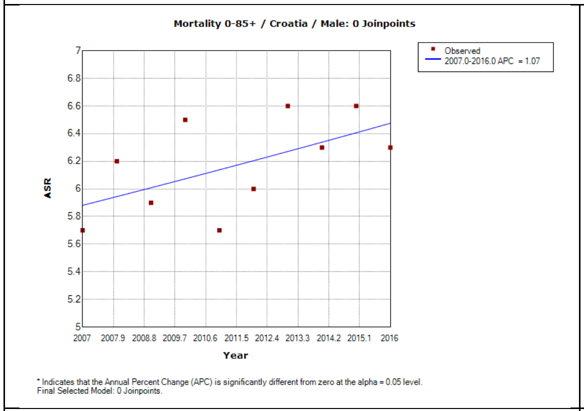

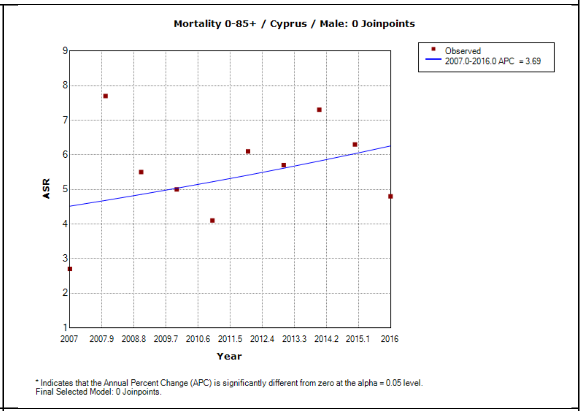

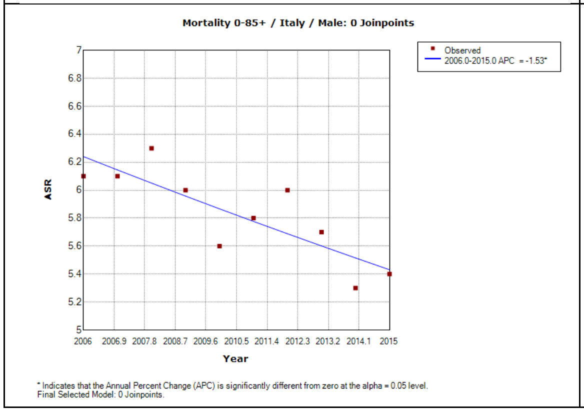

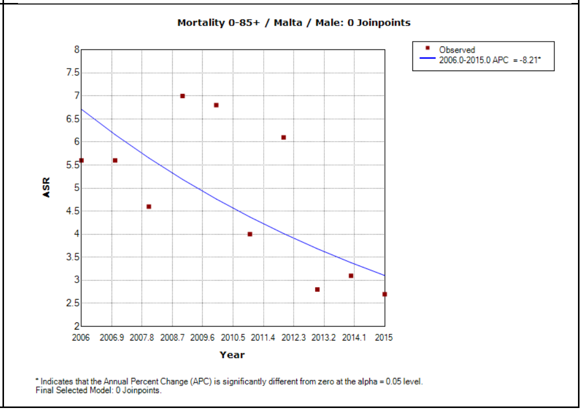

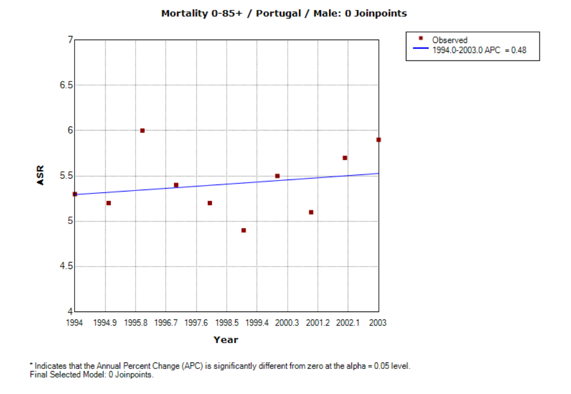

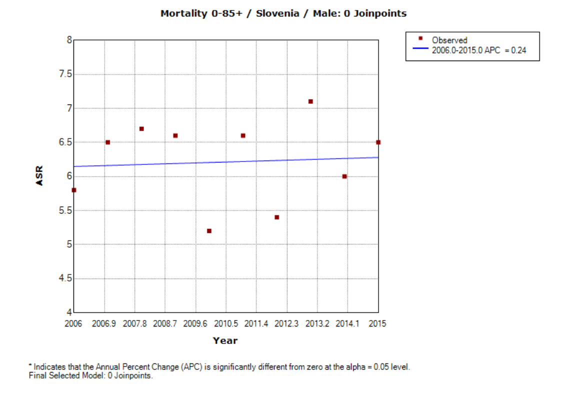

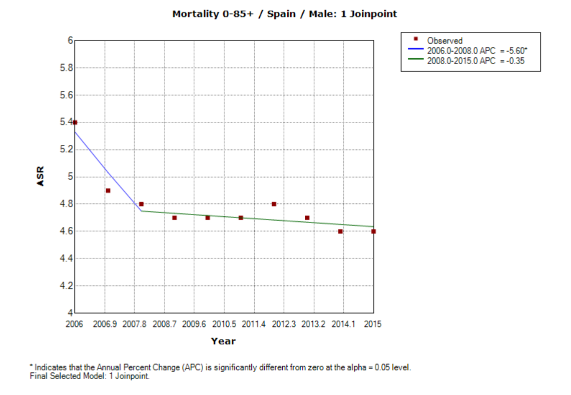

Eastern Europe

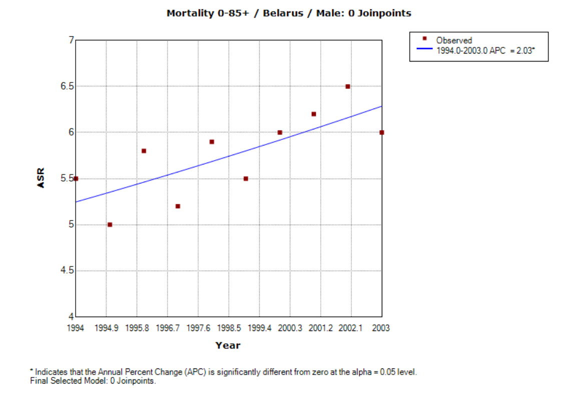

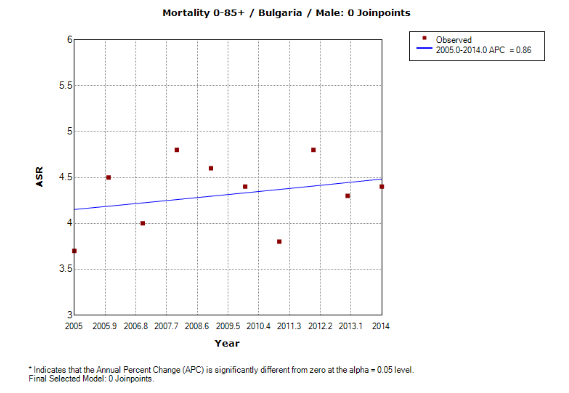

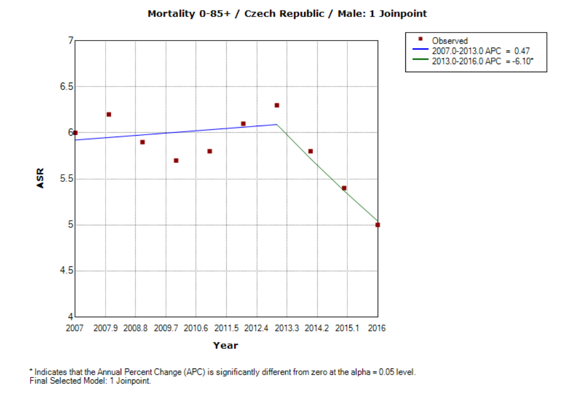

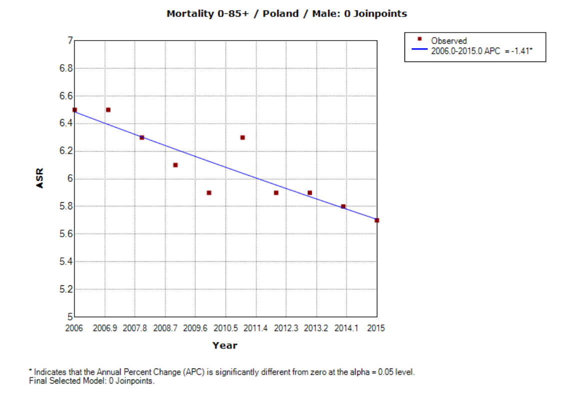

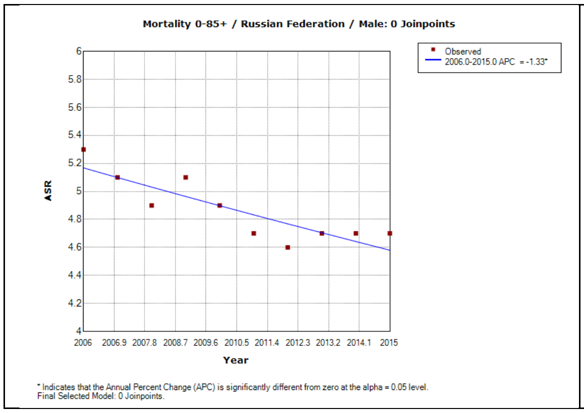
Mortality 0-85+ / Russian Federation / Male: 0 Joinpoints
Observed
2006.0-2015.0 APC = -1.33*
ASR
6
5.8
5.6
5.4
5.2
5
4.8
4.6
4.4
4.2
4
2006
2006.9
2007.8
2008.7
2009.6
2010.5
2011.4
2012.3
2013.2
2014.1
2015
Year
* Indicates that the Annual Percent Change (APC) is significantly different from zero at the alpha = 0.05 level.
Final Selected Model: 0 Joinpoints.

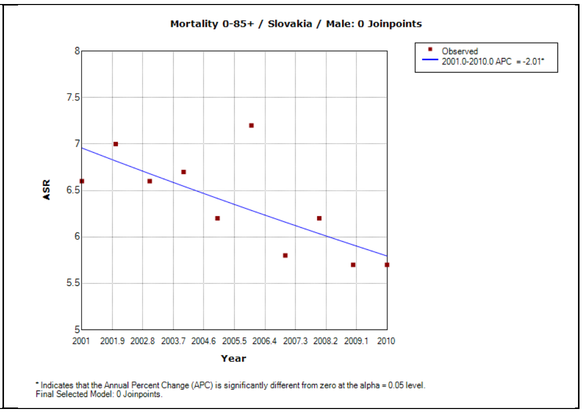
Mortality 0-85+ / Slovakia / Male: 0 Joinpoints
Observed
2001.0-2010.0 APC = -2.01*
ASR
8
7.5
7
6.5
6
5.5
5
2001
2001.9
2002.8
2003.7
2004.6
2005.5
2006.4
2007.3
2008.2
2009.1
2010
Year
* Indicates that the Annual Percent Change (APC) is significantly different from zero at the alpha = 0.05 level.
Final Selected Model: 0 Joinpoints.

j.) Mortality female all ages

Asia

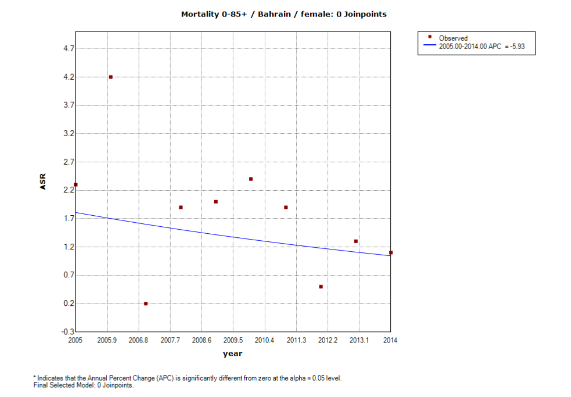

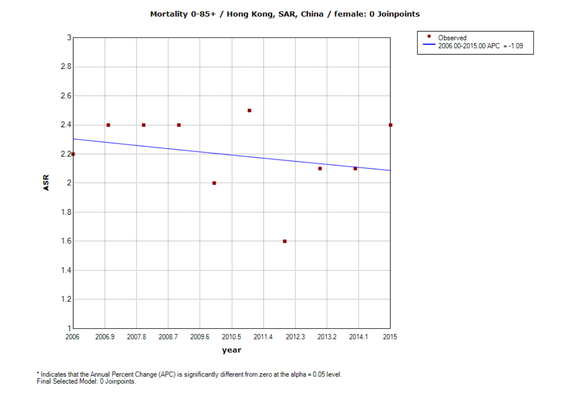

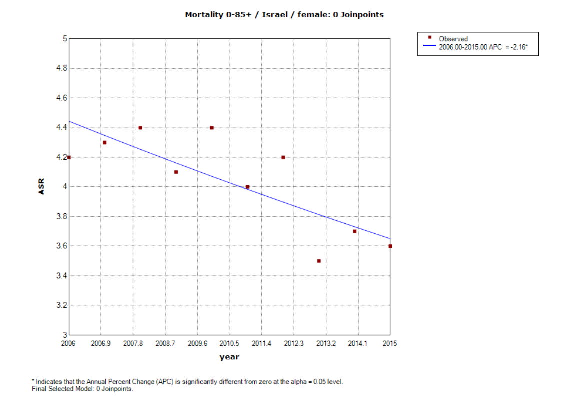

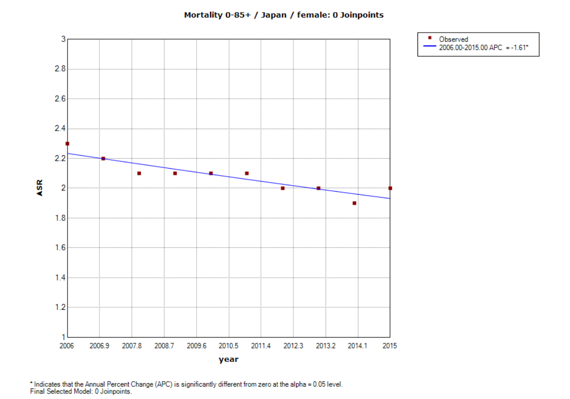

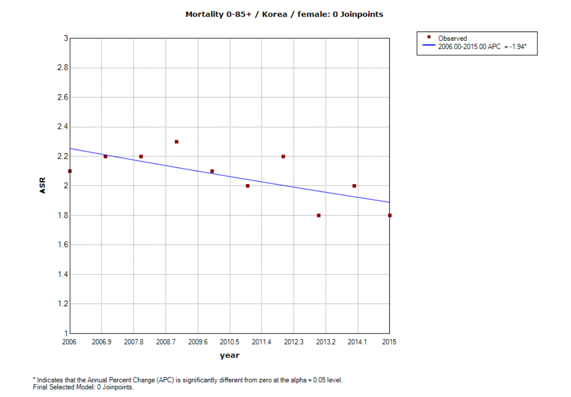

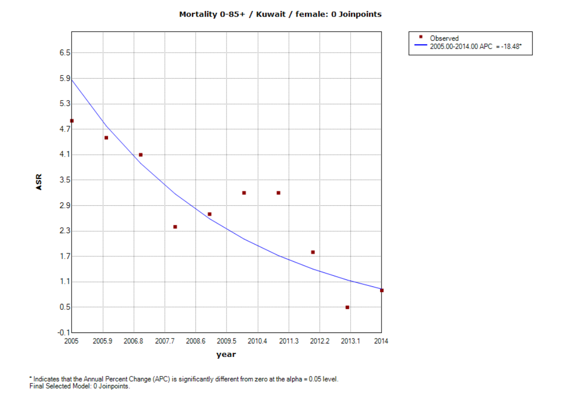

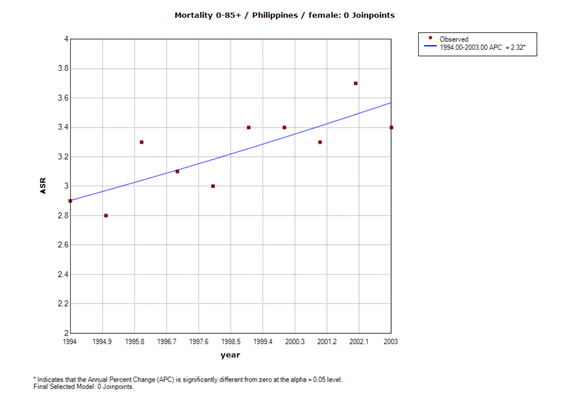
Mortality 0-85+ / Philippines / female: 0 Joinpoints
Observed
1994.00-2003.00 APC = 2.32*
ASR
year
* Indicates that the Annual Percent Change (APC) is significantly different from zero at the alpha = 0.05 level.
Final Selected Model: 0 Joinpoints.

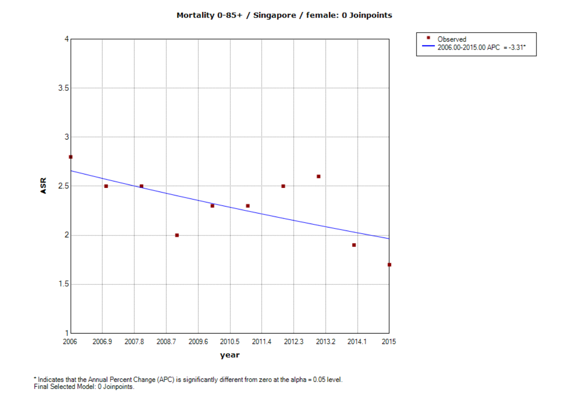
Mortality 0-85+ / Singapore / female: 0 Joinpoints
Observed
2006.00-2015.00 APC = -3.31*
ASR
year
* Indicates that the Annual Percent Change (APC) is significantly different from zero at the alpha = 0.05 level.
Final Selected Model: 0 Joinpoints.

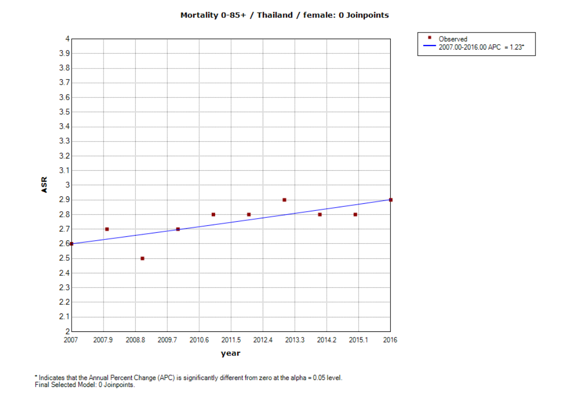
Mortality 0-85+ / Thailand / female: 0 Joinpoints
Observed
2007.00-2016.00 APC = 1.23*
ASR
year
* Indicates that the Annual Percent Change (APC) is significantly different from zero at the alpha = 0.05 level.
Final Selected Model: 0 Joinpoints.

Oceania

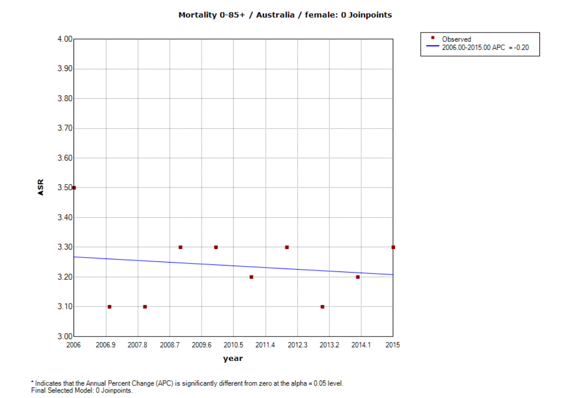
Mortality 0-85+ / Australia / female: 0 Joinpoints
Observed
2006.00-2015.00 APC = -0.20
ASR
year
* Indicates that the Annual Percent Change (APC) is significantly different from zero at the alpha = 0.05 level.
Final Selected Model: 0 Joinpoints.

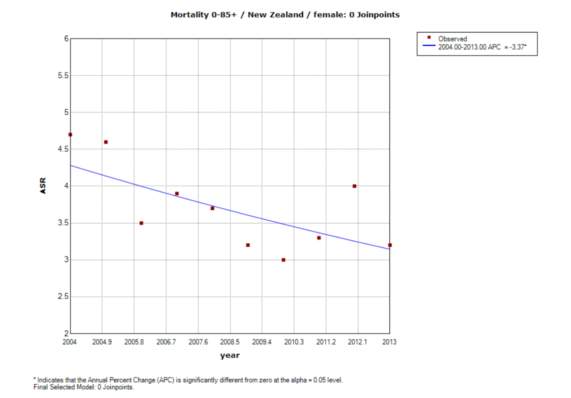
Mortality 0-85+ / New Zealand / female: 0 Joinpoints
Observed
2004.00-2013.00 APC = -3.37*
ASR
year
* Indicates that the Annual Percent Change (APC) is significantly different from zero at the alpha = 0.05 level.
Final Selected Model: 0 Joinpoints.

Northern America

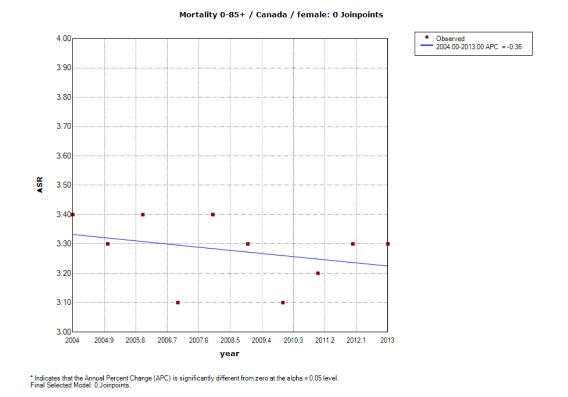

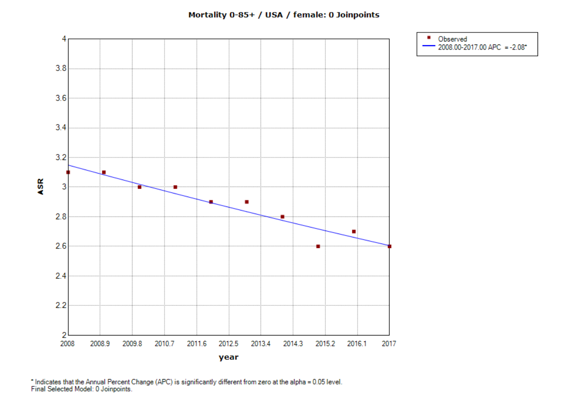

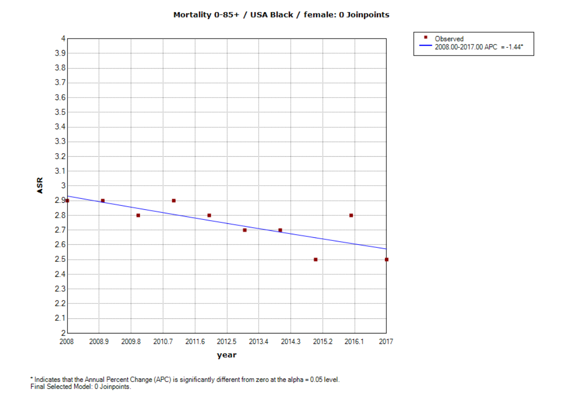

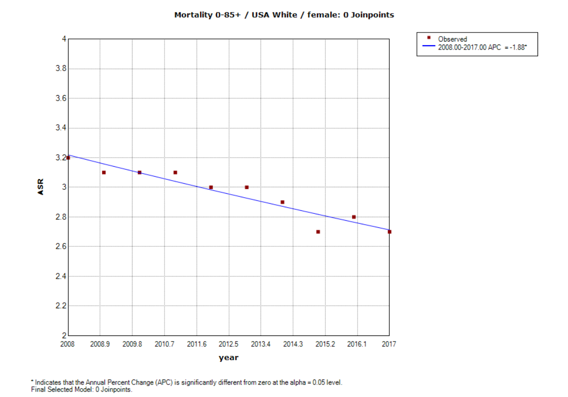

Southern America

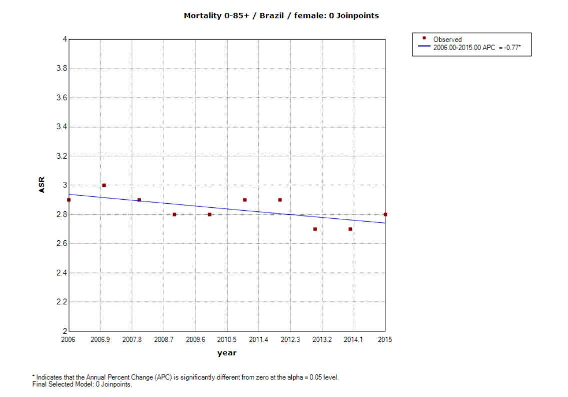

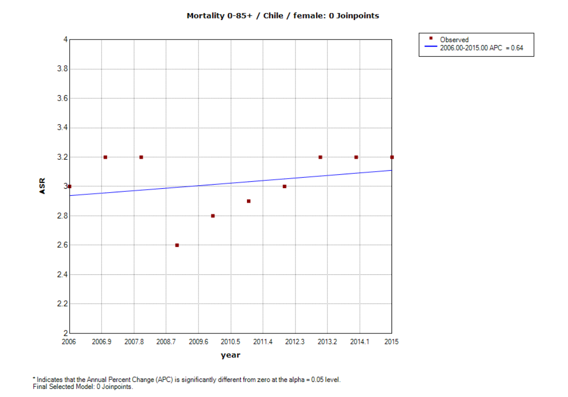

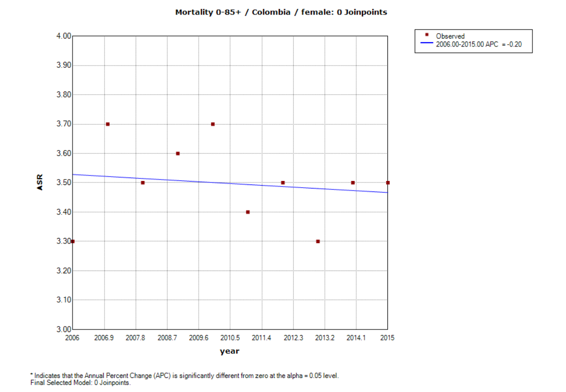

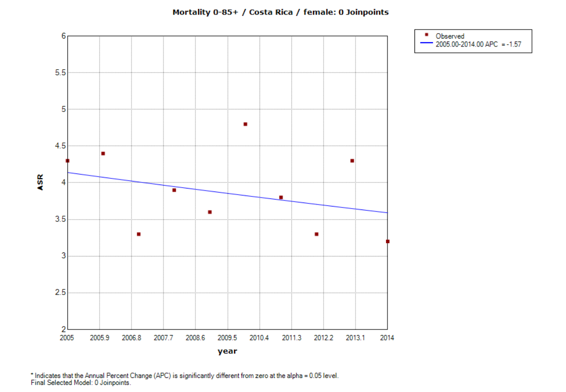

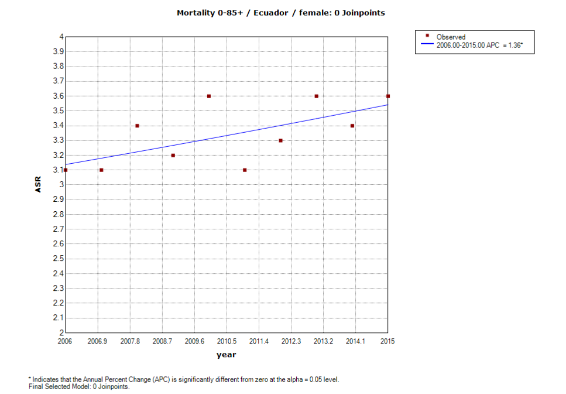

Northern Europe

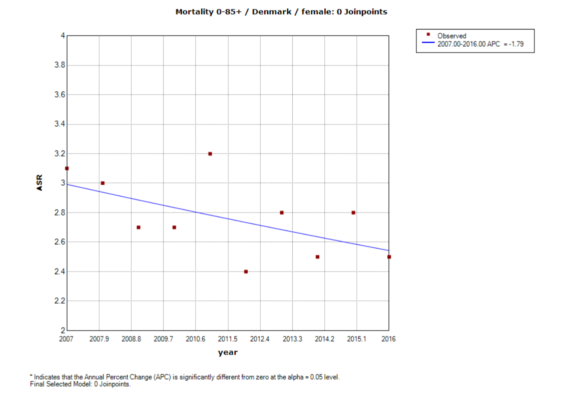

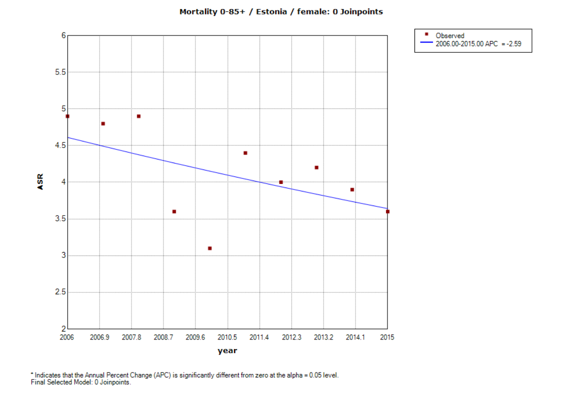

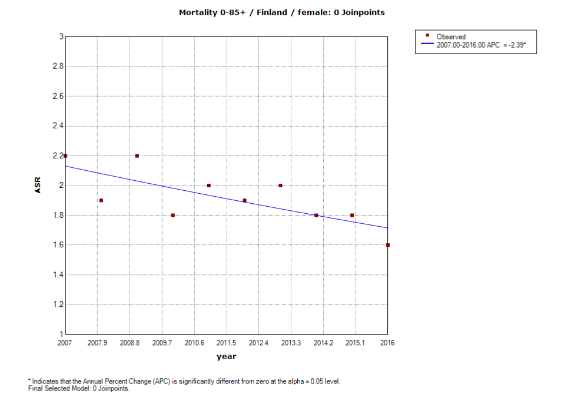

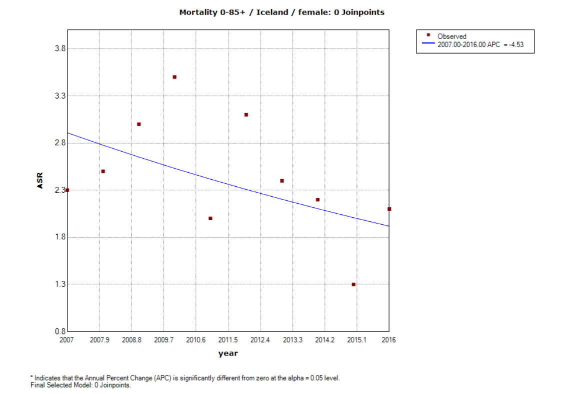

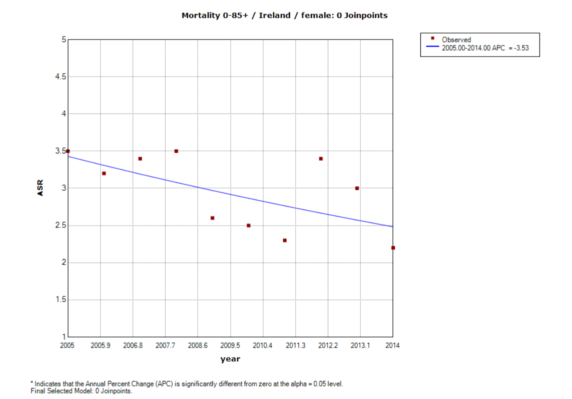

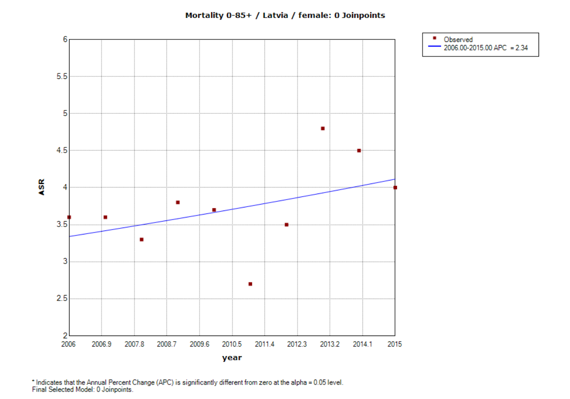

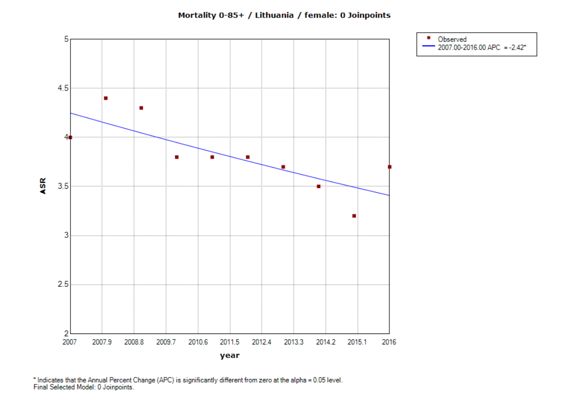

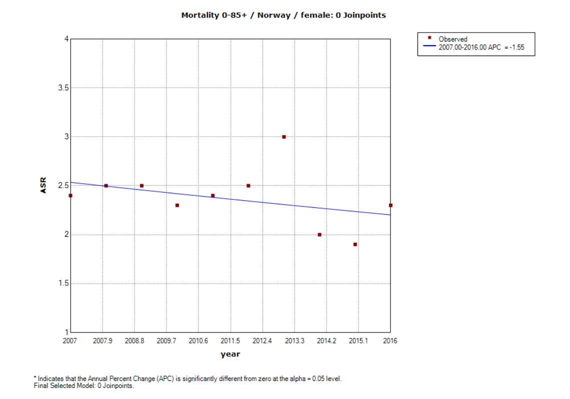

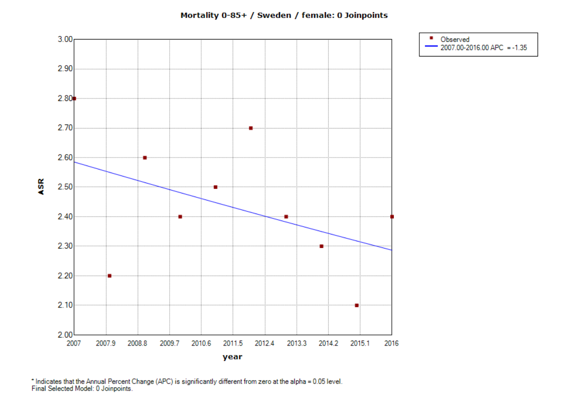

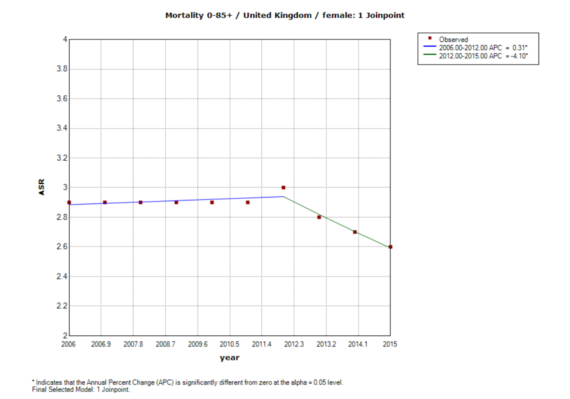

Western Europe

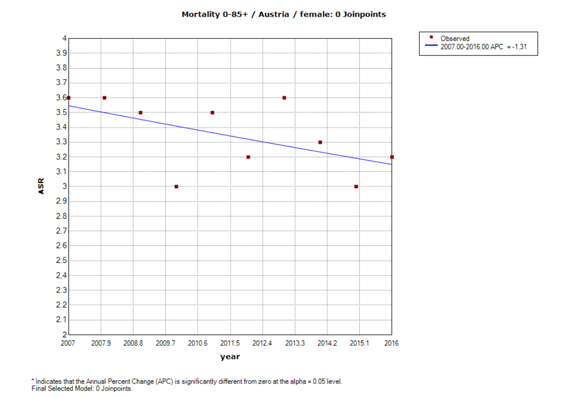

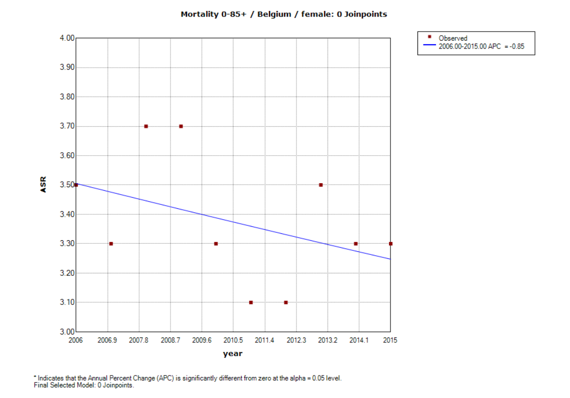

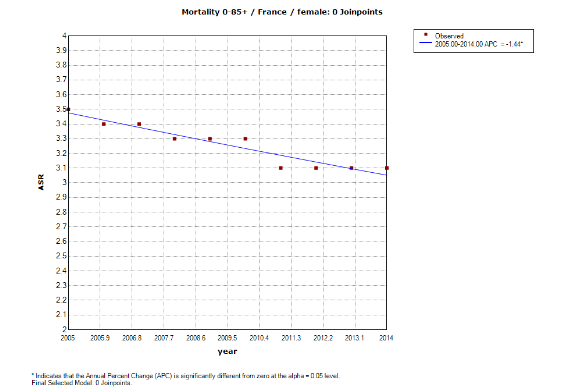

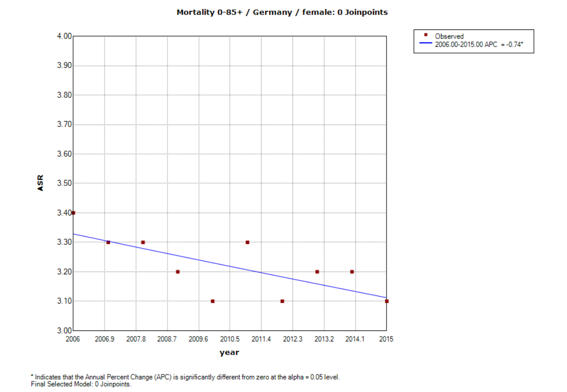

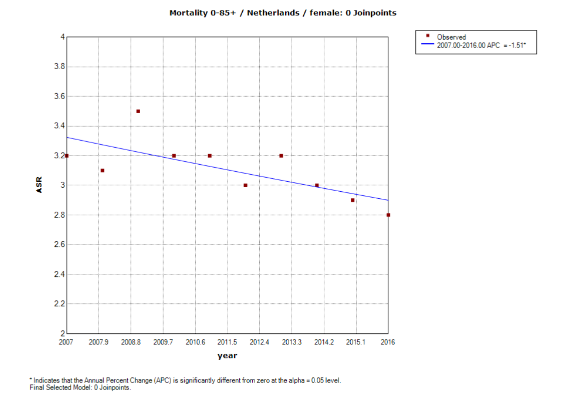

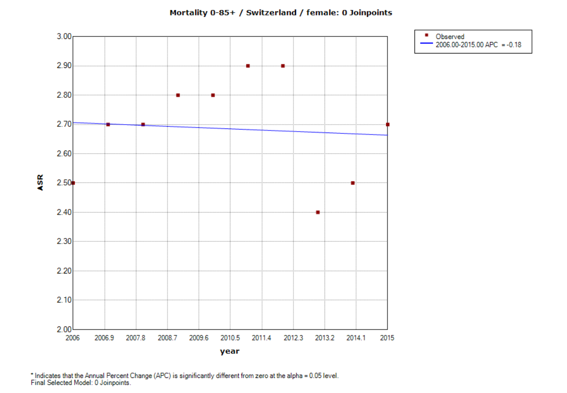

Southern Europe

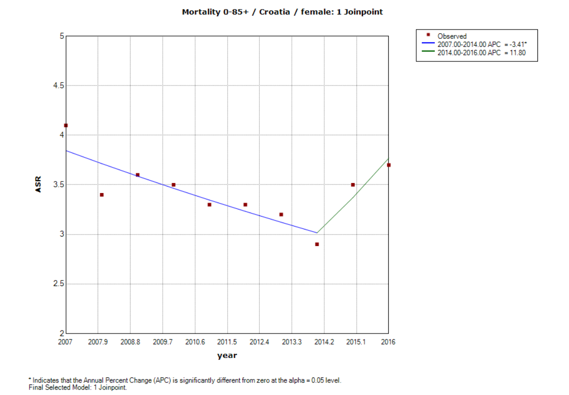

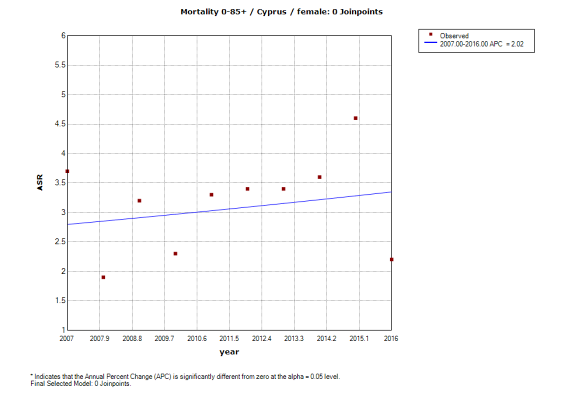

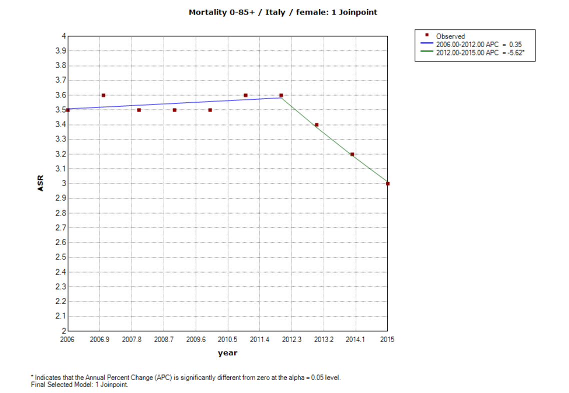

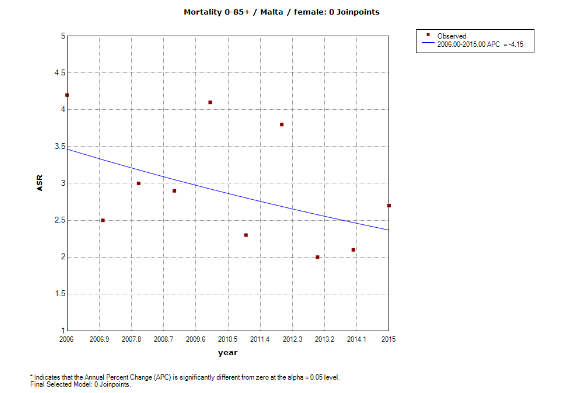

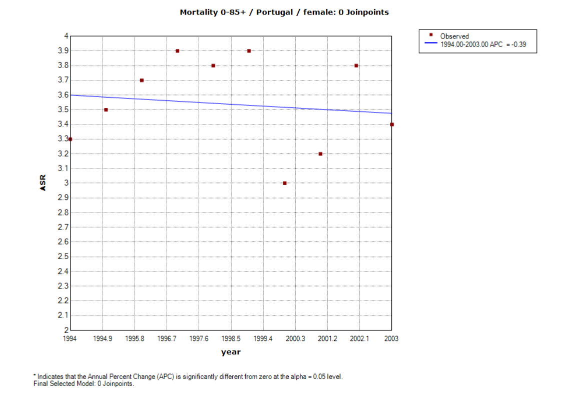

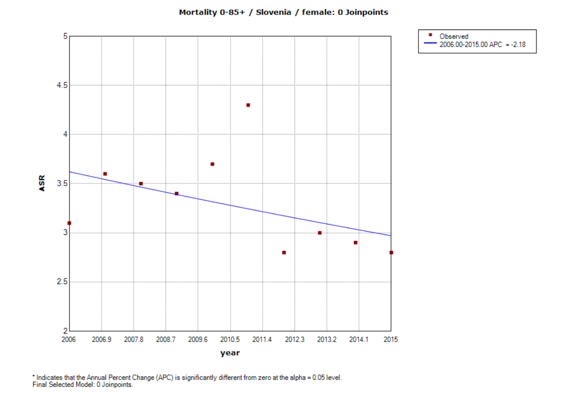

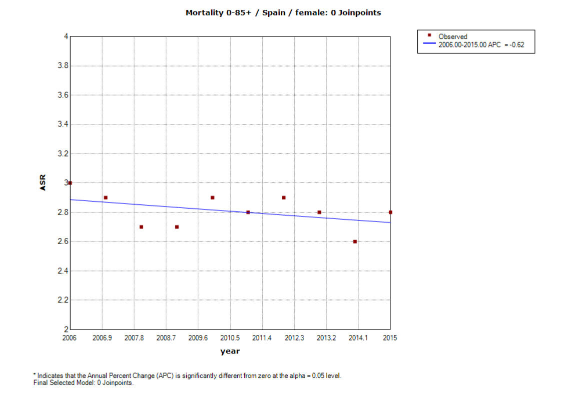

Eastern Europe

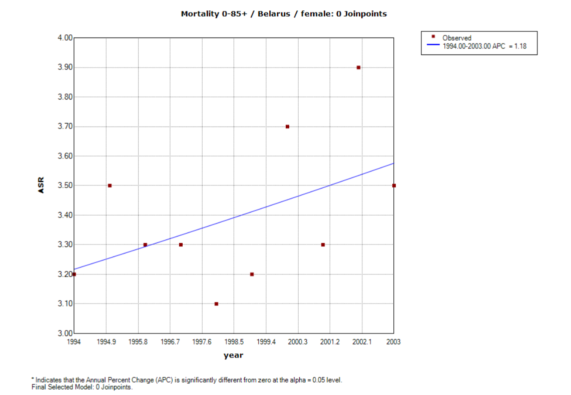

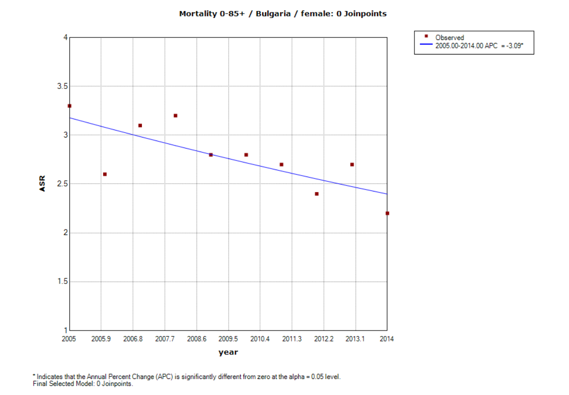

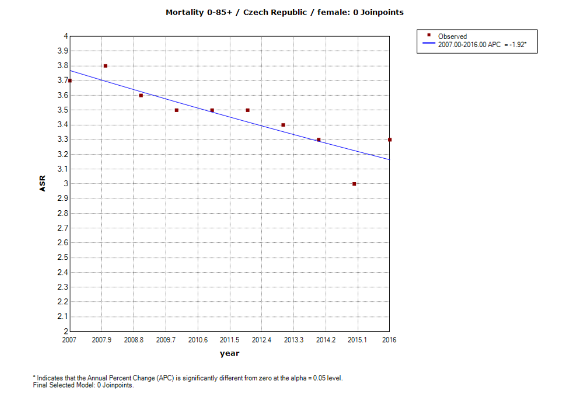

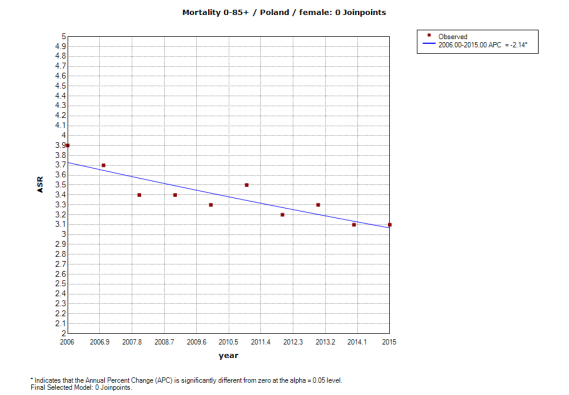

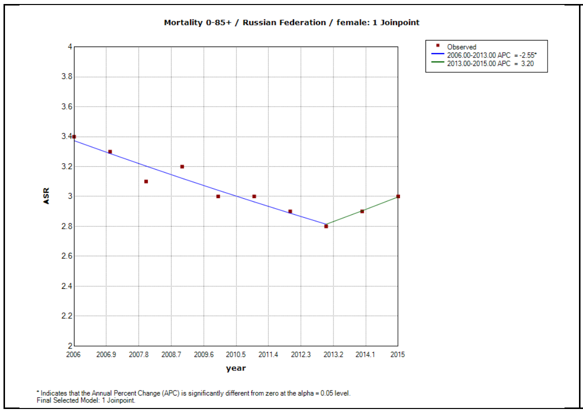
Mortality 0-85+ / Russian Federation / female: 1 Joinpoint
Observed
2006.00-2013.00 APC = -2.55*
2013.00-2015.00 APC = 3.20
ASR
4
3.8
3.6
3.4
3.2
3
2.8
2.6
2.4
2.2
2
2006
2006.9
2007.8
2008.7
2009.6
2010.5
2011.4
2012.3
2013.2
2014.1
2015
year
* Indicates that the Annual Percent Change (APC) is significantly different from zero at the alpha = 0.05 level.
Final Selected Model: 1 Joinpoint.

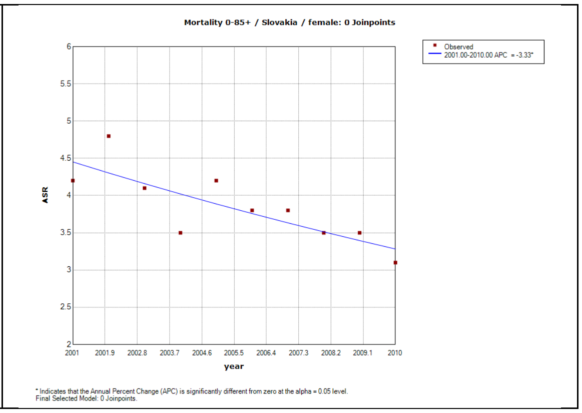
Mortality 0-85+ / Slovakia / female: 0 Joinpoints
Observed
2001.00-2010.00 APC = -3.33*
ASR
6
5.5
5
4.5
4
3.5
3
2.5
2
2001
2001.9
2002.8
2003.7
2004.6
2005.5
2006.4
2007.3
2008.2
2009.1
2010
year
* Indicates that the Annual Percent Change (APC) is significantly different from zero at the alpha = 0.05 level.
Final Selected Model: 0 Joinpoints.

Supplementary Figure 3. AAPC of incidence of leukaemia aged 50 years and older

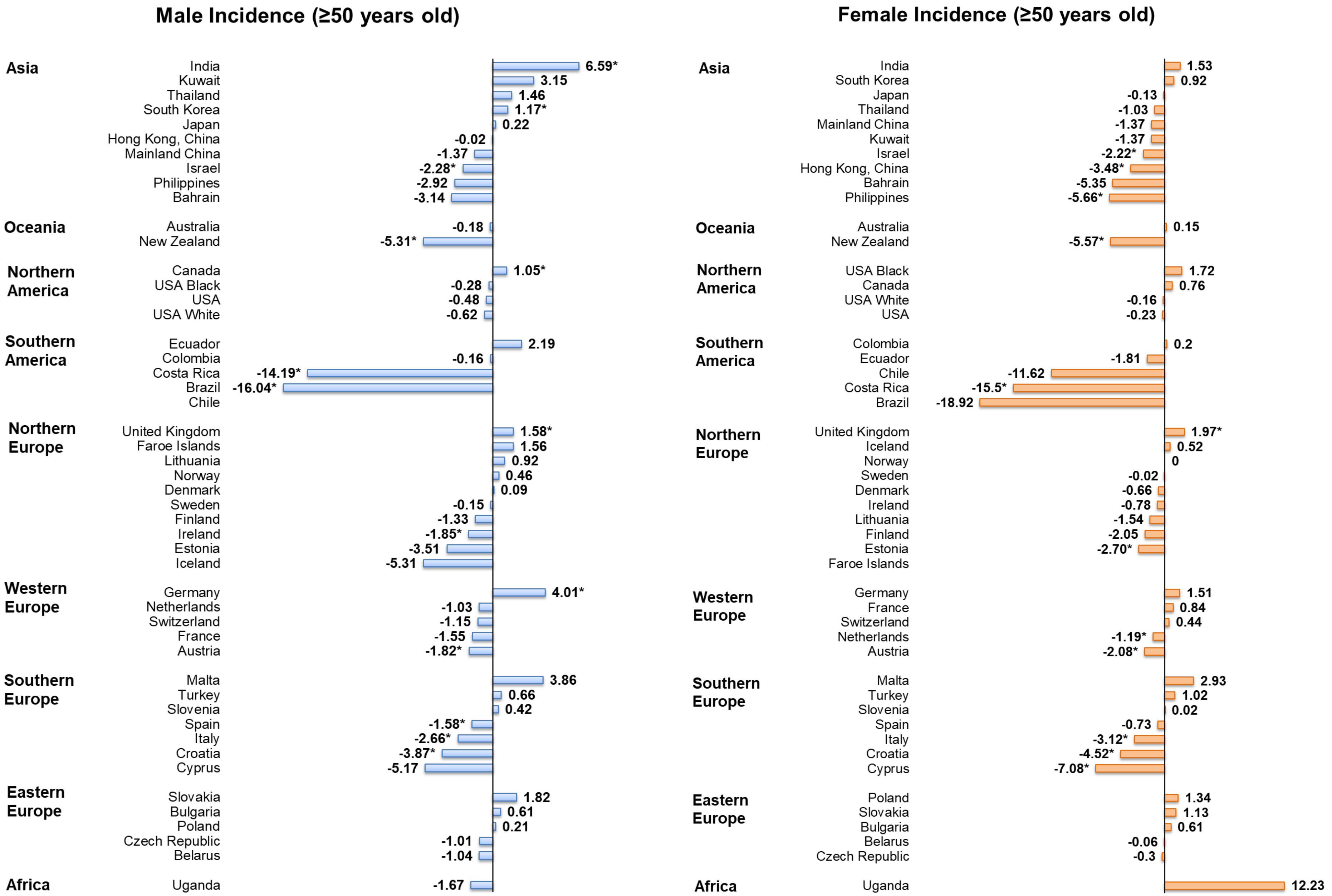

AAPC, annual percentage change; **p* values less than 0.05.

Supplementary Figure 4. AAPC of incidence of leukaemia aged < 40 years old

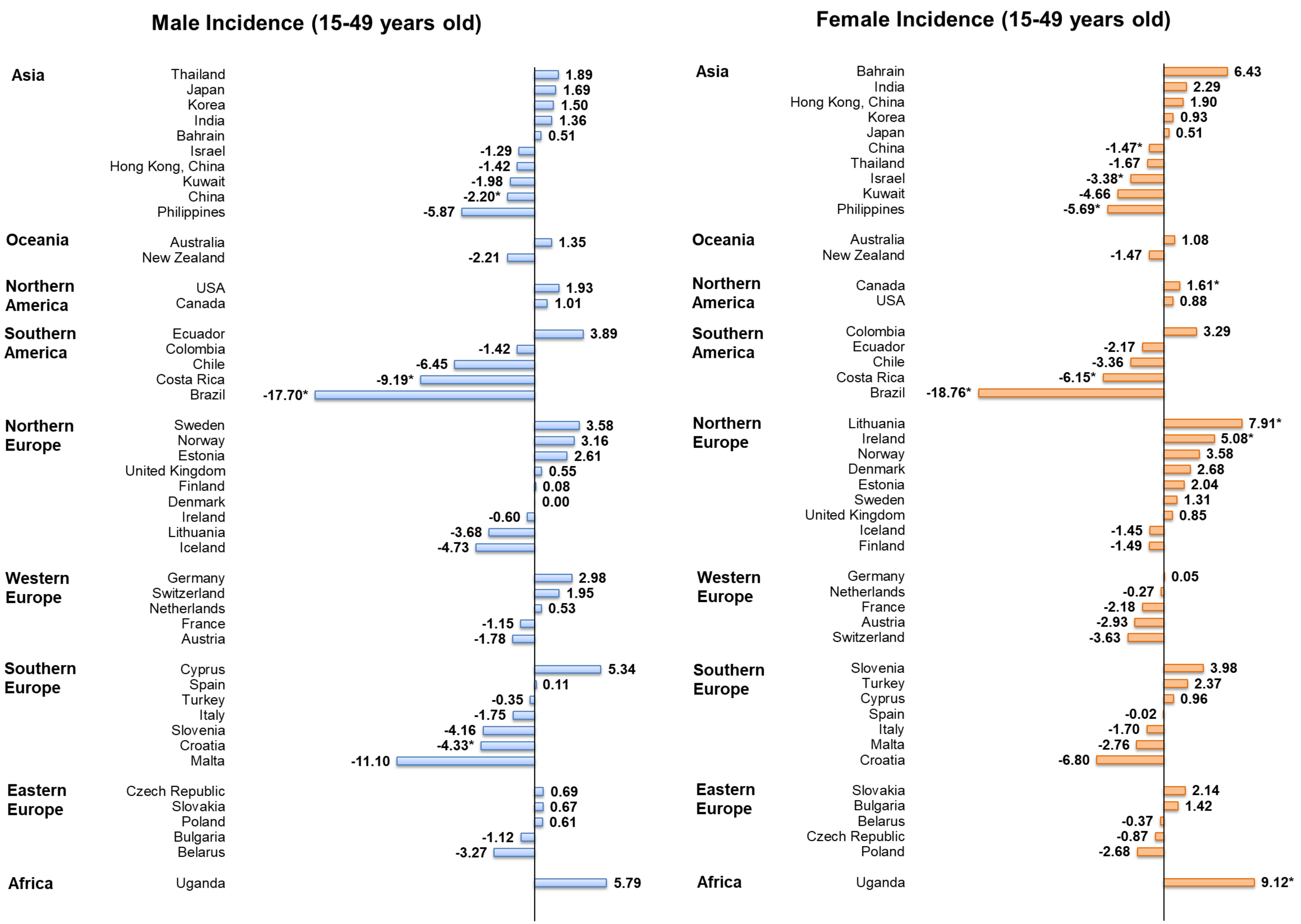

AAPC, annual percentage change; *p values less than 0.05.

Supplementary Figure 5. AAPC of incidence of leukaemia aged < 15 years old

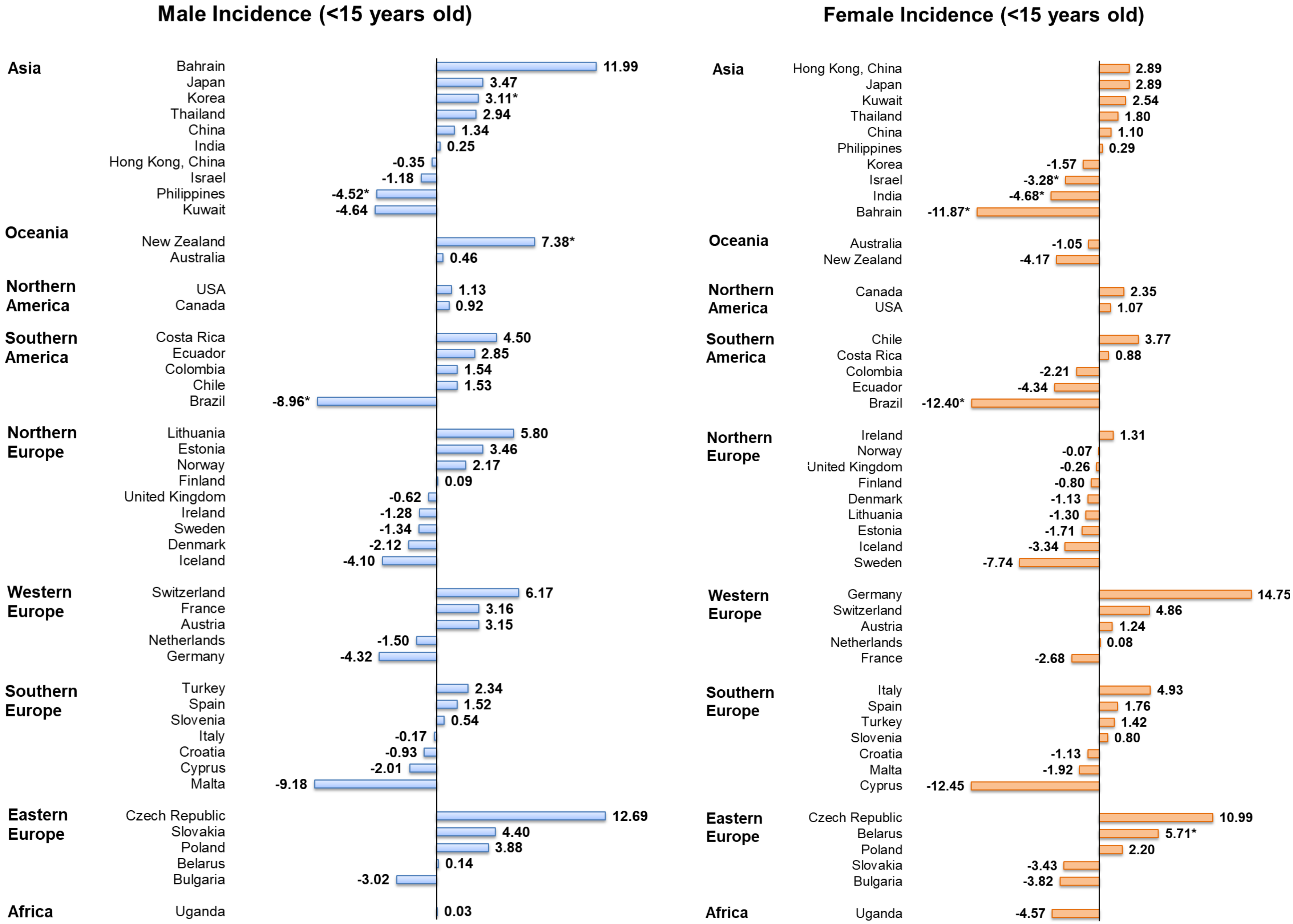

AAPC, annual percentage change; *p values less than 0.05.
